# Pyramidal cell types and circuit organization of the mouse insular cortex reveal functional specializations

**DOI:** 10.1038/s41593-026-02391-0

**Published:** 2026-08-07

**Authors:** Bart C. Jongbloets, Yang Chen, Michael A. Muniak, Ian K. Gingerich, Kayla A. Maanum, Tianyi Mao

**Affiliations:** 1 Vollum Institute, Oregon Health & Science University, Portland, OR USA; 2 Division of Cell Biology, Neurobiology and Biophysics, Department of Biology, Utrecht University, Utrecht, the Netherlands; 3 Neuroscience Graduate Program, Oregon Health & Science University, Portland, OR USA; 4 Behavioral and Systems Neuroscience Graduate Program, Oregon Health & Science University, Portland, OR USA; 5 Present Address: Department of Cellular and Circuit Neuroscience, Swammerdam Institute for Life Sciences, Amsterdam Neuroscience, University of Amsterdam, Amsterdam, the Netherlands; 6 Present Address: Geisel School of Medicine, Dartmouth College, Hanover, NH USA

**Keywords:** Neuroscience, Cellular neuroscience

## Abstract

The insular cortex integrates interoceptive and exteroceptive information to mediate bodily homeostasis, emotion and learning. However, the cellular and circuit substrates governing insular functions are poorly understood compared to primary cortices. Here we quantify dendritic morphology together with projections, electrical properties and/or local inputs of 1,130 mouse insular pyramidal neurons. Neurons are mapped onto a quantitative Nissl-derived model of the insula. Using improved algorithms, we define 21 morphological, 12 electrical and 9 input neuronal types, some unique to the insula. Several types exhibit elaborate dendrites, reminiscent of primate cortical neurons. Furthermore, morphological properties constrain and often predict inputs, electrical properties, projection targets or molecular markers. Eight morphological types are differentially distributed between the functionally distinct anterior and posterior insula, facilitating a quantitative demarcation between these subregions. Certain types receive intra-insular excitatory inputs originating far beyond cortical columns, which functionally bridge a long-range thalamus-to-amygdala circuit linking sensory information to valence behaviorally. Our work establishes a structure-and-function foundation for investigating the insula.

## Abstract (web summary)

A multimodal cell-type atlas of the mouse insular cortex reveals specialized pyramidal types and circuits in distinct insular subregions, as well as intra-insular functional connections that integrate sensory inputs with valence.

## Main

The insular cortex (IC), an evolutionarily conserved associative cortex, is an integration hub^1–3^ that orchestrates diverse functions, such as external and internal sensory perception, bodily homeostasis, learning, motivation and self-awareness and emotional awareness^4–7^. Knowledge of the neural substrates underlying insular functional diversity is in its infancy, but one major theme is that functional differences originate from anatomically defined insular subregions. In humans, the anteriodorsal, anterioventral and midposterior insula predominantly mediate cognition, emotions and sensorimotor integration, respectively^8,9^. In rodents, anatomical subdivisions also prominently correspond with functional domains despite gross anatomical differences between these species. Although certain functions are shared across its span^7,10–12^, the rodent anterior insula typically governs cognitive functions^13,14^, whereas the posterior insula primarily integrates external and internal sensory signals^7,11,15,16^. It is interesting that, although distinct, functions distributed along the insula’s anterior–posterior axis are often related^4,17^. In humans, the anterior and posterior ICs have been suggested to govern perceptive and sensory dimensions of nociception, respectively^17^. Furthermore, temporally synchronized electrical activity triggered by nociception arises within multiple locations of the IC along the posterior–anterior axis in both humans and rodents^18,19^, implying that these related functions are integrated via intra-insular connectivity^4,17,20^.

Neuroanatomical heterogeneity also contributes to the diverse roles of the IC. Conserved between mice and humans, the IC has a noncanonical layer composition containing agranular, dysgranular and granular subregions (AI, DI and GI, respectively), based on the absence, emergence and full presence of cortical layer 4 (L4)^6,21,22^. Functional diversity may also be associated with its differential long-range projections^3,23^, for example, to the nucleus tractus solitarii (NTS) for motivational vigor^13^, nucleus accumbens (NAc) and contralateral insula for social behaviors^24,25^ and mediodorsal (MD) and parafascicular thalamus (PF) for cognition, associative functions and pain processing^2,26^. In addition, IC projections to the basolateral (BLA) and central amygdala (CeA) are linked to taste processing and anxiety^10,14^, and aversive state processing and satiety^11,27^, respectively. These projections primarily originate from anatomically distinct insular subregions, with neurons projecting to the MD, parafascicular and posterior thalamus (PF/PO), NTS, NAc core and anterior BLA residing in the anterior IC and neurons projecting to the CeA residing in the posterior IC^1,11,13,26^. However, the neuronal substrates that underlie heterogeneity of function and information processing in the IC remain poorly characterized.

Pyramidal neurons and their local connectivity form the backbone of cortical computation and are crucial for the functional specialization of cortical subregions^28–30^. Comprehensive cell typing of pyramidal neurons based on anatomical properties, dendritic morphology and electrical physiology have revealed how primary cortices function^30–35^. However, thorough cell typing is not yet available for most associative cortices, including the IC. Existing knowledge of insular neuronal types has largely stemmed from qualitative morphological descriptions. In primates, von Economo neurons and fork cells—defined by their distinct and stereotypical morphologies—are found exclusively in the insula and anterior cingulate cortex^36^, whereas evidence of analogous neuronal types in mice remains limited^37^. Furthermore, in addition to communicating with different input–output regions, distinct cell types may also form unique local connections to achieve specific information processing within individual cortical regions. Yet, the intricate associations between local connections and other cell-type properties are typically not available comprehensively in primary cortices^38^ and remain largely unknown for associative cortices.

Here we systematically examined the cell-type identities in the mouse IC from a dataset of 1,130 quality-controlled pyramidal neurons. We registered neurons to a topographic atlas of the insula and defined pyramidal types that exhibit distinct morphologies, long-range output targets, electrical properties and local inputs. Intriguingly, certain morphological features of layer 5 (L5) projection types resembled their analogs in primates and were much more elaborate than counterparts in other mouse cortices. Supporting functional heterogeneity, compositions of pyramidal cell types differed along the anterior–posterior axis of the IC. Certain cell types received noncanonical, laterally extending, local excitatory inputs to facilitate intra-insular information propagation. Leveraging our multimodal dataset, we predicted molecular markers for several morphology types, identified biologically relevant correlates of structural and functional hallmarks at the cellular level and provided tools to identify insular cell types in future studies. Functionally, we found that intra-insular posterior–anterior excitatory circuits bridge a thalamus–amygdala pathway critical for coupling taste and potentially interoception with sensory valence. Together, our study provides insights into the neuronal substrates of functional diversity in the insula and analytical tools for cell-type identification.

## Results

### Anatomical hallmarks of the IC suggest a diverse circuit architecture

**Fig. 1:**
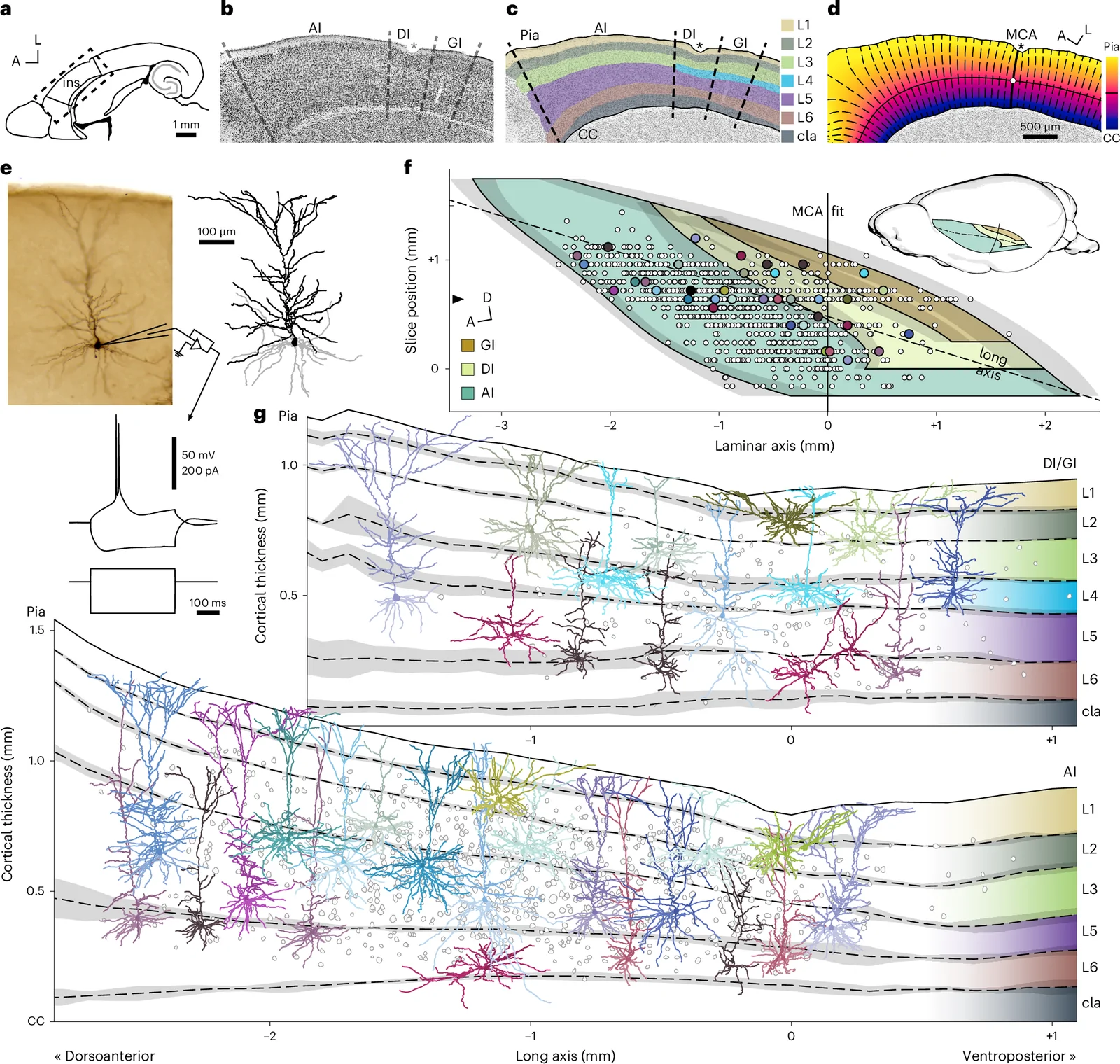
Anatomical hallmarks of the IC suggest a diverse circuit architecture. **a**–**c**, Example horizontal section (**a**) centered on the IC with Nissl staining (**b**) and manual annotations (**c**; Supplementary Fig. 1). Dashed lines indicate subregion boundaries and the asterisk the middle cerebral artery (MCA) position. Color blocks in **c** indicate manual cortical layer delineations between the pia and corpus callosum (CC) boundary lines (solid). **d**, Solution to Laplace’s equation on the same section from **b** and **c**. The thin black line is the trajectory of laminar cortical axis, the white circle the MCA-derived coordinate origin for section (Extended Data Fig. 1 and Methods), the solid streamline the MCA reference and the dashed streamlines the Laplacian gradients at 100-µm intervals relative to the MCA reference. **e**, Photomicrograph (top left) of an example DAB-stained neuron from an acute slice after physiology characterization (responses to current injections, bottom) and biocytin fill, with post hoc morphological reconstruction (top right) showing apical (black) and basal (gray) dendrites. **f**, Topographic bounds of insular subregions (filled contours) and the s.d. of their laminar boundaries (gray shading) represented on a 2D flatmap (Extended Data Fig. 1). The solid line shows the MCA fit (Extended Data Fig. 1b) and the dashed line the long axis (Extended Data Fig. 1d). The black triangle on the *y* axis shows the position of example Nissl section from **b**–**d**. Inset: approximate position of flatmap on whole mouse brain (adapted from ref. ^70^). **g**, Topographic distributions of recorded insular neuronal somas (gray outlines) and selected examples of reconstructed dendrites in the DI/GI (top) and AI (bottom) in their assigned cortical layers (Extended Data Fig. 1h). Sampled cells from **e** and **g** are highlighted with circles of corresponding colors in **f**. Layer boundaries, calculated based on flatmaps from three animals, are shown as the mean (dashed lines) and s.d. (gray shading) (Extended Data Fig. 1e,h). Panel **a** adapted with permission from ref. ^44^, Elsevier. cla, claustrum; ins, insula.

**Extended Data Fig. 1:**
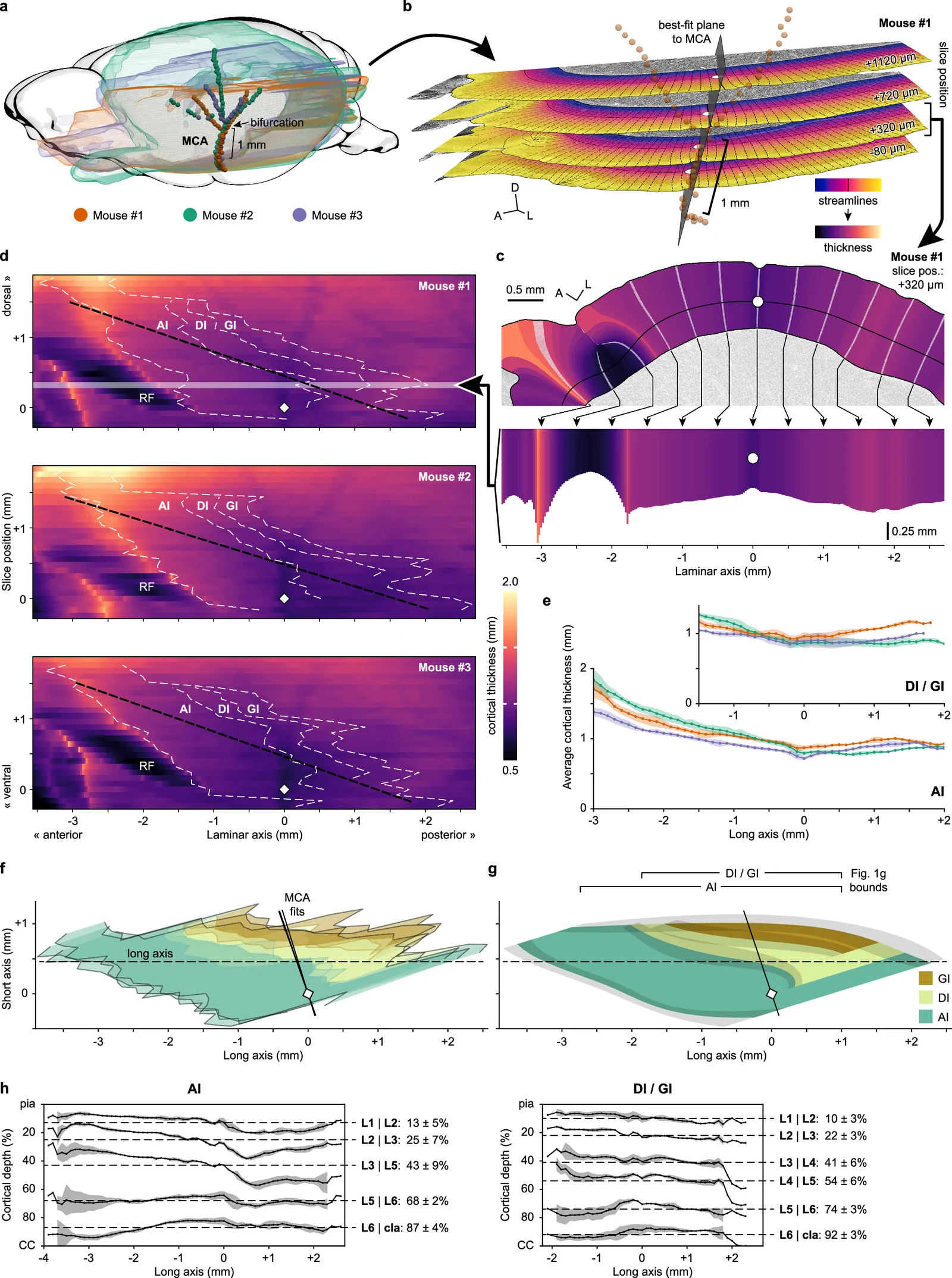
Construction of an average insular 2D flatmap. **a**, 3D reconstructions from brain sections in Supplementary Fig. 1 are registered to a single coordinate space. The excellent spatial alignment of the proximal MCA across three animals (Mouse #1, #2, and #3; colored spheres), along with its proximity to the insular cortex, motivated its utility as an anchoring point for organizing spatial data. Model brain adapted from Janke et al.^70^ was used for illustration only. Visualization was mirrored to the left hemisphere for consistent display orientation. **b**, Four example sections from Mouse #1 (Supplementary Fig. 1) shown in 3D space with Laplacian streamline overlays (Fig. 1d). White circles: the points of intersection between the Laplacian isocontour lines and the vertical 2D plane (gray) fit to the proximal MCA annotations (orange spheres; Methods). **c**, Estimated cortical thickness across the laminar axis on an example section from Mouse #1. Arrows (displayed at 0.5 mm intervals) show the relative mapping of 25-µm wide streamline bins (white lines) from the physical section (top) to a ‘flattened’ laminar axis plot (bottom). White circles: MCA-derived coordinate origin. **d**, Individual flatmaps of the right insular cortex for the three animals from (**a**). Each flatmap is a grid of 25-µm horizontal × 80-µm vertical voxels. The 2D coordinate origin (white diamonds) corresponds to the MCA position on the ventral-most Nissl section containing layer 4 (Supplementary Fig. 1). Vertical axis (that is, ‘cutting axis’) represents tissue sections; horizontal axis represents laminar distance from MCA origin (Fig. 1d). Luminance indicates cortical thickness (that is, streamline length in (**c**)). The relatively dark zone at the lower-left of each map reflects the indented contour of the rhinal fissure (RF). White dashed lines: annotated boundaries of insular subregions; black dashed line: long axis of an ellipse fit to the outermost boundaries of the insular annotations; thick arrowhead and semitransparent bar: row in Mouse #1 corresponding to data shown in (**c**). **e**, Mean cortical thickness in the AI (bottom) or DI/GI (top) for each mouse, binned along the long axis. Shaded regions: standard deviation. **f**, Overlay of insular flatmap subregion boundaries from all three mice. Data are aligned by rotating each flatmap about its MCA coordinate origin (white diamond) such that the long axis (dashed line) is horizontal. Solid black lines: trajectories of the best-fit planes to each animal’s MCA. **g**, An average 2D flatmap of the insula was generated by spatially averaging and smoothing (cubic spline; Methods) each set of lines from (**f**). Gray shading: smoothed standard deviation. A, anterior; D, dorsal; L, lateral; AI, agranular insula; DI, dysgranular insula; GI, granular insula; MCA, middle cerebral artery; RF, rhinal fissure. **h**, Grand mean of layer boundaries for AI (left) and DI/GI (right). Dashed lines: subregion-averaged cortical depth values (shown at right), obtained by subsequent averaging across the long axis. Shaded regions: standard deviation. AI, agranular insula; DI, dysgranular insula; GI, granular insula; MCA, middle cerebral artery; cla, claustrum; CC, corpus callosum.

**Extended Data Fig. 2:**
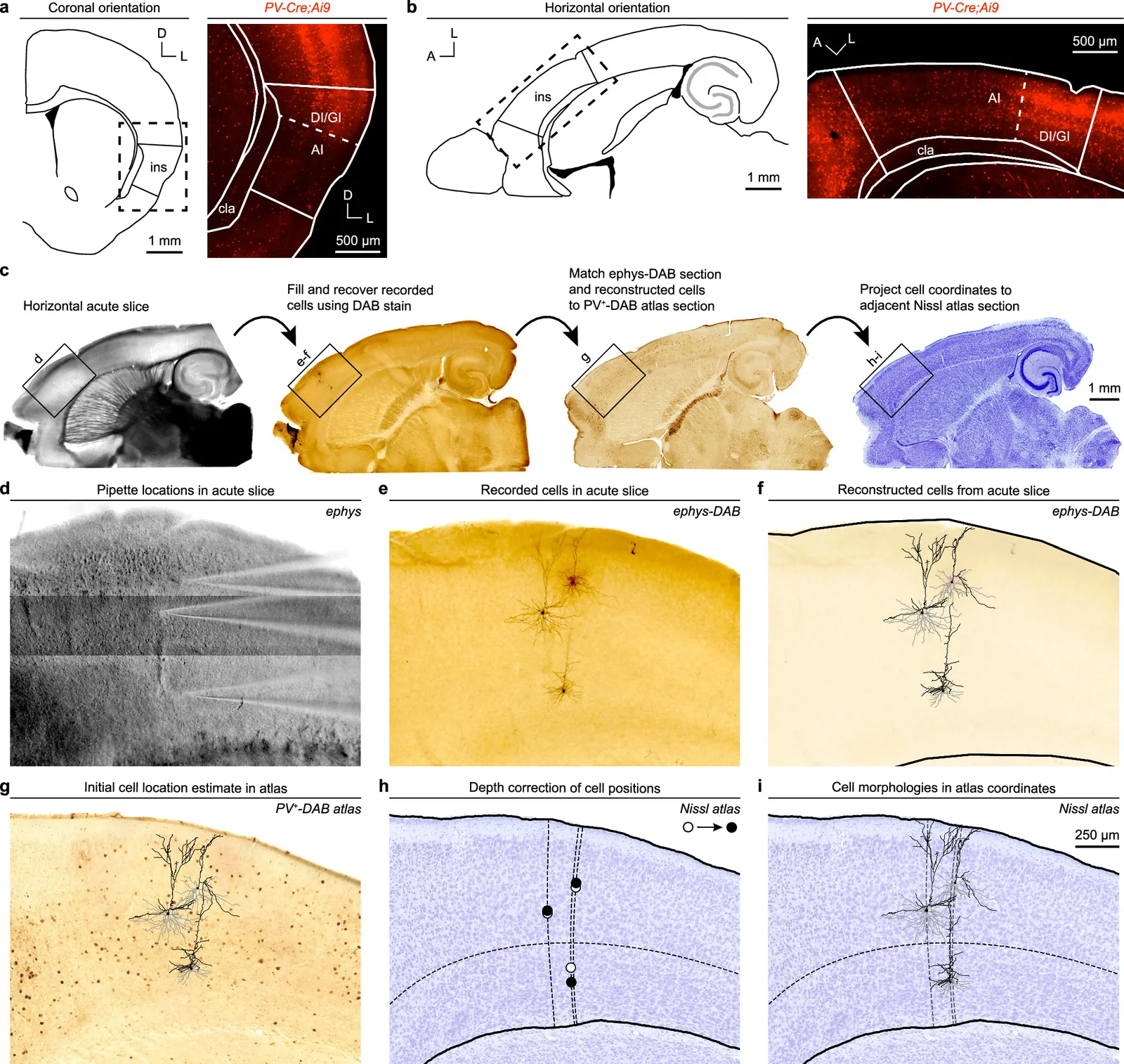
Registration of recorded cells from acute slices to the Nissl atlas. **a** and **b**, Example coronal (**a**) and horizontal (**b**) sections from *PV-Cre*^*+/−*^*;Ai9*^*+/−*^ mice highlighting the paucity of PV^+^ somas in the AI. Dotted boxes on schematics: image locations. **c**, Overview of registration procedure. **d**, Photomontage of pipette locations for three separate electrophysiology recording sessions from the same acute slice. **e**, Recovery of dendritic morphologies from (**d**) following DAB staining. **f**, Reconstructions of cells from (**e**). Solid lines: pia (top) and CC (bottom) annotations. **g**, Estimated positions of cells from (**e** and **f**) on matched PV^+^-DAB atlas section following alignment. **h**, Estimated positions of cells from (**e** and **f**) on Nissl atlas section following ventral projection from adjacent PV^+^-DAB atlas section, both before (open circles) and after (filled circles) correction of cortical depth (Methods). **i**, Cell morphologies in final atlas coordinates. Vertical dashed lines: streamlines intersecting cell positions; horizontal dashed line: mid-value Laplacian isocontour. Panels **a** (left) and **b** (left) adapted with permission from ref. ^44^, Elsevier.

The IC contains subregions of distinct cytoarchitecture marked by the gradual emergence of a granular L4—AI, DI and GI—which may support the integration of diverse input–output networks^1^. To contextualize their underlying cellular types and local circuit connections, we generated an anatomically quantifiable framework of the IC. To register our large-scale dataset onto a precisely defined common reference that accurately captured the subregional and layer demarcations in the IC (Fig. 1a–d and Extended Data Figs. 1 and 2), we leveraged distinctions in parvalbumin-positive (PV^+^) neuron density between the AI and the combined DI and GI (DI/GI) (Extended Data Fig. 2a,b)^22^, as well as cortical layer organization revealed by Nissl staining (Fig. 1b). Annotated Nissl sections (Fig. 1a,c and Supplementary Fig. 1) were transformed into insular flatmaps to enable quantitative comparisons and averaging across sections and animals (Fig. 1d, Extended Data Fig. 1 and Methods). Derived from these flatmaps, the insular long axis—the widest breadth of its bounds—provided a close approximation for the functional gradient along the anterior–posterior axis (Extended Data Figs. 1 and 2 and Supplementary Figs. 2 and 3). Strikingly, cortical thickness (pia to corpus callosum) of the AI nearly doubled from posterior (888 ± 35 µm, mean ± s.d.) to anterior (1,641 ± 235 µm) along a 5-mm stretch of the long axis (Extended Data Fig. 1e), which covaried with a near inversion of the relative thickness of superficial (L1–L3; anterior: 32 ± 4%; posterior: 54 ± 3%) versus deep (L5–L6; anterior: 62 ± 4%; posterior: 34 ± 1%) layers (Extended Data Fig. 1h; local claustral thickness included in calculation). Cortical thickness of the DI/GI increased only moderately toward the anterior direction (17 ± 23% across a 3-mm stretch), primarily driven by a relative expansion of L5. Importantly, we established subregion-specific layer demarcations for AI and DI/GI (Extended Data Fig. 1h and Methods). Overall, the marked differences across subregions and along the long axis suggested the potential presence of specialized circuit architecture across subregions and functional axes, emphasizing the necessity to register our cell atlas within this three-dimensional (3D) topography.

### Generation of a position-mapped multimodal neuronal dataset

To investigate insular cell types, we recorded the electrical activities of pyramidal neurons in acute insular brain slices in a modified horizontal orientation using whole-cell patch clamp with biocytin-filled electrodes (Fig. 1e and Methods). We reconstructed dendritic morphologies and registered them to our common reference framework (Fig. 1e and Extended Data Fig. 2c–f). Soma locations were subsequently placed onto the insular flatmap (Fig. 1f and Supplementary Figs. 2 and 3), highlighting comprehensive coverage across insular subregions and layers (Fig. 1f,g). Mapping our cell atlas to this common reference framework set the stage for investigations on the relationship of IC cell types with the anatomical and functional topography of the insula.

### Insular pyramidal neurons display distinct morphological types

**Extended Data Fig. 3:**
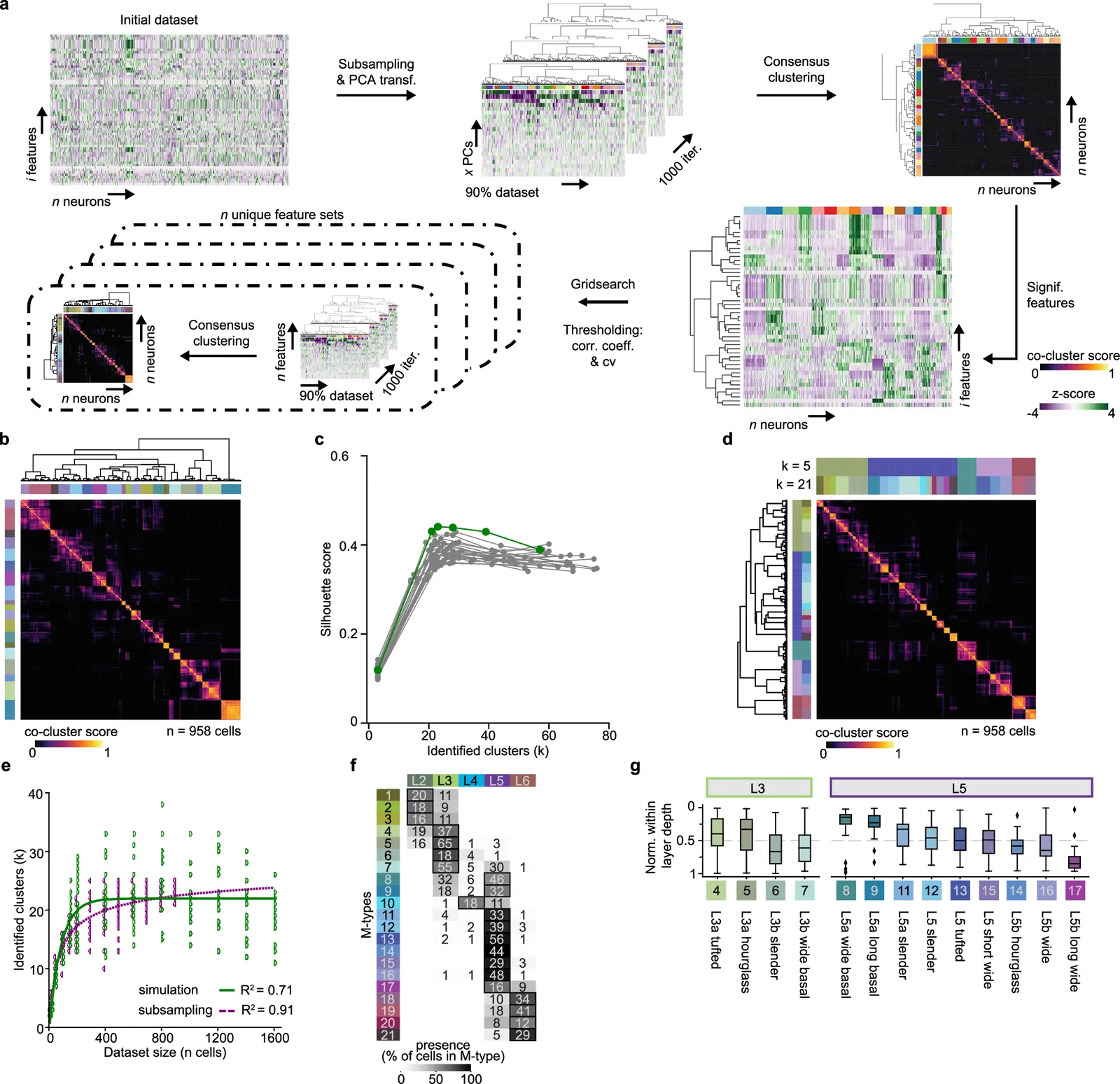
Iterative consensus cluster analysis pipeline and evaluation of its performance. **a**, Analysis pipeline of iterative consensus clustering analysis (iCCA) using neuronal properties (that is, features; Methods). **b**, Hierarchical cluster analysis (HCA) on pair-wise co-cluster scores across 958 cells using PCA-transformed morphology dataset initially identified 20 morphology clusters (different from the final 21 M-types), color-coded. **c**, Feature set performance as described by the silhouette score and number of identified clusters (k) for each tested unique feature set (individual curves) at different dendrogram cut levels (3 clusters and 5 ‘deepsplit’ levels; Methods). The best-performing feature set is highlighted (green). **d**, Hierarchical cluster analysis (HCA) on pair-wise co-cluster scores across 958 cells using the best performing morphological feature set. Dendrogram-based grouping is color-coded for 21 clusters (k = 21) based on optimal silhouette score, as well as for 5 clusters (k = 5). **e**, Number of identified clusters as a function of dataset size based on iterations of HCA on subsampled data (purple) or simulated data (green). Solid and dashed lines: logistic regression fit on subsampled and simulated data, respectively. **f**, M-type cell counts by layer. Horizontal colored boxes: layer identities. Vertical colored boxes: M-type aliases sorted by median soma location, color-coded corresponding to k = 21 results in (**d** and Fig. 2a). Number in gray boxes: cell counts per layer per M-type. Grayscale luminance indicates the percentage of counts per layer for each M-type. The predominant layer allocations are boxed (presence > 45%). **g**, The within-layer locations of several morphology types are biased to either the upper or lower half of the layer, thereby allocating them into L3a vs. L3b, or L5a vs. L5b morphology types. Number of observations for all groups provided in Supplementary Table 11. Boxplots (**g**) use the following convention: center line, median; box limits, upper and lower quartiles; whiskers, 1.5x interquartile range; diamonds, outliers.

To identify insular pyramidal cell types, we classified neurons based on interpretable dendritic morphology features. We analyzed 958 high-quality dendritic reconstructions (Supplementary Fig. 4) and extracted a curated list of morphological features^33,35,39^, including the shape and length of local dendritic arbors relative to the soma or pial surface, along with additional components to capture spatial dendritic complexity (Supplementary Table 1). We performed iterative consensus cluster analysis (iCCA) to select nonredundant interpretable features, achieving high co-cluster scores for robust clustering (Extended Data Fig. 3a–d and Methods). To assess whether our sample size was adequate, we repeated the iCCA on subsampled original data or simulated data (Extended Data Fig. 3e and Methods). Both approaches showed cluster stability beyond 800 cells, suggesting that our data size was sufficient to capture putative insular cell types.

**Fig. 2:**
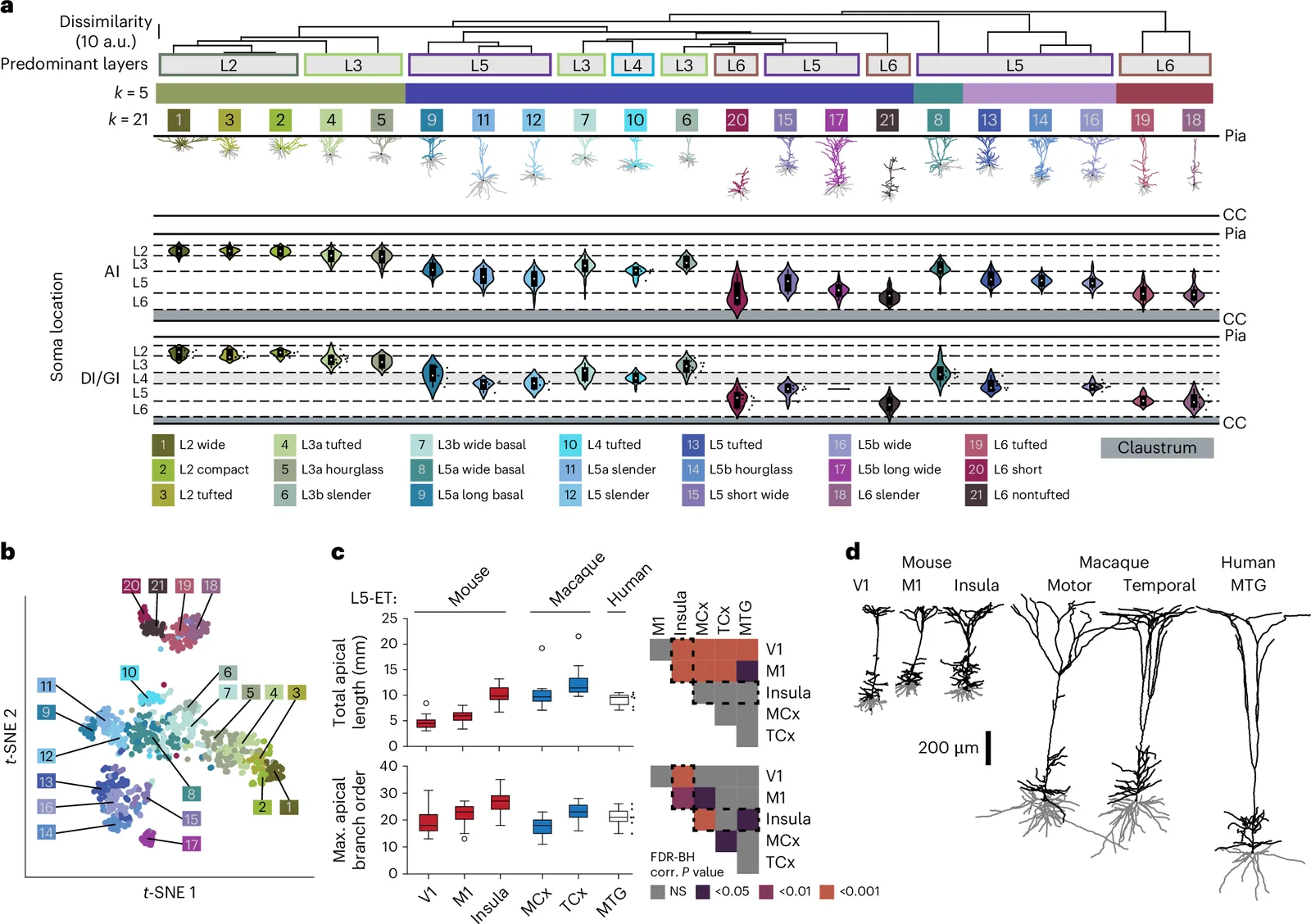
Insular pyramidal neurons display distinct morphological types with complex dendritic arborization. **a**, Predominant layer(s) and hierarchical dendrogram allocations of M-types (top) with representative morphologies (middle) and soma distributions in the AI and DI/GI as violin plots with embedded boxplots (bottom). **b**, The *t*-SNE distribution of M-types. Colored boxes with numbers are M-types color coded for *k* = 21 in **a**. **c**,**d**, Comparative analysis of L5-ET projection neurons across species and cortical regions. **c**, Left: quantification of total apical length (top) and maximal apical branch order (bottom) per cortical region in mouse (V1; M1, primary motor cortex; insula), macaque (MCx, motor cortex; TCx, temporal cortex) and human (MTG, middle temporal gyrus). Right: corresponding heatmaps of *P* values from pairwise comparisons with false discovery rate Benjamini-Hochberg correction (FDR-BH corr.; Methods). Dashed rectangles show pairwise comparisons with mouse insular L5-ET. **d**, Representative morphologies included in the comparative analysis. Noninsular L5-ET neurons were reproduced from publicly available datasets^33,41–43^. Boxplots display median as center dots (**a**) or center lines (**c**). The box limits indicate the upper and lower quartiles, the whiskers 1.5× the interquartile range (IQR) and circles the outliers. The number of observations (*n*) for boxplots (**a**, bottom and **c**) and exact *P* values are provided in Supplementary Tables 6 and 7, respectively. Individual points are shown for groups with *n* ≤ 10 (black dots). Max., maximum; NS, not significant.

**Extended Data Fig. 4:**
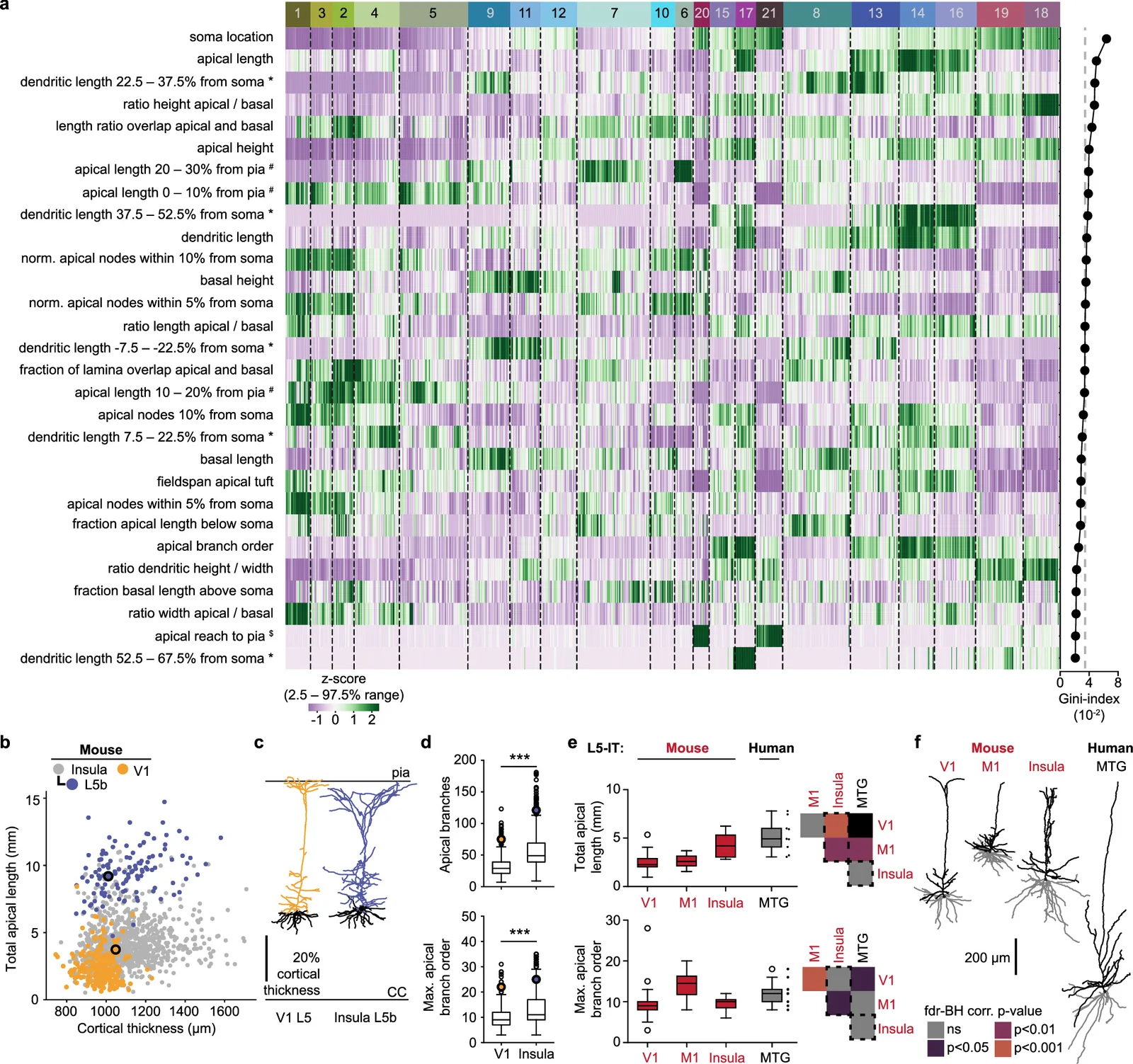
Insular M-types display hallmark dendritic features that are distinct from pyramidal neurons in the primary cortices. **a**, Z-scored morphology features (left), ranked by random forest classifier Gini-index (right, gray dashed line indicates equal feature contribution [1/29 features]). *: Dendritic length located within a 15% local cortical thickness bin positioned relative to the soma, normalized to local cortical thickness. ^#^: Apical length within a 10% local cortical thickness bin positioned relative to the pia, normalized to total apical length. ^$^: Vertical distance between pia and closest apical dendrite, normalized to local cortical thickness. Colored boxes with numbers: M-types color-coded for k = 21 as in Fig. 2a. **b**–**d**, Morphometric comparison of insular and V1 pyramidal neurons in mouse. Exemplar neurons are highlighted with black circles (**b** and **d**) and displayed in (**c**). **b**, Distribution of total apical length over local cortical thickness shows no linear correlation for insular (grey- and blue-filled circles, n = 958 cells, Pearson’s correlation coefficient = 0.0794, p = 0.0139) and V1 (orange-filled circles, n = 249 cells, Pearson’s correlation coefficient = −0.0694, p = 0.2752; data from Gouwens et al.^33^ pyramidal neurons. The L5b insular neurons (blue-filled circles) display long apical dendrites. **c**, Exemplar morphologies of V1 L5 and insular L5b neurons, as highlighted in (**b** and **d**), normalized to local cortical thickness. **d**, Number of apical branches (Mann-Whitney two-sided U = 191395, p = 4.55·10^−49^) and the maximal apical branch order (Mann-Whitney two-sided U = 159157.5, p = 3.27·10^−16^). **e** and **f**, Comparative analysis of L5 intratelencephalic (L5-IT) projection neurons across cortical regions and between mouse and human (two-sided Mann-Whitney U test). **e**, Left: total apical length (top) and maximal apical branch order (bottom) of L5-IT neurons per cortical region in mouse (red; V1, primary visual; M1, primary motor; insular cortex) and human (black; MTG, middle temporal gyrus). Right: corresponding heatmaps of p-values from pair-wise comparisons (Kruskal-Wallis test with post-hoc Dunn’s test with false-discovery rate Benjamini-Hochberg (fdr-BH) correction). Dashed rectangles highlight the pair-wise comparisons with mouse insular L5-IT neurons. **f**, Exemplar morphologies of mouse V1, M1, and insular neurons and a human MTG neuron, as highlighted in (**e**). Number of observations (n) and exact p-values for boxplots (**e**) provided in Supplementary Table 12; individual points are shown for groups with n ≤ 10 (black dots). CC: corpus callosum. The boxplots (**d**, **e**) use the following convention: center line, median; box limits, upper and lower quartiles; whiskers, 1.5x interquartile range; circles, outliers.

Our morphology feature-based iCCA selected 29 features and yielded 21 morphology types (M-types) (Supplementary Table 2 and Supplementary Fig. 5), comparable to the number of M-types in other cortical areas^33,35,39^. Reducing cluster numbers (for example, *k* = 3, 5 or 14) resulted in groupings of cells that were highly heterogeneous in layer location and morphology (Fig. 2a and Supplementary Fig. 6a). We named these M-types based on their predominant layer allocation and morphological characteristics (Fig. 2a,b, Extended Data Figs. 3f,g and 4a and Supplementary Fig. 6). In addition, we trained random forest classifiers using subsets of the original data with the selected 29 morphology features for iCCA and achieved ~84.0% accuracy (median *F*_1_ score, range 82.9–85.3%, *n* = 5 iterations; Supplementary Table 2 and Methods) on the withheld dataset (‘M-features × M-types’, Supplementary Figs. 7a and 8a). Thus, these M-type classifiers are reliable tools that require a minimal list of features to predict dendritic morphologies.

Analyses of M-types and their associated morphological features revealed several remarkable characteristics (Fig. 2b–d, Extended Data Fig. 4 and Supplementary Fig. 6b). First, when visualized in two dimensions using *t*-distributed stochastic neighbor embedding (*t*-SNE; Methods), similar morphologies were located close together and cell types from different layers were separated (Fig. 2b). The separation of the larger L5 morphologies (M-types 14, 16 and 17)—primarily in deeper L5 (Fig. 2a and Extended Data Fig. 3g)—from the smaller L5 morphologies (M-types 8 and 9) highlighted the presence of canonical L5a and L5b pyramidal neurons (Fig. 2b)^40–42^. Consistent with empirical properties that are highly relevant in defining cortical pyramidal neuron types^33,35,39^, soma location, total apical length and the ratio of apical-over-basal dendritic height were among the most informative features in the classifiers (Extended Data Fig. 4a). In comparison to mouse primary visual cortex (V1) pyramidal neurons (Extended Data Fig. 4b–d), insular pyramidal neurons displayed larger apical dendritic length, branch number and maximal branch order. This larger apical length was not due to widening of the cortical thickness (Pearson’s correlation coefficient = 0.0794; Extended Data Fig. 4b). In particular, L5b M-types had total dendritic lengths extending over 10 mm (Extended Data Fig. 4b,c and Supplementary Fig. 6b), with no equivalents identified in the mouse V1 dataset using random forest classifiers trained on nonscaled insular morphology features (‘M-features × M-types’; Supplementary Figs. 7a, 8a and 9). The slender and wide apical tufted morphologies of L5a and L5b M-types are reminiscent of the classic L5 intratelencephalic (L5-IT; Extended Data Fig. 4e,f, cortical projecting) and L5 extratelencephalic (L5-ET; Fig. 2c,d, subcortical projecting) projection neurons, respectively^29,33,41^. We further compared L5-IT and L5-ET projection types, identified by retrograde bead injections in their target regions (L5-IT: contralateral AI; L5-ET: thalamus and medulla; Methods), to those reported in other mouse, macaque and human cortices (Fig. 2c,d and Extended Data Fig. 4e,f)^33,41–43^. Certain features, such as total length and maximal branch order, of apical dendritic morphologies of both insular projection types were distinct from those reported in other mouse cortices, but were more comparable to macaque and human L5-ET and L5-IT neurons^33,41,42^. The elaborated postsynaptic compartments in these insular neuronal types may enable integration from complex inputs.

### Insular M-types are enriched in subregions and have preferential projection targets

**Fig. 3:**
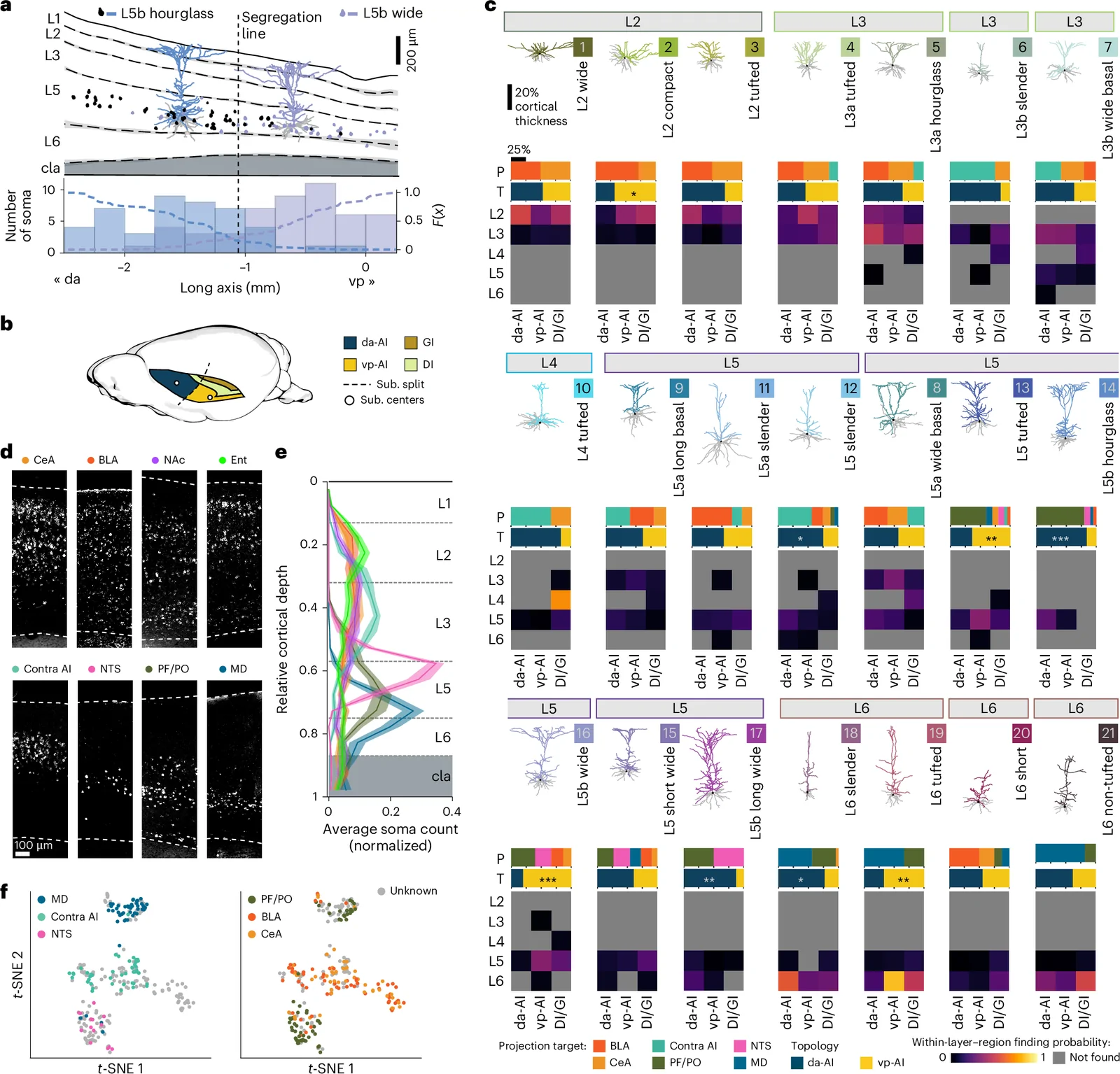
Insular M-types are enriched in subregions and have preferential projection targets. **a**, Soma distribution (top; Fig. 1g) and corresponding histograms (bottom) of two M-types (L5b hourglass and L5b wide). The colored bars and dashed lines (bottom) show the soma count (left, *y* axis) and cumulative distribution probability (right, *y* axis), respectively, along the long axis. The black dashed vertical line shows the segregation line at which the sum of cumulative distribution probability of the two M-types is the smallest. **b**, Three-dimensional representation of the topographic split location (dashed line) within a model mouse brain^70^. The white circles are suggested coordinates to target da-AI and vp-AI subregions. **c**, Quantification of projection targets (P, top bar plot), topography (T, bottom bar plot) and within-layer and within-region finding probabilities (bottom heatmap) per M-type. M-types are sorted based on predominant layer allocation and grouped by dendrogram hierarchy as shown in Fig. 2a. Within-layer and within-region finding probabilities show the number of neurons for a given M-type allocated to a specific layer and subregion (for example, L2 of da-AI) divided by the total number of neurons in that layer and subregion. Two-sided Fisher’s exact test with false discovery rate Benjamini–Hochberg (FDR-BH) correction: *P ≤ 0.05, **P ≤ 0.01, ***P ≤ 0.001. **d**,**e**, Representative photomicrographs (**d**) and normalized density (**e**) of retrogradely labeled neurons in the agranular IC projecting to the CeA, BLA, NAc, Ent, contra AI, NTS, PF/PO and MD, respectively. For **e**, averaged density of shaded region is the s.e. **f**, Projection target identities (colored dots) revealed in *t*-SNE representations of the morphology dataset (all dots), as shown in Fig. 2b. Number of observations for **c** and **e** and exact *P* values are provided in Supplementary Tables 8 and 9, respectively. norm., normalized; Sub., subregion.

**Extended Data Fig. 5:**
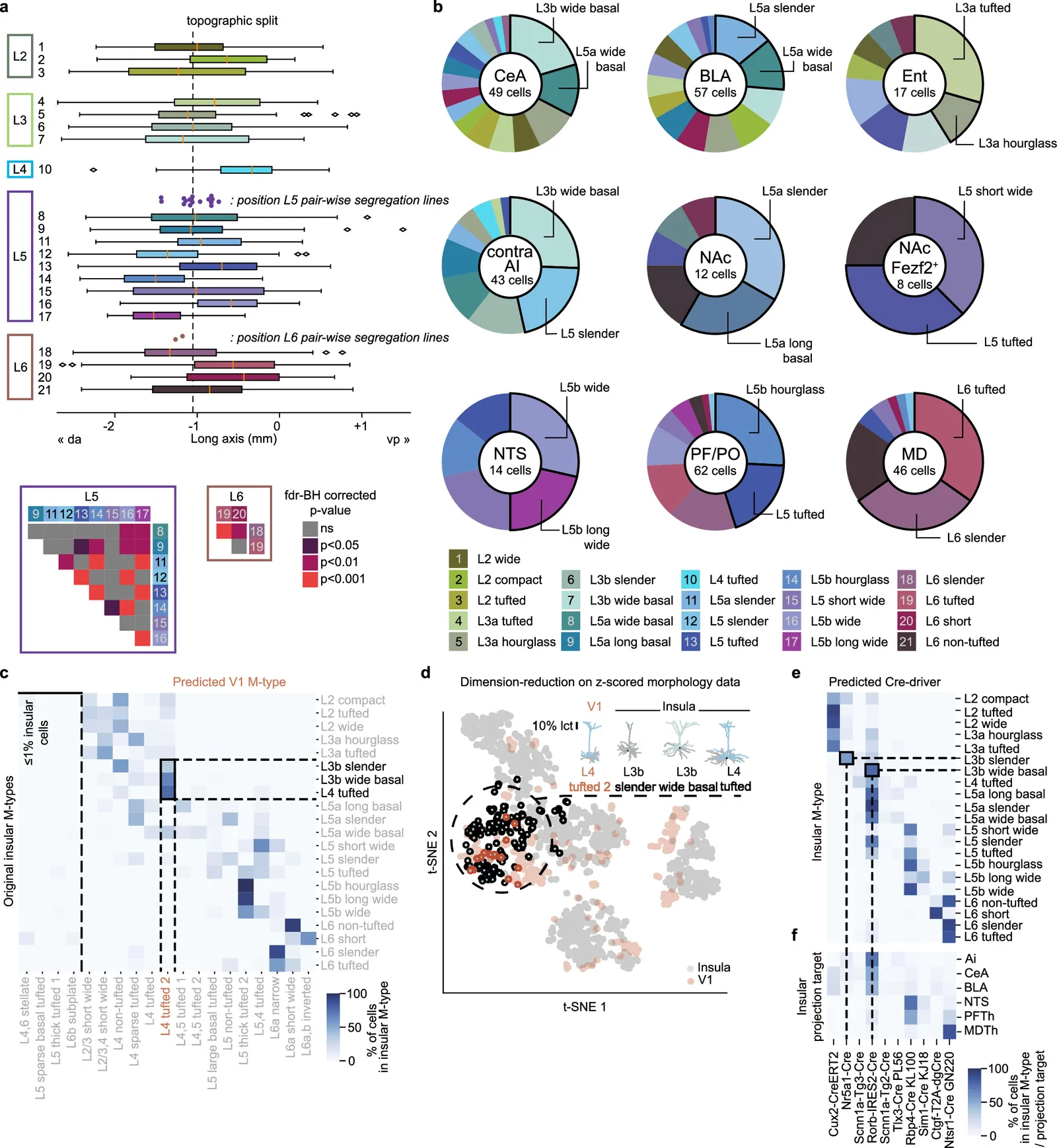
Topographic- and projection-based distributions and predicted transgenic identities of M-types. **a**, Topographic distribution of all M-types along the long axis of the insula (top), and the corresponding heatmaps of p-values from within-layer pair-wise comparisons in L5 and L6 (bottom, Mann-Whitney U two-sided test with false-discovery rate Benjamini-Hochberg (fdr-BH) correction). Note that all pair-wise comparisons within L2 and L3 were non-significant and, therefore, not shown here. Number of observations (n) for all groups provided in Supplementary Table 13. Color-filled circles: position of pair-wise segregation lines from all significant within-layer comparisons; open diamonds: outliers; dashed line: average position of segregation lines; orange ticks: median location for individual M-types. **b**, M-type composition of each projection target. The top two most-abundant morphology types are annotated. Note, the M-type identities of Ent- and NAc-projecting neurons were predicted based on a random forest classifier using their extracted morphology features (Supplementary Table 2; “M-features x M-types”, Supplementary Figs. 7a and 8a). CeA: central amygdala; BLA: basolateral amygdala; Ent: Entorhinal cortex; contra AI: contralateral agranular insula; NAc: nucleus accumbens; Fezf2^+^: *Fezf2-CreER* positive neurons; NTS: nucleus tractus solitarius; PF/PO: parafascicular/posterior thalamus; MD: mediodorsal thalamus. **c**, Matching of insular neurons to V1 M-types by classifiers trained on V1 data^33^. Black rectangle highlights the allocation of insular L3b M-types to V1 ‘L4 tufted 2’ M-type. Blue luminance: percentage of cells from a given insular M-type that are allocated to a V1 M-type by the classifier. **d**, t-SNE visualization of the z-score transformed morphology data from both the V1 (red filled-circles, n = 249 cells) and insular (grey filled-circles, n = 958 cells) datasets. The bona fide V1 L4 M-type intermingles with insular L3b and L4 M-types (black dashed circle). **e** and **f**, Predicted transgenic identities of insular M-types (**e**) and projection neurons (**f**). Black rectangles highlight the predicted L4-like transgenic identities (*Nr5a1-Cre* and *Rorb-IRES2-Cre*) of the L3b insular M-types. The boxplots (**a**) use the following convention: center line, median; box limits, upper and lower quartiles; whiskers, 1.5x interquartile range; diamonds, outliers.

As IC subregions associate with long-range input–output circuits and functions^1,7–11,13,15,16^, we hypothesized that specialized M-types may be enriched in these subregions to support their distinct roles. We therefore set out to map the insular M-types onto anatomical and projection target modalities (Fig. 3 and Extended Data Fig. 5a,b).

Although a major theme of functional diversity in the IC is its spatial separation along its anterior–posterior axis^4,9,23^, the precise demarcation is not well defined^10,11,13,14^. Given the posterior–anterior broadening of cortical and layer thickness along the long axis, especially within the AI (Fig. 1g and Extended Data Fig. 1), we tested whether M-types of the IC were differentially distributed across this axis. This was indeed the case—most notably, several M-types within the same layer, for example L5b hourglass and L5b wide, exhibited strong topographic preferences either toward dorsoanterior or ventroposterior subregions (Fig. 3a and Extended Data Fig. 5a). To systematically quantify this tendency for each individual M-type, we first identified an objective ‘topographic split’ line centered at −1,052 ± 48 µm (s.e.; *n* = 18 segregation lines; Methods)^8^ along the insular long axis (Fig. 3b and Extended Data Fig. 5a). As the AI stretched across most of the insula’s anterior–posterior axis, we used this topographic split to define dorsoanterior AI (da-AI) and ventroposterior AI (vp-AI). We registered the topographic split to the Franklin and Paxinos mouse atlas^44^ (Fig. 3b and Methods) and estimated the stereotaxic coordinates for targeting the da-AI (−2.56, 2.20, 2.55 mm; dorsal–ventral, anterior–posterior and lateral–medial relative to bregma) and vp-AI (−3.76, 1.00, 3.80 mm). Based on this topographic split (Extended Data Fig. 5a), eight M-types located in L2, L5 and L6 had statistically significant enrichment in either da-AI or vp-AI (Fig. 3c). The existence of distinct M-types in specific AI subregions implies specialized local insular subcircuits.

We next asked whether AI and DI/GI encompass different compositions of M-types. L5b hourglass and L5b long wide cells were found almost exclusively in the AI (Figs. 2a and 3c). L4 tufted neurons residing in the DI/GI resembled the typical L4 pyramidal neuronal morphology in the primary visual cortex^45^. Yet, spiny stellate cells, such as those in the somatosensory cortex, were not identified in the IC^45^. Notably, a sparse presence of L4 tufted neurons was also observed in the AI. In addition, L3b slender and L3b wide basal neurons located adjacent to L4 had morphological characteristics most similar to L4 tufted neurons (Fig. 2a,b and Extended Data Fig. 4a) and were predominantly in the da-AI (Fig. 3c). Indeed, M-type classifiers trained on the normalized mouse V1 dataset^33^ classified insular L4 and L3b neurons as L4 V1 M-types and this analysis further predicted two candidate transgenic mouse lines that may label these types (Nr5a1-Cre and Rorb-IRES2-Cre; Extended Data Fig. 5c–e, Supplementary Figs. 7b and 8b and Methods). Our topographic analysis, therefore, identified L4-like neurons and subregion-specific M-types in the AI.

To identify anatomical and morphological features of projection neurons in the IC, we quantified the soma distribution of IC neurons projecting to the CeA, BLA, MD, PF/PO, NTS, NAc, entorhinal cortex (Ent) or contralateral AI (contra AI) labeled by retrograde tracers. The soma distributions of neurons projecting to contra AI, Ent, NTS, PF/PO and MD showed clear layer preferences, whereas corticostriatal (that is, NAc-projecting) and corticoamygdalar neurons were distributed across L2–L6 (Fig. 3d,e). To identify morphological characteristics of projection neurons, we mapped our reconstructed morphologies with projection target information (*n* = 271 neurons; Supplementary Fig. 4) onto the *t*-SNE morphology feature space. Neurons with the same projection target, such as NTS-projecting or MD-projecting neurons, shared overlapping morphological traits (Fig. 3f). Projection targets also constrained M-types (Extended Data Fig. 5b). For example, MD-projecting neurons largely resembled L6 tufted and L6 slender M-types but rarely L6 short, which projected mainly to the amygdala. To showcase the utility of M-type classifiers, M-type predictions on an independent dataset of Ent-projecting or NAc-projecting neurons also showed general projection–morphology associations (Extended Data Fig. 5b). Specifically, the Ent-projecting neurons were allocated to M-types predominantly in superficial layers and most L5-located NAc-projecting neurons were L5a slender and L5a long basal. Several reports have shown correspondence between projection targets and transgenic markers, including *Satb2*, *Ctip2* and *Fezf2* (refs. ^13,25^). It is interesting that NAc-projecting neurons expressing *Fezf2* genes were enriched primarily in L5 short wide and L5 tufted, suggesting that molecular markers may further specify M-types (Extended Data Fig. 5b). To further investigate the association of morphology, projection targets and gene expression, we trained M-type classifiers on the normalized V1 mouse dataset with known molecular information^33^ and predicted transgenic mouse lines for contra AI-projecting, CeA-projecting and BLA-projecting neurons (*Rorb-IRES2-Cre*) and L5-ET (NTS and PF/PO versus MD projecting; *Rbp4-Cre* KL100 versus *Ntsr1-Cre* GN220) neurons (Extended Data Fig. 8f and Methods).

Together, we comprehensively identified insular pyramidal M-types that were associated with specific layers, topographic distributions and projection targets (Fig. 3c), which may underlie IC subcircuit diversity. In addition, we identified several molecular markers that may distinguish these insular pyramidal types (Extended Data Fig. 5e,f).

### Distinct electrical profiles associate with insular layers and M-types

**Fig. 4:**
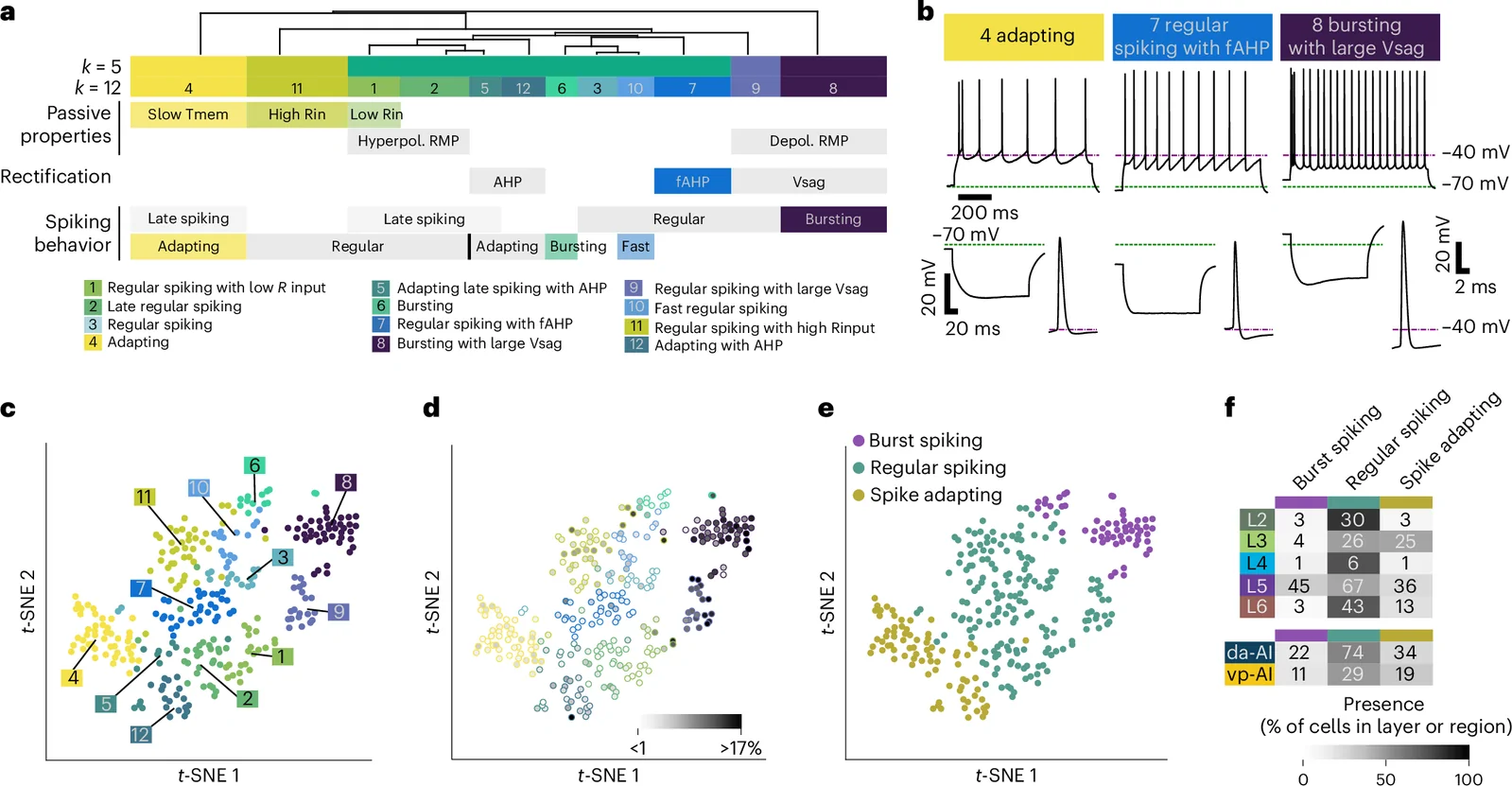
Distinct electrical profiles associate with insular layers and M-types. **a**, E-profiles and their most prominent electrical features. **b**, Representative voltage traces of three contrastive E-profiles, including action potential spiking patterns during suprathreshold stimulation (rheobase + 125 pA, top), responses to hyperpolarizing current injection (−300 pA; bottom left for each E-profile) and isolated action potentials near rheobase (bottom right for each E-profile). **c**, The *t*-SNE distribution of E-profiles. The colored boxes with numbers are E-profiles color coded for *k* = 12 in **a**. **d**, Distribution of voltage sag, a representative electrical feature contrasting E-profiles. The parameter amplitudes are shown in grayscale luminance of the filled dots, mapped on to the *t*-SNE plot in **c**. The edges of the filled dots are color coded by E-profiles. **e**, Assignment of the three E-classes—burst spiking, regular spiking and spike adapting—mapped on to the *t*-SNE plot in **c**. **f**, Contingency tables of layer (top) or AI subregion (bottom) distributions for the three E-classes. Grayscale luminance is the percentage of cells in an E-class allocated to a given layer or subregion. Numbers in gray boxes are the *n* of observed cells. Depol., depolarized; fAHP, fast afterhyperpolarization; Hyperpol., hyperpolarized; Rin, input resistance; RMP, resting membrane potential; Tmem, membrane time constant; Vsag, voltage sag.

**Extended Data Fig. 6:**
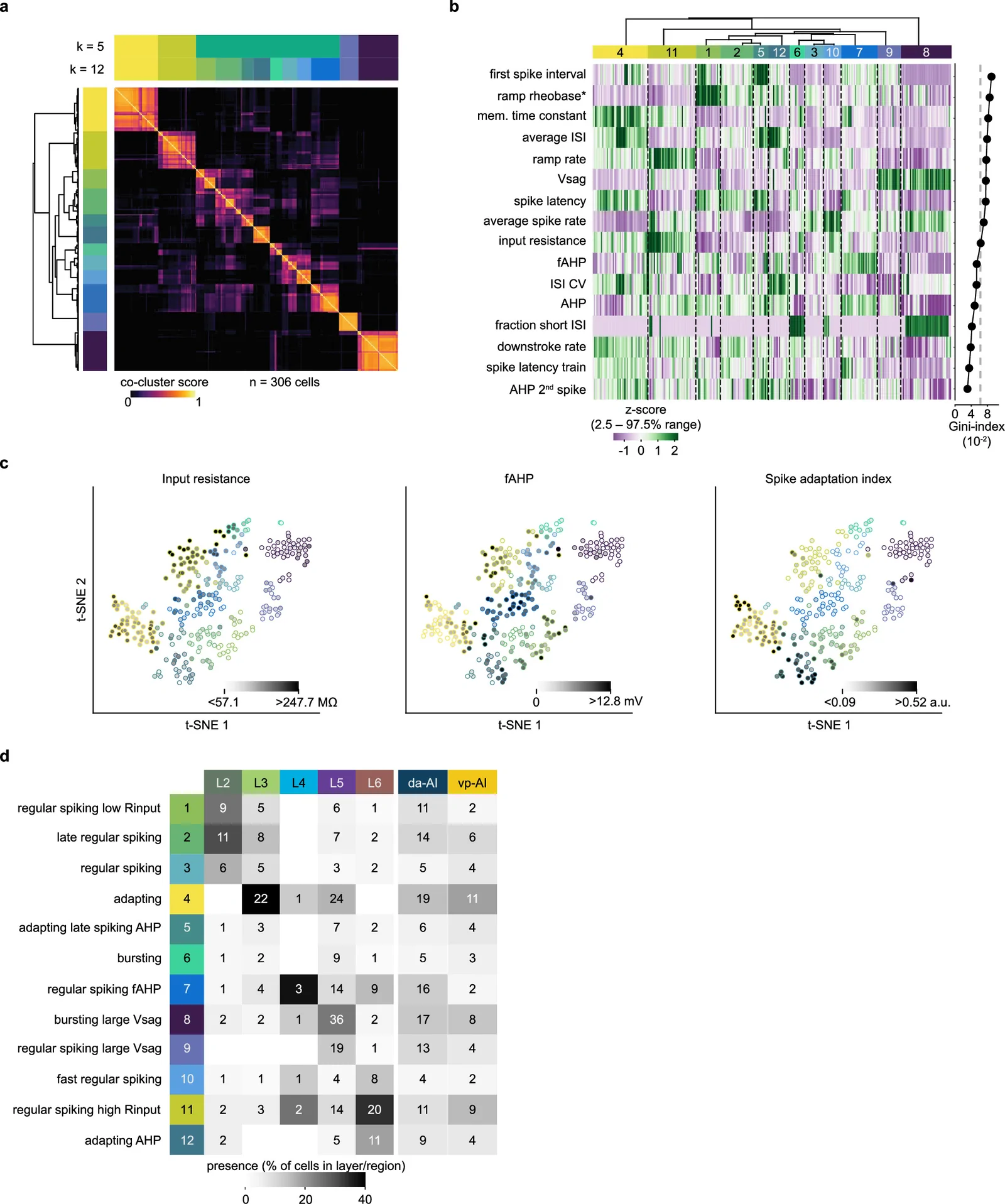
Distinct features of E-profiles. **a**, Hierarchical cluster analysis (HCA) on pair-wise co-cluster scores across 306 cells using the best performing electrical feature set. Dendrogram-based grouping is color-coded for 12 clusters (k = 12) based on optimal silhouette score, as well as for 5 clusters (k = 5). **b**, Z-scored electrical features (left), ranked by random forest classifier Gini-index (right, gray dashed line indicates equal feature contribution [1/16 features]). *: Current required to elicit the first action potential during ramping depolarizing current injection. **c**, Representative electrical features contrasting E-profiles. The parameter amplitudes are shown in grayscale luminance of the filled dots, mapped onto the t-SNE plot in Fig. 4c. Edges of the filled dots are color-coded by E-profiles. **d**, Contingency table of cell allocations to either layer (left) or subregion (right) for each E-profile. The E-profiles are named based on their prominent electrical properties. Grayscale luminance: the percentage of cells in each E-profile allocated to a given layer or subregion. Numbers in gray boxes: n of observed cells (when n > 0). Boxplots (**d**) use the following convention: center line, median; box limits, upper and lower quartiles; whiskers, 1.5x interquartile range; diamonds, outliers.

To investigate electrical cell types, we analyzed the intrinsic electrical properties of 306 pyramidal neurons in whole-cell current clamp (Fig. 4 and Extended Data Fig. 6). Similar to our M-type analysis, we conducted iCCA and identified 12 electrical profiles (E-profiles) based on 16 features describing passive, rectifying and spiking properties (Fig. 4a,b, Extended Data Fig. 6a,b, Supplementary Table 3 and Methods). Mapping E-profiles and features in a dimensionality-reduced *t*-SNE plot revealed prominent features of specific E-profiles (Fig. 4c,d and Extended Data Fig. 6c). E-profile grouping based solely on spiking patterns resulted in three main electrical classes (E-classes)—regular spiking, burst spiking and spike adapting (Fig. 4a,e)—consistent with other cortices^33,39,46^, which occupied well-separated spaces in the *t*-SNE plot (Fig. 4e). It is interesting that our burst spiking E-class (E-profiles 6 and 8) was strictly separated from other E-profiles by their fraction of short inter-spike interval and first spike interval (Extended Data Fig. 6b,d and Supplementary Fig. 10), whereas electrical property-based cell types in V1 did not reveal this strict demarcation by burst spiking features^33^. Taken together, IC pyramidal neurons comprise three E-classes, each containing E-profiles, further defined by physiological traits related to passive and rectifying properties, for example, the presence of a voltage sag (Fig. 4a, E-profiles 6 versus 8).

In contrast to M-types, the 12 individual E-profiles did not show clear layer predominance or topographic bias (Extended Data Fig. 6d). However, the relative composition of E-profiles and E-classes differed in each layer (Fig. 4f and Extended Data Fig. 6d). Notably, L2 neurons were mainly regular spiking, whereas L3 neurons displayed spike adaptation or regular spiking, but infrequently burst spiking behavior and L5 neurons displayed all three main electrical classes. Although L2 and L3 are often lumped together as one layer^33^, our results suggested an interesting segregation of L2 and L3 in the IC based on electrical properties, which is consistent with our anatomical annotation and M-types (Figs. 1g and 2a and Extended Data Fig. 3f). The differential layer presence of E-classes might contribute to layer-specific information relay in the IC.

### Insular pyramidal neurons receive noncanonical, laterally extending, local excitatory inputs

**Fig. 5:**
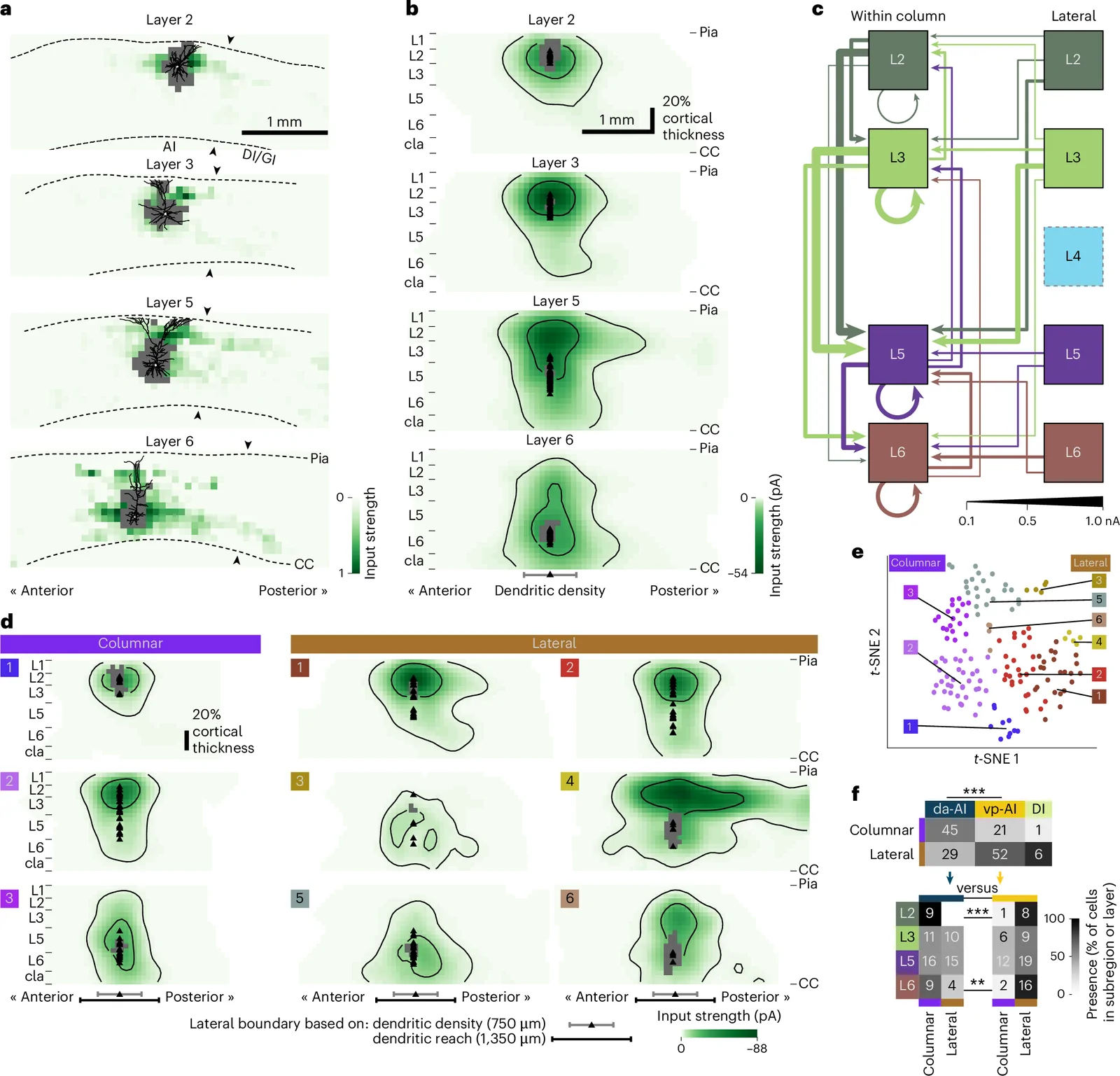
Insular pyramidal neurons receive noncanonical, laterally extending, local excitatory inputs. **a**,**b**, Example (**a**) and average (**b**) local input maps of L2 (*n* = 18 cells from 10 mice), L3 (*n* = 36 cells from 22 mice), L5 (*n* = 64 from 26 mice) and L6 (*n* = 35 cells from 20 mice) neurons aligned by pia. The example maps are shown in a linear color scale and the average maps are Gaussian filtered for visualization, shown in an exponential color scale to highlight distal inputs and contoured at the 15th and 85th percentiles of all responses (Methods). The arrowheads show the estimated boundary between AI and DI/GI (left and right of arrowheads, respectively), gray pixels the direct response sites and black triangles the soma locations. **c**, Circuit diagram of local excitatory inputs to AI layers. The arrow thickness indicates the mean input strength arriving at each layer. Only input strengths >0.1 nA are displayed. Left: within-column inputs summarizing inputs confined within the dendritic density. Right: lateral inputs summarizing distal inputs extending beyond the dendritic density (Methods). The L4 box is semitransparent because L4 is absent in the AI and is beyond the sampling range for some neurons. **d**, Average local input maps of the nine I-profiles. The format is as in **b**. **e**, The I-profile allocations revealed in the *t*-SNE-transformed local input feature space, color coded as in **d**. **f**, Contingency tables of the input classes versus subregions (top) and layers (bottom). Fisher’s exact test (two sided): **P < 0.01, ***P < 0.001, *P*_da-AI versus vp-AI_ = 1.26 × 10^−4^, *P*_L2_ = 4.11 × 10^−4^, *P*_L6_ = 1.73 × 10^−3^. Grayscale luminance shows the percentage of cells with either input class within a given subregion or layer. Numbers in gray boxes are the *n* of observed cells (when *n* > 0).

**Extended Data Fig. 7:**
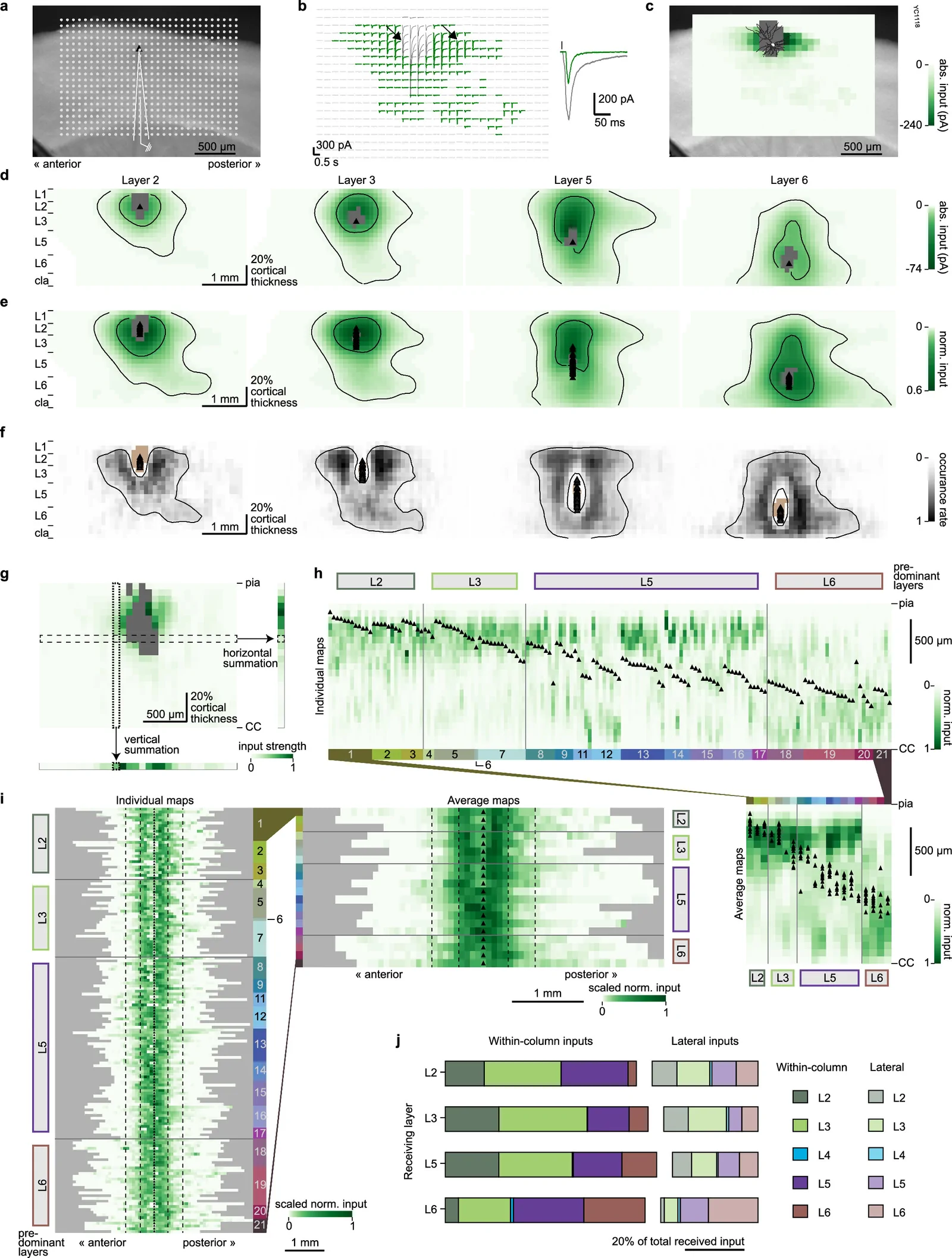
Laminar and intracolumnar analysis of LSPS maps. **a**, Example horizontal acute slice for LSPS recording. White dots: 75 µm × 75 µm stimulation grid; solid lines: the recording electrode; black triangle: soma location of the recorded cell. **b**, Trace map of current responses (left) corresponding to the stimulation sites in (**a**) and enlarged example traces of direct and synaptic responses (right, marked by arrowheads on the trace map). Sites with synaptic responses, direct responses, and no response are colored in green, dark gray, and light gray, respectively. Vertical tick: stimulation onset. **c**, Heatmap corresponding to the trace map in (**b**), overlaid with the acute slice shown in (**a**) and reconstructed dendritic morphology (black lines) of the recorded cell. Gray pixels: direct response sites. **d** and **e**, Average absolute input maps aligned by soma (**d**) and normalized input maps aligned by pia (**e**) of L2 (n = 18), L3 (n = 36), L5 (n = 64), and L6 (n = 35) neurons. Gray pixels: direct response sites; black triangles: soma locations. The average maps are Gaussian-filtered for visualization, shown in exponential color scale to highlight distal inputs, and contoured at 15^th^ and 85^th^ percentiles of all responses. **f**, Probability maps of input occurrence aligned by pia of the same neurons from (**d** and **e**). Probability maps are not Gaussian-filtered but contoured at 20% occurrence rate of their Gaussian-filtered counterparts to minimize noise. Brown pixels: direct response sites; black triangles: soma locations. **g**, 2D interpolated LSPS map of an example neuron and its vertically (bottom) and horizontally (right) collapsed 1D maps (Methods). Dotted column: example column on 2D map that sums up to the corresponding pixel (dotted pixel) on 1D map (bottom). Dashed row: example row on 2D map that sums up to the corresponding pixel (dashed pixel) on 1D map (right). All maps were normalized to their own maximum values and shown in linear color scale. **h**, Individual (top) and average (bottom right) horizontally-collapsed maps grouped by M-types (n = 154). Individual maps are sorted within groups by soma cortical depth. Heatmaps are shown in linear scale. **i**, Individual (left) and averaged (upper right) vertically-collapsed maps grouped by M-types (n = 154). Individual maps are sorted within groups by the number of non-zero pixels. Dashed lines indicate 95% dendritic density (inner) or 95% maximum dendritic reach (outer). Heatmaps are shown in exponential scale to highlight distal inputs. For (**h** and **i**), colored boxes: M-types; trapezoids: connections of the same morphology types on the outer edges of individual and averaged maps; black triangles: soma locations. **j**, Normalized input strength arriving at each AI cortical layer. Within-column (saturated colors) and lateral (semitransparent colors) inputs are separated by gaps. cla, claustrum; CC: corpus callosum.

The computational diversity of individual pyramidal neurons is also shaped by local connectivity across layers. Here we systematically investigated the excitatory local inputs to AI neurons using glutamate uncaging with laser scanning photostimulation (LSPS)^47^. Representative (Fig. 5a) and averaged heatmaps (Fig. 5b and Extended Data Fig. 7) revealed heterogeneous origins and strengths of inputs onto pyramidal neurons located in different layers. Strikingly, local inputs extended laterally along the anterior–posterior axis, sometimes originating from the DI/GI (Fig. 5a), distinct from canonical cortices^38,48,49^. We summarized the flow of information across insular layers based on the absolute inputs originating from each layer, both within-column and laterally extending (≤375 µm or >375 µm distance from the soma, respectively; Fig. 5c and Methods). We found that L2 and L3, although often grouped together as the superficial layers^31^, received differential local inputs (Fig. 5b,c and Extended Data Fig. 7d). Specifically, L3 received stronger inputs than L2, especially from L2, L3 and L6. These results indicated distinct roles for L2 and L3 in local AI processing, consistent with divergent L2 and L3 microcircuits in some primary cortices^48,50,51^. The deep layers, L5 and L6, received marked inputs from the claustrum and at locations extending far away from the soma. In particular, L5 neurons received the strongest and most distal inputs from superficial layers, often posteriorly biased and up to 1.5–2 mm away from the soma (Fig. 5a–c and Extended Data Fig. 7d,e). L6 received the strongest laterally extending inputs from deep layers in both anterior and posterior directions, whereas its superficial inputs originated mostly within column (Fig. 5b and Extended Data Fig. 7d,e,j). Such lateralized local inputs to L5 and L6 are distinct from canonical microcircuits in other cortices^38,48,49^.

Intralaminar connectivity, although underestimated due to the exclusion of synaptic responses inseparable from direct responses, was evident within all layers and strongest in L3, followed by L5 and L6 (Fig. 5c). In addition, we identified reciprocal inter-laminar connections, characterized by strong descending inputs (for example, L2 → L5, L3 → L5) and relatively weak ascending inputs (for example, L5 → L2, L5 → L3). Although strong descending inputs were conserved among the AI, agranular motor cortex and canonical granular cortices^48,52^, the strong ascending inputs to L2 and/or L3 observed in granular cortices^48,51^ were not observed in the agranular motor cortex^48,52^ or AI, supporting the observation that local circuits of agranular and granular cortices are distinct.

**Extended Data Fig. 8:**
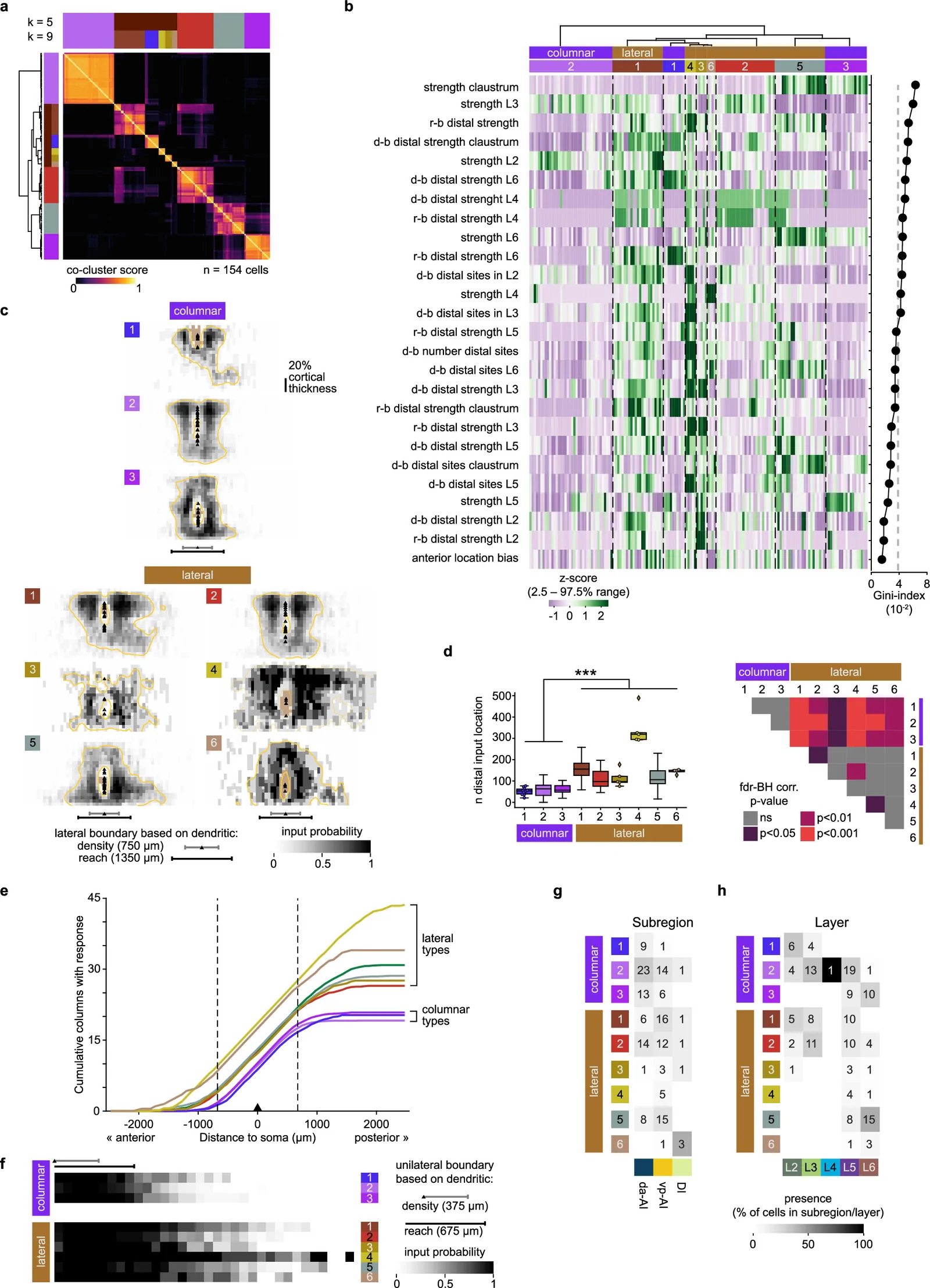
Local input features highlight lateral extent of input profiles. **a**, Hierarchical cluster analysis (HCA) on pair-wise co-cluster scores across 154 cells using the best performing input feature set. Dendrogram-based grouping is color-coded for 9 clusters (k = 9) based on optimal silhouette score, as well as for 5 clusters (k = 5). **b**, Z-scored input features (left), ranked by random forest classifier Gini-index (right, gray dashed line indicates equal feature contribution [1/26 features]). Input profile color-coding is consistent throughout this figure and Fig. 5. For lateral input features, the lateral border was defined by boundaries based on either 95% dendritic density (density-based, d-b) or 95% maximum dendritic reach (reach-based, r-b). **c**, Probability maps of input occurrence aligned by pia for all I-profiles. Maps are interpolated vertically and normalized to the cortical thickness. Probability maps are not Gaussian-filtered but contoured at 20% occurrence rate of their Gaussian-filtered counterparts to minimize noise. Brown pixels: direct response sites; black triangles: soma locations; gray and black lateral whisker bars: lateral boundary thresholds used during feature extraction, based on dendritic density (750 µm) or reach (1350 µm), respectively. **d**, Number of distal input locations beyond the dendritic density-based borders (left), and the corresponding heatmap of p-values from pair-wise comparisons between I-profiles (right, Kruskal-Wallis H (8) = 80.77, p = 3.4·10^−14^, with post hoc Dunn’s two-sided comparisons with false-discovery rate Benjamini-Hochberg correction, fdr-BH corr.). Mann-Whitney U two-sided tests were used to further compare columnar and lateral input classes; U = 675.5, ***: p = 3.4·10^−16^. Number of observations (n) and exact p-values for all groups provided in Supplementary Table 15; individual points are shown for groups with n ≤ 10 (dots or diamonds). **e**, Mean cumulative number of responsive columns in a 2D input map (each recording column is 75 µm in width) for each I-profile, displayed from anterior (left) to posterior (right). Dashed lines: boundaries of the dendritic reach. **f**, Input probability as a function of distance from soma. **g** and **h**, Contingency tables of I-profiles and -classes versus subregion (**g**) and layer (**h**). Grayscale luminance: the percentage of cells in each I-profile allocated to a given aforementioned modality. Numbers in gray boxes: n of observed cells (when n > 0). Boxplots (**d**) use the following convention: center line, median; box limits, upper and lower quartiles; whiskers, 1.5x interquartile range; diamonds, outliers.

Beyond layer-based grouping^48,49^, we identified nine prototypical input profiles (I-profiles) using our iCCA approach (Fig. 5d–f, Extended Data Fig. 8 and Methods). Six out of nine I-profiles showed pronounced lateralized inputs, with more lateral inputs located beyond the dendritic density border (375 µm from the soma, Kruskal–Wallis *H*(8) = 80.77, *P* < 0.001) and sustained input probabilities located beyond the maximal dendritic reach (675 µm from the soma; Extended Data Fig. 8d–f). Moreover, these six I-profiles were well separated in the *t*-SNE plot from I-profiles with primarily columnar inputs (Fig. 5e), confirming the distinction between the columnar and lateral input classes. Notably, similar to the subregional preferences of morphology-defined cell types, neurons located in the da-AI or vp-AI received predominantly columnar or lateral inputs, respectively (Fisher’s exact *P* < 0.001; Fig. 5f and Extended Data Fig. 8g). Such predominance was observed for only L2 and L6, but not L3 and L5, neurons, likely reflecting the functional divergence of L2 and L6 neurons in da-AI and vp-AI, rather than a potential failure to preserve lateral inputs in brain slices. These results indicate that columnar or lateral integration preferentially occurs depending on the anatomical location of neurons.

### Multimodal analyses distinguish specific insular cell types

**Fig. 6:**
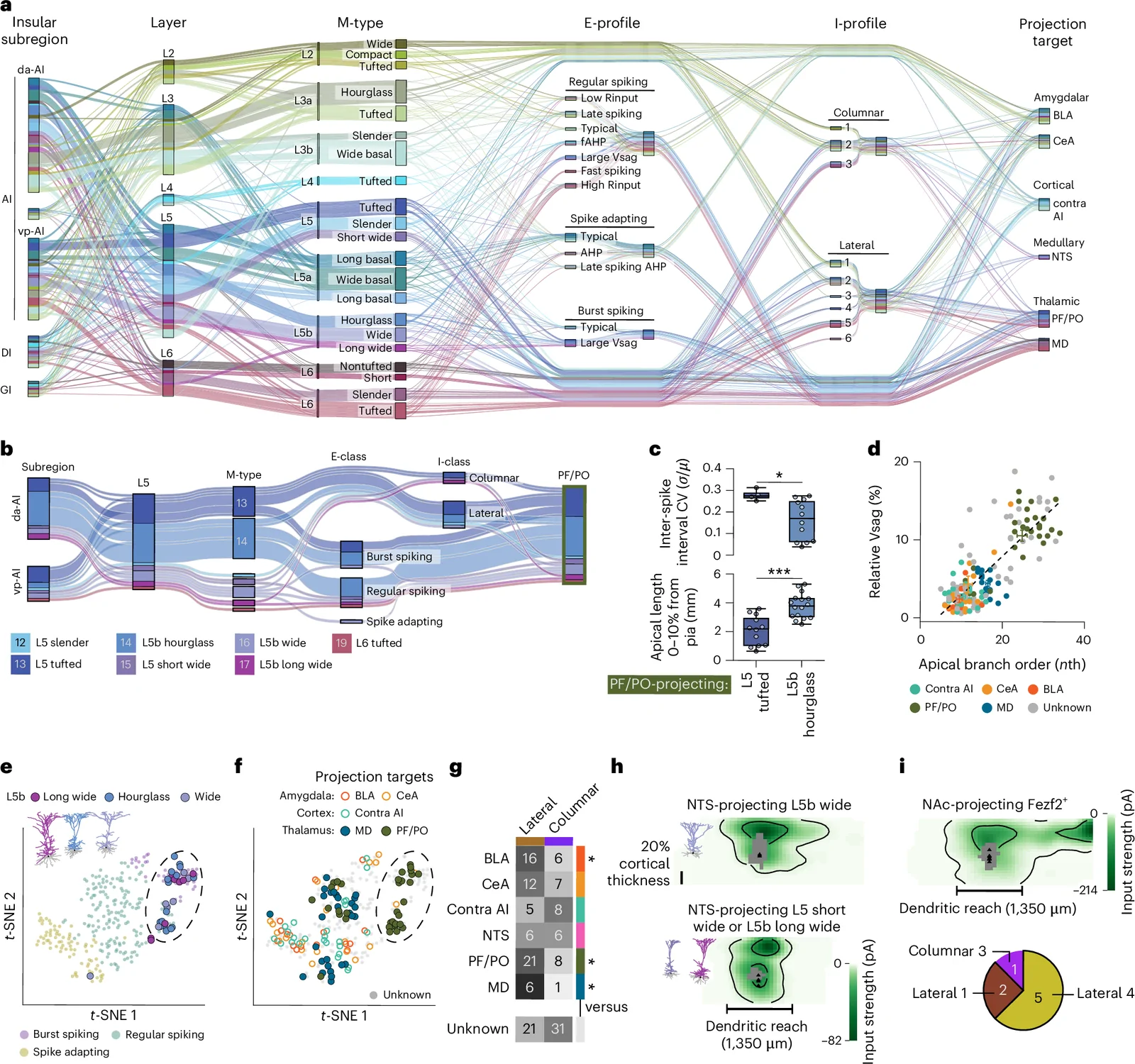
Multimodal analyses distinguish specific insular cell types. **a**, Parallel categories diagram of all neurons and their allocations to subregions, layers, M-classes (based on predominant layer and cluster hierarchy from Fig. 2a), M-types, E-profiles, I-profiles and projection targets. **b**, Parallel categories diagram of L5 PF/PO-projecting neurons and their allocations to subregions, M-types, E-classes and I-classes. **c**, Quantification of electrical (top, inter-spike interval coefficient of variation, two-sided Mann–Whitney *U*-test, *U* = 44, *P* = 0.013) and morphological (bottom, apical length within 10% cortical thickness bin from pia, two-sided Mann–Whitney *U*-test, *U* = 20, *P* = 4.5 × 10^−4^) characteristic features for PF/PO-projecting L5 tufted (M-type 13) and L5b hourglass (M-type 14) neurons. The circles show individual observations, boxplot center lines the median, box limits the upper and lower quartiles, whiskers 1.5× the IQR and diamonds the outliers. The numbers of observations for all groups are provided in Supplementary Table 10. **d**, Relative voltage sag (Vsag) as a function of apical branch order (Spearman’s correlation = 0.70), with projection target labeled. The dashed line shows linear fit. **e**,**f**, M-type (**e**) or projection target (**f**) identities mapped onto the *t*-SNE plot from Fig. 4c. Dashed ellipses highlight a group of L5b neurons with similar electrical properties that primarily project to the PF/PO. **g**, Contingency table of the input classes versus projection targets (two-sided Fisher’s exact test: *P < 0.05, ***P < 0.001, *P*_PF/PO_ = 0.010, *P*_MD_ = 0.040, *P*_BLA_ = 0.021). The numbers in gray boxes are the *n* of observed cells. **h**,**i**, Three examples of averaged NTS-projecting (**h**) or NAc-projecting (**i**) local input maps with either prominent columnar or lateral connectivity based on specific cell types defined by other modalities (morphology, projection target, transgenic expression). The average map format is as in Fig. 5b,d. Pie chart (**i**, bottom) shows distribution of I-profiles for NAc-projecting Fezf2^+^ neurons as assigned by the random forest classifier (Supplementary Table 2; ‘I-features × I-profiles’; Supplementary Figs. 7a and 8a; *n* = 8 cells; numbers in pie chart are the *n* of observed cells). Fezf2^+^, *Fezf2-CreER-*positive neurons; CV, coefficient of variaiton; proj., projecting.

**Extended Data Fig. 9:**
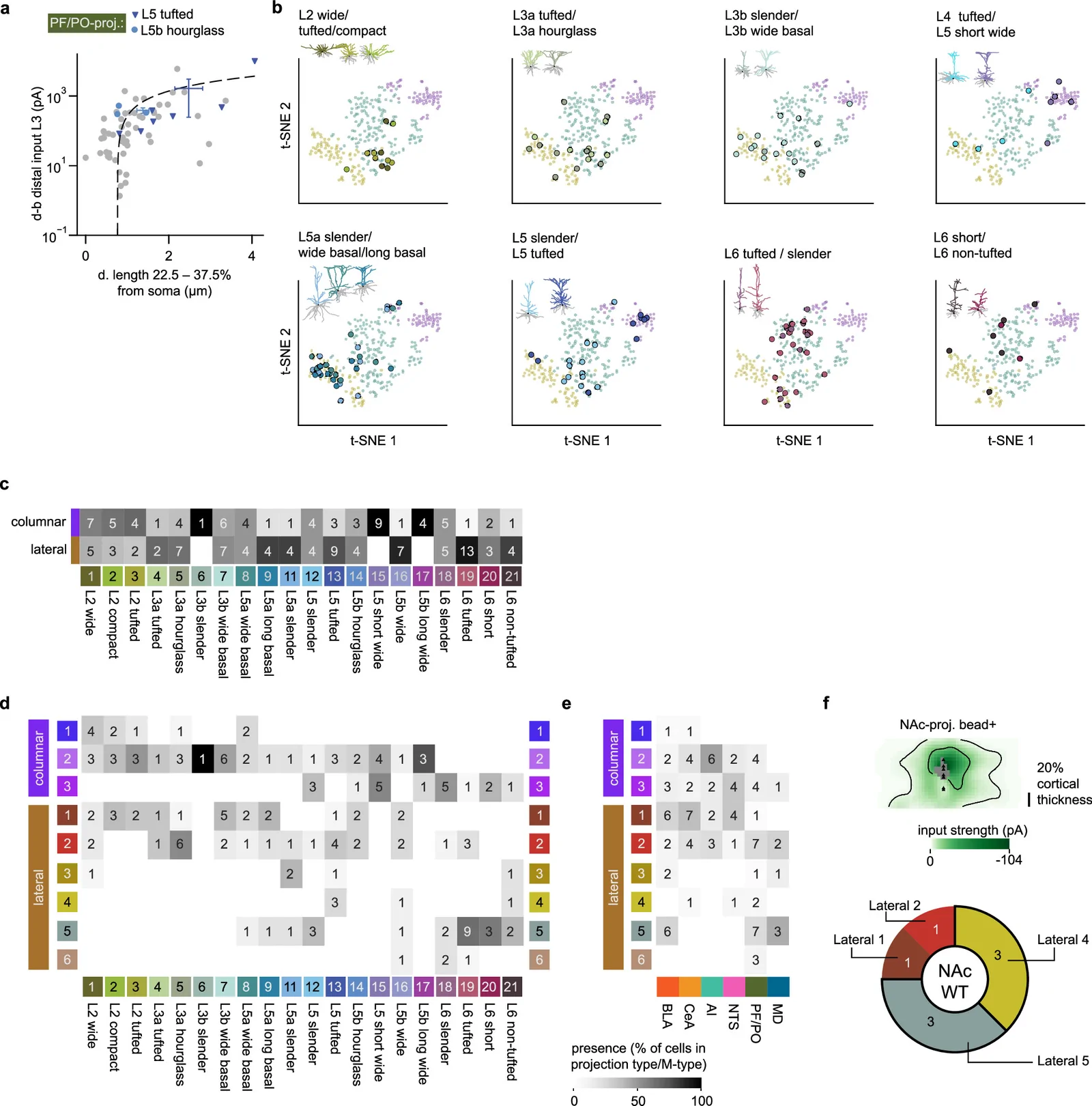
Electrical and input characteristics are related to specific morphologies and projection targets. **a**, For all L5 neurons (n = 63), including PF/PO-projecting *L5 tufted* (M-type 13, n = 7 cells) and *L5b hourglass* (M-type 14 n = 3 cells) neurons, distal L3 inputs are monotonically correlated with dendritic length, normalized to cortical thickness, within a 22.5 – 37.5% bin from soma towards pia (Spearman correlation = 0.52, semi-log scale). L3 distal input is defined as the summed L3 inputs beyond the dendritic density-based (d-b) boundaries. A linear fit (dashed line, 943.85 x – 704.55; r^2^ = 0.28) was applied. Color-coded whiskers: mean and standard error of plotted features for PF/PO-projecting *L5 tufted* (M-type 13) and *L5b hourglass* (M-type 14) neurons. **b**, M-types mapped onto the tSNE plot in Fig. 4e. **c**, Contingency table of the input classes versus M-types. **d** and **e**, Contingency tables of the input profiles versus M-types (**d**) and projection targets (**e**). For (**c**–**e**), grayscale luminance: the percentage of cells with either input profile/class within a given M-type or projection target type. Numbers in gray boxes: n of observed cells (when n > 0). **f**, Averaged input map (top) and I-profile distribution (bottom) of NAc-projecting neurons labeled with retrograde beads (n = 8 cells, numbers in pie chart: n of observed cells). I-profiles were assigned by an I-profile classifier (Supplementary Table 2; “I-features x I-profiles”, Supplementary Figs. 7a and 8a; Methods). The top two most frequent I-profiles are highlighted in the pie chart.

To comprehensively associate multiple modalities used to define a cell type, we generated a visualization of all individual IC pyramidal cells across modalities and properties in our dataset (Fig. 6a and Methods), where cell types and modalities could be queried (MouseInsularAtlas: https://gitlab.com/maolab/mouseinsularatlas). Based on an exploration of this visualization along with the underlying features that drive cell-type specification (Extended Data Figs. 4a, 6b and 8b), we tested the ability to differentiate cell types by specific features and the relationship between cell-type modalities (Fig. 6 and Extended Data Fig. 9).

#### Specific insular cell types are separated by individually salient features

One advantage of our iCCA approach is that it is based on interpretable, biologically relevant features that quantify neuronal properties underlying a cell type. The availability of these quantitative features allowed us to address two fundamental questions: what properties distinguish a cell type and how do structural properties (for example, morphology) and functional properties (for example, electrical properties) correlate? First, we compared properties between cell types and identified salient features that distinguished them (Supplementary Fig. 11 and Methods). For example, although both L5 tufted and L5b hourglass PF/PO-projecting neurons received strong lateral L3 inputs (Extended Data Fig. 9a), these two cell types were distinguishable by spiking properties and apical tuft lengths (Fig. 6b,c). Similarly, L3b–L5a amygdala-projecting cell types in different input classes were distinguishable by apical tuft lengths (Supplementary Fig. 12). Second, we found multiple pairs of crossmodal features with significant monotonic association (Fig. 6d, Supplementary Fig. 13 and Methods). For example, the relative size of voltage sag correlated with maximal apical branch order (Fig. 6d and Supplementary Data Fig. 13a), consistent with reported hyperpolarization-activated cyclic nucleotide-gated (HCN) channel gradients along apical dendrites of cortical neurons^53^. This correlation also revealed a clear separation of PF/PO-projecting neurons from other projection neurons by their large complex dendritic arbors and strong voltage sags (Fig. 6d, and see also Figs. 3c, 4d and 6e,f and Extended Data Fig. 5b). Beyond this example, morphological features also correlated with electrical and input features within all layers except L2 (Supplementary Fig. 13), indicating that the size and complexity of dendritic morphology may influence cellular physiology and local inputs.

#### Morphology and projection targets associate with specific electrical and input profiles

We next explored crossmodal associations with electrical properties. Mapping M-type identities onto the *t*-SNE plot of electrical features indicated that several M-types, located within the same layer, shared electrical properties (Fig. 6e and Extended Data Fig. 9b). For example, most L5b neurons, M-types 14, 16 and 17, displayed two modes of spiking behaviors, bursting and regular spiking (Figs. 4e and 6e), reminiscent of deep L5-ET neurons found in sensory cortices^46,54^. Mapping projection targets onto the *t*-SNE plot of electrical features showed that IT neurons (for example, cortical-projecting neurons) and ET neurons (for example, thalamic-projecting neurons) had distinct electrical properties (Fig. 6f), supporting this notion observed in the primary cortices^46,54^. Although corticothalamic MD-projecting and PF/PO-projecting neurons were located in overlapping cortical layers (Fig. 3d,e), we found that only PF/PO-projecting neurons resembled the aforementioned L5b neurons defined by their morphology and physiology, which might contribute to the functional differences of these two thalamic nuclei (Fig. 6e,f and Supplementary Fig. 10).

Several morphological types and projection targets also showed prominent associations with specific local input patterns (Fig. 6g–i and Extended Data Fig. 9c–f). First, L5 short wide and L5b long wide neurons received exclusively columnar inputs (I-profiles 2 and 3), whereas L6 tufted neurons associated overwhelmingly with lateral inputs (I-profile 5) (Extended Data Fig. 9c,d). Second, IC neurons projecting to the PF/PO and MD thalamus, as well as the BLA, but not the CeA, contra AI or NTS, displayed a significantly higher frequency of lateral inputs, compared to null (‘unknown’) neurons (Fig. 6g). Guided by our visualization (Fig. 6a), we found that remarkable preferences for columnar or lateral input patterns could also be deduced from combinations of the morphology with the projection target. Notably, NTS-projecting neurons could be divided into two groups for their local inputs, depending on their morphology (Fig. 6h). As NTS-projecting neurons express the *Fezf2* gene^13^, we examined another set of Fezf2^+^ neurons with projections to the NAc and found that they predominantly exhibited lateral I-profiles, suggesting that projection targets can further specify input patterns among neurons expressing the same molecular marker (Fig. 6i and Extended Data Fig. 9f). These results indicate that pyramidal neurons defined by morphology and projection target may specifically receive columnar or lateral inputs. Our visualization tool (Fig. 6a), combined with the identified salient and crossmodal-correlated features, offers an intuitive interface for connecting the cellular properties of insular neurons to their functions.

### Multimodal features predict specific insular cell types

We quantified the contribution of different modalities on determining a cell type (Supplementary Figs. 14 and 15) based on two iCCAs combining morphological features with either electrical or input features (Methods). Morphology features were found to be the most informative in determining cell types, evidenced by their top Gini index rankings during classifier training (Supplementary Figs. 14b and 15b). In contrast to V1^33^, using morpho-electrical or morpho-input feature sets resulted in heterogeneous neuronal groups, meaning that each group often comprised multiple M-types, E-profiles or I-profiles (Supplementary Figs. 14g,h and 15h,i).

**Fig. 7:**
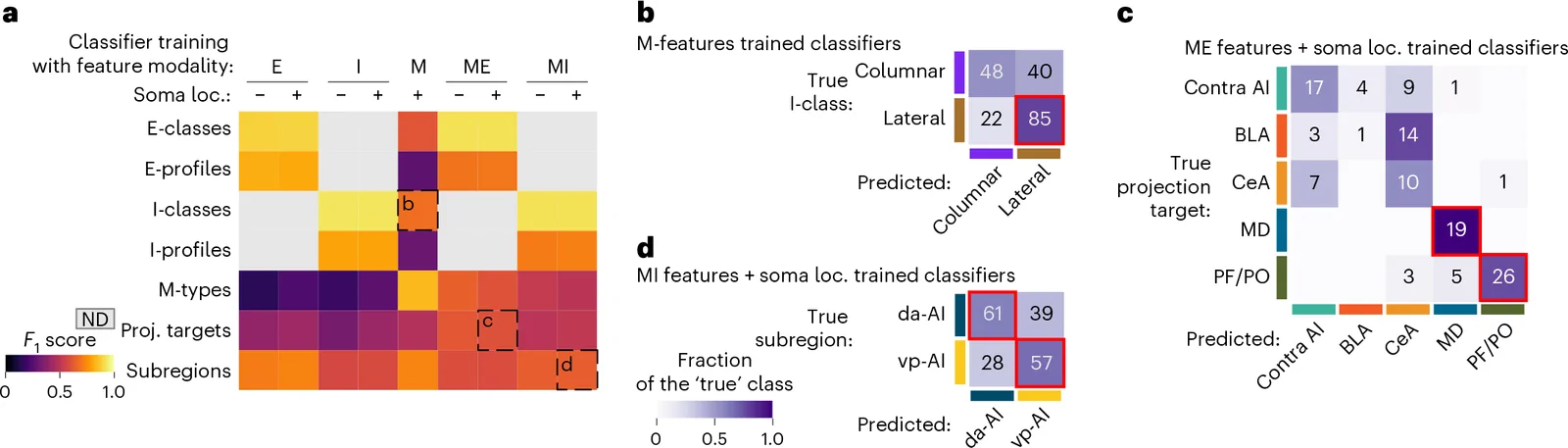
Multimodal features predict specific insular cell types. Performance of random forest classifiers trained on specific unimodal and multimodal feature sets to predict classes and types across different modalities. **a**, Classifier accuracy (*F*_1_ score; Methods). The dashed boxes show *F*_1_ scores for classifier predictions from **b**–**d**. **b**, The I-classes predicted by M-features. **c**, Projection targets predicted by morpho-electrical (ME) features and soma location. **d**, Subregions predicted by morpho-input (MI) features and soma location. For **b**–**d**, purple luminance shows the percentage of neurons correctly predicted by classifiers for a given I-class, projection target or subregion and red boxes the cell types well predicted by classifiers. loc., location; ND, no data; proj., projection.

To explore the utility of our multimodal dataset for future cell-typing efforts, we trained random forest classifiers with our iCCA features to quantitatively assess whether they accurately assign neurons to their known types (Fig. 7a–d, Supplementary Figs. 7a and 8a and Methods). We identified crossmodal classifiers that could predict a subset of cell types (Fig. 7a), although not all crossmodality-trained classifiers assigned cell types accurately (Supplementary Fig. 7a). Prominently, classifiers trained on the feature sets that included morphology alone accurately predicted whether a cell received lateral inputs (Fig. 7b). When electrical or input features were combined with morphology, trained classifiers accurately predicted thalamic-projecting neurons (Fig. 7c) or subregion location (da-AI versus vp-AI; Fig. 7d), respectively. These multimodal classifiers, along with high-performing within-modality classifiers (Fig. 7a), serve as valuable platforms for future work to predict cell-type identity when certain modalities are unavailable.

### Posterior–anterior local inputs bridge a thalamocortico-amygdalar long-range circuit

**Extended Data Fig. 10:**
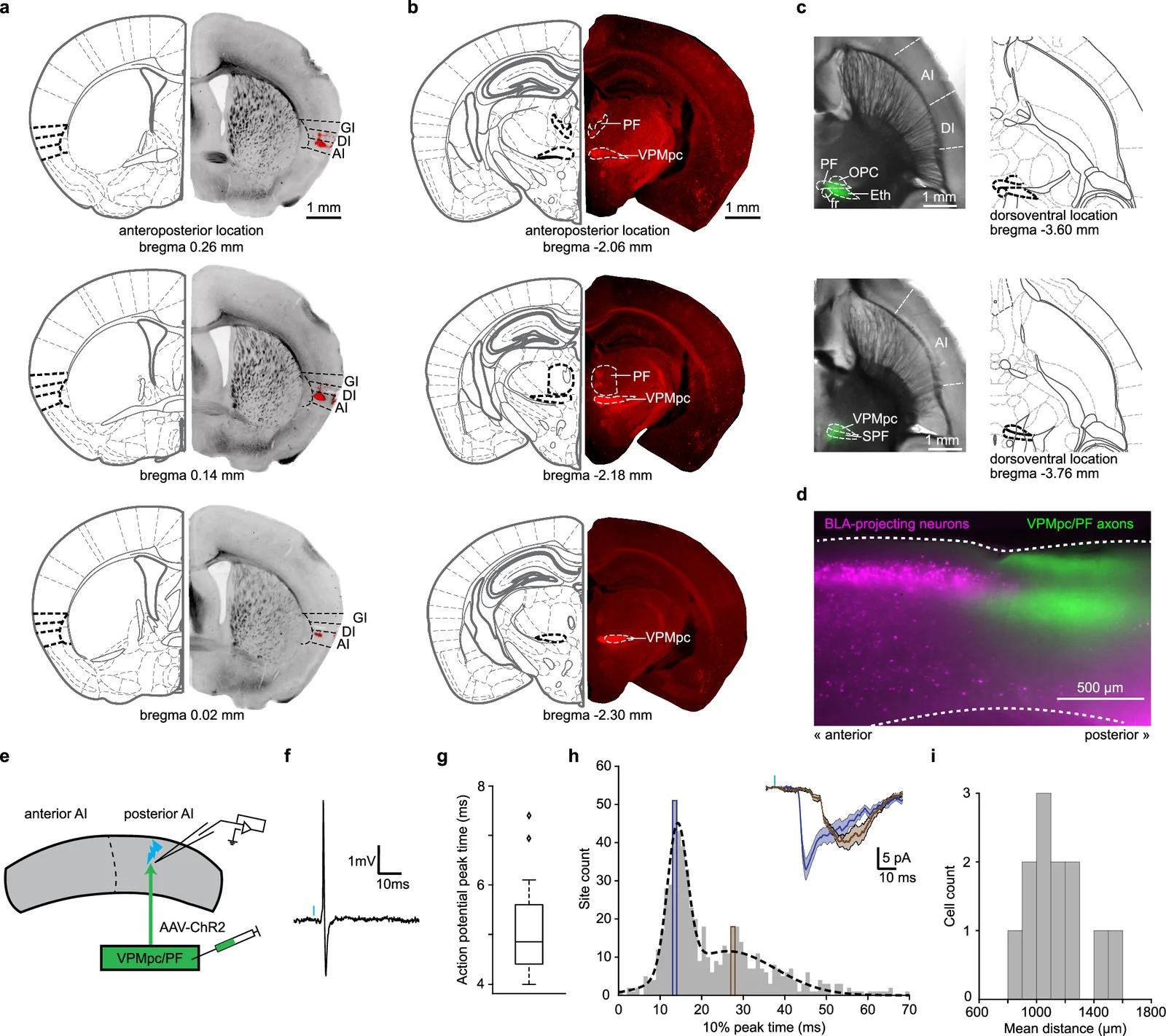
Anatomical analysis of the VPMpc/PF-AI circuit and kinetics of the synaptic responses. **a**, Example AI injection site of retrograde beads. Brightfield images (right) of all coronal sections (thickness: 50 µm) containing the injection site (red fluorescence) are shown and matched to equivalent sections from ref. ^44^ (left). **b**, Example epifluorescent images of bead locations in VPMpc and PF (right) and matched sections from the Franklin and Paxinos atlas (left), from the same animal shown in (**a**). Bead location images were linearly adjusted for brightness and contrast. **c**, Brightfield examples of acute horizontal brain slices (thickness: 350 µm) containing the thalamic ChR2 injection (left, green fluorescence) and matched sections from the Franklin and Paxinos atlas (right). All slices containing the injection are shown. For (**a**–**c**), positions relative to bregma are noted according to the best matched atlas sections. **d**, Overlay of retrogradely-labeled BLA-projecting neurons (magenta) and ChR2-positive VPMpc/PF axons (green). **e**, Schematic of ChR2 stimulation and loose-patch recording in the posterior AI. Blue flash: optogenetic stimulation. **f**, Example loose-patch trace acquired in an experiment illustrated in (**e**). Blue tick: stimulation onset. **g**, Time delay from stimulation onset to action potential peak (n = 14 responsive, out of 29 total recorded cells). **h**, Histogram of time delay from posterior stimulation onset to anterior response when it reaches 10% peak response and average response trace (inset) at the 14 – 15 ms (blue box) or 27 – 28 ms (brown box) bin. Blue tick: stimulation onset. Shaded area: standard error. Dotted line: bimodal Gaussian fit of the distribution. **i**, Histogram of mean anterior-to-posterior distance between recorded soma and response-yielding posterior stimulation sites (Fig. 8b). The closest distances from the soma to the stimulation columns containing each response-yielding site were averaged and plotted. PF, Parafascicular thalamus; OPC, Oval paracentral thalamus; Eth, Ethmoid thalamus; fr, fasciculus retroflexus; VPMpc, Ventroposteromedial thalamus, parvicellular part; SPF, Subparafascicular thalamus. Boxplot (**g**) uses the following convention: center line, median; box limits, upper and lower quartiles; whiskers, 1.5x interquartile range; diamonds, outliers. Panels **a**,**b** (left) and **c** (right) adapted with permission from ref. ^44^, Elsevier.

To demonstrate the utility of our multimodal dataset for functional studies, we investigated the behavioral relevance of a prominent feature of insular local circuits, the laterally extending input profiles (Fig. 5b,d). We hypothesized that this local circuit is in place to connect anatomically separated but functionally related long-range inputs and outputs. Anatomically, thalamic nuclei—the parvicellular ventroposteromedial thalamus (VPMpc) and the nearby PF—preferentially innervated the posterior IC, whereas insular neurons projecting to the anterior BLA resided in the anterior IC (Extended Data Fig. 10a–d)^1,3,55^. Behaviorally, both VPMpc and BLA are critically involved in conditioned taste aversion (CTA)^56^. As our earlier results suggested that BLA-projecting neurons preferentially receive the lateralized local inputs (Figs. 5b,d and 6g), we hypothesized that these inputs could be the missing link between the thalamic nuclei that relay gustatory and interoceptive information and the amygdala, which mediates valence and behavioral choices based on such sensory information.

**Fig. 8:**
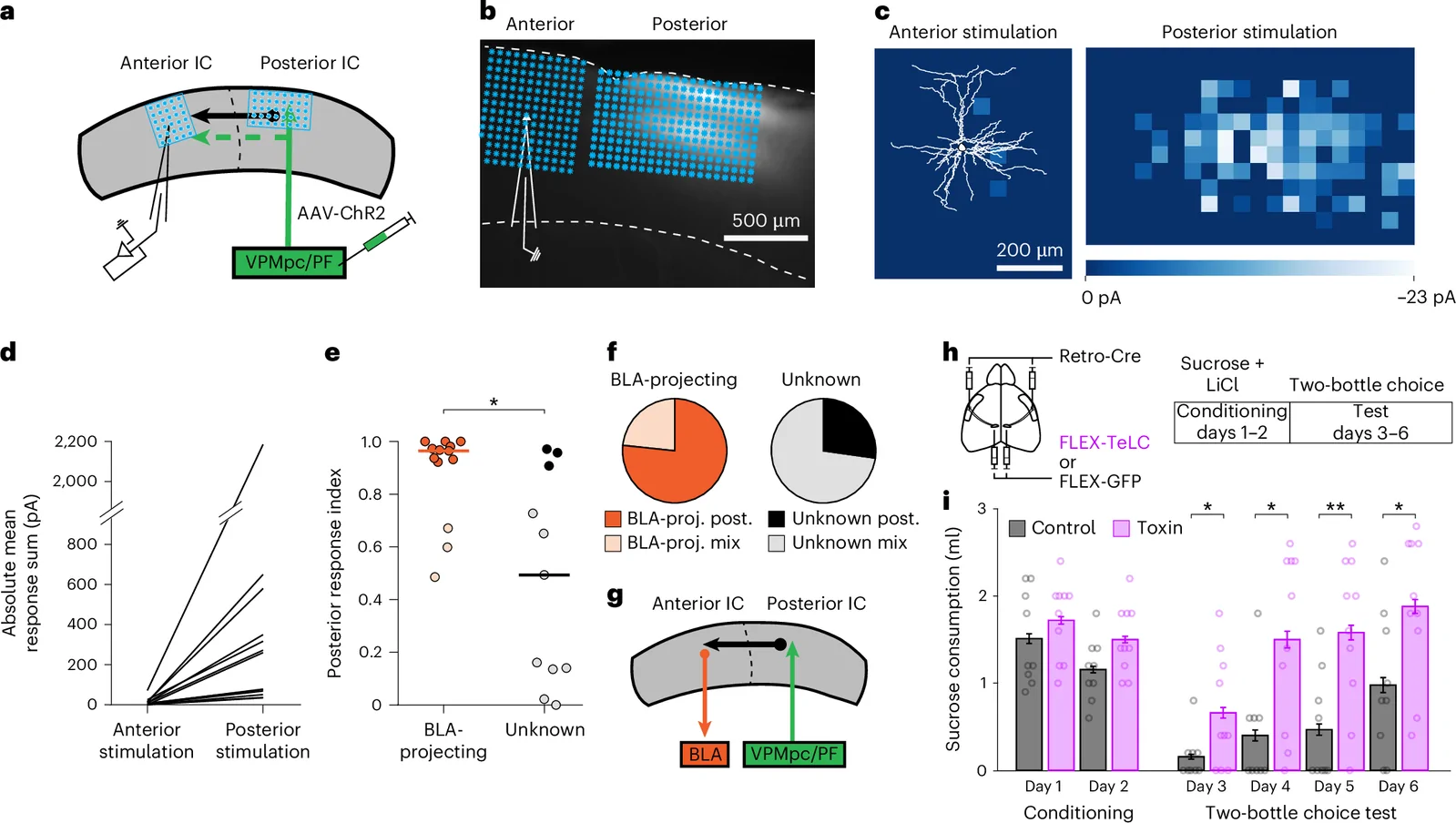
Posterior–anterior local inputs bridge a thalamocortico-amygdalar long-range circuit. **a**,**b**, Schematic (**a**) and example horizontal acute slice (**b**) of a recording in the anterior IC in response to ChR2 stimulations of VPMpc/PF axons in either the anterior (left) or the posterior (right) IC. Blue dots are a 50 µm × 50 µm grid of stimulation locations. **c**, Example ChR2-assisted circuit mapping (CRACM) maps corresponding to anterior (left) and posterior (right) stimulations shown in **b**. Reconstructed dendritic morphology (left) of the recorded neuron is overlaid. **d**, Quantification of CRACM maps in response to anterior versus posterior stimulations. The sum of the responses from neurons responding strongly to posterior stimulation and minimally to anterior stimulation are shown (*n* = 13; anterior response <10% of posterior response; Methods). **e**, Posterior response index of BLA-projecting neurons versus neurons with unknown projection targets (*P* = 0.0206, one-sided Fisher’s exact test). The horizontal bars show the median, the dark circles the neurons with posterior response index >0.9 and the light circles the posterior response index ≤0.9 (*n* = 13 for BLA-projecting neurons; *n* = 11 for neurons of unknown projection targets). *P < 0.05. **f**, Distribution of response types shown in **e**. **g**, Schematic of the VPMpc/PF–IC–BLA long-range circuit. **h**, Injection schematic (left) and behavioral paradigm (right) for CTA (Methods). **i**, Sucrose consumption level across days (control: *n* = 9; toxin injected: *n* = 10, days 3–6; *P* = 0.0435, 0.0111, 0.0058 and 0.0303, respectively, two-sided, unpaired Student’s *t*-test, without corrections for multiple comparisons). The bar plots show the mean consumption, the open dots the individual consumption and the error bars the s.e. *P < 0.05, **P < 0.01. post., posterior; proj., projecting; stim., stimulation; TeLC, tetanus toxin light chain.

To test the synaptic connection of this circuit, we performed loose-patch recordings of posterior IC pyramidal neurons while photostimulating VPMpc and PF (VPMpc/PF) axons expressing channelrhodopsin (ChR2). VPMpc/PF projections drove action potentials in the posterior IC (Extended Data Fig. 10e,f; *n* = 14 out of 29 neurons)^57^. To test whether VPMpc/PF signals route via posterior–anterior local inputs, we recorded neurons in the anterior IC while performing ChR2-assisted circuit mapping (Fig. 8a,b)^58^. Most neurons in the anterior IC responded strongly to optogenetic stimulations in the posterior IC and minimally to stimulations in the anterior IC (Fig. 8c,d; anterior response <10% of posterior response, *n* = 13 out of 24 neurons). This result indicated that VPMpc/PF input required posterior–anterior local connections to reach the anterior IC. The poly-synaptic nature of this thalamoanterior IC circuit was supported by the delayed onset of anterior IC responses to VPMpc/PF stimulation (Extended Data Fig. 10g,h). Also, the distance from the VPMpc/PF axon stimulation sites to the responding anterior IC neurons was within the range of observed posterior–anterior local input distances (Fig. 5b and Extended Data Fig. 10i). Thus, the lateralized local inputs within the IC conveyed VPMpc/PF signals from the posterior to the anterior IC. Furthermore, we demonstrated that IC neurons labeled by retrograde injections in the anterior BLA responded preferentially to posterior photostimulation compared to nearby bead-negative neurons (Fig. 8e,f), indicating that thalamic signals were integrated and transmitted to the BLA via posterior–anterior connections within the IC (Fig. 8g).

Finally, to probe the in vivo function of the VPMpc–IC–BLA pathway, we investigated the role of its key component, the VPMpc–posterior IC projection, in CTA. Using tetanus toxins to specifically inhibit the VPMpc–posterior IC synapse^59^, we evaluated sucrose drinking paired with malaise-inducing LiCl (Fig. 8h and Methods). Tetanus-injected animals displayed faster extinction of CTA compared to GFP-injected controls (Fig. 8i), suggesting that the VPMpc–posterior IC projection contributes critically to the retention of CTA. Taken together, we demonstrate that long-range, VPMpc–posterior IC inputs are essential to CTA and that lateral, posteriorly biased local inputs within the IC connect these thalamic inputs to the BLA.

## Discussion

Our multimodal dataset, comprising 1,130 pyramidal neurons, provides a comprehensive view of the cellular and circuit organization of the insula and establishes the foundation for future behavioral studies. Cell-typing analyses revealed several remarkable aspects of insular neurons, including large and elaborate dendrites, the anterior–posterior topographic distributions of M-types, layer-specific spike pattern distributions and lateralized local inputs, along with associations of these properties with specific projection targets. In general alignment with the empirically determined IC subregions (Supplementary Notes), quantitative anatomical analyses revealed that the da-AI and vp-AI subregions can be defined by the topographical distributions of M-types, thereby allowing users to identify subregions enriched for specific cell types. We further demonstrated that laterally extending intra-insular microcircuits connect the functionally distinct anterior and posterior subregions and mediate long-range thalamus–amygdala connectivity. Future work could leverage our anatomical framework and trained classifiers to both map and categorize cells obtained from imaging or physiology experiments to gain insights into circuit functions.

### Insular cell types and crossmodal associations

Von Economo neurons, considered a hallmark neuronal type in the insula of higher-order species^36^, were not observed in our morphology dataset, despite saturating sampling depth (Extended Data Fig. 3e). However, we found a subset of L5a wide basal neurons with two apical dendrites—a potential analog to the ‘fork cells’ found in human ICs^37^. Surprisingly, several mouse insular M-types exhibited elaborate dendritic structures resembling those of primate cortical neurons, suggesting that these complex morphologies may support multimodal signal processing in the IC.

Crossmodal analyses of cell types revealed broad heterogeneity in categorical associations throughout our dataset, such as one M-type associating with multiple E-profiles, while also highlighting specific cell types from different modalities that may capture largely overlapping sets of neurons (Fig. 6, Supplementary Figs. 14 and 15 and Supplementary Notes). The former prompts the hypothesis that modalities, such as electrical and input properties, may not be fixed for every cell type defined by morphology, but rather could change based on neuromodulators (for example, dopamine, acetylcholine) that shift neurons to particular ‘cell states’ (Supplementary Notes)^60^.

The growing availability of transcriptomic datasets^61,62^, which often now include morphological information^63^, provides platforms for linking all modalities presented here to transcriptional information through morphology. Practical challenges remain in the stratification of metadata and cell-typing strategies (for example, feature extraction and selection) across datasets^64^. Therefore, our atlas-registered dataset with a reproducible clustering pipeline will facilitate future work reconciling existing whole-brain transcriptomic types^61^ with our multimodal insular cell types.

### Characteristics of local insular computation

Distinct yet continuous AI, DI and GI subregions piqued our interest regarding information flow within and between these subregions. The AI, despite lacking a Nissl-defined L4, sparsely hosts neurons reminiscent of L4 morphologies (M-types 6, 7 and 10; Fig. 3c). In canonical granular cortices, L4 is considered the initiation layer of cortical processing that integrates thalamocortical excitation^30,31^. Whether these aforementioned AI neurons function as bona fide L4 neurons in an agranular cortex^65^ invites efforts to test whether they express the L4 marker *Rorb*^65^, send ascending inputs to L2 and L3 or receive thalamic inputs^30,31^. Although our dataset prioritized AI microcircuits, we did identify inputs from DI/GI to AI, in line with previous studies^6,21,26^. Future studies on DI/GI microcircuits will reveal whether six-layered IC subregions employ distinct local computations compared to the AI and whether information flows from deep layers of agranular regions to superficial layers of fully laminated subregions as proposed by tracing-based connectivity models^66^.

A striking feature of the IC is that its lateralized local inputs (Fig. 5d) extend well beyond the width of canonical cortical columns^67^. Although out-of-column cortical connections have been implied by axonal reconstructions and in vivo recordings^68,69^, our dataset provides direct physiological evidence for the extensive lateral reach of intra-insular integration across subregions along its anterior–posterior axis, distinguishing the IC from other cortices^48–52^. Our data further validated the connections between the anterior and posterior ICs previously inferred from anatomical and in vivo data^4,19,20^ and demonstrated its necessity in linking taste-related thalamic and valence-related amygdalar regions through the insula (Fig. 8)^10,14,56^. As these local inputs preferentially arrived from the posterior to the anterior IC, this result aligned with the tract-tracing-derived hypothesis that information flows from superficial to deep layers intra-cortically toward higher-order brain regions^66^ and provides physiological evidence for the previously proposed connectivity from the posterior to the anterior IC^4,9^. Notably, projections from the anterior to the posterior IC are also present^1,20^ (Fig. 5b,d), and this direction of connectivity has been shown to mediate the retrieval of a conditioned immune response^20^. Future studies investigating how these reciprocal anterior–posterior connections coordinate to evaluate sensory information and orchestrate bodily responses will provide further insights into the computational roles of the insula.

Local connections within the insula might also serve as a bridge between multiple topographically organized, long-range circuits. In addition to the VPMpc–insula–BLA pathway described in our study (Fig. 8g), inputs from both the VPMpc and posterior BLA have been shown to converge onto the midposterior insula^57^. Furthermore, the posterior IC projects to the CeA as well as to the posterior BLA^1,26^. Future work will be required to determine whether these distinct pathways connecting the insula, thalamus and amygdala differentially contribute to specific dimensions of sensory-valence processing.

### Limitation and future directions

Several aspects beyond the present scope can further enhance the comprehensiveness of our multimodal insular atlas. We usually recorded features across two or more modalities per neuron, but never all modalities simultaneously due to technical constraints. In our crossmodal analyses, morphology features provided the most cell-typing information, with several of them correlating with input and electrical features; we therefore used morphology as a gateway to connect other modalities. In addition, we demonstrated one example of using our dataset to facilitate circuit mapping; more applications of our dataset can be carried out by future studies.

## Methods

### Animals

All animal experiments were conducted according to the US National Institutes of Health (NIH) guidelines for animal research and were approved by the Oregon Health & Science University Institutional Animal Care and Use Committee. Male and female wild-type C57BL/6 mice (Charles River Laboratories) and *PV-Cre*, *Sst-Cre*, *Gad2-Cre*, *Drd1-EGFP*, *Dat-Cre*, *Fezf2-CreER*, *Ai32* and *Ai9* mice—all on a C57BL/6 background—were used (Supplementary Tables 4 and 5). *PV-Cre*^*+/−*^*;Ai9*^*+/−*^ mice were used to aid demarcation of the insula subregions during the establishing phase of the study. Mice were housed in group housing, given free access to food and water and maintained on a 12-h light-to-dark cycle, unless noted otherwise. Animal ages are specified in the following experiments.

### Atlas collection and histology

Four wild-type (two females and two males) and six *PV-Cre*^*+/−*^*;Ai9*^*+/−*^ (three females and three males) mice (age postnatal (P)49 ± 1 d) were used to develop a reference atlas of the insular cortex. Mice were transcardially perfused with ~50 ml of 4% paraformaldehyde (PFA) in 0.1 M phosphate buffer (PB), pH 7.2. Each brain was dissected from the skull and post-fixed overnight in 4% PFA, then stored in phosphate-buffered saline (PBS), pH 7.4, until sectioning. All subsequent steps were conducted at room temperature unless otherwise stated. To prepare for sectioning, each brain was dry blotted and the dorsal surface superglued onto a wedge (10° tilt, 2.5% agarose or aluminum) with the anterior side tilting upward. This slicing method was used throughout the study to preserve the functional anterior–posterior axis of the insula as well as its dendritic morphologies and is referred to as ‘modified horizontal’ sectioning for simplicity. The brain was then embedded in 3% agarose, blocked, then sectioned in this modified horizontal orientation at 40 µm in PBS on a vibrating microtome (Leica Biosystems, VT1200S). Alternating sections were either directly mounted onto slides (Superfrost Plus, Thermo Fisher Scientific) for Nissl staining or collected as free-floating sections in welled plates for PV immunohistochemistry.

Nissl staining was performed following a standard protocol^71^, and stained slides were coverslipped with Eukitt (Sigma-Aldrich, cat. no. 03989). For PV immunohistochemistry, sections were first rinsed 3× 10 min in Tris-buffered saline (TBS), then incubated with 3% H_2_O_2_ + 10% MeOH in TBS for 30 min to quench endogenous peroxidase activity, followed by another 3× rinse. Sections were then blocked for 1–2 h with 5% goat serum in TBS + 0.1% Triton X-100 (Sigma-Aldrich), then incubated overnight at 4 °C with mouse anti-PV primary antibody (Sigma-Aldrich, cat. no. P3088, RRID:AB_477329, 1:1,000) in the blocking solution. The next day, sections were rinsed 3×, then incubated for 1–2 h with horseradish peroxidase-conjugated goat anti-mouse secondary antibody (Jackson ImmunoResearch Laboratories, Inc., cat. no. 115-035-003, RRID:AB_10015289, 1:500) in the blocking solution. Sections were again washed 3×, then incubated for 90 min with ABC (Vectastain Elite ABC-HRP Kit, Vector Laboratories, cat. no. PK-6100), washed 3×, developed with nickel-intensified DAB (Sigma-Aldrich, cat. no. 5905) and washed a final time. Last, sections were washed in 0.05 M PB, mounted on microscope slides (Superfrost Plus) and coverslipped with Eukitt.

### Atlas microscopy and annotation

All slides were imaged on an Axio Scan.Z1 (Zeiss) using a ×20 objective. Image data were subsequently processed in FIJI^72^ (v2.14) using customized scripts. Each brain was first reconstructed in 3D by importing 16× downsampled images in serial order into TrakEM2^73^, where they were semi-automatically aligned across the cutting axis (Supplementary Fig. 1 and Extended Data Fig. 1b). This operation was completed separately for Nissl-stained and PV-stained slides. For later steps, a merged atlas was also created for each brain by interleaving the two histological datasets in serial cutting order and applying a 4% enlargement to PV sections to correct for the average difference in shrinkage artifact between the two methods. All stained slide series were manually inspected for histological quality—that is, absence of shrinkage and staining artifacts and contiguous undamaged sections containing the insula. No qualitative differences were observed between *PV-Cre;Ai9* and wild-type samples.

For Nissl reconstructions, the three highest-quality *PV-Cre;Ai9* specimens (one male and two female) were selected for further processing (Supplementary Fig. 1). Each section was manually annotated in TrakEM2 using a graphics tablet (Artist 22R Pro, XP-Pen or Cintiq 22HD, Wacom) to segment the following conspicuous cortical features: (1) the pial surface, (2) the border between cortical layers 1 and 2, (3) the superficial edge of the CC and (4) the pial indentation(s) corresponding to the MCA (Fig. 1 and Supplementary Fig. 1). Next, a 3D bounding box that contained the entirety of the right IC was established to spatially restrict subsequent analyses. Using the rigid affine transforms established by alignment in TrakEM2, a full-resolution region of interest (ROI) was extracted from each section that intersected with this bounding box, thus creating a spatially coherent image stack through the IC (Fig. 1b and Supplementary Fig. 1). For each section in the stack, a customized script utilizing the MorphoLibJ library^74^ automatically segmented the tissue from the slide background with occasional minor manual corrections when needed, and the image contrast was globally enhanced (normalized) using intensity data within the tissue mask.

Each image stack was loaded in Photoshop (v23; Adobe Inc.) where two expert annotators each independently delineated the boundaries of cortical layers by painting a color-coded overlay using the Pencil tool. Subregional bounds (for example, AI–DI border) were indicated (Supplementary Fig. 1). A histological atlas^44^ was used as a visual reference during annotation. A final consensus was reached through an averaging routine: identically annotated pixels were preserved by taking the intersection of the two overlays, after which an operational loop expanded each color-coded region into unallocated space by 1 pixel per iteration until the entire ROI bounded by the pial, CC and outermost subregional annotations was filled with coded pixels (Fig. 1c and Supplementary Fig. 1).

### Laplacian analysis

To register Nissl atlas locations into a reference coordinate space that accounts for the variable curvature and cortical thickness of the insula, we employed a Laplacian operator. Laplace’s equation was solved for the scalar field, ψ, enclosed between the two lines defined by annotations of the CC and L1–L2 boundaries described above in Nissl sections^75–77^. It takes the form:

$${\nabla }^{2}\psi =\frac{{\partial }^{2}\psi }{{\partial x}^{2}}+\frac{{\partial }^{2}\psi }{{\partial y}^{2}}$$

where *x* and *y* are the standard Cartesian coordinates in the Nissl section, ∇ the Laplacian gradient of the scalar field and ∂ the partial derivatives of the Cartesian coordinates. The L1–L2 boundary was used in lieu of the pial boundary because the latter was often subject to minor deformations and folds during tissue mounting that could misrepresent local curvature. Laplace’s equation was iteratively solved using Jacobi’s method, where the CC and L1–L2 boundaries were always fixed to 0.0 and 1.0, respectively, with standard iterative (*i*) relaxation taking the form:

$$\begin{array}{ll}{\psi }_{i+1}\left(x,y\right)=\left[{\psi }_{i}\left(x+\Delta x,y\right)+{\psi }_{i}\left(x-\Delta x,y\right)\right.\\\left.+{\psi }_{i}\left(x,y+\Delta y\right)+{\psi }_{i}\left(x,y-\Delta y\right)\right]/4.\end{array}$$

Following ref. ^76^, an additional ‘resistive’ boundary was imposed to account for the fact that the CC and L1–L2 boundaries do not intersect. All pixels outside the scalar field, ψ, enclosed between these lines were fixed to NaN and, with each iteration, calculations for edge pixels of ψ discounted contributions from any adjacent null pixels. The final equidistance field was then iteratively extrapolated in 1-px steps following two-point gradients until the entire ROI bounded by the pial and CC annotations was filled (Fig. 1d). Streamlines (that is, the steepest gradient descent paths) were then computed at any point within ψ between the pia and CC and were used to estimate local columnar orientation and cortical thickness.

### Insular flatmap

We transformed each brain into a 2D flatmap that topographically organized data with respect to (1) the relative section along the cutting axis and (2) the relative position along the laminar axis, which was defined as the mid-value (=0.5) isocontour line of the Laplacian gradient, ψ (Fig. 1d and Extended Data Fig. 1b,c) and approximated the anterior–posterior axis. To align data across the cutting axis in flatmap coordinates, a common reference was identified in the following way, which could be located at a specific point along the laminar axis of each section. Surface reconstructions of each right hemisphere were visualized with ParaView (v5.10; Kitware, Inc.). Using affine transforms obtained by solving linear systems—each containing 21–25 matched fiducial coordinates between pairs of brains—we aligned these surfaces within a common 3D reference frame (Extended Data Fig. 1a). Applying these transforms to the MCA annotation coordinates revealed remarkable consistency in the placement of the trunk of the MCA proximal to its bifurcation across all three animals (Extended Data Fig. 1a). We therefore established a common reference as follows: for each mouse, take the MCA annotation coordinate from each section that falls within 1 mm ventral (proximal) to its point of bifurcation (*n* = 12 sections; Extended Data Fig. 1a,b). Second, calculate the streamline that intersects with the MCA annotation of each section and take the coordinate of the intersection of this streamline with its respective laminar axis. Third, determine the plane that best fits these 24 coordinates (2 per section × 12 sections) in 3D space using singular value decomposition (Extended Data Fig. 1b). The point of intersection between this plane and the laminar axis of each section was defined as the section’s coordinate origin (Extended Data Fig. 1b,c, white circles) and all points or streamlines within a section were therefore plotted onto the flatmap in terms of their relative distance to this origin along the laminar axis. The cortical thickness on each flatmap was generated by sampling streamline lengths at 25-µm intervals along the laminar axis for each section across the cutting axis (Extended Data Fig. 1c,d).

To define the subregional bounds of the insula flatmap—anterior edge, AI–DI border, DI–GI border and posterior edge—the intersections of each annotator’s lines with the laminar axis were identified, converted to relative positions along this axis and averaged (Fig. 1b,c, Supplementary Fig. 1, black dashed line, and Extended Data Fig. 1d, white dashed line). The coordinates of the cutting axis were also adjusted to make them relative to the ventral-most section that contained an annotation for L4 (Supplementary Fig. 1, dashed box and Extended Data Fig. 1d, white diamonds). The orientation of the long axis (Extended Data Fig. 1d, black dashed) for each insular flatmap was defined as the angle between the cutting plane and the major axis of an ellipse fit to the outermost boundaries of the insular flatmap; the short axis was orthogonal to this angle. This ellipse was calculated as the area having the same second-order central moments as the insular flatmap region.

To compare data across brains, each flatmap was rotated about its 2D origin (defined by the L4-MCA origin; Extended Data Fig. 1d, white diamonds) such that the long axes were parallel across flatmaps and overlaid (Extended Data Fig. 1f). Average laminar boundaries were generated as follows: for each subregional boundary, coordinates from the three individual aligned maps were binned and averaged at 250-µm intervals along the cutting axis, then smoothed with a cubic spline (Extended Data Fig. 1g). The average insula was defined as the area between the average anterior and posterior bounds along the laminar axis. The average GI was defined as the area between the average DI–GI border and the posterior edge. The average DI was defined as the area between the average AI–DI border and the posterior edge, minus the GI area. The remaining area defined the average AI. An equivalent operation was applied to the error bounds (±1 s.d.) of each edge (Extended Data Fig. 1g, gray shading).

### Cortical layer analysis

Layer annotations were transformed to the flatmap space using streamlines spaced at 25-µm intervals along the laminar axis, as above (Fig. 1d and Supplementary Fig. 2). The pixel color or values of each annotated painting were queried at 0.1-µm steps along each streamline, yielding the thickness of each cortical layer at a given laminar position (Supplementary Fig. 2a,b). Dividing these values by the total length of each streamline (that is, cortical thickness) produced normalized values. Thicknesses for all sections were then assembled within the 2D flatmap to highlight topographic variation (Supplementary Fig. 2c).

As each reference mouse brain required a slightly different rotation to its flatmap (Extended Data Fig. 1f), the individual 2D sample grids (Supplementary Fig. 2c) did not precisely overlap when overlaid on each other. Therefore, a grand 2D average of normalized thickness for each layer was computed by binning and averaging values from each mouse brain at 100-µm and 250-µm intervals along the long and short axes, respectively, relative to the MCA origin, and finally averaging across mice (Supplementary Fig. 3a). This operation was run separately for bins assigned to AI or DI/GI (Supplementary Figs. 2c and 3a) due to the presence or absence of L4 annotations.

### Stereotaxic injections

Wild-type C57BL/6 mice (age P21–P35) were anesthetized with 2% isoflurane and injected with adeno-associated virus (AAV) and/or fluorescent retrograde microbeads (LumaFluor, Inc.) as previously described^40^. For retrograde labeling of AI output neurons, 30–60 nl of red retrograde beads (Red Retrobeads IX, 1:1 diluted in filtered PBS) or green retrograde beads (Green Retrobeads IX, 1:1 diluted or undiluted) were injected in up to two selected projection target regions per animal, namely contralateral AI, Ent, NAc, BLA, CeA, MD, PF/PO and NTS. For retrograde labeling of thalamic nuclei projecting to the posterior AI, 30 nl of red retrograde beads (1:1 diluted in filtered PBS) was injected in the ipsilateral posterior AI. For retrograde labeling of Fezf2^+^ insular neurons projecting to the NAc, 40–80 nl of AAVretro-FLEX-tdTomato virus (Addgene, cat. no. 28306; titer: 2.1 × 10^13^ GC per ml) was injected into the NAc of *Fezf2-CreER* animals. For CRACM experiments, 30–40 nl of AAV1 ChR2-Venus (Addgene, cat. no. 20071; titer: 1.8 × 10^12^ GC per ml) or AAV1 hChR2-TdTomatoH134R virus (Penn Viral Core, lot V0314; titer: 6.7 × 10^12^ GC per ml; also available from Addgene, cat. no. 28017) was injected in the ipsilateral VPMpc/PF thalamus. For CTA experiments, 100–120 nl of AAVretro-Cre virus (Addgene, cat. no. 105553; titer: 1.8 × 10^13^ GC per ml) was injected bilaterally in the posterior IC and 60–80 nl of AAV5-CAG-FLEX-TeLC-GFP (a gift from E. Schnell’s laboratory; titer: 1 × 10^13^ GC per ml) or AAV5-CAG-FLEX-GFP (a gift from E. Schnell’s laboratory; titer: 1 × 10^13^ GC per ml) in the VPMpc thalamus. Note that, due to technical limitations, thalamic injections often targeted more than one nucleus. PF/PO injections were aimed at the PF and partially labeled the adjacent PO region; VPMpc/PF injections were aimed at both VPMpc and PF, and sometimes led to minor spillover in neighboring nuclei (Extended Data Fig. 10c), which was negligible because they did not project to the IC (Extended Data Fig. 10a,b). Cre-dependent expression for the CTA experiment was restricted to the VPMpc → posterior IC pathway. Injection coordinates are measured in micrometers relative to bregma (unless noted otherwise) along anterior–posterior (AP), medial–lateral (ML) and dorsal–ventral (DV) axes as follows: contralateral AI (AP: 1,350 to 1,700; ML: −2,850 to −2,900; DV: 3,700 to 3,800), BLA (AP: −1,350; ML: 3,100; DV: 5,100), CeA (AP: −1,050; ML: 2,600; DV: 4,750), MD (AP: −1,050; ML: 250 to 300; DV: 3,400), PF/PO (AP: −2,050 to −2,175; ML: 550 to 600; DV: 3,400), NTS (AP: −6,200; ML: 1,150; DV from brain surface: 3,900), posterior AI (AP: 750; ML: 3,550; DV: 3,900), entorhinal cortex (AP: −2,500; ML: 4,200; DV: 4,200), NAc (AP: 1,100; ML: 1,450 to 1,600; DV: 4,500 to 4,900) and VPMpc/PF (AP: −2,050 to −2,175; ML: 550 to 600; DV: 3,900).

### Tamoxifen induction of *Fezf2-CreER* animals

*Fezf2-CreER* animals received an intra-peritoneal injection of 1 mg of tamoxifen (5 mg ml^−1^ dissolved in corn oil; Sigma-Aldrich, cat. no. T5648) 7 d after stereotaxic surgery. For a subset of animals, the tamoxifen injection was repeated 2 d after the initial treatment. Brains were collected for experiments at least 7 d after the initial tamoxifen induction. Both induction strategies labeled an adequate amount of Fezf2^+^ neurons for whole-cell recordings.

### Electrophysiology recordings

To preserve the functional anterior–posterior axis within the insula, slices were prepared in a modified horizontal orientation. For intrinsic property experiments, brain slices were prepared from P21–P107 animals (median = P38 for animals included in electrical feature-based iCCA; median = P40 for animals included in morphological feature-based iCCA); for LSPS, brain slices were prepared from P29–P59 animals (median = P38). Brains injected with retrograde beads and/or virus at P21–P35 were sliced 3–14 d after bead injection or 14–28 d after viral infection of ChR2-expressing AAV. Wild-type C57BL/6 or *PV-Cre*^+/−^;*Ai9*^+/−^ mice were transcardially perfused with artificial cerebrospinal fluid (aCSF) cutting solution (in mM: 127 NaCl, 25 NaHCO_3_, 1.25 NaH_2_PO_4_·H_2_O, 25 glucose, 2.5 KCl, 2 CaCl_2_and 1 MgCl_2_) and decapitated to obtain modified horizontal brain slices as described above. Perfusion and slicing aCSF temperature was matched to subsequent slicing protocol. For intrinsic property recordings, brains were sliced at 300-µm thickness with warm (~34 °C) aCSF cutting solution. For LSPS and ChR2 experiments, brains were sliced at 350-µm thickness with ice-cold choline cutting solution (in mM: 25 NaHCO_3_, 1.25 NaH_2_PO_4_·H_2_O, 25 glucose, 2.5 KCl, 7 MgCl_2_·6H_2_O and 0.5 CaCl_2_; with 110 choline chloride, 11.5 sodium ascorbate and 3 sodium pyruvate added freshly before the experiment). Whole-cell recordings were performed using a Multiclamp 700B amplifier (Molecular Devices) with borosilicate pipettes (3–6 MΩ; Sutter Instruments) under an Olympus microscope (BX51W with ×60, 1.0 numerical aperture (NA) water immersion objective), and data were collected using Ephus software^78^ within MATLAB (v2007b; Mathworks). A cesium-based internal solution was used for voltage-clamp recordings (in mM: 135 Cs gluconate, 1 ethylenebis(oxonitrilo)tetra-acetate (EGTA), 1.5 MgCl_2_, 10 Hepes, 2 Na_2_ ATP, 0.3 GTP, 7.8 Na_2_ phosphocreatine and 0.015 Alexa Fluor-594 (Invitrogen), adjusted to pH 7.35–7.4 using CsOH, ~280 mosmol, with 3 QX-314·chloride (Tocris Biosciences, cat. no. 2313) added before experiments) and a potassium-based internal solution was used for current-clamp recordings (in mM: 120 K gluconate, 5 NaCl, 2 MgCl_2_, 10 Hepes, 1.1 EGTA, 4 Mg_2_ ATP, 0.4 Na_2_ GTP, 15 Na_2_ phosphocreatine and 0.015 Alexa Fluor-594, adjusted to pH 7.25 using KOH, 290 mosmol); 3–5 mg ml^−^^1^ of biocytin (Sigma-Aldrich, cat. no. B4261) was included in the internal solutions for morphological reconstructions. For all experiments, brain slices were continuously perfused in aCSF (in mM: 127 NaCl, 25 NaHCO_3_, 1.25 NaH_2_PO_4_·H_2_O, 25 glucose, 2.5 KCl, 2 CaCl_2_ and 1 MgCl_2_) with *N*-methyl D-aspartate receptor blocker (R)-CPP (5 μM; Thermo Fisher Scientific, cat. no. 02-471-0). Intrinsic property recordings were performed at near-physiological temperatures (30–34 °C); LSPS and ChR2 recordings were performed at room temperature (~23 °C). All recordings were performed in the right hemisphere. At the conclusion of each recording, a microphotograph was captured using a low-magnification objective (×4, 0.16 NA; UPlanApo, Olympus) used for the estimation of cortical soma position and shrinkage factor.

Intrinsic property recordings consisted of three current-step protocols during which sweeps were recorded at 5-s inter-sweep intervals: (1) a current-voltage protocol with repetitive sweeps containing 200-ms-long square pulse current steps starting at −300 pA with 25-pA increments every fourth sweep until 625 pA was reached or spike generation was occluded by depolarization-block; (2) a ramp protocol with stimulation sweeps consisting of a 3-s-long rising current injection at a rate of 165 pA s^−^^1^, repeated for 10 sweep iterations; and (3) a spike train protocol with repetitive sweeps containing 1-s-long current steps, starting at 0 pA with 50-pA increments every fourth sweep until 1,050 pA was reached or spike generation was occluded by depolarization block.

### Biocytin labeling and staining

During whole-cell recordings, neurons were filled with internal solutions (see above) containing 3–5 mg ml^−^^1^ of biocytin and subsequently visualized using DAB staining (adapted from ref. ^79^). Briefly, after recording, slices were fixed for 12 h in a 4% PFA solution and stored in PB until staining. After 6× 10-min washes in PB, slices were incubated 2× for 20 min in 3% H_2_O_2_ in PB solution to quench endogenous peroxidases. Slices were washed 4× for 5 min in PB and incubated for 1 h at room temperature, followed by overnight incubation at 4 °C and then again at room temperature for 1 h the following day in PB containing 1% Vectastain ABC reagent (Vector Laboratories) and 0.1% Triton X-100 (Sigma-Aldrich). After 6× 10-min washes in PB, slices were incubated for 30 min in cobalt-and-nickel-intensified DAB in PB solution (2 μl of CoCl_2_ and 4 μl of (NH_4_)_2_Ni(SO_4_)_2_ per 1 mg ml^−^^1^ of DAB) at 4 °C. Slices were developed using 8 μl of 3% H_2_O_2_ in PB solution per slice for 15 s to 2 min before being washed 3× for 10 min in PB, with the final wash containing a 1:5,000 or 1:10,000 dilution of Hoechst 33342 (Thermo Fisher Scientific, cat. no. H3570). Slices were then mounted using spun-down Aqua-Poly/Mount (Polysciences, cat. no. 18606-5) medium and the coverslip was sealed with clear nail polish (Super Shine, Sally Hansen). Slices were dried and settled for a minimum of 2 weeks before morphological reconstruction.

### Morphological reconstruction and processing

Biocytin-labeled neurons were viewed under an Axio Imager.M2 microscope (Zeiss) at ×4 magnification to assess filling and staining quality, as well as to identify cell locations. Cells were inspected for intact apical dendrites that extended all the way to the pial surface (except some L6 cell types), a dark biocytin fill that persisted to the distal parts of both apical and basal dendrites and the presence of dendritic spines to confirm an excitatory neuronal identity. Neurons were reconstructed manually using Neurolucida (v2017.0.2; MBF Bioscience) at ×40 magnification with an oil objective (EC Plan-Neofluar ×40, 1.3 NA, Zeiss). Cell bodies, apical dendrites, basal dendrites and axons were traced and annotated. All processes were traced to their termination points, a truncation point or location where staining could no longer be visualized. Section contours were traced in three dimensions. The pial edge was traced along the top and bottom surfaces of each slice. Additional contours were traced along the outline of multiple reference structures (lateral ventricles, pial surface, CC, fimbria, fornix, internal capsule and outline of the right hemisphere) as landmarks.

Individual reconstructed cells were isolated from original Neurolucida files (DAT) containing multiple cells on the same acute slice and converted to SWC+ files using the HBP Neuron Morphology Viewer^80^. Individual SWC+ files were then processed in Python (BrainCellSuite Python package: https://gitlab.com/maolab/braincellsuite_submission) to extract cortical thickness, correct for post-fixation slice shrinkage and set the coordinate origin at the soma center. The slice shrinkage was calculated as the ratio between pia–soma distance in the biocytin-stained section and pia–soma distance before fixation. The average of this post-fixation slice shrinkage was 0.96 ± 0.14 (mean ± s.e.) and 0.49 ± 0.15 across the *x*–*y* plane and *z* depth, respectively. The 3D morphology plots and flattened 2D images were then generated from these processed files (BrainCellSuite). The 3D morphology plots were used to check reconstruction quality. Cells with apical dendrites that were truncated due to slicing or were missing apical and/or basal dendrite annotations due to poor staining quality were excluded from the dataset. Processed morphologies were then used for feature extraction.

### Cell assignments

Reconstructed cells were matched to our histological atlas of the insula (Extended Data Fig. 2c), thus allowing us to estimate a flatmap coordinate for each cell that consists of: (1) slice position along the cutting axis; (2) anterior–posterior position along the laminar axis; and (3) normalized cortical depth.

After post hoc recovery of cell morphology (Extended Data Fig. 2e,f), each DAB-stained slice was inserted into the PV atlas for ‘Mouse #2’. The PV atlas was used because it also utilized DAB processing and thus appeared texturally similar to the recovered acute slices. As acute slices were thicker (300–350 µm) than atlas sections (40 µm, every other; see above), each acute slice was compared to a range of atlas sections before inserting it into the middle of the matched stack within the atlas’s TrakEM2 project. Within TrakEM2, the slice was manually aligned to the reference atlas section. We prioritized the matching of features (for example, pial and CC edges, MCA) that were proximal to the labeled cells to minimize the effects of tissue distortions that can accumulate with histological processing. Each acute slice was independently matched to the atlas and had no bearing on the placement of the other slices.

Once aligned to the PV-atlas reference frame (Extended Data Fig. 2g), the coordinate position for the soma of each recovered cell was noted, then shifted −40 µm along the cutting axis to place it within the adjacent, interleaved Nissl atlas reference frame (Extended Data Fig. 2h, open circles), yielding the first flatmap coordinate component: slice position. A streamline was then calculated that intersected with the soma (Extended Data Fig. 2h, vertical dashed lines). The crossing of this streamline with the mid-value Laplacian isocontour (Extended Data Fig. 2h, horizontal dashed line) yielded the second flatmap coordinate component: anterior–posterior position. Last, the soma location was corrected by shifting it along the streamline such that its normalized pia-to-CC position matched the measurement obtained from the acute slice (Extended Data Fig. 2h, filled circles), yielding the third flatmap coordinate component: cortical depth. When possible, this depth value was measured from pipette position during electrophysiology (for example, Extended Data Fig. 2d); otherwise it was obtained after morphological recovery (for example, Extended Data Fig. 2e). The first two dimensions of the flatmap coordinate were interchangeable with the long-flatmap and short-flatmap axes using a change of basis based on the 2D rotation of the ‘Mouse #2’ flatmap about the MCA origin (see above; slice and anterior–posterior positions). Using their estimated flatmap coordinates, we assigned each reconstructed cell to both an insular subregion and a cortical layer (Fig. 1f,g and Extended Data Figs. 1h and 2). A handful of cells (14 out of 1,109) with estimated topographical positions that fell just outside the bounds of the average flatmap, yet remained within the error margins, were assigned to the nearest subregion within the same slice position (Fig. 1f).

### Imaging and quantification of retrograde fluorescent bead distribution

To quantify the distribution of labeled AI output neurons along cortical depth, brains from wild-type C57BL/6 mice injected in the contra AI, Ent, NAc, BLA, CeA, MD, PF or NTS with retrograde beads were perfused, sliced at 50-µm thickness, stained with Hoechst 33342 (1:5,000), mounted in aqueous mounting medium (FluoroMount-G, Sigma-Aldrich) and tile imaged with a ×10 or ×20 objective on an Axio Imager.M2 microscope (Zeiss). For each brain, bead-positive cells were manually counted in FIJI^72^. The relative cortical depth of each bead-positive cell was calculated by normalizing its distance to pia over the average cortical thickness in an ~0.3-mm wide rectangular ROI surrounding the cell that covered the full cortical depth. The normalized distribution of bead-positive neurons was then averaged across slices for each projection target (Fig. 3e; *n* for each group: AI: three males, one female; Ent: two males, one female; NAc: three males; BLA: three males, two females; CeA: three males, two females; MD: two males, one female; PF/PO: five males; and NTS: two males, one female). To identify thalamic projections to posterior AI (Fig. 8 and Extended Data Fig. 10a,b), brains injected in the posterior AI with retrograde beads were perfused, sliced at 100-µm thickness, mounted in aqueous mounting medium (FluoroMount-G) and imaged under the bright-field and red fluorescence channels on an Axio Zoom.V16 microscope (Zeiss).

### Laser scanning photostimulation

LSPS was performed as previously described^81^. Briefly, MNI-caged-L-glutamate (200 μM, Tocris Biosciences, cat. no. 1490) was uncaged in confined areas by 1-ms collimated ultraviolet laser stimulation (DPSS Lasers, Series 3500), with 20 mW of light power measured after the objective (XLFLUOR2X/340, 0.14 NA, Olympus Corporation). For each map, stimulation sites were positioned across a 75 µm × 75 µm-spaced grid, with light pulses given at each site (Extended Data Fig. 7a; beam size: ~60 µm, full-width at half-maximum light intensity) in pseudo-randomized order that maximized the distance between consecutive stimuli using galvanometer scanners controlled by Ephus software^78^. Postsynaptic pyramidal cells were recorded in voltage clamp at −70 mV, with map recordings repeated 2–4×.

For each neuron, a local input map was generated by thresholding at 6× the s.d. of the baseline, averaging traces at each stimulation site across trials, then integrating responses within a 75-ms window after laser onset. The map was then plotted as a heatmap and overlaid with the reconstructed morphology of the recorded cell after accounting for its post-fixation shrinkage factor (Extended Data Fig. 7b,c). For stimulation sites that overlap with the dendritic morphology, currents elicited by direct activation of glutamatergic receptors on the recorded neurons were separated from synaptic responses by their fast onset (20% peak response time <10 ms) and were excluded from connectivity analysis. Next, to account for cortical curvature and differences in cortical thickness along the anterior–posterior axis, individual heatmaps were interpolated along each stimulation column between pia and CC to produce the same number of response pixels per column (*n* = 22 pixels; Extended Data Fig. 7g). To generate the average input maps, interpolated heatmaps were either normalized to the maximum response of individual maps (Extended Data Fig. 7e) or kept as absolute mean without normalization (Fig. 5b,d and Extended Data Fig. 7d), and then grouped by soma layer location (Fig. 5b and Extended Data Fig. 7d,e). To average, individual maps were aligned either by pia such that all somas were distributed by their cortical depth on the vertical axis and shared the same relative anterior–posterior location on the horizontal axis (Fig. 5b and Extended Data Fig. 7e) or additionally by soma such that all somas were also positioned to the same location at the center depth of each layer group (Extended Data Fig. 7d). After alignment and averaging, all pixels with sample repeats (*n* ≥ 2) were displayed, and pixels at the horizontal edges with single samples (*n* = 1) were hidden. For visualization only, averaged heatmaps were Gaussian filtered, contoured at the 15th and 85th percentiles of all responses and overlaid on heatmap displays (Fig. 5b,d and Extended Data Fig. 7d,e). To generate the circuit diagram (Fig. 5c), absolute mean input strength was calculated by summing the absolute inputs arriving from each input layer and dividing by the number of recorded postsynaptic neurons in the receiving layer; only inputs with mean strength >0.1 nA were shown. In this circuit diagram, within-column and laterally extending inputs were calculated separately based on the 2.5th to 97.5th percentile horizontal range of summed dendritic densities of all morphologies included in this dataset, spanning 342 µm (5 proximal columns, rounded to 5 columns × 75 µm per column = 375 µm) laterally on each side of the soma. Within-column inputs were defined as local inputs arriving within this dendritic density range and laterally extending inputs were defined as distal inputs arriving from beyond this border (≤375 µm or >375 µm from the soma, respectively). Input from L4 was not included in the analysis because L4 is absent in the AI and outside the stimulation area for a subset of neurons.

To generate the probability map of input occurrence (Extended Data Figs. 7f and 8c), interpolated heatmaps were binarized by assigning responsive or nonresponsive pixels with values of 1 or 0, respectively. The binary maps were then aligned by pia, grouped by soma layer location (Extended Data Fig. 7f) or input profile (Extended Data Fig. 8c) and averaged across the same stimulation sites. To aid visualization, the 20% occurrence rate was calculated from Gaussian-filtered counterparts of the averaged binary maps and overlaid on top of the unfiltered averaged binary maps. Interpolated heatmaps were collapsed horizontally or vertically to one dimension by summing along rows or columns, respectively (Extended Data Fig. 7g–i). Individual collapsed maps were grouped by morphology type (Extended Data Fig. 7h,i) and averaged. Of note, the horizontally collapsed maps (Extended Data Fig. 7i) show the anterior–posterior extent of local excitatory inputs from left to right. To calculate normalized input strength arriving at each AI cortical layer (Extended Data Fig. 7j), within-column and laterally extending inputs from each layer were first normalized to the total cortical inputs of each cell and then averaged across cells of the same soma layer.

### Feature extraction

#### Cell morphology feature extraction

The L-measure package^82^ was used to calculate the numbers of stems, bifurcations, branches and tips of the dendritic tree as well as dendritic width, height, length, Euclidean and path distances, branch order, contraction, partition asymmetry and local and remote bifurcation amplification. All remaining morphological features (Supplementary Table 1) were extracted using custom-written software (BrainCellSuite). For features that described dendritic length within a specified cortical laminar bin—that is, a volume defined by an upper and lower border (*y* axis, infinite along *x* and *z* axes) parallel to the pial surface—we summed the 3D segment lengths that fell within each specified bin. In cases where a dendritic segment traversed multiple bins, the segment was split at its intersection with each border plane; the resulting subsegments were included in the calculations for their respective bins. To calculate the overlap between apical and basal dendrites, we first established for each morphology a 3D density matrix with a voxel size of 18 × 18 × 18 µm^3^ based on the dataset averaged soma diameter of 18.07 µm. We then registered the total apical and basal dendritic lengths separately per voxel. We calculated the number of voxels that contained both apical and basal dendrites as a fraction of all voxels that contained a dendrite and the total dendritic length within voxels that contained both apical and basal dendrites as a fraction of the total dendritic length. For the 18-µm laminar features, we first collapsed voxels along the *y* and *z* axes—that is, parallel to the pial surface—into laminar cortical bins and then calculated the same fractions as stated above. The voxel-based, apical–basal overlap features were log_2_-transformed to reduce their strong non-normal distribution, skewness (0.96 and 0.96, respectively) and kurtosis (1.26 and 1.07, respectively).

#### Electrical property feature extraction

Custom-written software (BrainCellSuite), which included the importing of Ephus data files (XSG), was used. Feature details are described in Supplementary Table 1. A measured liquid-junction potential of 14 mV was not corrected for. Spike detection was performed on 1-kHz low-pass, Bessel-filtered signal traces in which putative spikes were identified using the rise rate criterion ≥20 mV ms^−1^. Spike characteristics (features) of the identified putative spikes were then extracted from the raw signal traces. Spiking behavior features were extracted from traces with a depolarizing 1-s-long current step at rheobase plus 125 pA.

Summary plots of electrical properties were made for each recorded cell, which included access and input resistance, resting membrane potential and Δ membrane potential between the start and end of recorded sweeps, action potential absolute peak and relative to spike onset and noise and patch root mean squared (1.5-ms and 50-ms periods, respectively). Large deviations across sweeps were manually inspected and individual sweeps were omitted when deviations indicated technical noise or loss of whole-cell mode. Cells were included for iCCA analysis when access resistance was <40 MΩ, action potential threshold ≤−20 mV and amplitude ≥40 mV, and single action potential and spike train data were available.

#### LSPS map feature extraction

The interpolated averaged input maps that were normalized to the maximal input were used. Pixel-wise (dis)similarity measurements, such as mean squared error or structural similarity index^83^, were attempted as distance matrices for the iCCA and were found to be confounded by the exclusion of direct excitation from recorded neurons. Instead, we extracted input features that resembled input strength and number based on local layer and soma-centric proximity allocations. To do so, the assigned location of each soma on our insular atlas was used as a reference point for registering its input map to the insular atlas, thus allowing us to allocate each input location of the interpolated input map to a cortical layer. Next, we allocated the columns of the input map to either proximal or distal input sites based on two levels of a lateral input border: The dendritic density-based lateral border was based on the 2.5th to 97.5th percentile range of the summed dendritic densities (375 µm from the soma, defined above). The dendritic reach-based lateral border was based on the 2.5th to 97.5th percentile range of the summed most extreme dendritic reach of all morphologies in this dataset, spanning 675 µm (9 columns) laterally on each side of the soma. Using these two lateralization levels and layer allocations, we calculated relative input strengths and numbers of input locations (Supplementary Table 1). To calculate columnar input probability (Extended Data Fig. 8f), we converted the columnar collapsed input maps to a binary mask (that is, columns with at least one input location that yielded one). The input probability was calculated as the fraction of cells with an input location in the given column over the total sampled cells.

### Iterative consensus cluster analysis

The iCCA was performed in two phases: first, on principal component analysis (PCA)-transformed datasets and, second, on sets of biologically interpretable features (Extended Data Fig. 3a and Supplementary Table 2).

#### PCA feature-based CCA

During the first phase, we determined the number of putative clusters using PCA-transformed data as the input data for consensus clustering. We established, per dataset modality (morphology, electrical properties, input source or ‘crossmodal’ combinations thereof), 1,000 iterations of 90% subsampled datasets with each PCA transformation. For each subsampled dataset, we determined the optimal number of principal components (PCs) using column-wise *k*-fold crossvalidation (MEDA toolbox)^84^. In short, a PCA model containing a variable number of PCs was fitted to each subsampled dataset and used to reconstruct the original subsampled dataset based on a single column (that is, feature). An estimation error was calculated by comparing the reconstructed and original datasets^84^. This process was repeated for all features and the resulting summed estimation error was used to identify the ‘optimal’ number of PCs for the subsampled dataset, defined by the lowest summed estimation error (Supplementary Table 2). Hierarchical cluster analysis (HCA, Euclidean distance and Ward linkage) was performed on each PCA-transformed subsampled dataset for which cluster allocations were determined using the cutreeDynamics function (dynamicTreeCut Python package)^85^. The resulting dendrogram allocated leaves (that is, individual cells) to the clusters based on a set of criteria, including minimal number of cluster members (*n* = 3 cells), intra-cluster cohesion and inter-cluster distance, which were collectively controlled by one hyperparameter: ‘deepsplit’. To evaluate the cluster results, a pairwise co-cluster score was calculated by taking the number of HCA iterations during which a cell pair was allocated to the same cluster (irrespective of the cluster identity across HCA iterations) relative to the total HCA iterations during which the cell pair was included (±900 iterations). The co-cluster score matrix therefore resembled a pairwise similarity score for which a score of 1 indicated that the cell pair was always allocated to the same cluster. The inverted co-cluster matrix (1 − co-cluster matrix) was used as the distance input matrix for a second HCA (Ward linkage) to allocate all frequently co-clustered cell pairs to the same cluster (that is, consensus clustering). Based on this consensus clustering, we identified the most informative features that resembled distinct characteristics between pairs of clusters. To do so, we performed cluster pairwise feature comparisons using the Mann–Whitney *U*-test with a post hoc Bonferroni’s correction for multiple testing and selected all features (referred to as informative features; Supplementary Table 2), for which at least one cluster pair displayed significantly distinct feature distributions with the minimal fold-change criteria (morphological ≥log_2_(1.75) and electrical, input or crossmodal ≥log_2_(1.25)).

#### Biologically interpretable, feature-based CCA

After generating a list of informative features from the above analysis, we performed the second phase of iCCA (Extended Data Fig. 3a) to identify clusters (cell types) based on biologically interpretable features. First, to reduce the number of highly correlated and concentrated features (that is, low coefficient of variation), we performed a grid search with unique feature sets using all combinations of criteria mentioned above for the Spearman’s correlation (≥0.70 − 1 with 0.05 increments) and the coefficient of variation (≤0.1–0.55, with 0.05 increments). With each unique feature set (Supplementary Table 2), we repeated the above-described consensus cluster analysis. In this part, however, instead of PCA-transformed data, the 1,000 iterations of 90% subsampled datasets were composed of *z*-score-transformed unique feature sets. The cutreeHybrid ‘deepsplit’ hyperparameter (dynamicTreeCut Python and R packages) level for the HCA on the individual subsampled datasets was kept the same as chosen for the PCA-transformed data; for the second ‘consensus’ HCA, all hyperparameter levels were explored. To identify the feature set with the optimal consensus cluster result (based on the co-cluster scores), we compared the cluster results of all unique feature sets using the silhouette score while considering multiple ‘deepsplit’ hyperparameter levels (0–4), which produced increasing numbers of clusters. The feature set and ‘deepsplit’ parameter that yielded the highest silhouette score with a minimum number of clusters were deemed the optimal feature set and cluster allocation hyperparameter, respectively (Extended Data Fig. 3c and Supplementary Table 2). In addition to optimal cluster allocations determined by the highest silhouette score (for example, *k* = 21 for M-types), we also showed a dendrogram cut level at *k* = 5 determined empirically for all iCCA analyses (Fig. 4a, Extended Data Figs. 3d, 6a and 8a and Supplementary Figs. 6a, 14a and 15a) to display reduced clusters at a higher-level hierarchy and to reveal groupings of clusters allocated by the highest silhouette score.

To display the cluster results in a reduced dimensionality, we performed *t*-SNE^86^ (scikit-learn Python package^87^). The *t*-SNE approach consists of two phases: in the first phase, it models the pairwise distances for all datapoints in the high-dimensional dataset as Gaussian distributions; in the second phase, it optimizes the distances (represented as *t*-distributions) of a randomly instantiated 2D data representation to resemble the distances in the high-dimensional dataset. The resulting optimized 2D representation mimics the local structure of the original high-dimensional dataset. The *z*-score-transformed datasets of the selected feature sets were used as input data and *t*-SNE model parameters were either held constant (learning rate: 500; iterations: 5,000; random initialization seed) or tuned (perplexity: 8–40; early exaggeration: 1–10) to obtain the best possible visualization with low intra-cluster and high inter-cluster distances among cells.

### Simulation

Two approaches were taken to study the relationship between dataset size and number of identified clusters (Extended Data Fig. 3e). First, a subsampling-based approach was used without corrections for data point imbalance (for example, the frequency of cells located in the different cortical layers differs). We then assessed at which dataset size we could have stopped collecting data and obtained the same number of clusters, keeping all cluster analysis parameters fixed. Next, we assessed the upper bound of identifiable clusters and its relationship to dataset size based on the data distribution. As mentioned previously, the sampling frequency of cells across cortical layers was not homogeneous; for example, deep L6 cells were less abundant in our dataset compared to L5 cells. To account for such imbalances in our simulation experiment, our second approach started by creating additional ‘fictive’ cells among these minority groups—located at supragranular and infragranular cortical depths—by imputing morphological feature datapoints using the synthetic minority oversampling technique (SMOTE; imbalanced-learn Python package)^88,89^. The SMOTE-generated dataset was then fit with multiple Gaussian mixture models (GMMs; scikit-learn Python package; built with component-wise general covariance matrices; maximal number of expectation-maximalization iterations: 1,000; number of initializations: 30) composed of varying numbers of components. Using the Bayesian information criterion, we selected the GMM with the fewest number of components and the lowest Bayesian information criterion score. The simulated datasets of varying sample sizes containing feature-based ‘fictive’ cells were generated using the selected GMM (six components). Both the subsampled and simulated datasets were used for consensus clustering as described above. Like the original data analysis, the same feature set (Extended Data Fig. 3e) and cutreeHybrid ‘deepsplit’ hyperparameter (level 2) during the HCA with 90% subsampled data were used (Extended Data Fig. 3d). The consensus cluster analysis for each tested dataset size (subsampled datasets: 10, 20, 50, 100, 150, 200, 300, 400, 500, 600, 700, 800 or 900 cells; simulated datasets: 10, 20, 50, 100, 150, 200, 400, 600, 800, 1,000, 1,200, 1,400 or 1,600 cells) was performed for 20 independently initialized instances. The recorded cluster number (Extended Data Fig. 3e) was based on either a fixed ‘deepsplit’ hyperparameter (level 2; subsampled datasets) or the maximal possible cluster number based on all five assessed ‘deepsplit’ levels (simulation datasets). The cluster number versus dataset size results were modeled with a five-parameter logistic regression that takes the form:

$$y=d+\frac{(a-d)}{{\left[1+{\left(x/c\right)}^{b}\right]}^{g}}.$$

Model parameters were obtained using nonlinear least squares to fit to our data (simulated datasets: *a* = −1.967, *b* = 0.9569, *c* = 1.964 × 10^10^, *d* = 21.91 and *g* = 1.105 × 10^8^; subsampled datasets: *a* = 0.8220, *b* = 1.967, *c* = 18.45, *d* = 30.12 and *g* = 0.1750).

### Comparative morphological analysis

Dendritic morphology datasets from mouse V1^33^, mouse M1^41^ (from NeuroMorpho.org, RRID:SCR_002145)^90^, macaque motor and temporal cortex^42^ and human middle temporal gyrus^43^ were obtained from their respective repositories or kindly provided by the authors. Morphological features were extracted using the same pipeline as our insular dataset except with a Python-wrapped implementation of L-measure software (PyLMeasure Python package: https://pypi.org/project/pylmeasure/) and our custom-written analysis code (BrainCellSuite). Note that context-dependent features, which quantified morphological aspects relative to the local cortical thickness, were available only from the mouse V1 dataset. The extracted data were crossreferenced and verified with the published metadata, including projection type, and feature datasets. Differences in tracing conventions led to a submillimeter underestimation of dendritic lengths using L-measure for the mouse M1 dataset; therefore, we extracted the total dendritic length using custom-written analysis code.

### Classifier training

Random forest classifiers (scikit-learn Python package) were trained on 75% of the dataset using untransformed features from morphological, electrical or input modalities or crossmodal combinations thereof (Fig. 7a). Classifier performances were assessed (*F*_1_ score, the harmonic mean of precision and recall) using the remaining unseen 25% of the dataset. For performance quantification, we trained and assessed random forest classifiers over five independently initialized instances. For the ranking of feature contribution displayed in *z*-score-transformed heatmaps (Extended Data Figs. 4a, 6b and 8b and Supplementary Figs. 14b and 15b), we used the Gini index based on random forest classifiers trained on the full dataset as the readout. We confirmed that the out-of-bag (OOB) performances of the full dataset-trained classifiers were similar to that of the classifiers trained on 75% of the dataset (OOB_full_ − OOB_75%_ ≤ 7%). The Gini index describes the total normalized reduction of the Gini impurity by a given feature, that is, a high Gini index indicates greater feature contribution to the classifier performance.

For training of classifiers for crossregion (V1 versus insula) comparisons (Extended Data Fig. 5c,e,f and Supplementary Figs. 7, 8 and 9), random forest classifiers were trained on either untransformed or *z*-score-transformed data of the whole V1 or insula morphology dataset. Use of normalized, *z*-score-transformed datasets allows for the comparison and prediction of classes (Extended Data Fig. 5c–f) based on similarity in data distribution despite absolute differences in features, such as the total apical length or maximal branch order (Fig. 2c and Extended Data Fig. 4e)^91^.

The confusion matrices for each classifier are displayed as the cumulative allocation of cells from the withheld test sets used in the five instances of classifier training (Supplementary Fig. 7). Due to the multi-class nature of the trained classifiers, the receiver-operating characteristic curves (scikit-learn Python package) were reconstructed and the related area-under-the-curve scores calculated for each instantiated classifier (five instances) using a macro-averaged one-versus-rest scheme (Supplementary Fig. 8).

### Topographic analysis

To estimate the topographic split line based on spatial distribution of M-types (Fig. 3a,c and Extended Data Fig. 5a), we first determined individual optimal segregation lines between each M-type pair located in the same layer (based on their predominant layer assignment; Extended Data Fig. 3f) along the long axis of the insula. These segregation lines were defined as the location at which two M-types, in a pairwise comparison, were best distinguished based on the distribution of their somas along the long axis. To establish the segregation line for a pair of M-types, we first used the relative positions of median soma location to designate each M-type to either the dorsoanterior or the ventroposterior side of the long axis. Cumulative probability plots of soma locations were generated for both M-types: for the dorsoanterior-biased M-types (for example, Fig. 3a: L5b hourglass), *F*(*x*) = 1 was located at the dorsoanterior-most position and decreased toward *F*(*x*) = 0 at the ventroposterior-most position, whereas ventroposterior-biased M-types were oriented in the opposite direction (for example, Fig. 3a: L5b wide). The location along the long axis with the lowest sum of both cumulative probabilities was defined as the segregation line between this pair. A topographic split for the entire dataset was calculated using the average of all segregation lines that met the following criteria: (1) the distributions of soma locations between the two M-types were significantly shifted along the long axis (two-sided Mann–Whitney *U*-test with post hoc FDR Benjamini–Hochberg correction) and (2) the segregation line was located between the median soma locations of the two M-types.

The topographic split line was located very anteriorly relative to the DI and GI—only two mapped cells in the DI were located anterior to this split line. Therefore, only cells mapped to the AI in our dataset were allocated to either the da-AI or vp-AI, based on their relative position to the topographic split line along the long axis (Fig. 3b). Statistics on topographic distributions of these da-AI and vp-AI cells were performed. To test whether M-types had a predominant topographic localization to either da-AI or vp-AI, we compared the frequency of both allocations (da-AI/vp-AI) within each M-type to the frequencies of all other M-types residing in the same 95th percentile range of the cortical depth, using two-sided Fisher’s exact test with post hoc FDR Benjamini–Hochberg correction. The local control population consisted of all AI cells, excluding the cells allocated to the tested M-type, located within the same cortical depth range (2.5th to 97th percentile range) as the tested M-type.

The within-layer and within-region (L2 da-AI versus L2 vp-AI) finding probabilities (Fig. 3c) were calculated by dividing the frequency of specified M-type cells allocated to a given layer–region combination (for example, L2 da-AI) by the total number of cells allocated to that layer–region.

### Crossmodal feature and cell-type intersectional analysis

To identify highly correlated crossmodal features, we performed Spearman’s correlation analysis on a curated list of features. This list included all features identified during the iCCA for morphological, electrical and input cell-type analyses (Extended Data Figs. 4a, 6b and 8b) and included the number of apical stems, resting membrane potential, adaptation index and mapped locations in the insular flatmap. Note that the untransformed absolute versions of morphological and input features were used for Spearman’s correlation analysis, as opposed to the relative versions used during iCCA. Correlation analysis was performed only on combinations of features belonging to two different modalities; the *P* value was based on a permutation test with post hoc FDR Benjamini–Hochberg correction (*n* = 1911 feature combinations; Supplementary Fig. 13).

To identify characteristic features of cell types, we performed statistical feature distribution comparisons across a list of curated, pairwise, cell-type combinations (*n* = 309 combinations). The cell-type combination list was constructed based on morphological, electrical, local connectivity (input) and topographic classifications (*n* = 102 unique cell types, with *n* ≥ 3 cells per cell type). The same feature list, excluding mapped locations in the insular flatmap, was used to perform the feature distribution comparisons (two-sided Mann–Whitney *U*-test with post hoc FDR Benjamini–Hochberg correction; *n* = 6,900 tested comparisons). Only features available for the specified cell-type combination (*n* ≥ 3 cells per cell type for a given feature) were tested and displayed. For each feature comparison the log_2_(fold-change) was color coded and the bubble size was displayed based on the *P-*value thresholds, corrected for multiple testing (Supplementary Fig. 11).

Crossmodal cell-type classifications were displayed using a parallel categories plot (plotly Python package: https://plotly.com/). Vertical locations of categories were manually adjusted for display.

### Channelrhodopsin-assisted circuit mapping

Using brain slices from mice injected with virus-expressing ChR2 in the VPMpc/PF thalamus, anterior AI neurons were recorded in voltage clamp at −70 mV, whereas ChR2-expressing thalamic axons in the AI were stimulated by a 473-nm laser (Crystal Laser, cat. no. CL473-025) in two 50 μm × 50 μm-spaced stimulation grids (Fig. 8a,b). One stimulation grid was positioned in the posterior AI covering the dense expression zone of VPMpc/PF axons. The other stimulation grid was positioned in the anterior AI covering the dendrites and soma of the recorded neuron. Stimulation trials (complete grid) were repeated 3–5× and corresponding responses were averaged across the same stimulation sites. To quantify the strength of multisynaptic integration (posteriorly positioned grid) versus monosynaptic response (anteriorly positioned grid), we calculated the posterior response index (Fig. 8e), where Σ responses to posterior stimulation is the sum of all averaged responses to stimulations in the posterior AI and Σ responses to anterior stimulation is the sum of all averaged responses to stimulations in the anterior AI:

$${{\mathrm{Posterior}}}\,{{\mathrm{response}}}\,{{\mathrm{index}}}=1-\frac{\sum {{\mathrm{Responses}}}\,{{\mathrm{to}}}\,{{\mathrm{anterior}}}\,{{\mathrm{stimulation}}}}{\sum {{\mathrm{Responses}}}\,{{\mathrm{to}}}\,{{\mathrm{posterior}}}\,{{\mathrm{stimulation}}}}.$$

### Conditioned taste aversion

Wild-type C57BL/6 mice received stereotaxic injections of virus-expressing tetanus toxin (AAV5-CAG-FLEX-TeLC-GFP; seven male and five female) or GFP control (AAV5-CAG-FLEX-GFP; five male and four female) at P29–P36 as described above and recovered for 2 weeks in their home cages. Mice were then single housed in customized experimental cages and adapted to a reverse 12:12 dark-to-light cycle (lights off at 10:30 am) for 5 d with water and food provided freely. After habituation to the reverse light cycle, mice were water restricted for the remainder of the behavioral schedule during which they were given access to liquid from a graduated drinking tube only during two daily sessions: for 30 min at the beginning of their dark cycle in the morning session (henceforth, the ‘30-min session’) and 60 min in the afternoon (where water was always presented, 4 h after the end of the morning session). During water restriction training, mice were trained to drink >0.8 ml of water during the 30-min session for 2 d consecutively before proceeding to conditioning. Over 2 d of conditioning, mice were presented with sucrose (0.15 M in tap water) during the 30-min session, followed immediately by an intra-peritoneal LiCl injection (0.15 M, 2% ml per g body weight). In rare cases where mice drank <1 ml of sucrose on initial exposure to sucrose, the LiCl pairing was delayed until sucrose consumption exceeded 1 ml. After conditioning, CTA was assessed daily during test sessions over 4 d consecutively, where mice had free access to both water and sucrose during the ‘30-min session’. Bottle position was alternated daily throughout the behavioral schedule and all behavioral experiments were performed during the dark cycle. Experimenters remained blind to the treatment group throughout the behavior schedule. Mice were included in the cohort based on two criteria: (1) post hoc histological confirmation that stereotaxic injection sites were located in the target regions (10 of 12 mice in the tetanus toxin treatment group) and (2) an ability to adapt to the water deprivation paradigm (all mice in tetanus toxin treatment group, 9 of 11 mice in the control group).

### Statistics and reproducibility

No statistical methods were used to predetermine sample size; the group sizes used in each experiment are comparable to those generally employed in the field. Multiple cells were obtained per mouse (median: 3 cells per mouse, range: 1–13, Supplementary Table 5) and sample conditions were randomized for experiments. Data collection and analysis, with the exception of conducting CTA, were not performed blind to the conditions of the experiments. Criteria for excluding observations from analysis are listed in appropriate sections (Methods). Unless otherwise stated, the mean ± s.e.m. was used to report statistics. The statistical tests used, definition of *n* and multiple-comparison correction where appropriate are described in the figure legends and Methods. For pairwise comparison in Fig. 2c, a Kruskal–Wallis test with post hoc two-sided Dunn’s test and FDR Benjamini–Hochberg correction was used. Representative experiments in Fig. 1b and Extended Data Fig. 2a,b,h,i were independently repeated at least 3×. All boxplots use the following conventions: center line or dot for the median; box limits for upper and lower quartiles; whiskers for 1.5× the IQR; and diamonds for outliers. All statistical tests were two sided with significance defined as *α* = 0.05 unless noted otherwise. Statistical analyses were performed in R (v4.2), MATLAB (v2020a and v2022a) or Python (v3.8 and v3.9).

### Reporting summary

Further information on research design is available in the Nature Portfolio Reporting Summary linked to this article.

## Online content

Any methods, additional references, Nature Portfolio reporting summaries, source data, extended data, supplementary information, acknowledgements, peer review information; details of author contributions and competing interests; and statements of data and code availability are available at https://doi.org/10.1038/s41593-026-02391-0.

## Supplementary information

Supplementary Information Supplementary Notes, Figs. 1–15 and references.

Reporting Summary

Supplementary Tables Supplementary Tables 1–17, each on a separate worksheet. Supplementary Table 1: Overview of morphology, electrophysiology, and input properties/features. Supplementary Table 2: iCCA parameters and performance across analyzed modalities. Supplementary Table 3: List of morphology features used for final M-type classification. Supplementary Table 4: Mouse strains included in this database. Supplementary Tables 5–17: Number of observations and exact *P* values.

## Data Availability

All raw data supporting this study are deposited in format-appropriate repositories (BrainImageLibrary.org for Nissl images, NeuroMorpho.org^90^ for morphological reconstructions) and/or are available via Zenodo at https://doi.org/10.5281/zenodo.20714121 (ref. ^92^). Any additional information required to re-analyze the data reported in this paper is available from the corresponding authors upon request.

## Code availability

All customized analysis code is available via GitLab at https://gitlab.com/maolab/braincellsuite_submission and https://gitlab.com/maolab/mouseinsularatlas.
