# Supplementary material for: Pyramidal cell types and circuit organization of the mouse insular cortex reveal functional specializations
Source: Nat Neurosci. 2026 Aug 7;29(10):2599–612. doi: 10.1038/s41593-026-02391-0 (PMC13633118; doi:10.1038/s41593-026-02391-0)
Supplement: Supplementary file 1 [file 41593_2026_2391_MOESM1_ESM.pdf]

nature neuroscience

Supplementary information

https://doi.org/10.1038/s41593-026-02391-0

Pyramidal cell types and circuit organization of the mouse insular cortex reveal functional specializations

In the format provided by the authors and unedited

Supplementary Information

Supplementary Notes

Supplementary Fig. 1: Complete sets of consensus manual annotations of the insular cortex.

Supplementary Fig. 2: Quantification of insular cortex layer thickness.

Supplementary Fig. 3: Mean layer thickness across the insular cortex.

Supplementary Fig. 4: Overview of 958 reconstructed dendritic morphologies.

Supplementary Fig. 5: Comparison of M-type allocations between two iCCA approaches.

Supplementary Fig. 6: Distinct features of M-types.

Supplementary Fig. 7: Confusion matrices of trained random forest classifiers.

Supplementary Fig. 8: Receiver-operating characteristic (ROC) and total area under the curve (AUC) performances of trained forest classifiers.

Supplementary Fig. 9: No insular L5b M-type equivalents were found in V1.

Supplementary Fig. 10: Distinct features of E-profiles.

Supplementary Fig. 11: Informative features for pair-wise comparisons between cell types across all modalities.

Supplementary Fig. 12: Informative features for cell type identification.

Supplementary Fig. 13: Cross-modal feature correlations.

Supplementary Fig. 14: Analysis of combined morpho-electrical features and subsequent cell groups.

Supplementary Fig. 15: Analysis of combined morpho-input features and subsequent cell groups.

Supplementary Notes

Cross-modal associations between cell types and potential molecular contributors

Despite heterogeneous cross-modal associations between M-types and E- or I-profiles, groups of M-types occupied confined regions in the electrical and input feature spaces. For example, all L5b morphologies displayed large voltage sags with either burst or regular spiking behaviors (Fig. 6b). Such cross-modal associations were rooted in specific biological parameters: the more elaborate the distal dendritic arbor, the larger the voltage sags (Fig. 6d). This points to the functional significance of cellular structures, because distal dendrites preferentially host HCN channels,[53] and the regulation of HCN channel density is an important factor for synaptic integration at the dendritic level.[93] Further, such physiological properties can be dynamic and modulated by neuromodulators (e.g., dopamine, acetylcholine).[60,94] Additionally, cell type-specific modulation of input strength may fine-tune local input distributions.[60,94] Therefore, E- and I-profiles obtained from 'naïve' mice in the present work may reflect baseline electrical and input states from which cells can transition in response to neuromodulation.

IC subregions defined by topographical distributions of M-types

Our dorsoanterior-AI coordinates generally corroborate with the empirically defined anterior IC,[10,13] whereas our ventroposterior-AI coordinates are anteriorly-shifted relative to the empirical posterior IC.[11] This might be due to the technical constraints for empirically determined IC subregion—often from in vivo studies—in separating the AI/DI/GI, because the AI's center of mass is more anterior compared to the entire IC.

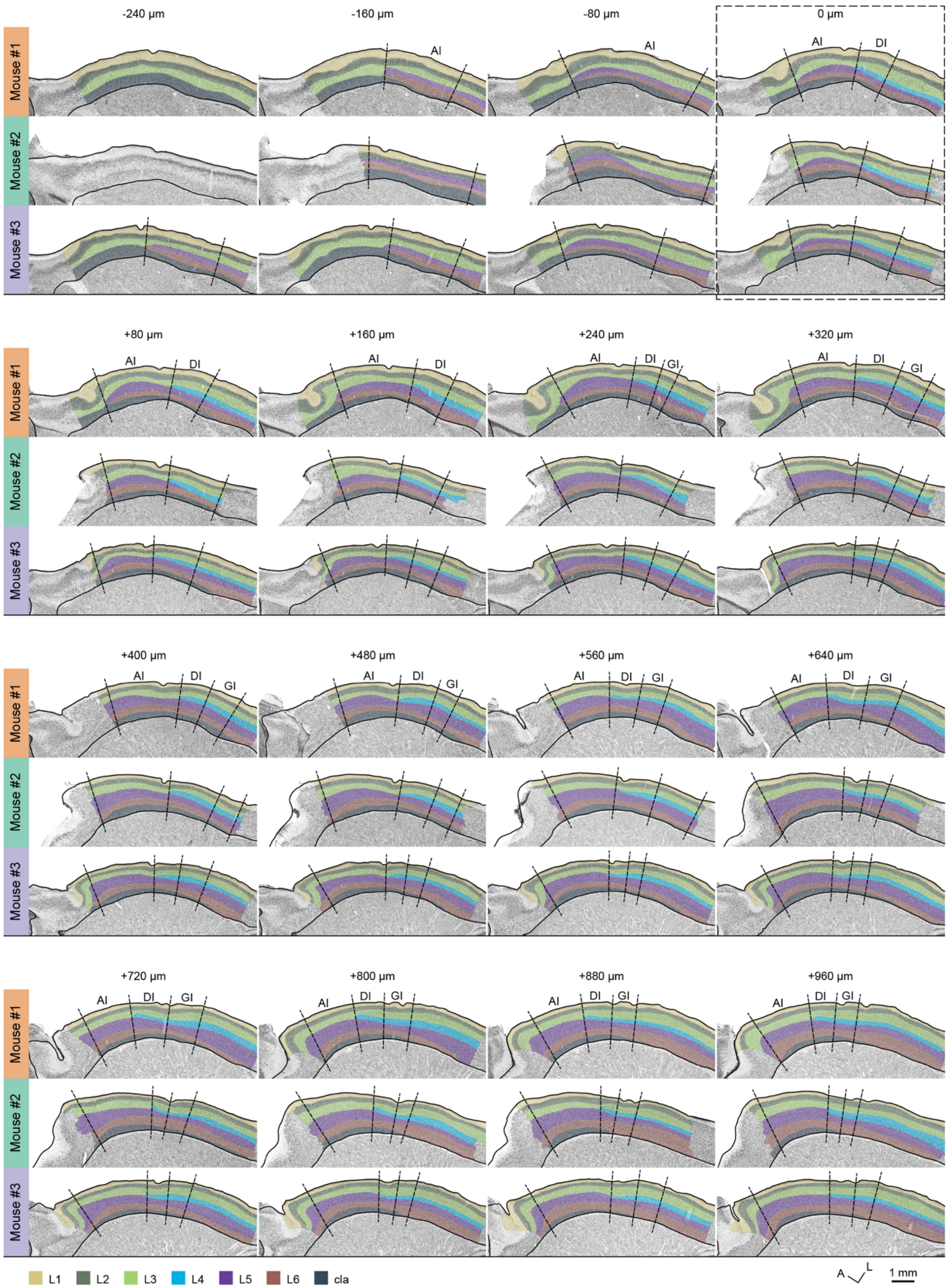

-240 µm
-160 µm
-80 µm
0 µm
+80 µm
+160 µm
+240 µm
+320 µm
+400 µm
+480 µm
+560 µm
+640 µm
+720 µm
+800 µm
+880 µm
+960 µm
Mouse #1
Mouse #2
Mouse #3
AI
DI
GI
L1
L2
L3
L4
L5
L6
cla
A
L
1 mm

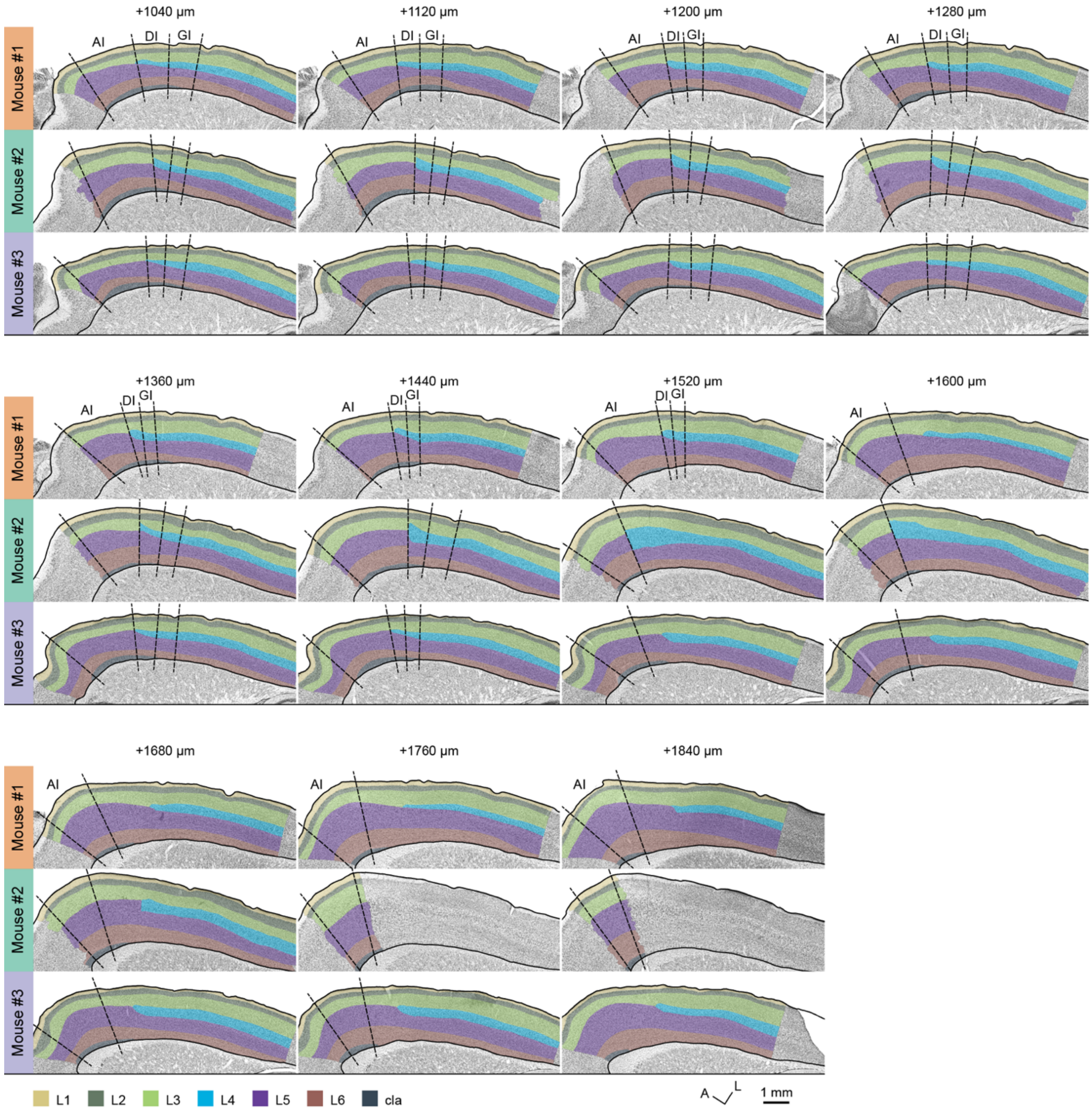

Supplementary Fig. 1: Complete sets of consensus manual annotations of the insular cortex. An alternating (every other) series of 40 µm-thick horizontal sections collected from three *PV-Cre*$^{+/-}$*;Ai9*$^{+/-}$ mice processed for Nissl histology. Overlay shows the average consensus from two independent expert annotators demarcating the locations of all cortical layers within and adjacent to the insular cortex, as well as the boundaries of its subregions. Sections from each animal are arranged in rows along the ventral-to-dorsal cutting axis (top-left to bottom-right) and aligned across animals based on the ventral-most section in which

layer 4 (L4) is conspicuous (dashed box, top row, 4th column). The position of each section relative to this L4 anchor along the ventral-to-dorsal cutting axis is indicated above each column in µm. A, anterior; L, lateral; AI, agranular insula; DI, dysgranular insula; GI, granular insula; cla, claustrum; L1-L6, cortical layers 1-6.

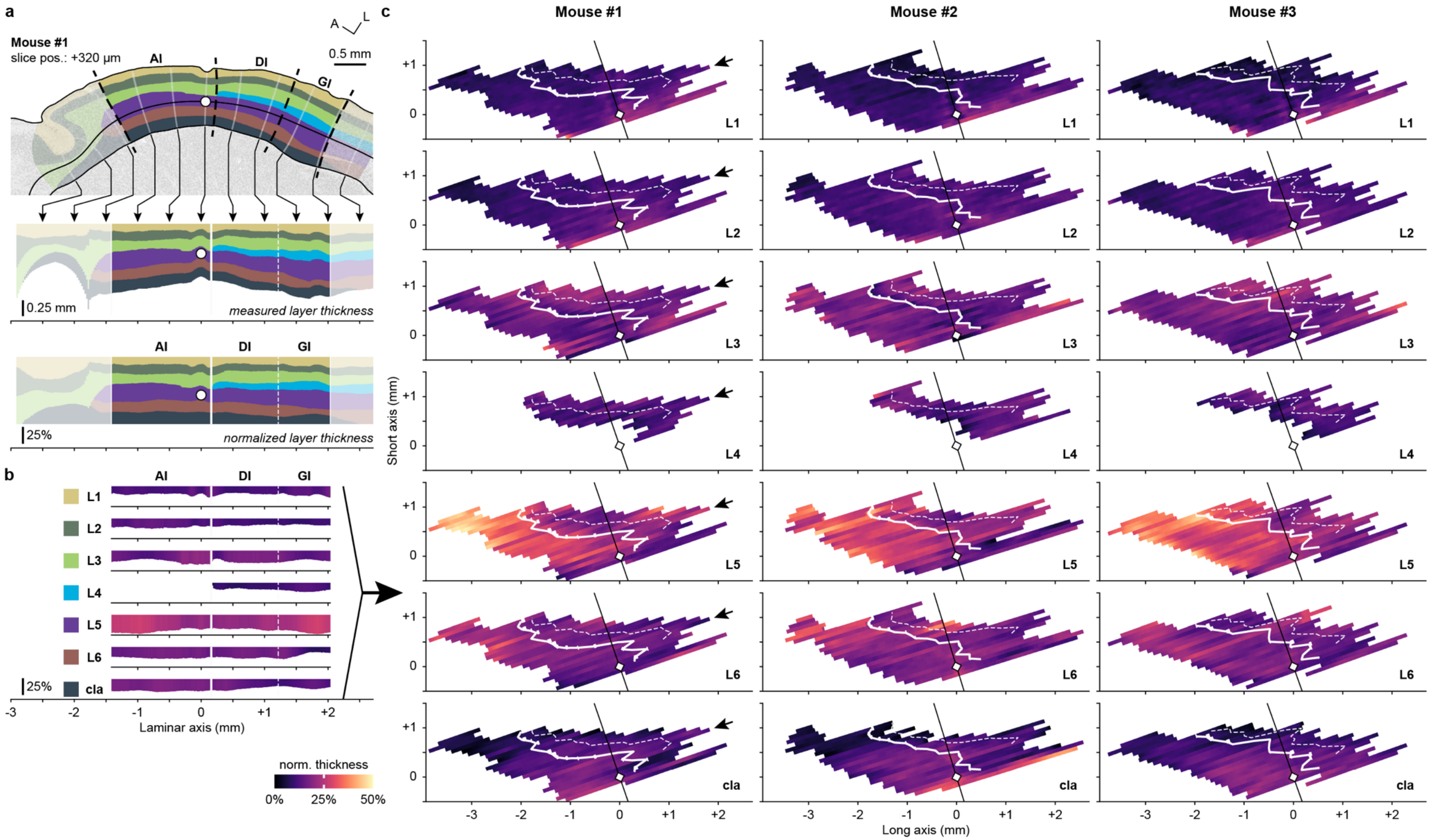

Supplementary Fig. 2: See next page for caption.

Supplementary Fig. 2: Quantification of insular cortex layer thickness. a, Example process of defining layer boundaries and calculating relative cortical depth. The layers were first manually annotated (top; Fig. 1c and Supplementary Fig. 1). The section was then 'flattened' according to streamlines (middle; Fig. 1d) and normalized by cortical thickness (bottom; Extended Data Figs. 1c,e). Arrows (displayed at 0.5 mm intervals) show the relative mapping of 25-µm wide streamline bins from the physical section (top) to a 'flattened' laminar axis plot (middle/bottom). White circles: MCA-derived coordinate origin. **b**, Visual representations of the change in normalized thickness for each layer from (**a**) along the laminar axis. Luminance indicates normalized cortical thickness. **c**, 2D flatmaps of relative layer thickness for each reference mouse, rotated as in Extended Data Fig. 1f. White diamond: MCA origin; black line: best-fit plane to MCA; thick white line: AI/DI border; thin dashed white line: DI/GI border; arrowheads: row in Mouse #1 corresponding to data shown in (**a** and **b**). A, anterior; L, lateral; AI, agranular insula; DI, dysgranular insula; GI, granular insula; MCA, middle cerebral artery; cla, claustrum.

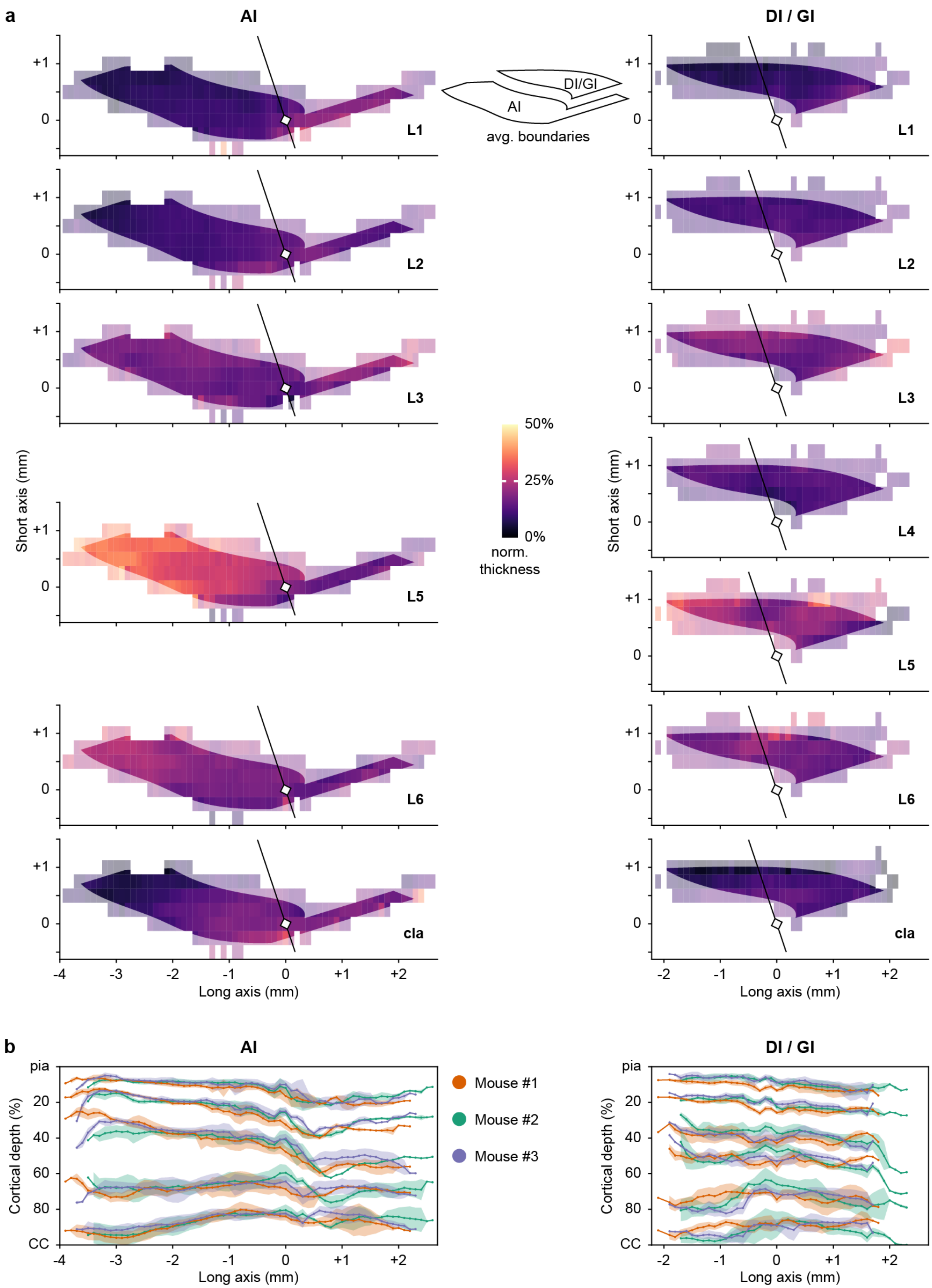

Supplementary Fig. 3: See next page for caption.

Supplementary Fig. 3: Mean layer thickness across the insular cortex. a, 2D flatmaps showing mean normalized layer thickness for AI (left) and DI/GI (right), respectively. Values from individual flatmaps (Supplementary Fig. 2c) were binned at 100-µm and 250-µm intervals along the long and short axes, respectively, relative to the MCA origin (white diamond). Luminance indicates normalized cortical thickness. Shaded areas: the average boundary of each subregion (Extended Data Fig. 1g); black line: best-fit plane to MCA. **b**, Mean normalized position of layer boundaries (Supplementary Fig. 2) binned along the flatmap long axis separately for AI (left) and DI/GI (right) for each mouse. Shaded regions: standard deviation. AI, agranular insula; DI, dysgranular insula; GI, granular insula; MCA, middle cerebral artery; cla, claustrum; CC, corpus callosum.

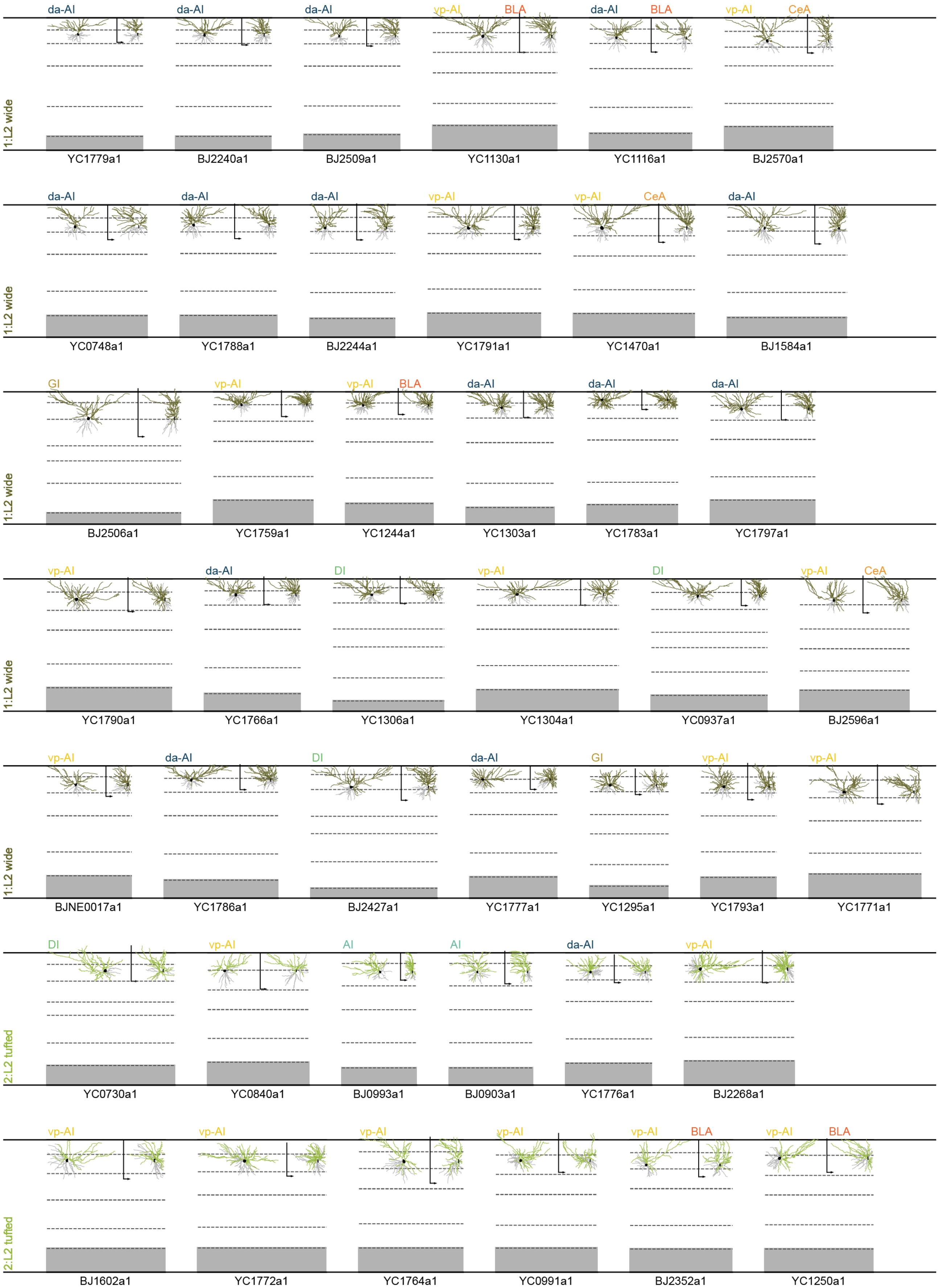

da-AI
da-AI
da-AI
vp-AI
BLA
da-AI
BLA
vp-AI
CeA
1:L2 wide
YC1779a1
BJ2240a1
BJ2509a1
YC1130a1
YC1116a1
BJ2570a1
da-AI
da-AI
da-AI
vp-AI
vp-AI
CeA
da-AI
1:L2 wide
YC0748a1
YC1788a1
BJ2244a1
YC1791a1
YC1470a1
BJ1584a1
GI
vp-AI
vp-AI
BLA
da-AI
da-AI
da-AI
1:L2 wide
BJ2506a1
YC1759a1
YC1244a1
YC1303a1
YC1783a1
YC1797a1
vp-AI
da-AI
DI
vp-AI
DI
vp-AI
CeA
1:L2 wide
YC1790a1
YC1766a1
YC1306a1
YC1304a1
YC0937a1
BJ2596a1
vp-AI
da-AI
DI
da-AI
GI
vp-AI
vp-AI
1:L2 wide
BJNE0017a1
YC1786a1
BJ2427a1
YC1777a1
YC1295a1
YC1793a1
YC1771a1
DI
vp-AI
AI
AI
da-AI
vp-AI
2:L2 tufted
YC0730a1
YC0840a1
BJ0993a1
BJ0903a1
YC1776a1
BJ2268a1
vp-AI
vp-AI
vp-AI
vp-AI
vp-AI
BLA
vp-AI
BLA
2:L2 tufted
BJ1602a1
YC1772a1
YC1764a1
YC0991a1
BJ2352a1
YC1250a1

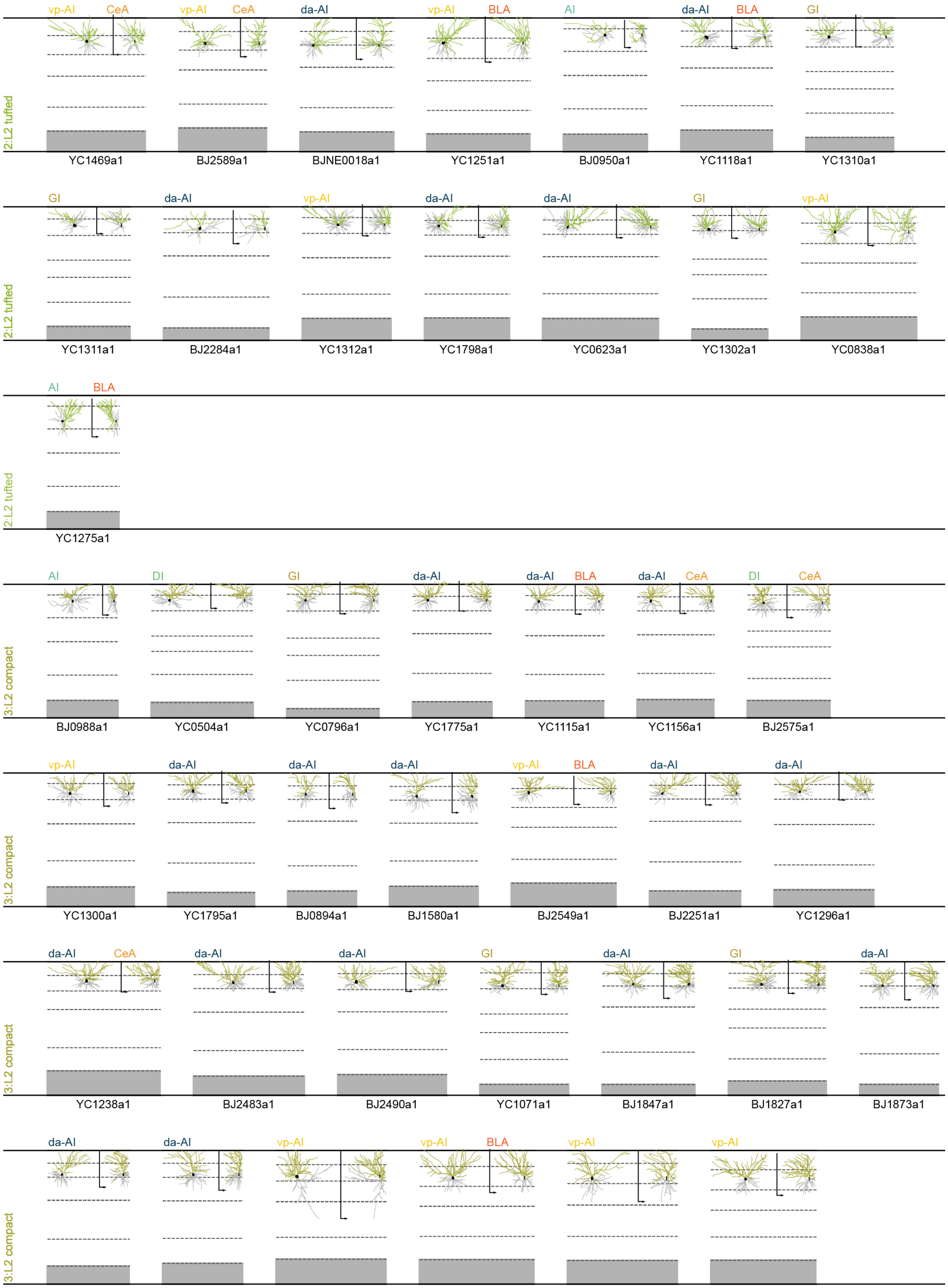
vp-AI
CeA
vp-AI
CeA
da-AI
vp-AI
BLA
AI
da-AI
BLA
GI
2:L2 tufted
YC1469a1
BJ2589a1
BJNE0018a1
YC1251a1
BJ0950a1
YC1118a1
YC1310a1
GI
da-AI
vp-AI
da-AI
da-AI
GI
vp-AI
2:L2 tufted
YC1311a1
BJ2284a1
YC1312a1
YC1798a1
YC0623a1
YC1302a1
YC0838a1
AI
BLA
2:L2 tufted
YC1275a1
AI
DI
GI
da-AI
da-AI
BLA
da-AI
CeA
DI
CeA
3:L2 compact
BJ0988a1
YC0504a1
YC0796a1
YC1775a1
YC1115a1
YC1156a1
BJ2575a1
vp-AI
da-AI
da-AI
da-AI
vp-AI
BLA
da-AI
da-AI
3:L2 compact
YC1300a1
YC1795a1
BJ0894a1
BJ1580a1
BJ2549a1
BJ2251a1
YC1296a1
da-AI
CeA
da-AI
da-AI
GI
da-AI
GI
da-AI
3:L2 compact
YC1238a1
BJ2483a1
BJ2490a1
YC1071a1
BJ1847a1
BJ1827a1
BJ1873a1
da-AI
da-AI
vp-AI
vp-AI
BLA
vp-AI
vp-AI
3:L2 compact
BJ2232a1
YC1762a1
YC1784a1
BJ2572a1
BJ2495a1
YC0831a1

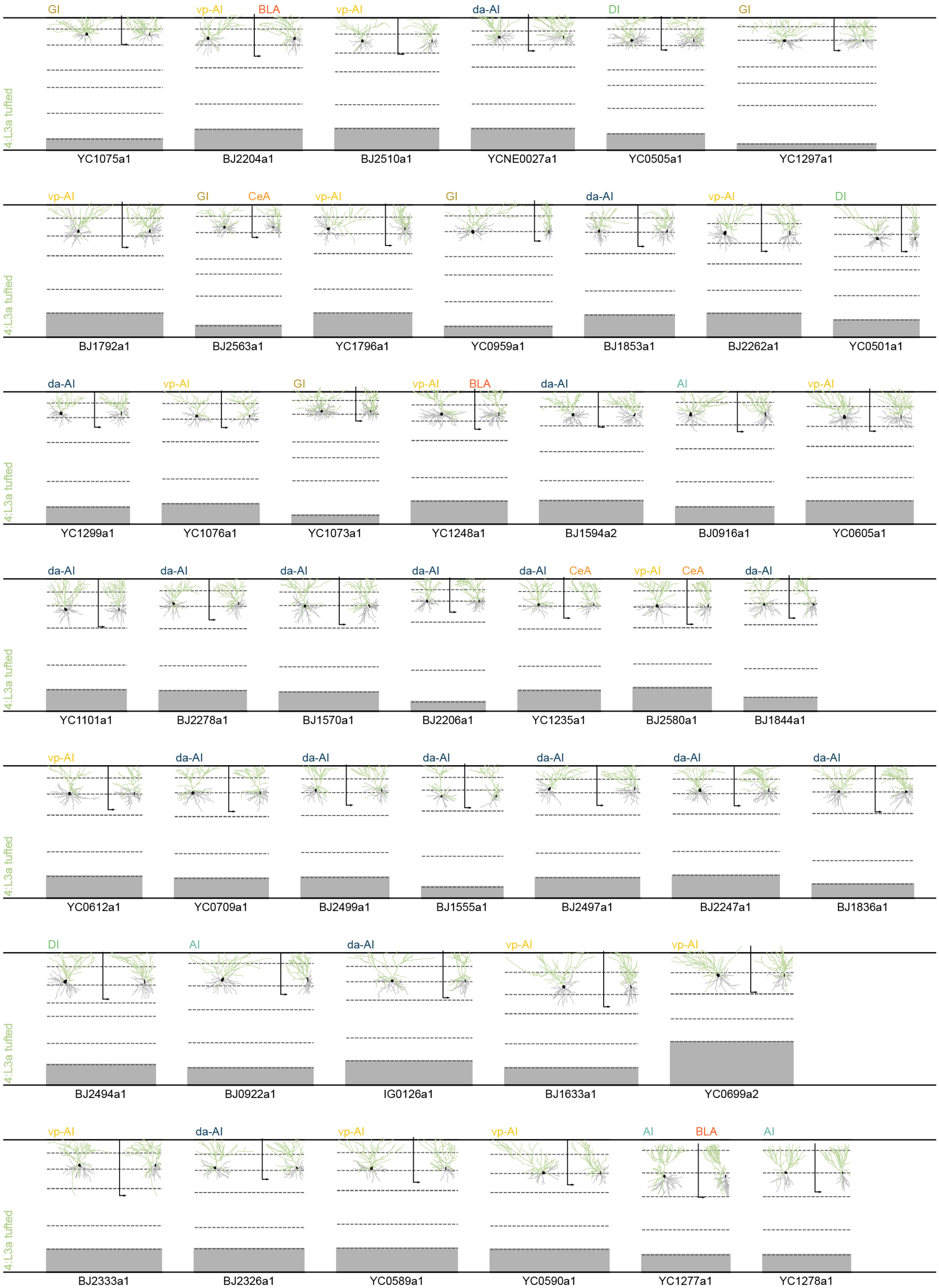
GI
vp-AI
BLA
vp-AI
da-AI
DI
GI
4:L3a tufted
YC1075a1
BJ2204a1
BJ2510a1
YCNE0027a1
YC0505a1
YC1297a1
vp-AI
GI
CeA
vp-AI
GI
da-AI
vp-AI
DI
4:L3a tufted
BJ1792a1
BJ2563a1
YC1796a1
YC0959a1
BJ1853a1
BJ2262a1
YC0501a1
da-AI
vp-AI
GI
vp-AI
BLA
da-AI
AI
vp-AI
4:L3a tufted
YC1299a1
YC1076a1
YC1073a1
YC1248a1
BJ1594a2
BJ0916a1
YC0605a1
da-AI
da-AI
da-AI
da-AI
da-AI
CeA
vp-AI
CeA
da-AI
4:L3a tufted
YC1101a1
BJ2278a1
BJ1570a1
BJ2206a1
YC1235a1
BJ2580a1
BJ1844a1
vp-AI
da-AI
da-AI
da-AI
da-AI
da-AI
da-AI
4:L3a tufted
YC0612a1
YC0709a1
BJ2499a1
BJ1555a1
BJ2497a1
BJ2247a1
BJ1836a1
DI
AI
da-AI
vp-AI
vp-AI
4:L3a tufted
BJ2494a1
BJ0922a1
IG0126a1
BJ1633a1
YC0699a2
vp-AI
da-AI
vp-AI
vp-AI
AI
BLA
AI
4:L3a tufted
BJ2333a1
BJ2326a1
YC0589a1
YC0590a1
YC1277a1
YC1278a1

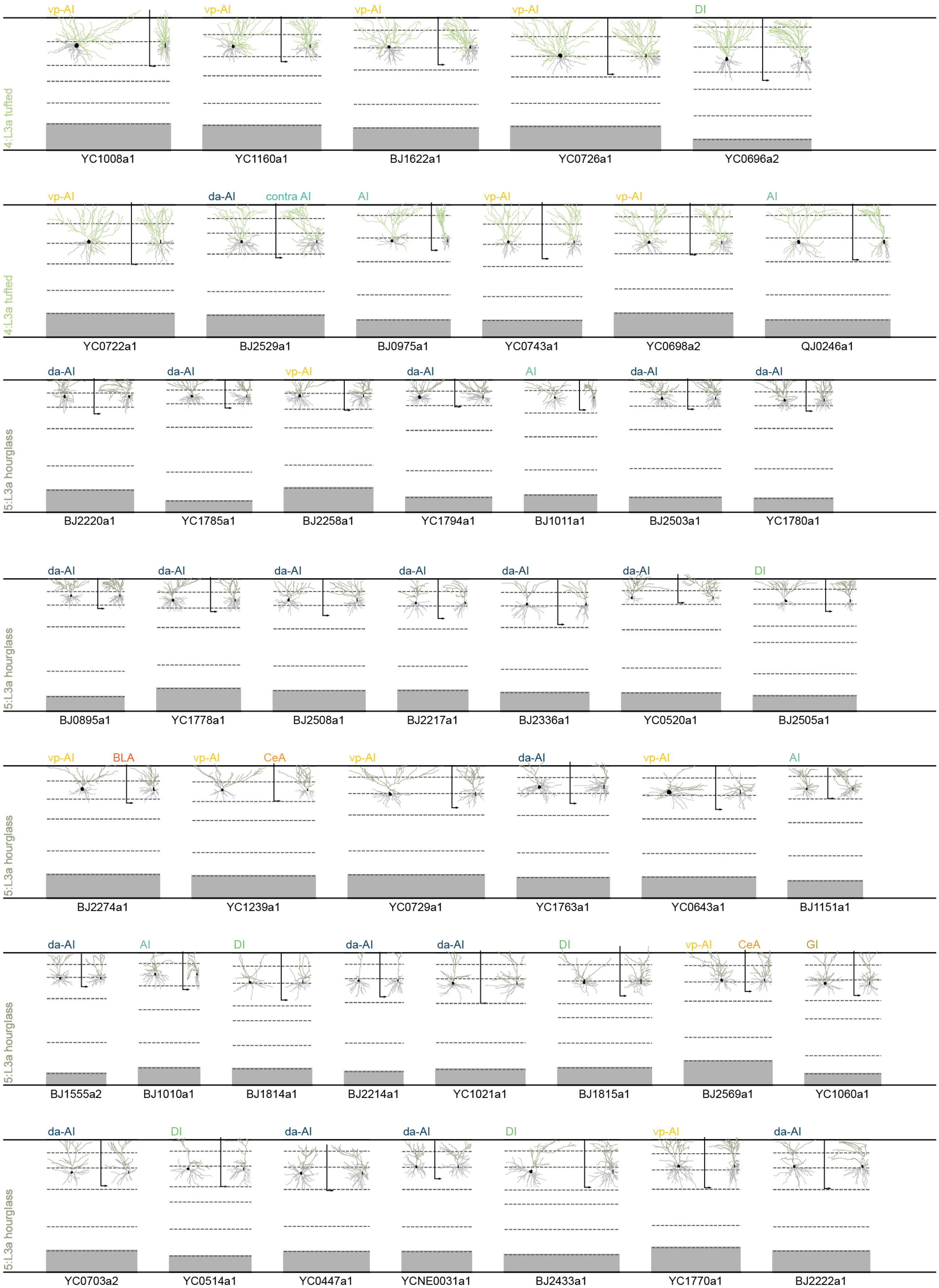

4:L3a tufted
vp-AI
YC1008a1
vp-AI
YC1160a1
vp-AI
BJ1622a1
vp-AI
YC0726a1
DI
YC0696a2
4:L3a tufted
vp-AI
YC0722a1
da-AI
contra AI
BJ2529a1
AI
BJ0975a1
vp-AI
YC0743a1
vp-AI
YC0698a2
AI
QJ0246a1
5:L3a hourglass
da-AI
BJ2220a1
da-AI
YC1785a1
vp-AI
BJ2258a1
da-AI
YC1794a1
AI
BJ1011a1
da-AI
BJ2503a1
da-AI
YC1780a1
5:L3a hourglass
da-AI
BJ0895a1
da-AI
YC1778a1
da-AI
BJ2508a1
da-AI
BJ2217a1
da-AI
BJ2336a1
da-AI
YC0520a1
DI
BJ2505a1
5:L3a hourglass
vp-AI
BLA
BJ2274a1
vp-AI
CeA
YC1239a1
vp-AI
YC0729a1
da-AI
YC1763a1
vp-AI
YC0643a1
AI
BJ1151a1
5:L3a hourglass
da-AI
BJ1555a2
AI
BJ1010a1
DI
BJ1814a1
da-AI
BJ2214a1
da-AI
YC1021a1
DI
BJ1815a1
vp-AI
CeA
BJ2569a1
GI
YC1060a1
5:L3a hourglass
da-AI
YC0703a2
DI
YC0514a1
da-AI
YC0447a1
da-AI
YCNE0031a1
DI
BJ2433a1
vp-AI
YC1770a1
da-AI
BJ2222a1

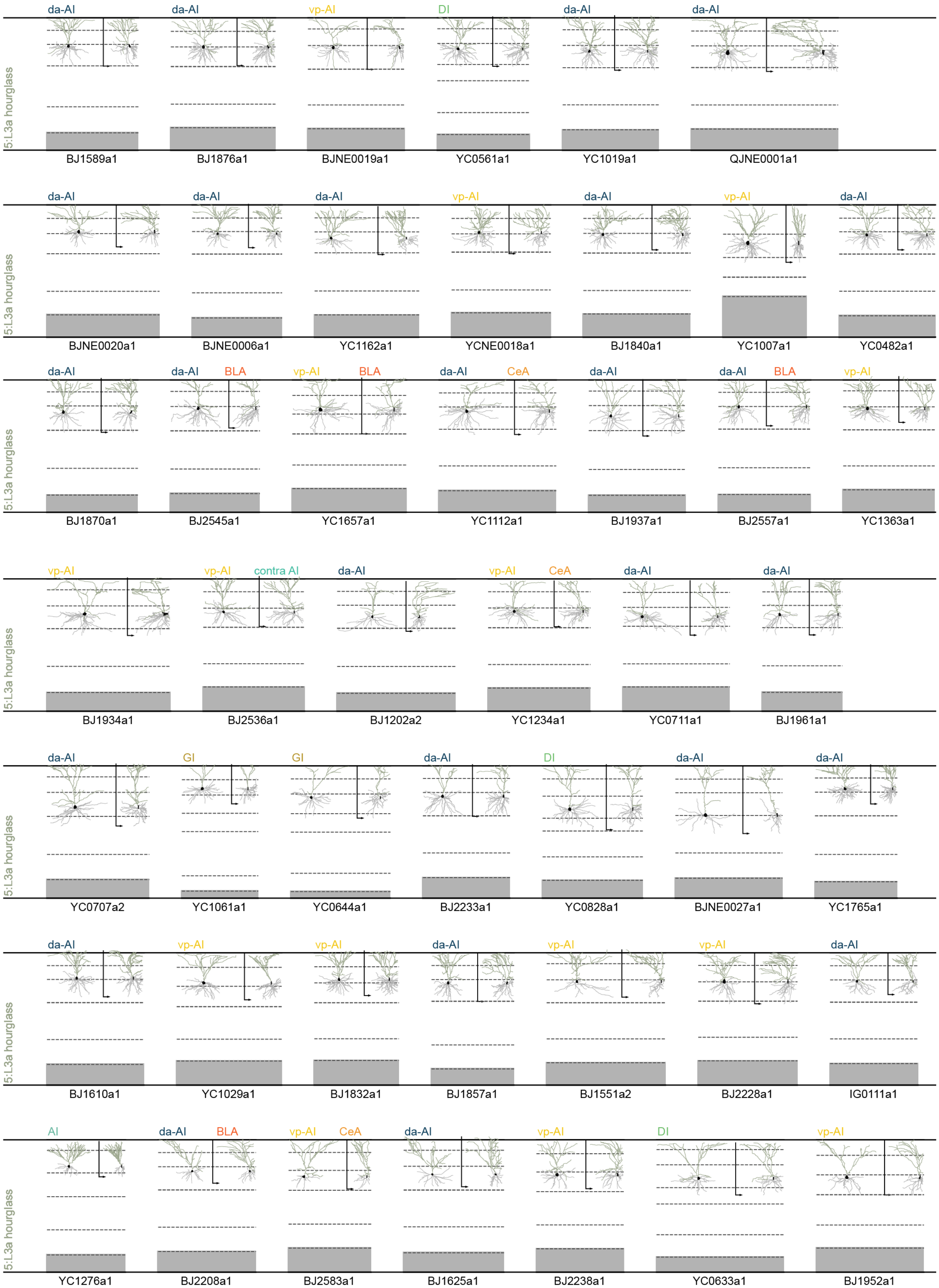
5:L3a hourglass
da-AI
da-AI
vp-AI
DI
da-AI
da-AI
BJ1589a1
BJ1876a1
BJNE0019a1
YC0561a1
YC1019a1
QJNE0001a1
5:L3a hourglass
da-AI
da-AI
da-AI
vp-AI
da-AI
vp-AI
da-AI
BJNE0020a1
BJNE0006a1
YC1162a1
YCNE0018a1
BJ1840a1
YC1007a1
YC0482a1
5:L3a hourglass
da-AI
da-AI BLA
vp-AI BLA
da-AI CeA
da-AI
da-AI BLA
vp-AI
BJ1870a1
BJ2545a1
YC1657a1
YC1112a1
BJ1937a1
BJ2557a1
YC1363a1
5:L3a hourglass
vp-AI
vp-AI contra AI
da-AI
vp-AI CeA
da-AI
da-AI
BJ1934a1
BJ2536a1
BJ1202a2
YC1234a1
YC0711a1
BJ1961a1
5:L3a hourglass
da-AI
GI
GI
da-AI
DI
da-AI
da-AI
YC0707a2
YC1061a1
YC0644a1
BJ2233a1
YC0828a1
BJNE0027a1
YC1765a1
5:L3a hourglass
da-AI
vp-AI
vp-AI
da-AI
vp-AI
vp-AI
da-AI
BJ1610a1
YC1029a1
BJ1832a1
BJ1857a1
BJ1551a2
BJ2228a1
IG0111a1
5:L3a hourglass
AI
da-AI BLA
vp-AI CeA
da-AI
vp-AI
DI
vp-AI
YC1276a1
BJ2208a1
BJ2583a1
BJ1625a1
BJ2238a1
YC0633a1
BJ1952a1

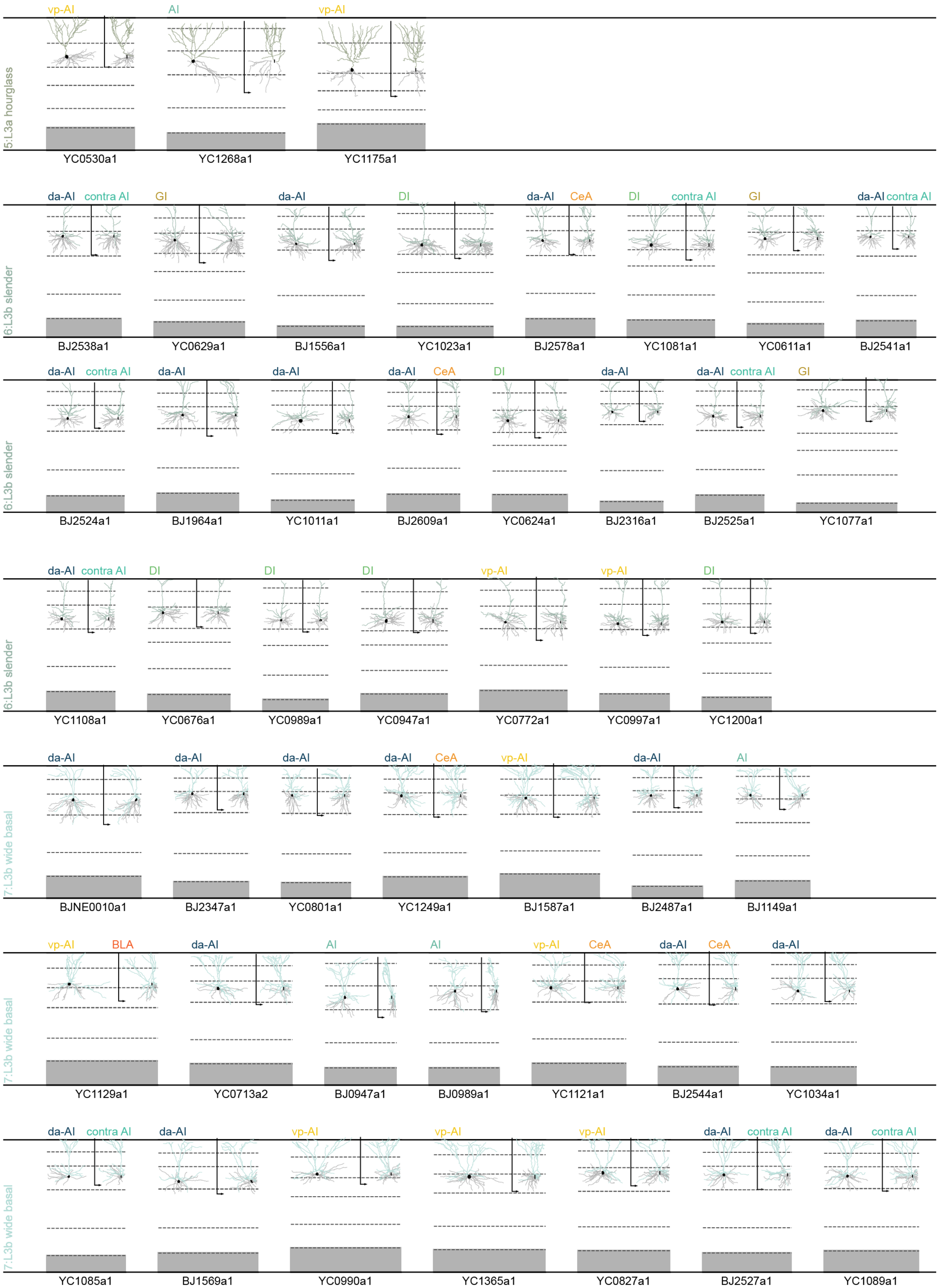
5:L3a hourglass
vp-AI
AI
vp-AI
YC0530a1
YC1268a1
YC1175a1
6:L3b slender
da-AI contra AI
GI
da-AI
DI
da-AI CeA
DI contra AI
GI
da-AI contra AI
BJ2538a1
YC0629a1
BJ1556a1
YC1023a1
BJ2578a1
YC1081a1
YC0611a1
BJ2541a1
6:L3b slender
da-AI contra AI
da-AI
da-AI
da-AI CeA
DI
da-AI
da-AI contra AI
GI
BJ2524a1
BJ1964a1
YC1011a1
BJ2609a1
YC0624a1
BJ2316a1
BJ2525a1
YC1077a1
6:L3b slender
da-AI contra AI
DI
DI
DI
vp-AI
vp-AI
DI
YC1108a1
YC0676a1
YC0989a1
YC0947a1
YC0772a1
YC0997a1
YC1200a1
7:L3b wide basal
da-AI
da-AI
da-AI
da-AI CeA
vp-AI
da-AI
AI
BJNE0010a1
BJ2347a1
YC0801a1
YC1249a1
BJ1587a1
BJ2487a1
BJ1149a1
7:L3b wide basal
vp-AI BLA
da-AI
AI
AI
vp-AI CeA
da-AI CeA
da-AI
YC1129a1
YC0713a2
BJ0947a1
BJ0989a1
YC1121a1
BJ2544a1
YC1034a1
7:L3b wide basal
da-AI contra AI
da-AI
vp-AI
vp-AI
vp-AI
da-AI contra AI
da-AI contra AI
YC1085a1
BJ1569a1
YC0990a1
YC1365a1
YC0827a1
BJ2527a1
YC1089a1

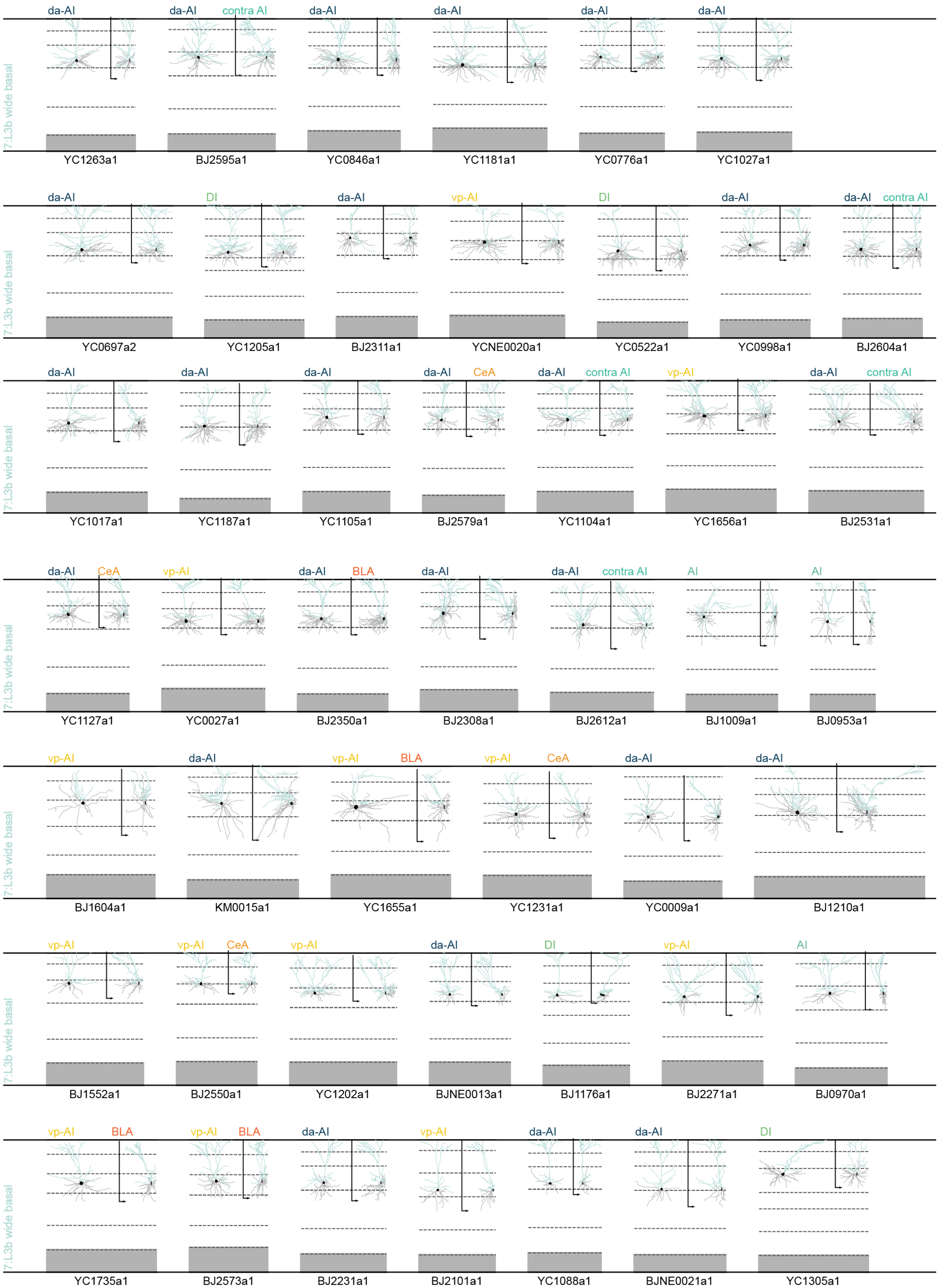
7:L3b wide basal
da-AI
contra AI
YC1263a1
BJ2595a1
YC0846a1
YC1181a1
YC0776a1
YC1027a1
DI
vp-AI
YC0697a2
YC1205a1
BJ2311a1
YCNE0020a1
YC0522a1
YC0998a1
BJ2604a1
CeA
YC1017a1
YC1187a1
YC1105a1
BJ2579a1
YC1104a1
YC1656a1
BJ2531a1
BLA
AI
YC1127a1
YC0027a1
BJ2350a1
BJ2308a1
BJ2612a1
BJ1009a1
BJ0953a1
BJ1604a1
KM0015a1
YC1655a1
YC1231a1
YC0009a1
BJ1210a1
BJ1552a1
BJ2550a1
YC1202a1
BJNE0013a1
BJ1176a1
BJ2271a1
BJ0970a1
YC1735a1
BJ2573a1
BJ2231a1
BJ2101a1
YC1088a1
BJNE0021a1
YC1305a1

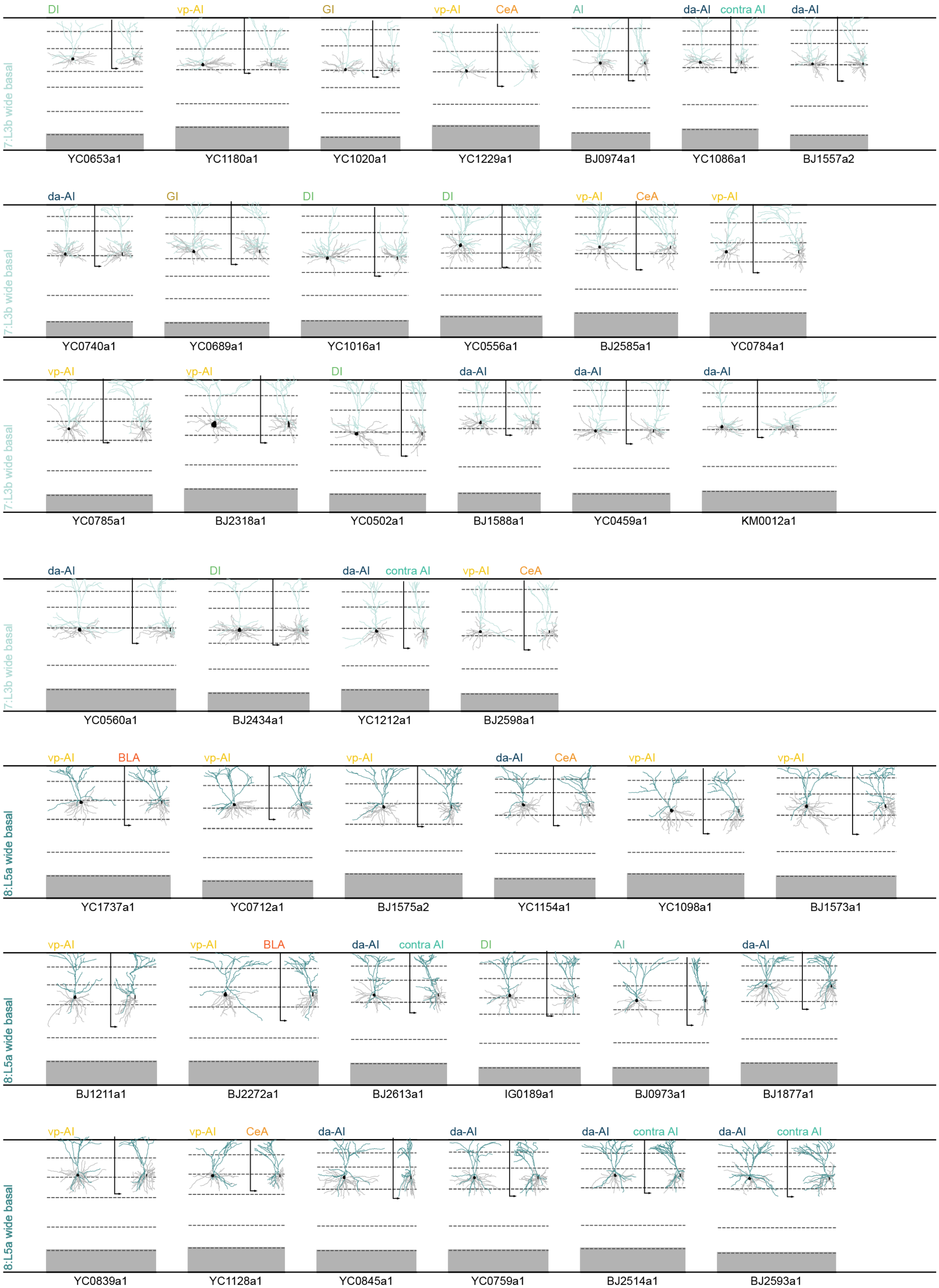

7:L3b wide basal
DI
vp-AI
GI
vp-AI
CeA
AI
da-AI
contra AI
da-AI
YC0653a1
YC1180a1
YC1020a1
YC1229a1
BJ0974a1
YC1086a1
BJ1557a2
7:L3b wide basal
da-AI
GI
DI
DI
vp-AI
CeA
vp-AI
YC0740a1
YC0689a1
YC1016a1
YC0556a1
BJ2585a1
YC0784a1
7:L3b wide basal
vp-AI
vp-AI
DI
da-AI
da-AI
da-AI
YC0785a1
BJ2318a1
YC0502a1
BJ1588a1
YC0459a1
KM0012a1
7:L3b wide basal
da-AI
DI
da-AI
contra AI
vp-AI
CeA
YC0560a1
BJ2434a1
YC1212a1
BJ2598a1
8:L5a wide basal
vp-AI
BLA
vp-AI
vp-AI
da-AI
CeA
vp-AI
vp-AI
YC1737a1
YC0712a1
BJ1575a2
YC1154a1
YC1098a1
BJ1573a1
8:L5a wide basal
vp-AI
vp-AI
BLA
da-AI
contra AI
DI
AI
da-AI
BJ1211a1
BJ2272a1
BJ2613a1
IG0189a1
BJ0973a1
BJ1877a1
8:L5a wide basal
vp-AI
vp-AI
CeA
da-AI
da-AI
da-AI
contra AI
da-AI
contra AI
YC0839a1
YC1128a1
YC0845a1
YC0759a1
BJ2514a1
BJ2593a1

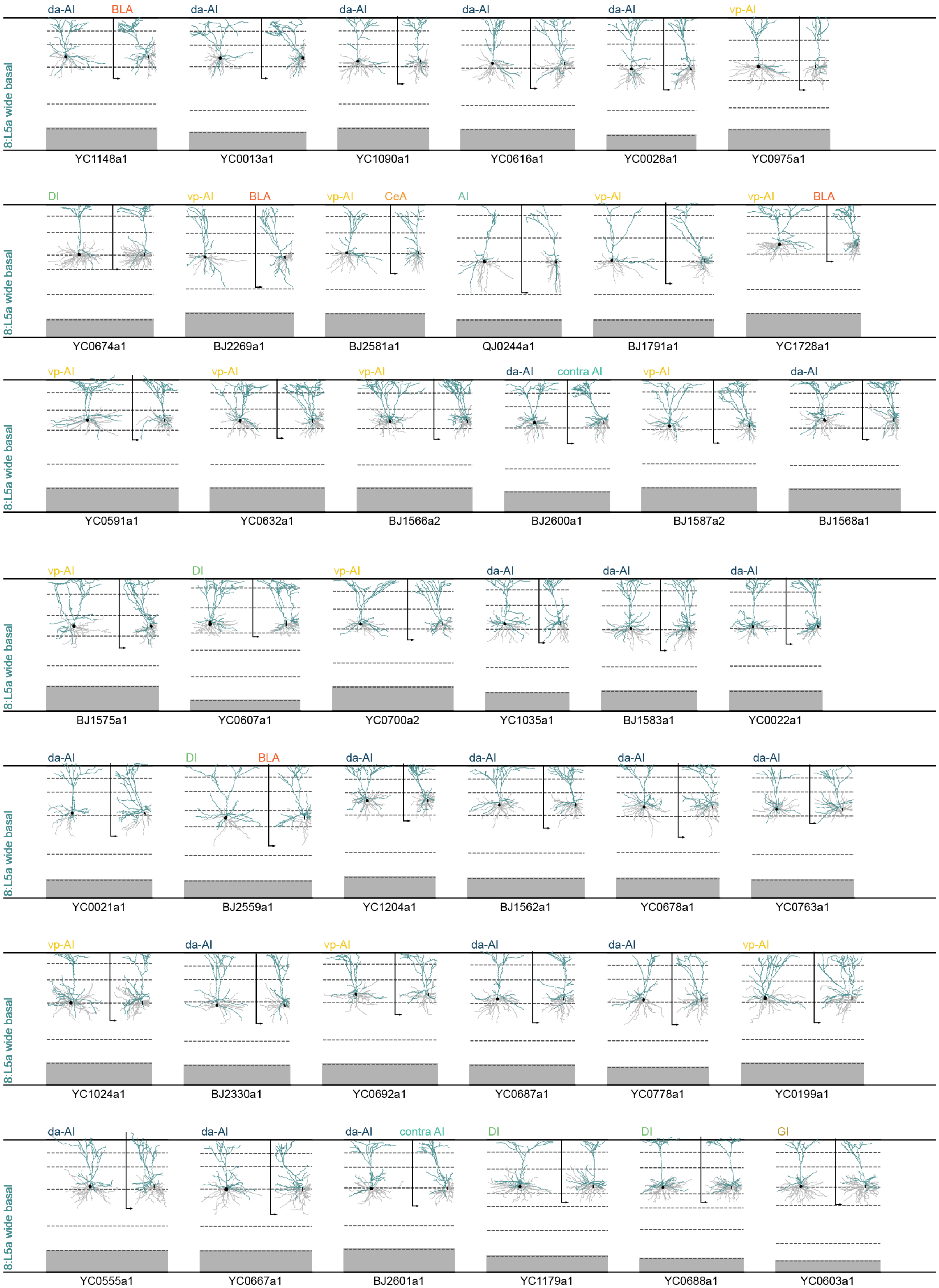
8:L5a wide basal
da-AI
BLA
vp-AI
DI
CeA
AI
contra AI
GI
YC1148a1
YC0013a1
YC1090a1
YC0616a1
YC0028a1
YC0975a1
YC0674a1
BJ2269a1
BJ2581a1
QJ0244a1
BJ1791a1
YC1728a1
YC0591a1
YC0632a1
BJ1566a2
BJ2600a1
BJ1587a2
BJ1568a1
BJ1575a1
YC0607a1
YC0700a2
YC1035a1
BJ1583a1
YC0022a1
YC0021a1
BJ2559a1
YC1204a1
BJ1562a1
YC0678a1
YC0763a1
YC1024a1
BJ2330a1
YC0692a1
YC0687a1
YC0778a1
YC0199a1
YC0555a1
YC0667a1
BJ2601a1
YC1179a1
YC0688a1
YC0603a1

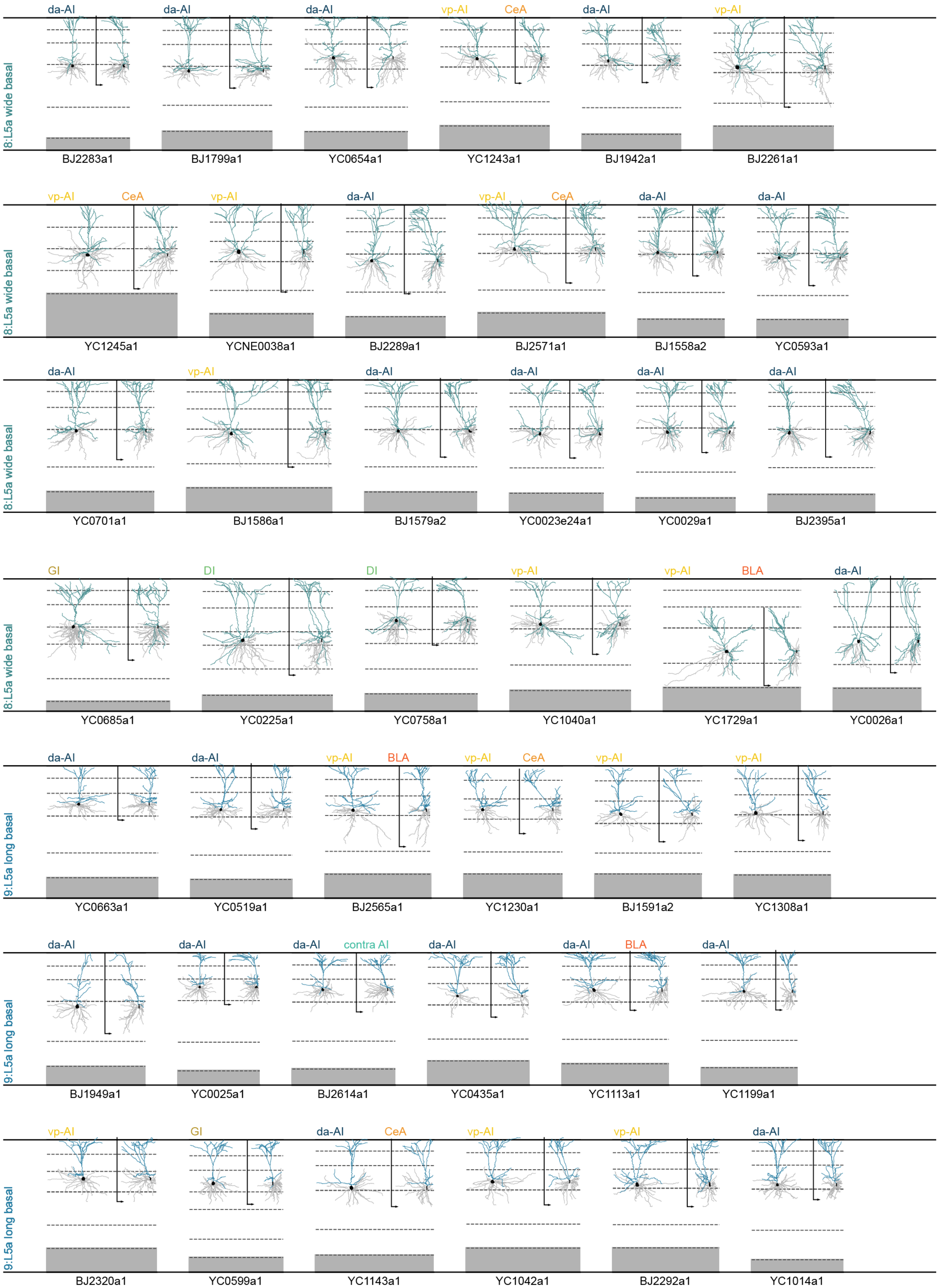
8:L5a wide basal
da-AI
da-AI
da-AI
vp-AI
CeA
da-AI
vp-AI
BJ2283a1
BJ1799a1
YC0654a1
YC1243a1
BJ1942a1
BJ2261a1
8:L5a wide basal
vp-AI
CeA
vp-AI
da-AI
vp-AI
CeA
da-AI
da-AI
YC1245a1
YCNE0038a1
BJ2289a1
BJ2571a1
BJ1558a2
YC0593a1
8:L5a wide basal
da-AI
vp-AI
da-AI
da-AI
da-AI
da-AI
YC0701a1
BJ1586a1
BJ1579a2
YC0023e24a1
YC0029a1
BJ2395a1
8:L5a wide basal
GI
DI
DI
vp-AI
vp-AI
BLA
da-AI
YC0685a1
YC0225a1
YC0758a1
YC1040a1
YC1729a1
YC0026a1
9:L5a long basal
da-AI
da-AI
vp-AI
BLA
vp-AI
CeA
vp-AI
vp-AI
YC0663a1
YC0519a1
BJ2565a1
YC1230a1
BJ1591a2
YC1308a1
9:L5a long basal
da-AI
da-AI
da-AI
contra AI
da-AI
da-AI
BLA
da-AI
BJ1949a1
YC0025a1
BJ2614a1
YC0435a1
YC1113a1
YC1199a1
9:L5a long basal
vp-AI
GI
da-AI
CeA
vp-AI
vp-AI
da-AI
BJ2320a1
YC0599a1
YC1143a1
YC1042a1
BJ2292a1
YC1014a1

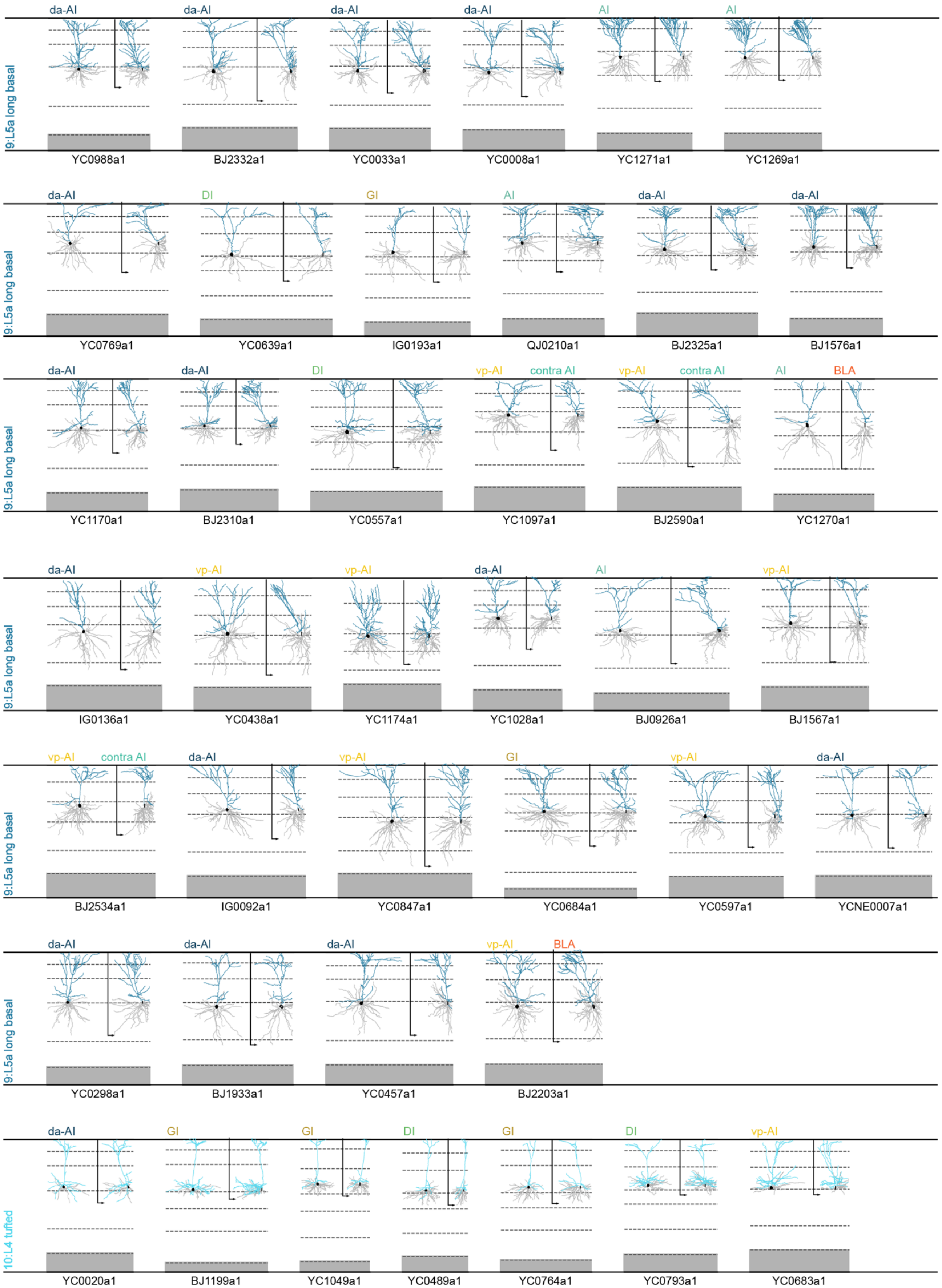
9:L5a long basal
da-AI
da-AI
da-AI
da-AI
AI
AI
YC0988a1
BJ2332a1
YC0033a1
YC0008a1
YC1271a1
YC1269a1
9:L5a long basal
da-AI
DI
GI
AI
da-AI
da-AI
YC0769a1
YC0639a1
IG0193a1
QJ0210a1
BJ2325a1
BJ1576a1
9:L5a long basal
da-AI
da-AI
DI
vp-AI
contra AI
vp-AI
contra AI
AI
BLA
YC1170a1
BJ2310a1
YC0557a1
YC1097a1
BJ2590a1
YC1270a1
9:L5a long basal
da-AI
vp-AI
vp-AI
da-AI
AI
vp-AI
IG0136a1
YC0438a1
YC1174a1
YC1028a1
BJ0926a1
BJ1567a1
9:L5a long basal
vp-AI
contra AI
da-AI
vp-AI
GI
vp-AI
da-AI
BJ2534a1
IG0092a1
YC0847a1
YC0684a1
YC0597a1
YCNE0007a1
9:L5a long basal
da-AI
da-AI
da-AI
vp-AI
BLA
YC0298a1
BJ1933a1
YC0457a1
BJ2203a1
10:L4 tufted
da-AI
GI
GI
DI
GI
DI
vp-AI
YC0020a1
BJ1199a1
YC1049a1
YC0489a1
YC0764a1
YC0793a1
YC0683a1

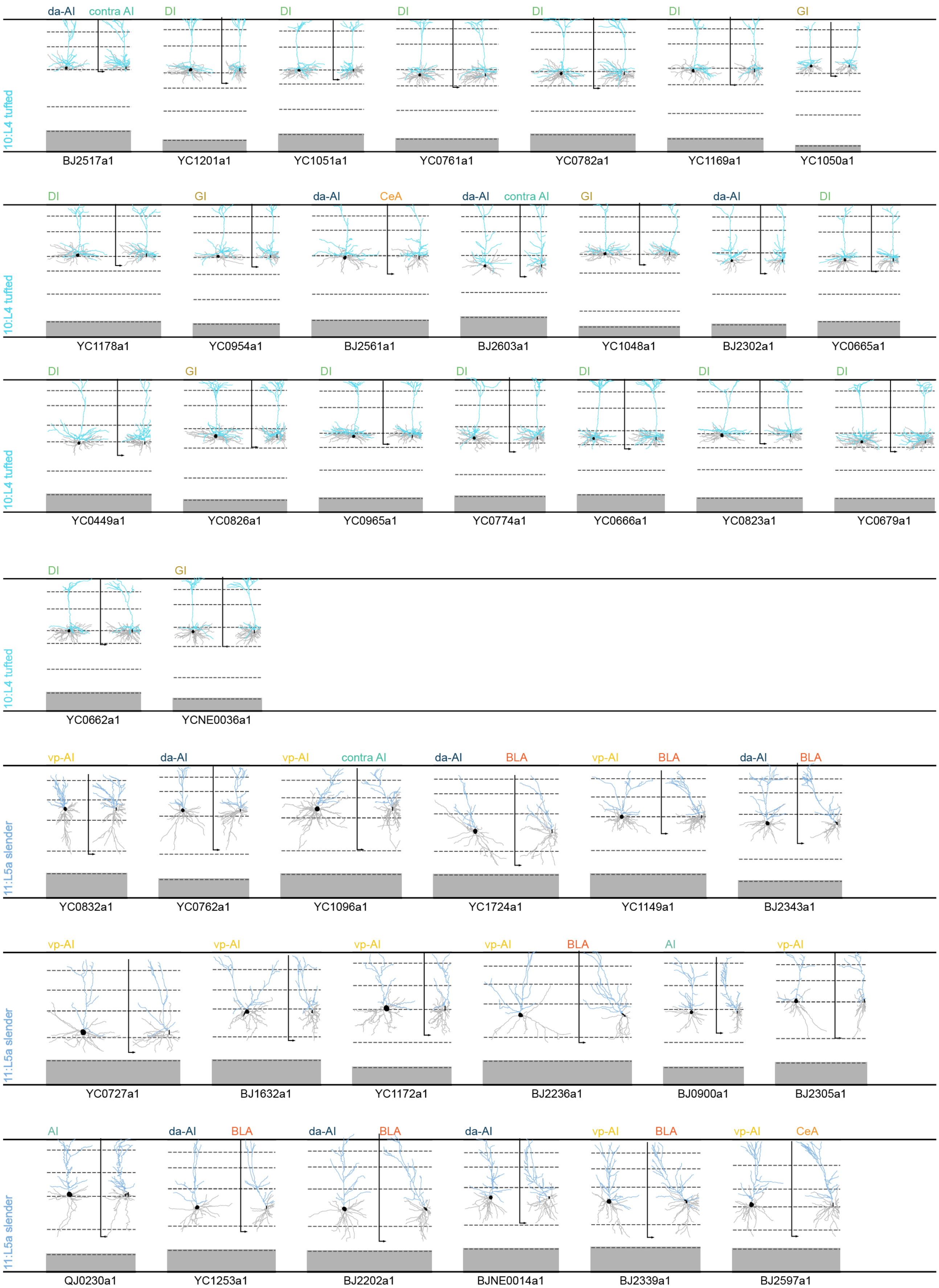
10:L4 tufted
da-AI
contra AI
DI
DI
DI
DI
DI
GI
BJ2517a1
YC1201a1
YC1051a1
YC0761a1
YC0782a1
YC1169a1
YC1050a1
10:L4 tufted
DI
GI
da-AI
CeA
da-AI
contra AI
GI
da-AI
DI
YC1178a1
YC0954a1
BJ2561a1
BJ2603a1
YC1048a1
BJ2302a1
YC0665a1
10:L4 tufted
DI
GI
DI
DI
DI
DI
DI
YC0449a1
YC0826a1
YC0965a1
YC0774a1
YC0666a1
YC0823a1
YC0679a1
10:L4 tufted
DI
GI
YC0662a1
YCNE0036a1
11:L5a slender
vp-AI
da-AI
vp-AI
contra AI
da-AI
BLA
vp-AI
BLA
da-AI
BLA
YC0832a1
YC0762a1
YC1096a1
YC1724a1
YC1149a1
BJ2343a1
11:L5a slender
vp-AI
vp-AI
vp-AI
vp-AI
BLA
AI
vp-AI
YC0727a1
BJ1632a1
YC1172a1
BJ2236a1
BJ0900a1
BJ2305a1
11:L5a slender
AI
da-AI
BLA
da-AI
BLA
da-AI
vp-AI
BLA
vp-AI
CeA
QJ0230a1
YC1253a1
BJ2202a1
BJNE0014a1
BJ2339a1
BJ2597a1

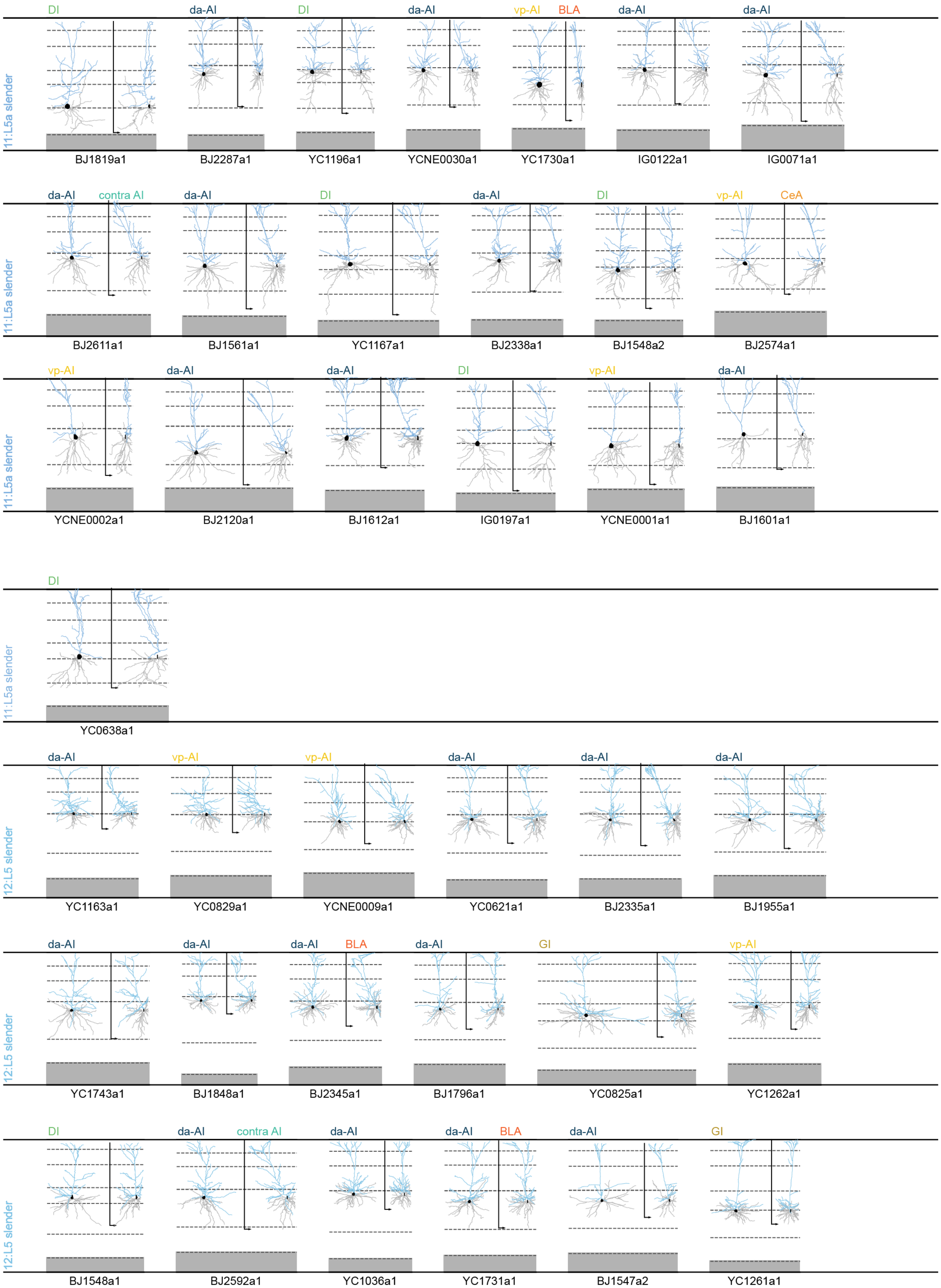

11:L5a slender
DI
da-AI
DI
da-AI
vp-AI
BLA
da-AI
da-AI
BJ1819a1
BJ2287a1
YC1196a1
YCNE0030a1
YC1730a1
IG0122a1
IG0071a1
11:L5a slender
da-AI
contra AI
da-AI
DI
da-AI
DI
vp-AI
CeA
BJ2611a1
BJ1561a1
YC1167a1
BJ2338a1
BJ1548a2
BJ2574a1
11:L5a slender
vp-AI
da-AI
da-AI
DI
vp-AI
da-AI
YCNE0002a1
BJ2120a1
BJ1612a1
IG0197a1
YCNE0001a1
BJ1601a1
11:L5a slender
DI
YC0638a1
12:L5 slender
da-AI
vp-AI
vp-AI
da-AI
da-AI
da-AI
YC1163a1
YC0829a1
YCNE0009a1
YC0621a1
BJ2335a1
BJ1955a1
12:L5 slender
da-AI
da-AI
da-AI
BLA
da-AI
GI
vp-AI
YC1743a1
BJ1848a1
BJ2345a1
BJ1796a1
YC0825a1
YC1262a1
12:L5 slender
DI
da-AI
contra AI
da-AI
da-AI
BLA
da-AI
GI
BJ1548a1
BJ2592a1
YC1036a1
YC1731a1
BJ1547a2
YC1261a1

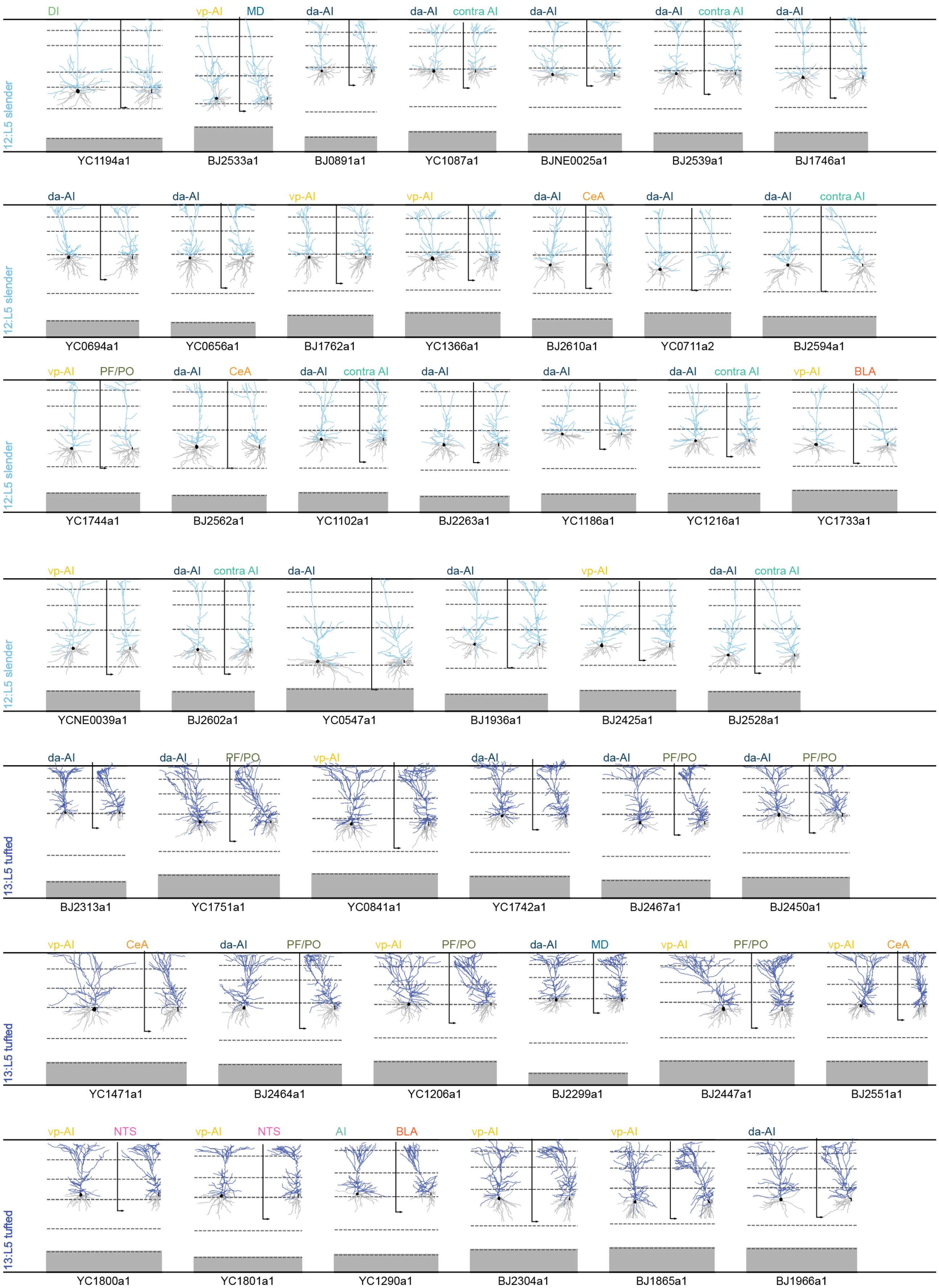
12:L5 slender
DI
YC1194a1
vp-AI MD
BJ2533a1
da-AI
BJ0891a1
da-AI contra AI
YC1087a1
da-AI
BJNE0025a1
da-AI contra AI
BJ2539a1
da-AI
BJ1746a1
12:L5 slender
da-AI
YC0694a1
da-AI
YC0656a1
vp-AI
BJ1762a1
vp-AI
YC1366a1
da-AI CeA
BJ2610a1
da-AI
YC0711a2
da-AI contra AI
BJ2594a1
12:L5 slender
vp-AI PF/PO
YC1744a1
da-AI CeA
BJ2562a1
da-AI contra AI
YC1102a1
da-AI
BJ2263a1
da-AI
YC1186a1
da-AI contra AI
YC1216a1
vp-AI BLA
YC1733a1
12:L5 slender
vp-AI
YCNE0039a1
da-AI contra AI
BJ2602a1
da-AI
YC0547a1
da-AI
BJ1936a1
vp-AI
BJ2425a1
da-AI contra AI
BJ2528a1
13:L5 tufted
da-AI
BJ2313a1
da-AI PF/PO
YC1751a1
vp-AI
YC0841a1
da-AI
YC1742a1
da-AI PF/PO
BJ2467a1
da-AI PF/PO
BJ2450a1
13:L5 tufted
vp-AI CeA
YC1471a1
da-AI PF/PO
BJ2464a1
vp-AI PF/PO
YC1206a1
da-AI MD
BJ2299a1
vp-AI PF/PO
BJ2447a1
vp-AI CeA
BJ2551a1
13:L5 tufted
vp-AI NTS
YC1800a1
vp-AI NTS
YC1801a1
AI BLA
YC1290a1
vp-AI
BJ2304a1
vp-AI
BJ1865a1
da-AI
BJ1966a1

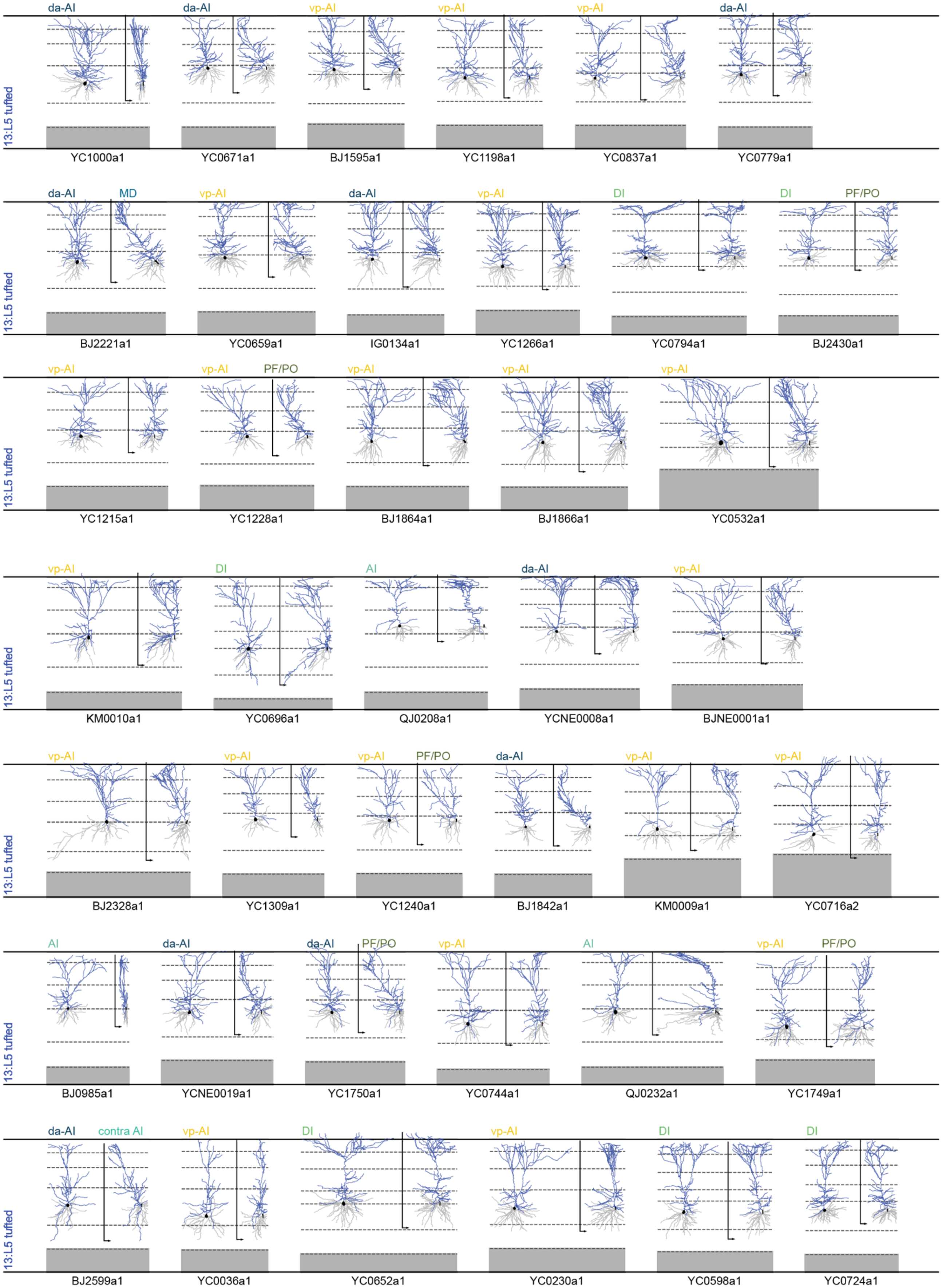
13:L5 tufted
da-AI
vp-AI
MD
DI
PF/PO
AI
contra AI
YC1000a1
YC0671a1
BJ1595a1
YC1198a1
YC0837a1
YC0779a1
BJ2221a1
YC0659a1
IG0134a1
YC1266a1
YC0794a1
BJ2430a1
YC1215a1
YC1228a1
BJ1864a1
BJ1866a1
YC0532a1
KM0010a1
YC0696a1
QJ0208a1
YCNE0008a1
BJNE0001a1
BJ2328a1
YC1309a1
YC1240a1
BJ1842a1
KM0009a1
YC0716a2
BJ0985a1
YCNE0019a1
YC1750a1
YC0744a1
QJ0232a1
YC1749a1
BJ2599a1
YC0036a1
YC0652a1
YC0230a1
YC0598a1
YC0724a1

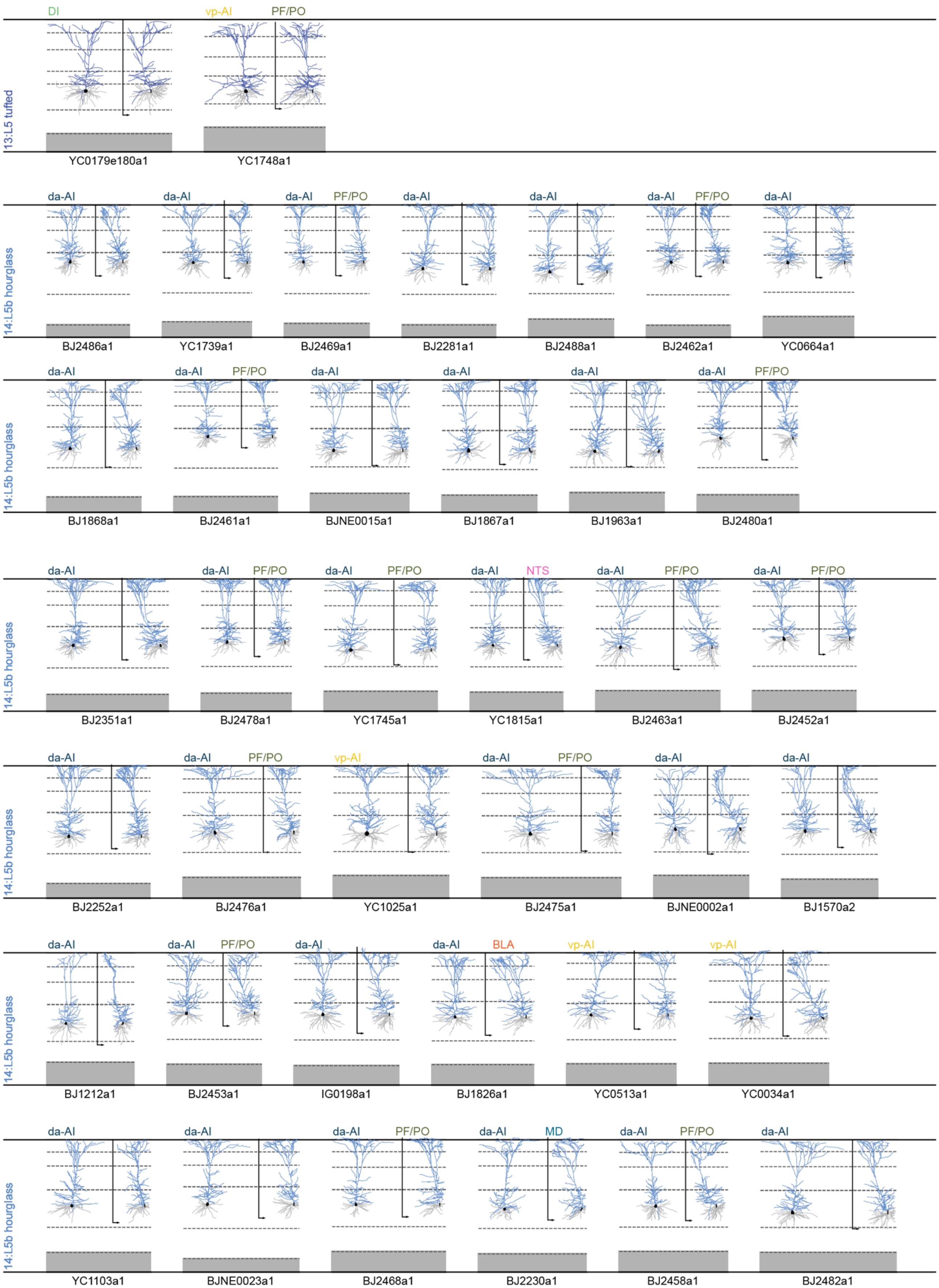
13:L5 tufted
DI
vp-AI
PF/PO
YC0179e180a1
YC1748a1
14:L5b hourglass
da-AI
PF/PO
BJ2486a1
YC1739a1
BJ2469a1
BJ2281a1
BJ2488a1
BJ2462a1
YC0664a1
BJ1868a1
BJ2461a1
BJNE0015a1
BJ1867a1
BJ1963a1
BJ2480a1
NTS
BJ2351a1
BJ2478a1
YC1745a1
YC1815a1
BJ2463a1
BJ2452a1
vp-AI
BJ2252a1
BJ2476a1
YC1025a1
BJ2475a1
BJNE0002a1
BJ1570a2
BLA
BJ1212a1
BJ2453a1
IG0198a1
BJ1826a1
YC0513a1
YC0034a1
MD
YC1103a1
BJNE0023a1
BJ2468a1
BJ2230a1
BJ2458a1
BJ2482a1

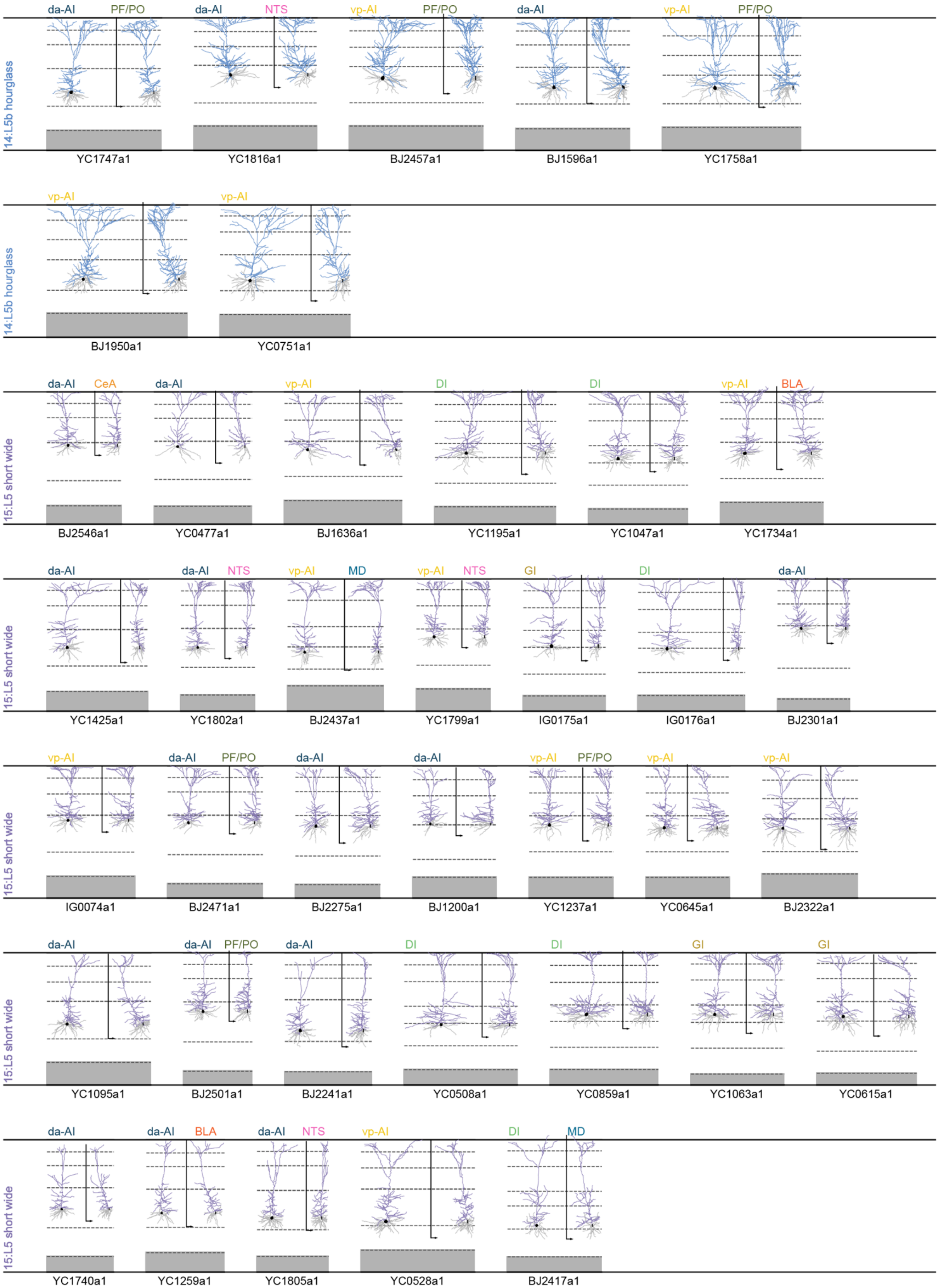
14:L5b hourglass
da-AI PF/PO
YC1747a1
da-AI NTS
YC1816a1
vp-AI PF/PO
BJ2457a1
da-AI
BJ1596a1
vp-AI PF/PO
YC1758a1
14:L5b hourglass
vp-AI
BJ1950a1
vp-AI
YC0751a1
15:L5 short wide
da-AI CeA
BJ2546a1
da-AI
YC0477a1
vp-AI
BJ1636a1
DI
YC1195a1
DI
YC1047a1
vp-AI BLA
YC1734a1
15:L5 short wide
da-AI
YC1425a1
da-AI NTS
YC1802a1
vp-AI MD
BJ2437a1
vp-AI NTS
YC1799a1
GI
IG0175a1
DI
IG0176a1
da-AI
BJ2301a1
15:L5 short wide
vp-AI
IG0074a1
da-AI PF/PO
BJ2471a1
da-AI
BJ2275a1
da-AI
BJ1200a1
vp-AI PF/PO
YC1237a1
vp-AI
YC0645a1
vp-AI
BJ2322a1
15:L5 short wide
da-AI
YC1095a1
da-AI PF/PO
BJ2501a1
da-AI
BJ2241a1
DI
YC0508a1
DI
YC0859a1
GI
YC1063a1
GI
YC0615a1
15:L5 short wide
da-AI
YC1740a1
da-AI BLA
YC1259a1
da-AI NTS
YC1805a1
vp-AI
YC0528a1
DI MD
BJ2417a1

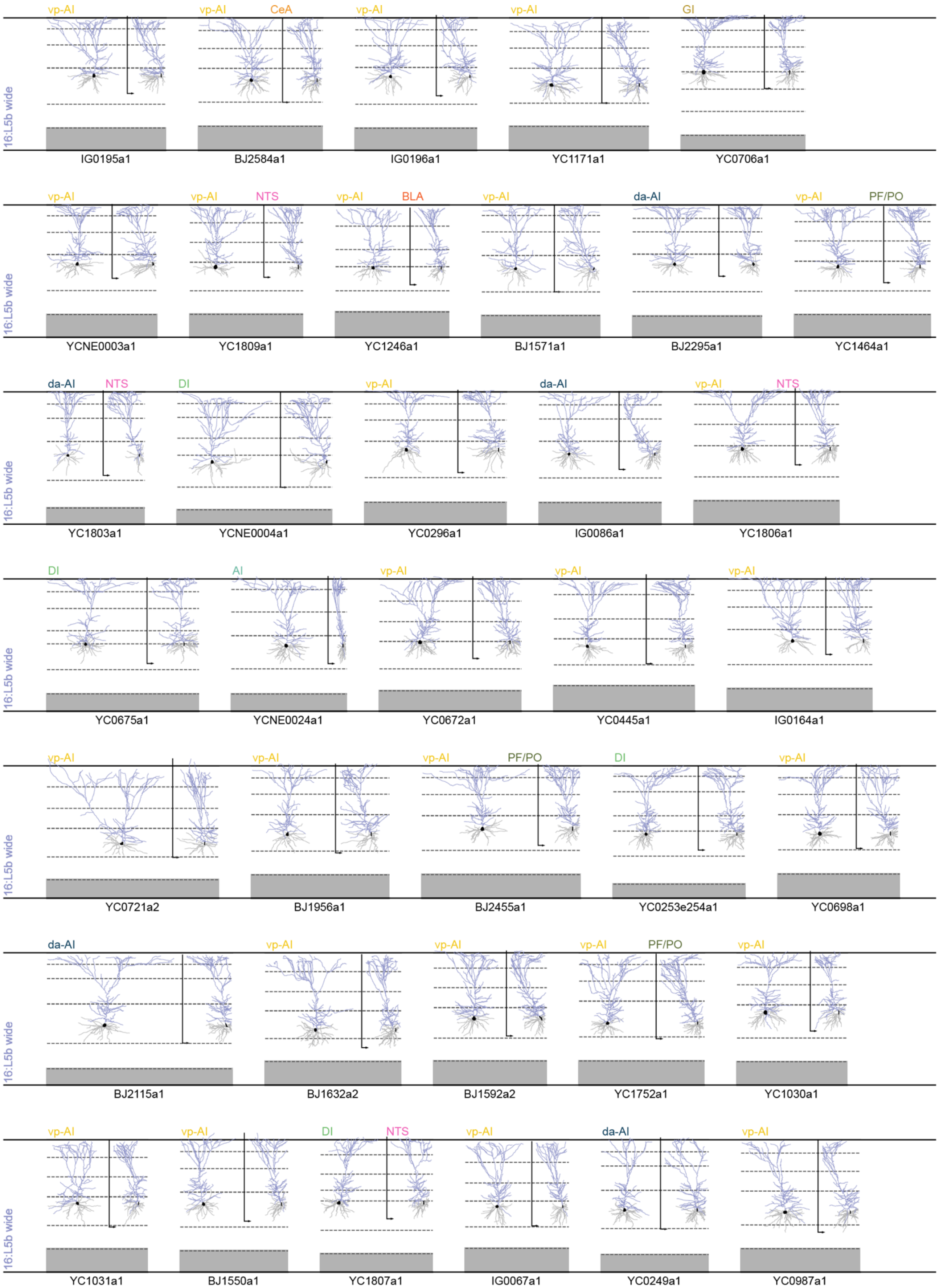
vp-AI
IG0195a1
vp-AI
CeA
BJ2584a1
vp-AI
IG0196a1
vp-AI
YC1171a1
GI
YC0706a1
16:L5b wide
vp-AI
YCNE0003a1
vp-AI
NTS
YC1809a1
vp-AI
BLA
YC1246a1
vp-AI
BJ1571a1
da-AI
BJ2295a1
vp-AI
PF/PO
YC1464a1
16:L5b wide
da-AI
NTS
YC1803a1
DI
YCNE0004a1
vp-AI
YC0296a1
da-AI
IG0086a1
vp-AI
NTS
YC1806a1
16:L5b wide
DI
YC0675a1
AI
YCNE0024a1
vp-AI
YC0672a1
vp-AI
YC0445a1
vp-AI
IG0164a1
16:L5b wide
vp-AI
YC0721a2
vp-AI
BJ1956a1
vp-AI
PF/PO
BJ2455a1
DI
YC0253e254a1
vp-AI
YC0698a1
16:L5b wide
da-AI
BJ2115a1
vp-AI
BJ1632a2
vp-AI
BJ1592a2
vp-AI
PF/PO
YC1752a1
vp-AI
YC1030a1
16:L5b wide
vp-AI
YC1031a1
vp-AI
BJ1550a1
DI
NTS
YC1807a1
vp-AI
IG0067a1
da-AI
YC0249a1
vp-AI
YC0987a1
16:L5b wide

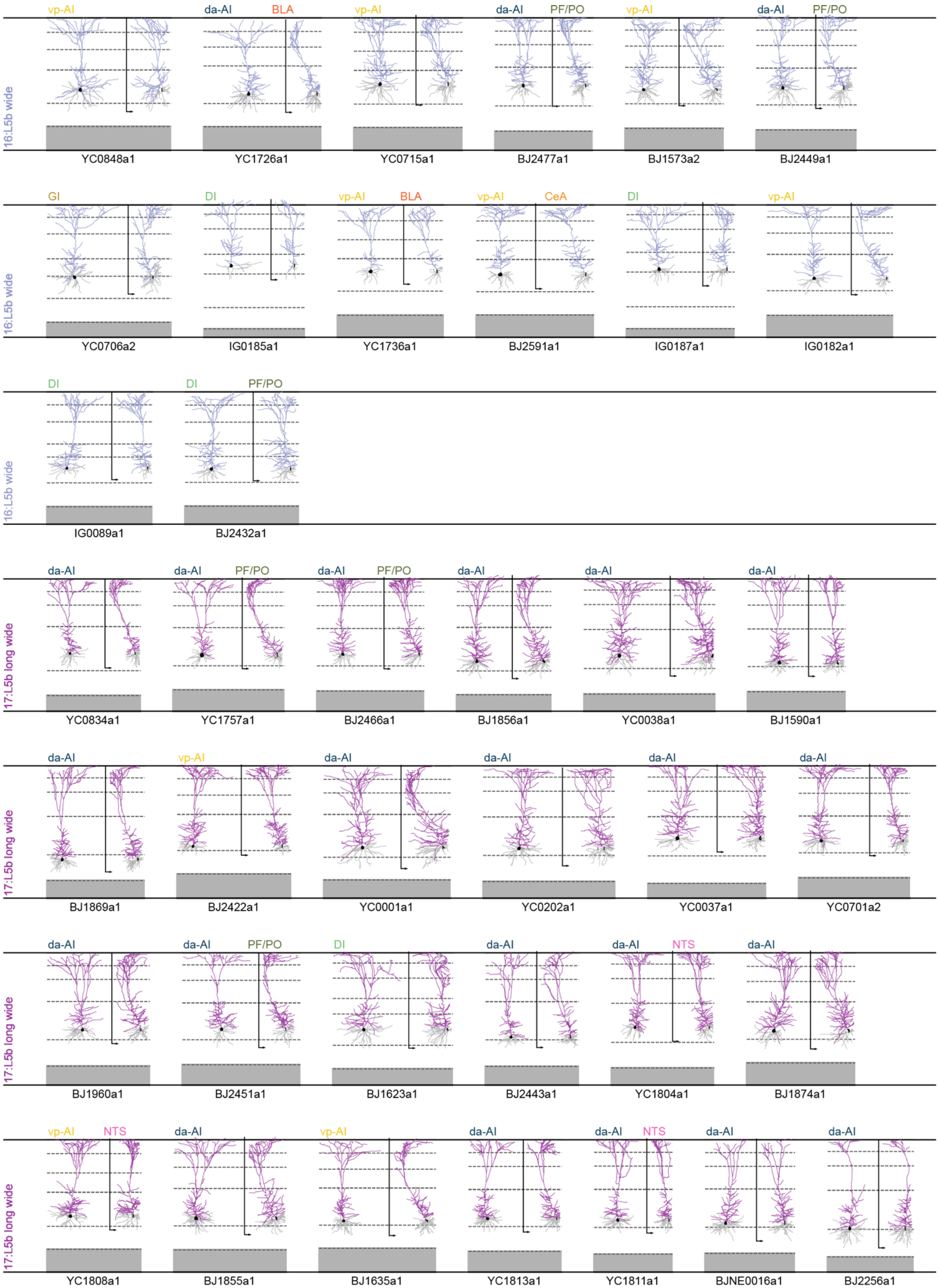
vp-AI
da-AI
BLA
vp-AI
da-AI
PF/PO
vp-AI
da-AI
PF/PO
16:L5b wide
YC0848a1
YC1726a1
YC0715a1
BJ2477a1
BJ1573a2
BJ2449a1
GI
DI
vp-AI
BLA
vp-AI
CeA
DI
vp-AI
16:L5b wide
YC0706a2
IG0185a1
YC1736a1
BJ2591a1
IG0187a1
IG0182a1
DI
DI
PF/PO
16:L5b wide
IG0089a1
BJ2432a1
da-AI
da-AI
PF/PO
da-AI
PF/PO
da-AI
da-AI
da-AI
17:L5b long wide
YC0834a1
YC1757a1
BJ2466a1
BJ1856a1
YC0038a1
BJ1590a1
da-AI
vp-AI
da-AI
da-AI
da-AI
da-AI
17:L5b long wide
BJ1869a1
BJ2422a1
YC0001a1
YC0202a1
YC0037a1
YC0701a2
da-AI
da-AI
PF/PO
DI
da-AI
da-AI
NTS
da-AI
17:L5b long wide
BJ1960a1
BJ2451a1
BJ1623a1
BJ2443a1
YC1804a1
BJ1874a1
vp-AI
NTS
da-AI
vp-AI
da-AI
da-AI
NTS
da-AI
da-AI
17:L5b long wide
YC1808a1
BJ1855a1
BJ1635a1
YC1813a1
YC1811a1
BJNE0016a1
BJ2256a1

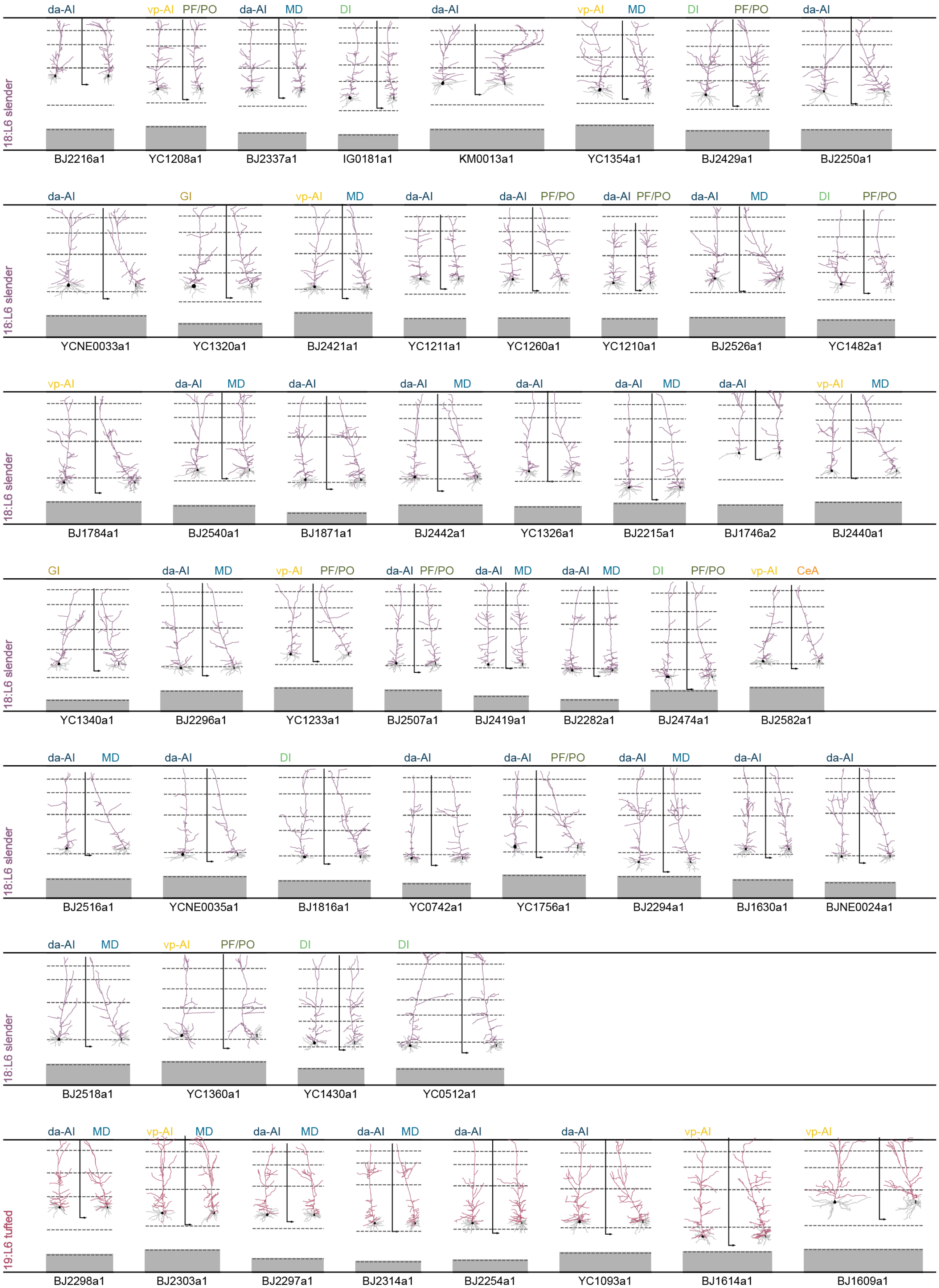
18:L6 slender
da-AI
vp-AI PF/PO
da-AI MD
DI
da-AI
vp-AI MD
DI PF/PO
da-AI
BJ2216a1
YC1208a1
BJ2337a1
IG0181a1
KM0013a1
YC1354a1
BJ2429a1
BJ2250a1
18:L6 slender
da-AI
GI
vp-AI MD
da-AI
da-AI PF/PO
da-AI PF/PO
da-AI MD
DI PF/PO
YCNE0033a1
YC1320a1
BJ2421a1
YC1211a1
YC1260a1
YC1210a1
BJ2526a1
YC1482a1
18:L6 slender
vp-AI
da-AI MD
da-AI
da-AI MD
da-AI
da-AI MD
da-AI
vp-AI MD
BJ1784a1
BJ2540a1
BJ1871a1
BJ2442a1
YC1326a1
BJ2215a1
BJ1746a2
BJ2440a1
18:L6 slender
GI
da-AI MD
vp-AI PF/PO
da-AI PF/PO
da-AI MD
da-AI MD
DI PF/PO
vp-AI CeA
YC1340a1
BJ2296a1
YC1233a1
BJ2507a1
BJ2419a1
BJ2282a1
BJ2474a1
BJ2582a1
18:L6 slender
da-AI MD
da-AI
DI
da-AI
da-AI PF/PO
da-AI MD
da-AI
da-AI
BJ2516a1
YCNE0035a1
BJ1816a1
YC0742a1
YC1756a1
BJ2294a1
BJ1630a1
BJNE0024a1
18:L6 slender
da-AI MD
vp-AI PF/PO
DI
DI
BJ2518a1
YC1360a1
YC1430a1
YC0512a1
19:L6 tufted
da-AI MD
vp-AI MD
da-AI MD
da-AI MD
da-AI
da-AI
vp-AI
vp-AI
BJ2298a1
BJ2303a1
BJ2297a1
BJ2314a1
BJ2254a1
YC1093a1
BJ1614a1
BJ1609a1

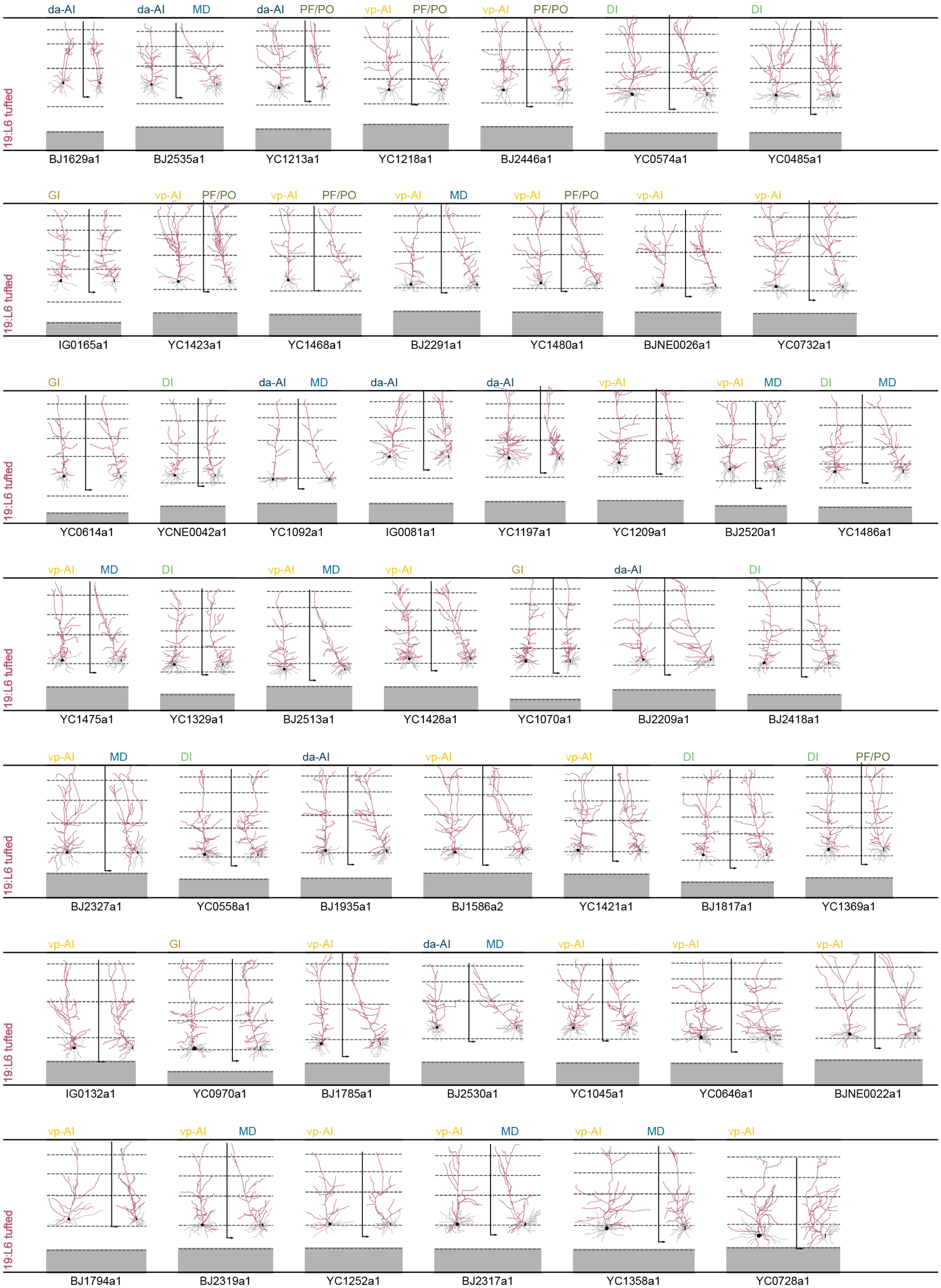

19:L6 tufted
da-AI
da-AI MD
da-AI PF/PO
vp-AI PF/PO
vp-AI PF/PO
DI
DI
BJ1629a1 BJ2535a1 YC1213a1 YC1218a1 BJ2446a1 YC0574a1 YC0485a1
19:L6 tufted
GI
vp-AI PF/PO
vp-AI PF/PO
vp-AI MD
vp-AI PF/PO
vp-AI
vp-AI
IG0165a1 YC1423a1 YC1468a1 BJ2291a1 YC1480a1 BJNE0026a1 YC0732a1
19:L6 tufted
GI
DI
da-AI MD
da-AI
da-AI
vp-AI
vp-AI MD
DI MD
YC0614a1 YCNE0042a1 YC1092a1 IG0081a1 YC1197a1 YC1209a1 BJ2520a1 YC1486a1
19:L6 tufted
vp-AI MD
DI
vp-AI MD
vp-AI
GI
da-AI
DI
YC1475a1 YC1329a1 BJ2513a1 YC1428a1 YC1070a1 BJ2209a1 BJ2418a1
19:L6 tufted
vp-AI MD
DI
da-AI
vp-AI
vp-AI
DI
DI PF/PO
BJ2327a1 YC0558a1 BJ1935a1 BJ1586a2 YC1421a1 BJ1817a1 YC1369a1
19:L6 tufted
vp-AI
GI
vp-AI
da-AI MD
vp-AI
vp-AI
vp-AI
IG0132a1 YC0970a1 BJ1785a1 BJ2530a1 YC1045a1 YC0646a1 BJNE0022a1
19:L6 tufted
vp-AI
vp-AI MD
vp-AI
vp-AI MD
vp-AI MD
vp-AI
BJ1794a1 BJ2319a1 YC1252a1 BJ2317a1 YC1358a1 YC0728a1

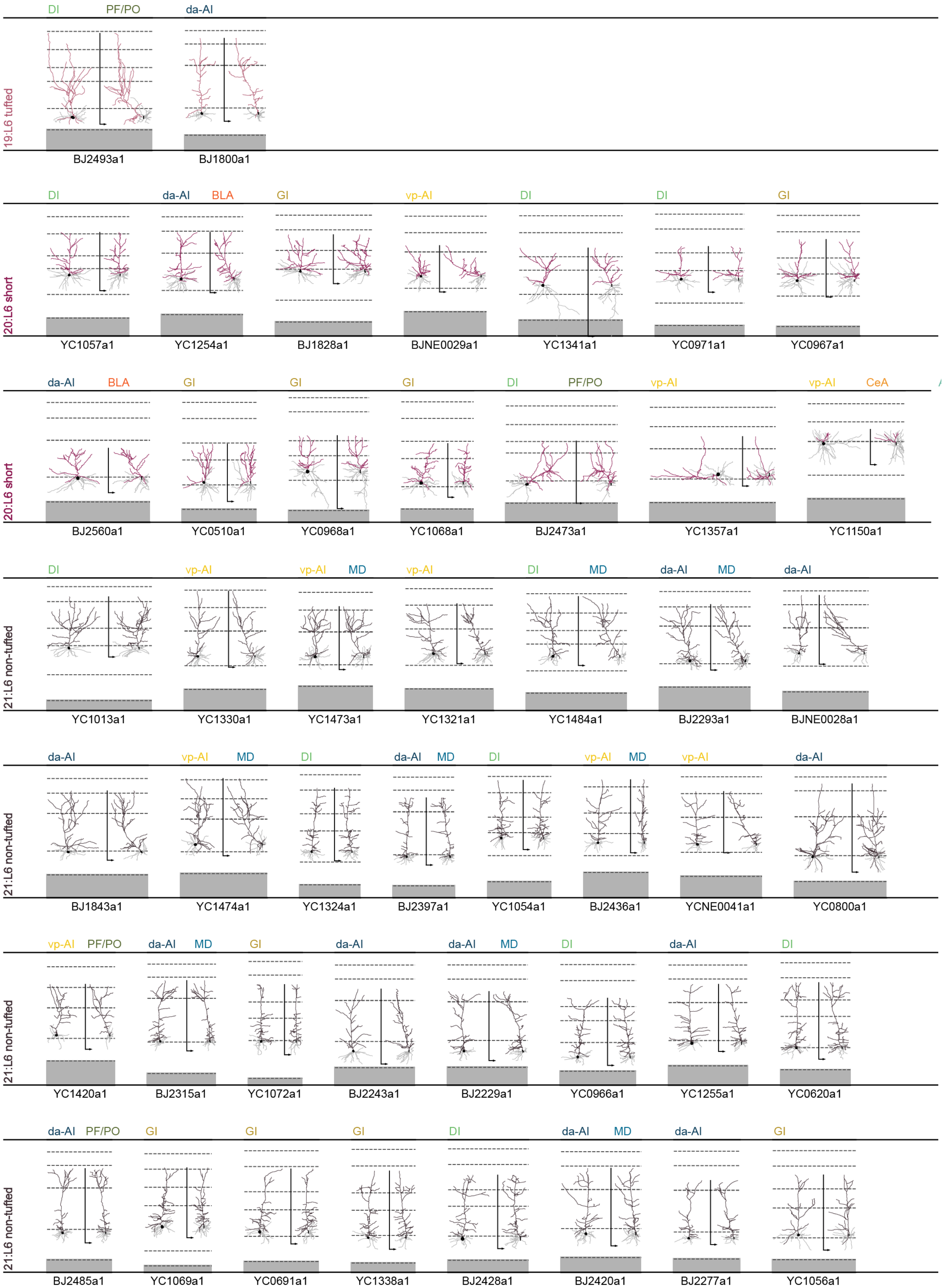

DI
PF/PO
da-AI
19:L6 tufted
BJ2493a1
BJ1800a1
DI
da-AI
BLA
GI
vp-AI
DI
DI
GI
20:L6 short
YC1057a1
YC1254a1
BJ1828a1
BJNE0029a1
YC1341a1
YC0971a1
YC0967a1
da-AI
BLA
GI
GI
GI
DI
PF/PO
vp-AI
vp-AI
CeA
20:L6 short
BJ2560a1
YC0510a1
YC0968a1
YC1068a1
BJ2473a1
YC1357a1
YC1150a1
DI
vp-AI
vp-AI
MD
vp-AI
DI
MD
da-AI
MD
da-AI
21:L6 non-tufted
YC1013a1
YC1330a1
YC1473a1
YC1321a1
YC1484a1
BJ2293a1
BJNE0028a1
da-AI
vp-AI
MD
DI
da-AI
MD
DI
vp-AI
MD
vp-AI
da-AI
21:L6 non-tufted
BJ1843a1
YC1474a1
YC1324a1
BJ2397a1
YC1054a1
BJ2436a1
YCNE0041a1
YC0800a1
vp-AI
PF/PO
da-AI
MD
GI
da-AI
da-AI
MD
DI
da-AI
DI
21:L6 non-tufted
YC1420a1
BJ2315a1
YC1072a1
BJ2243a1
BJ2229a1
YC0966a1
YC1255a1
YC0620a1
da-AI
PF/PO
GI
GI
GI
DI
da-AI
MD
da-AI
GI
21:L6 non-tufted
BJ2485a1
YC1069a1
YC0691a1
YC1338a1
BJ2428a1
BJ2420a1
BJ2277a1
YC1056a1

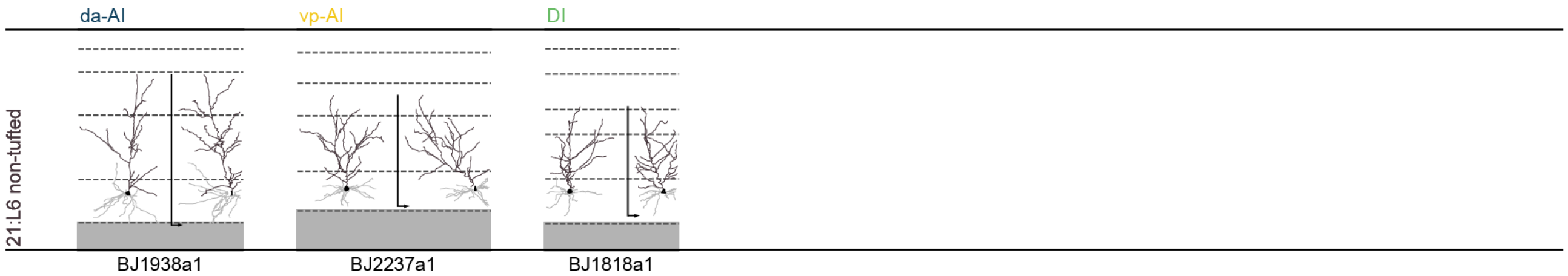

Supplementary Fig. 4: Overview of 958 reconstructed dendritic morphologies. Apical (colored) and basal (gray) dendrites shown in the *en face* (left) and side-view (right) planes for each reconstructed morphology that passed quality control—these morphologies were used for iCCA and as the classifier training dataset (Methods). Dimensions are scaled to the local cortical thickness. Dashed lines demarcate layer boundaries between pia (top) and corpus callosum (bottom) for L1, L2, L3, L4[#], L5, L6, and claustrum (gray zone), as determined by mean local layer thickness following registration to the Nissl atlas (Extended Data Figs. 1h and 2, Supplementary Fig. 3). Corresponding insular subregion allocations (top-left) and projection targets (top-right, if determined) are shown as text. Individual morphologies are sorted within each M-type based on cluster analysis dendrogram. [#]: L4 is only shown when located in the (dys)granular insula. da-AI: dorsoanterior agranular insula; vp-AI: ventroposterior agranular insula; AI[*]: agranular insula; DI: dysgranular insula; GI: granular insula; BLA: basolateral amygdala; CeA: central amygdala; contra AI: contralateral agranular insula; MD: mediodorsal thalamus; NTS: nucleus tractus solitarius; PF/PO: parafascicular/posterior thalamus. [*]: 39 cells were recorded in acute coronal brain slices and thus not assigned to AI subregions.

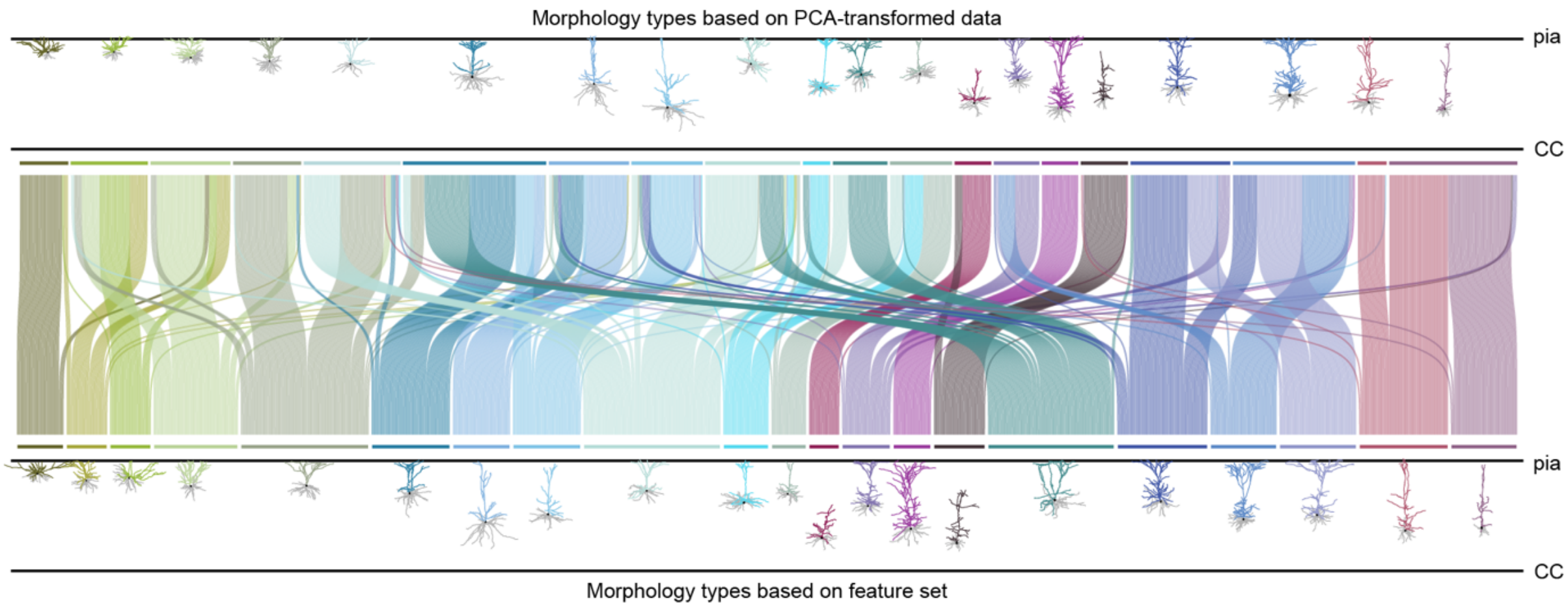

Supplementary Fig. 5: Comparison of M-type allocations between two iCCA approaches. Representative morphologies for each cluster based on the PCA-transformed dataset (top row) or the best-performing iCCA unique feature set (bottom row) and cell-wise correspondence between the two analysis approaches (adjusted Rand index = 0.36).

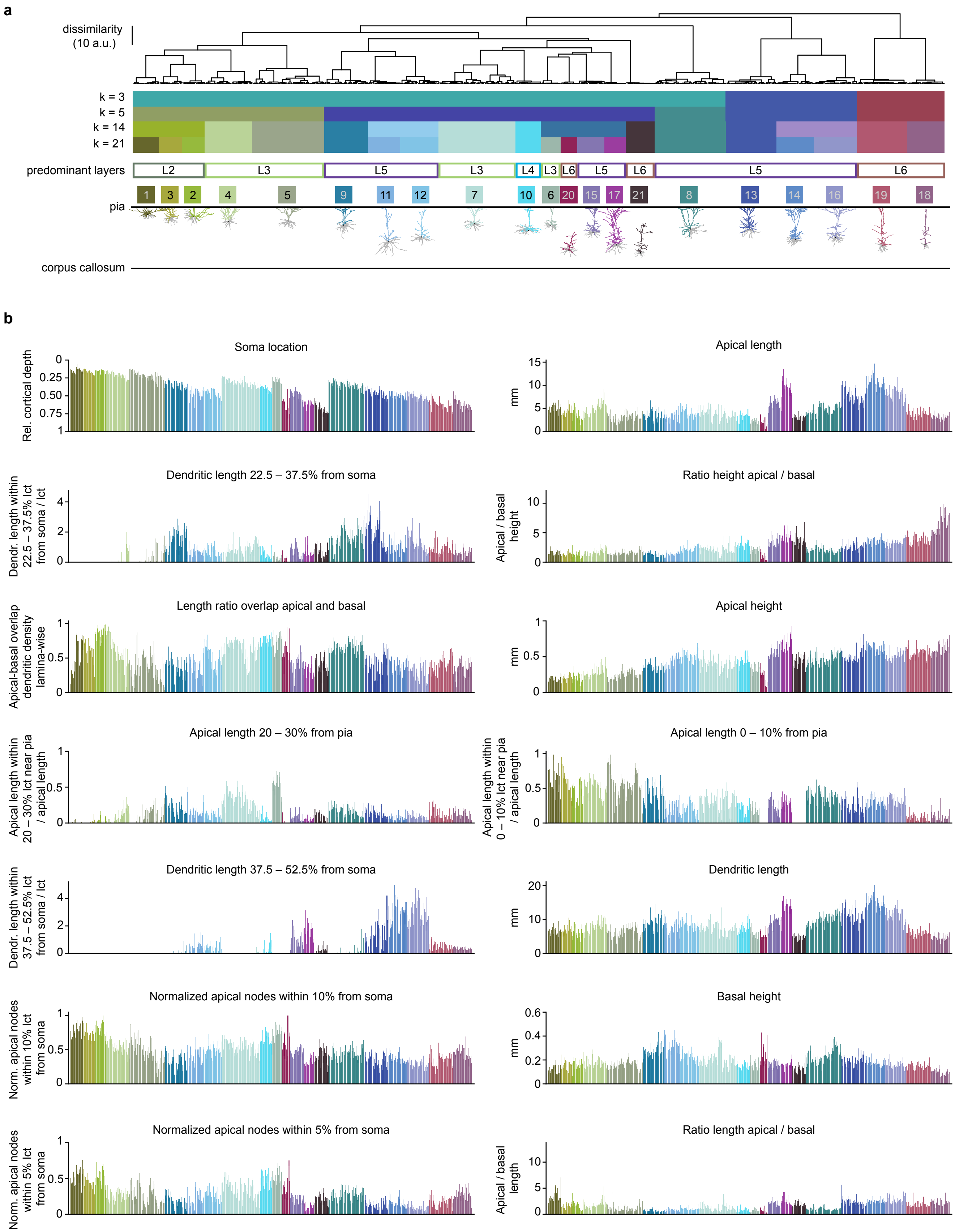
a
dissimilarity
(10 a.u.)
k = 3
k = 5
k = 14
k = 21
predominant layers
L2
L3
L5
L3
L4
L3
L6
L5
L6
L5
L6
1 3 2 4 5 9 11 12 7 10 6 20 15 17 21 8 13 14 16 19 18
pia
corpus callosum
b
Soma location
Rel. cortical depth
Apical length
mm
Dendritic length 22.5 – 37.5% from soma
Dendr. length within 22.5 – 37.5% lct from soma / lct
Ratio height apical / basal
Apical / basal height
Length ratio overlap apical and basal
Apical-basal overlap dendritic density lamina-wise
Apical height
mm
Apical length 20 – 30% from pia
Apical length within 20 – 30% lct near pia / apical length
Apical length 0 – 10% from pia
Apical length within 0 – 10% lct near pia / apical length
Dendritic length 37.5 – 52.5% from soma
Dendr. length within 37.5 – 52.5% lct from soma / lct
Dendritic length
mm
Normalized apical nodes within 10% from soma
Norm. apical nodes within 10% lct from soma
Basal height
mm
Normalized apical nodes within 5% from soma
Norm. apical nodes within 5% lct from soma
Ratio length apical / basal
Apical / basal length

b (continued)

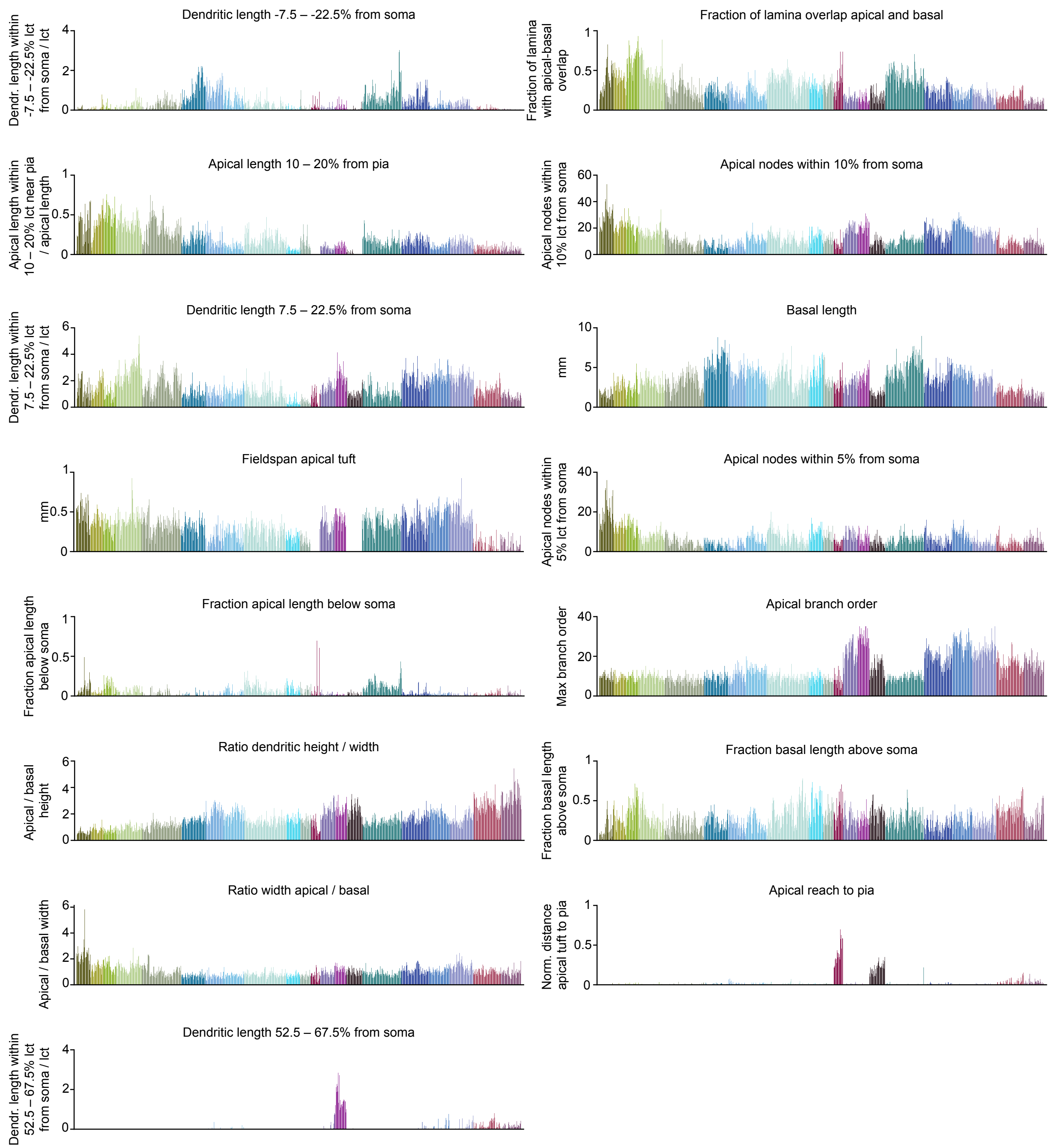

Supplementary Fig. 6: Distinct features of M-types. a, Consensus cluster dendrogram cut at different levels (k = 3, 5, 14, 21 clusters) indicated by color coding below the dendrogram to show how M-types relate hierarchically. The representative morphologies (bottom) were aligned to k = 21 clusters, same as in Fig. 2a.

Layer allocation was based on the predominant layer. **b**, All 29 features used for the iCCA, sorted by random forest classifier-derived Gini-index (Extended Data Fig. 4a). Histogram bars represent individual cells (n = 958) and are sorted in the same sequence as the dendrogram in (**a**), color-coded based on corresponding M-types.

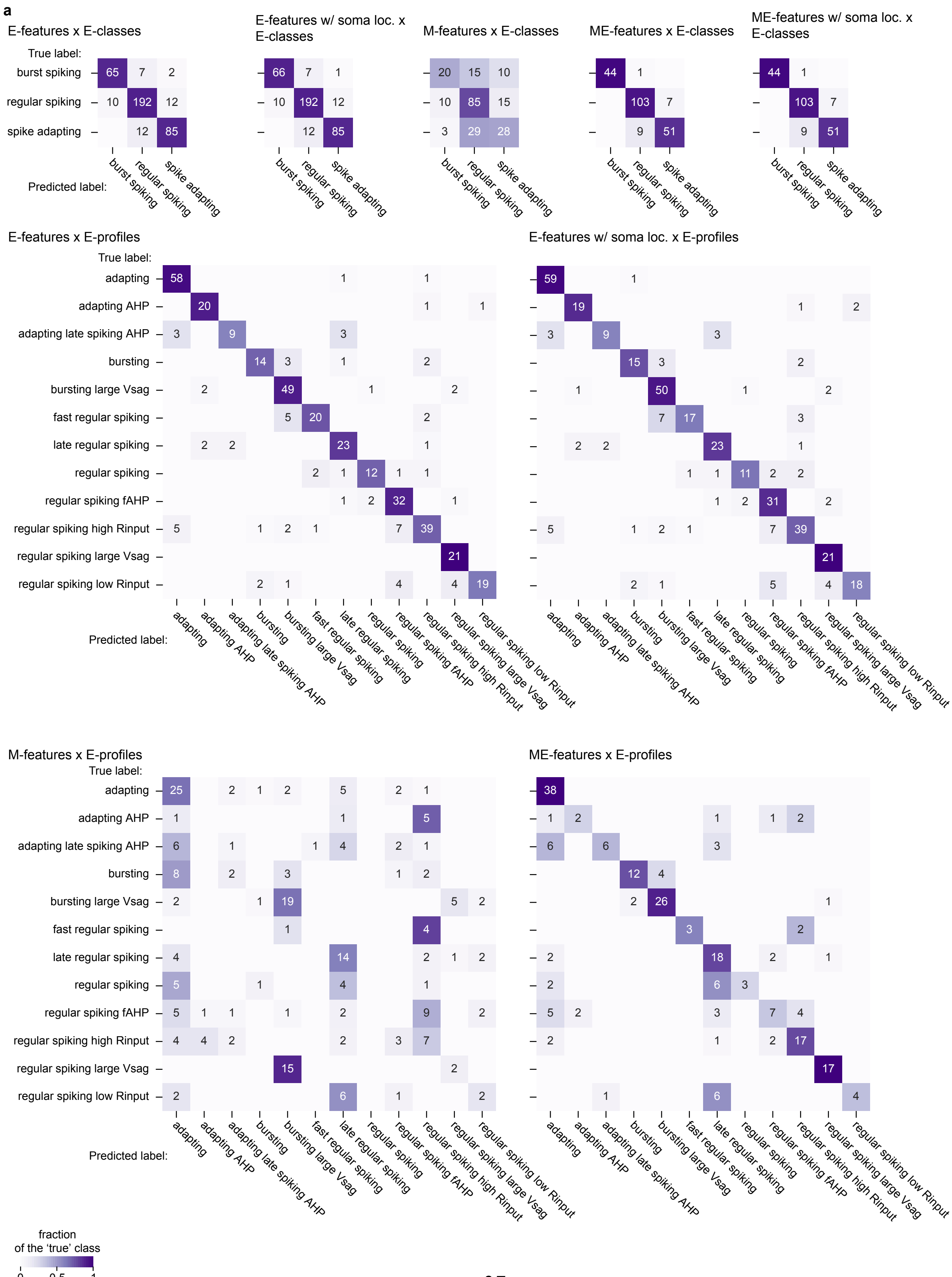
a
E-features x E-classes
E-features w/ soma loc. x E-classes
M-features x E-classes
ME-features x E-classes
ME-features w/ soma loc. x E-classes
True label:
burst spiking
regular spiking
spike adapting
Predicted label:
E-features x E-profiles
E-features w/ soma loc. x E-profiles
M-features x E-profiles
ME-features x E-profiles
adapting
adapting AHP
adapting late spiking AHP
bursting
bursting large Vsag
fast regular spiking
late regular spiking
regular spiking
regular spiking fAHP
regular spiking high Rinput
regular spiking large Vsag
regular spiking low Rinput
fraction
of the 'true' class
0
0.5
1

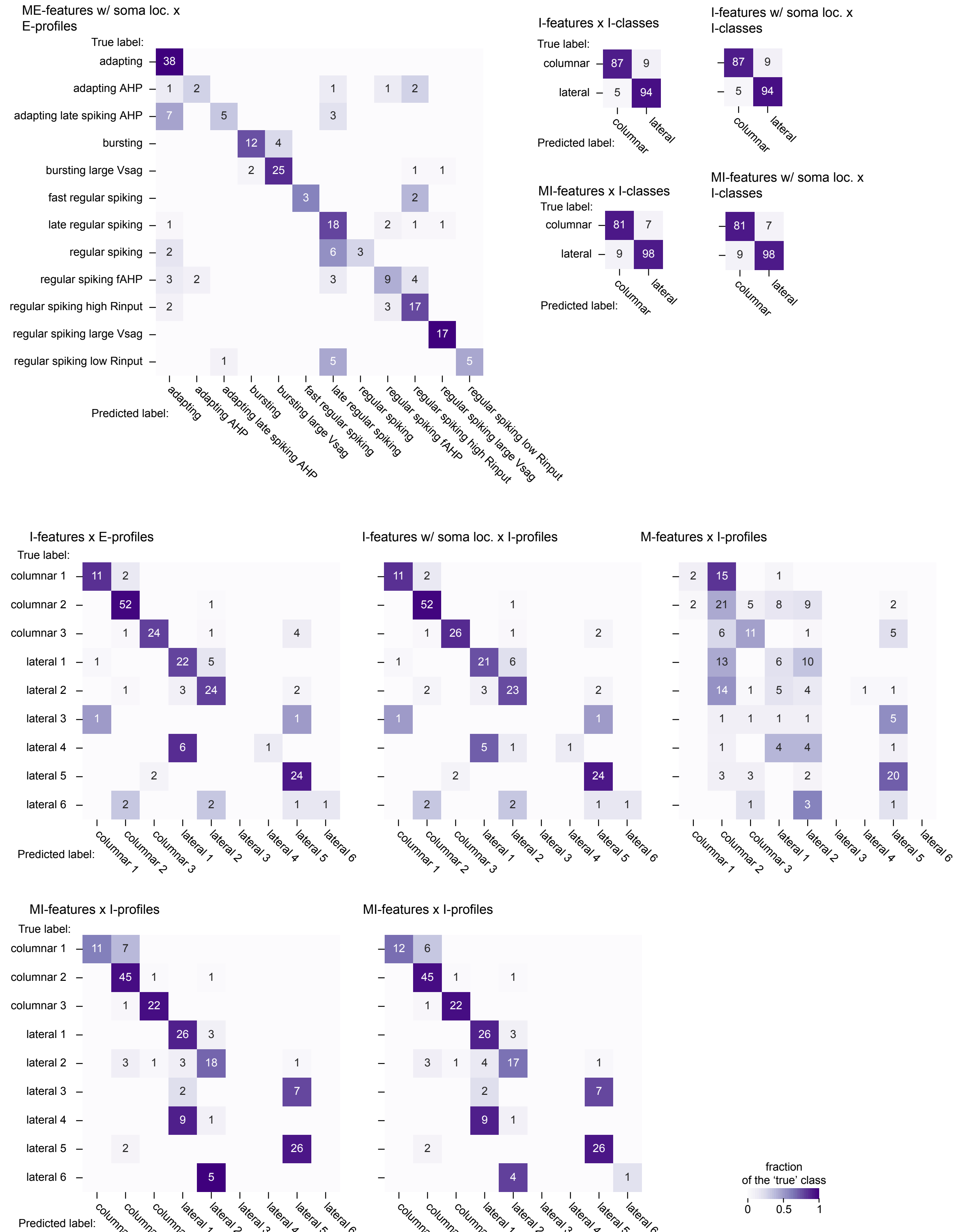
ME-features w/ soma loc. x E-profiles
True label:
adapting
adapting AHP
adapting late spiking AHP
bursting
bursting large Vsag
fast regular spiking
late regular spiking
regular spiking
regular spiking fAHP
regular spiking high Rinput
regular spiking large Vsag
regular spiking low Rinput
Predicted label:
I-features x I-classes
I-features w/ soma loc. x I-classes
MI-features x I-classes
MI-features w/ soma loc. x I-classes
columnar
lateral
I-features x E-profiles
I-features w/ soma loc. x I-profiles
M-features x I-profiles
MI-features x I-profiles
MI-features x I-profiles
columnar 1
columnar 2
columnar 3
lateral 1
lateral 2
lateral 3
lateral 4
lateral 5
lateral 6
fraction
of the 'true' class
0
0.5
1

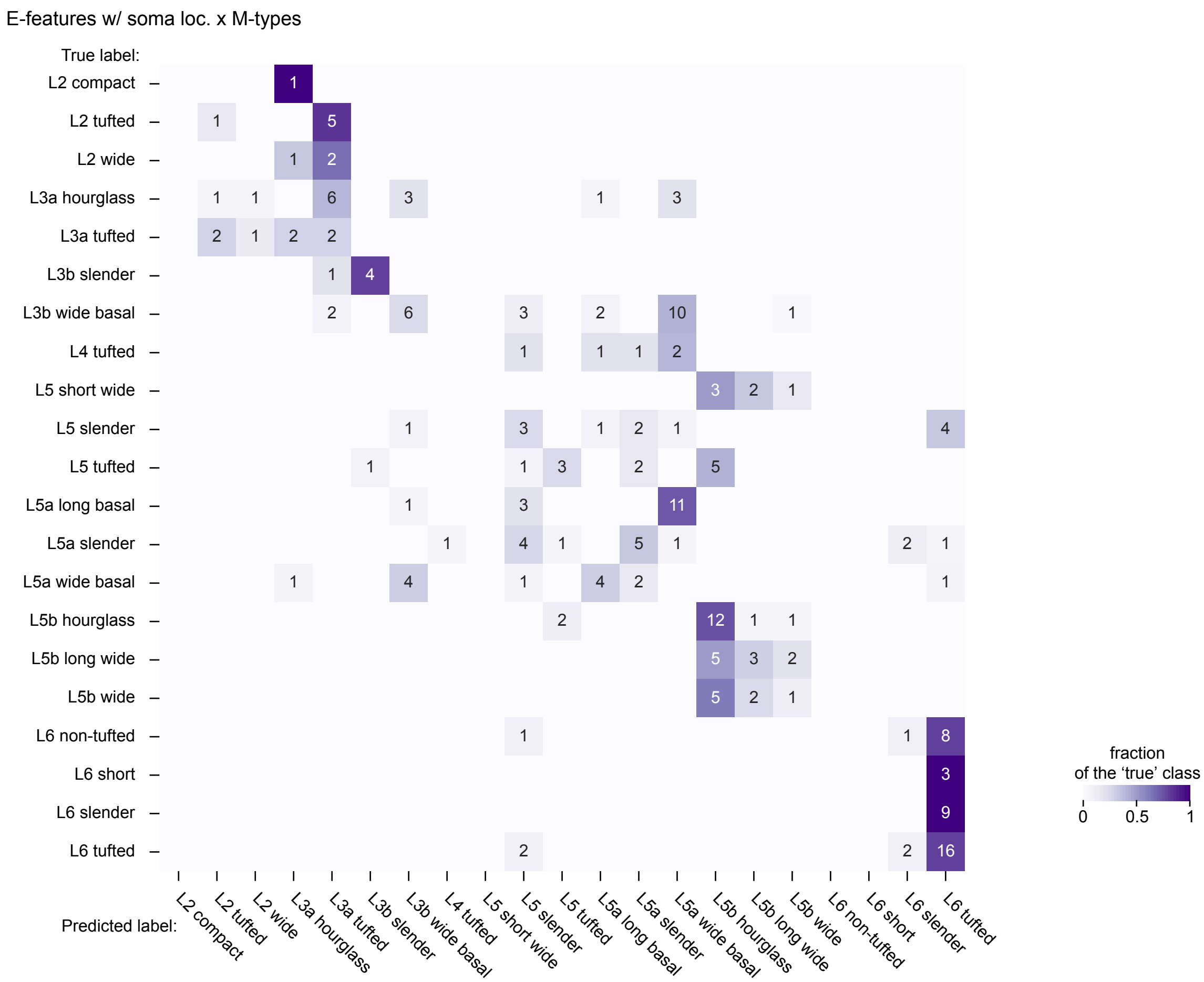
E-features w/ soma loc. x M-types
True label:
L2 compact
L2 tufted
L2 wide
L3a hourglass
L3a tufted
L3b slender
L3b wide basal
L4 tufted
L5 short wide
L5 slender
L5 tufted
L5a long basal
L5a slender
L5a wide basal
L5b hourglass
L5b long wide
L5b wide
L6 non-tufted
L6 short
L6 slender
L6 tufted
Predicted label:
fraction
of the 'true' class
0
0.5
1

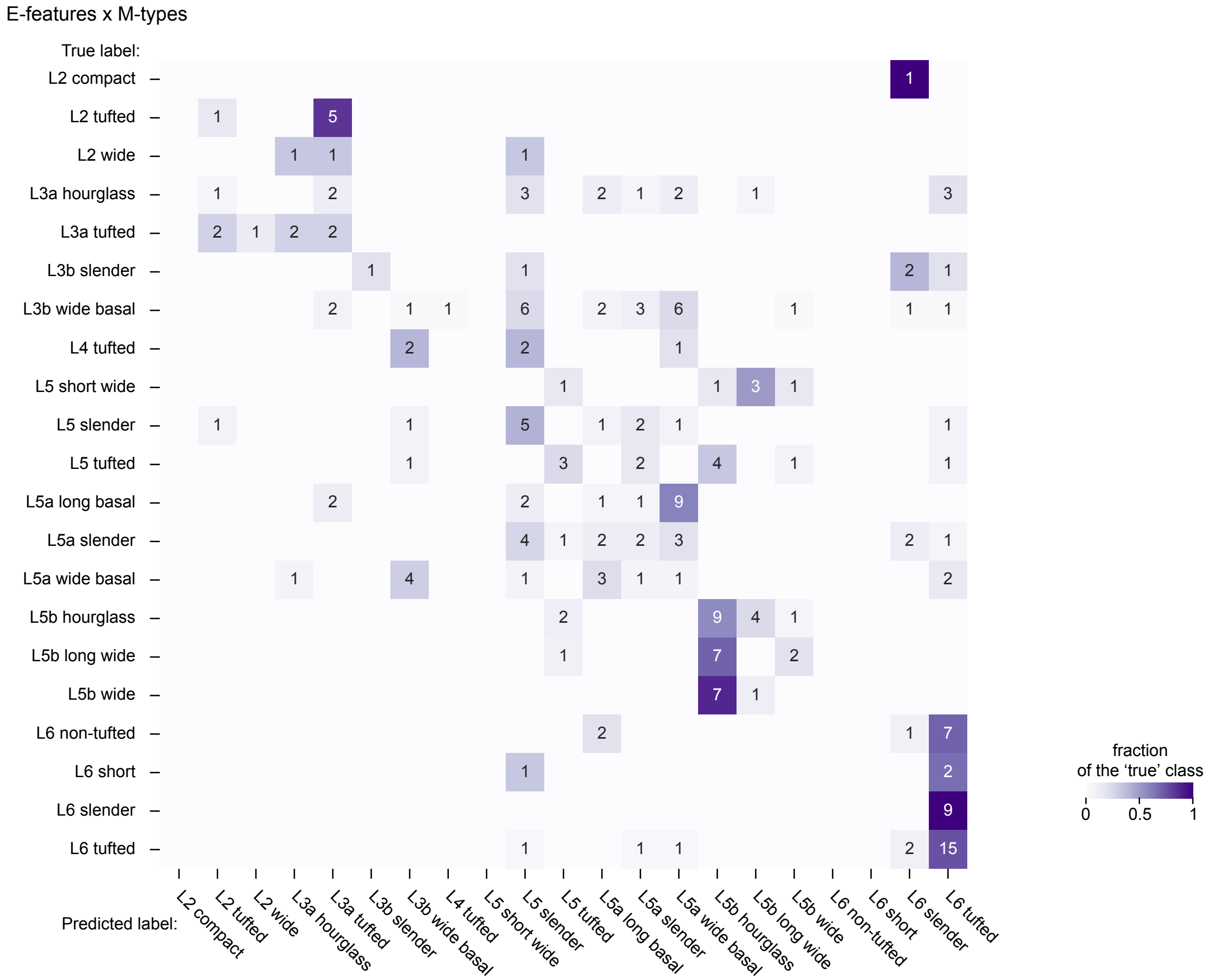
E-features x M-types
True label:
L2 compact
L2 tufted
L2 wide
L3a hourglass
L3a tufted
L3b slender
L3b wide basal
L4 tufted
L5 short wide
L5 slender
L5 tufted
L5a long basal
L5a slender
L5a wide basal
L5b hourglass
L5b long wide
L5b wide
L6 non-tufted
L6 short
L6 slender
L6 tufted
Predicted label:
fraction
of the 'true' class
0
0.5
1

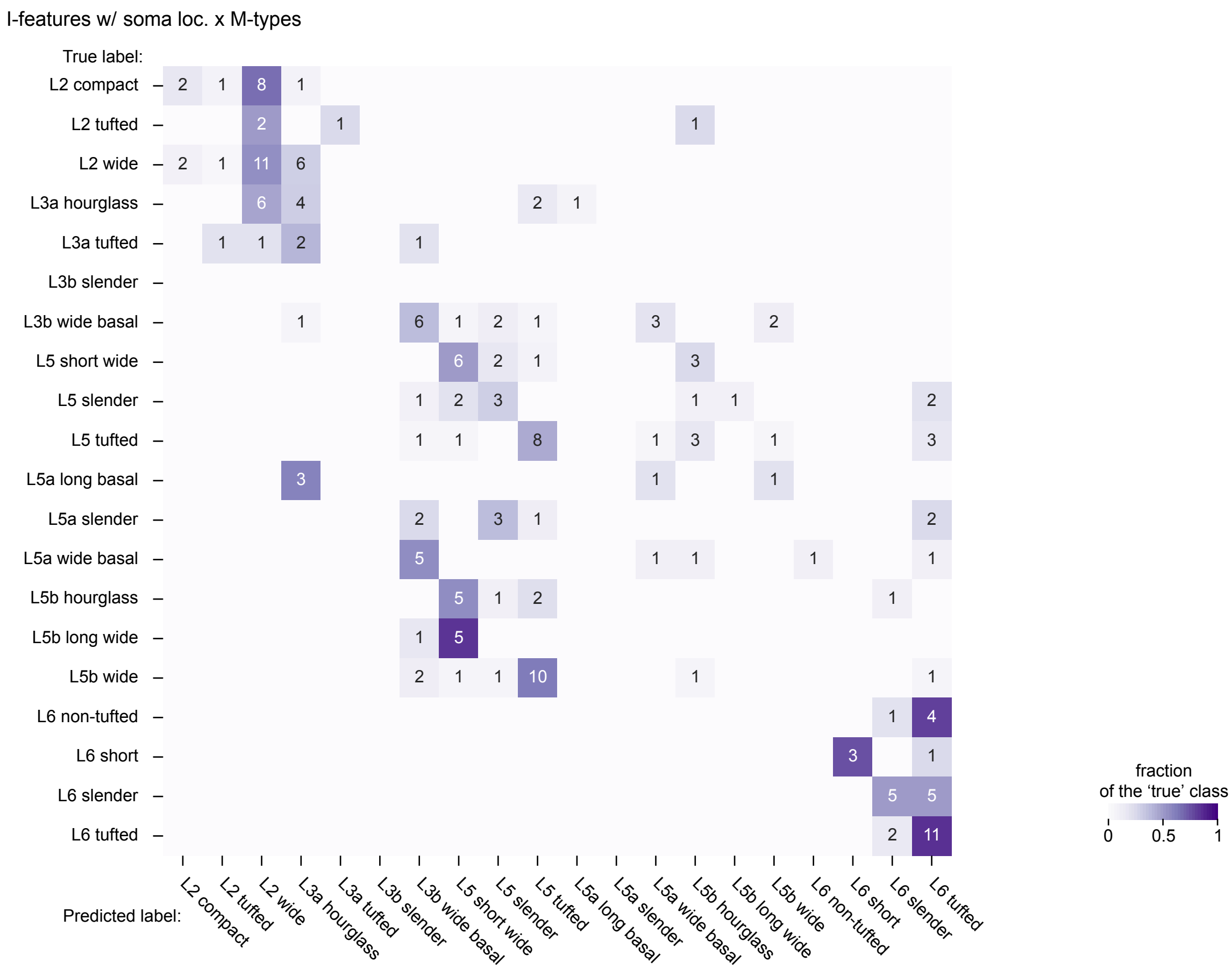
I-features w/ soma loc. x M-types
True label:
L2 compact
L2 tufted
L2 wide
L3a hourglass
L3a tufted
L3b slender
L3b wide basal
L5 short wide
L5 slender
L5 tufted
L5a long basal
L5a slender
L5a wide basal
L5b hourglass
L5b long wide
L5b wide
L6 non-tufted
L6 short
L6 slender
L6 tufted
Predicted label:
fraction
of the 'true' class
0
0.5
1

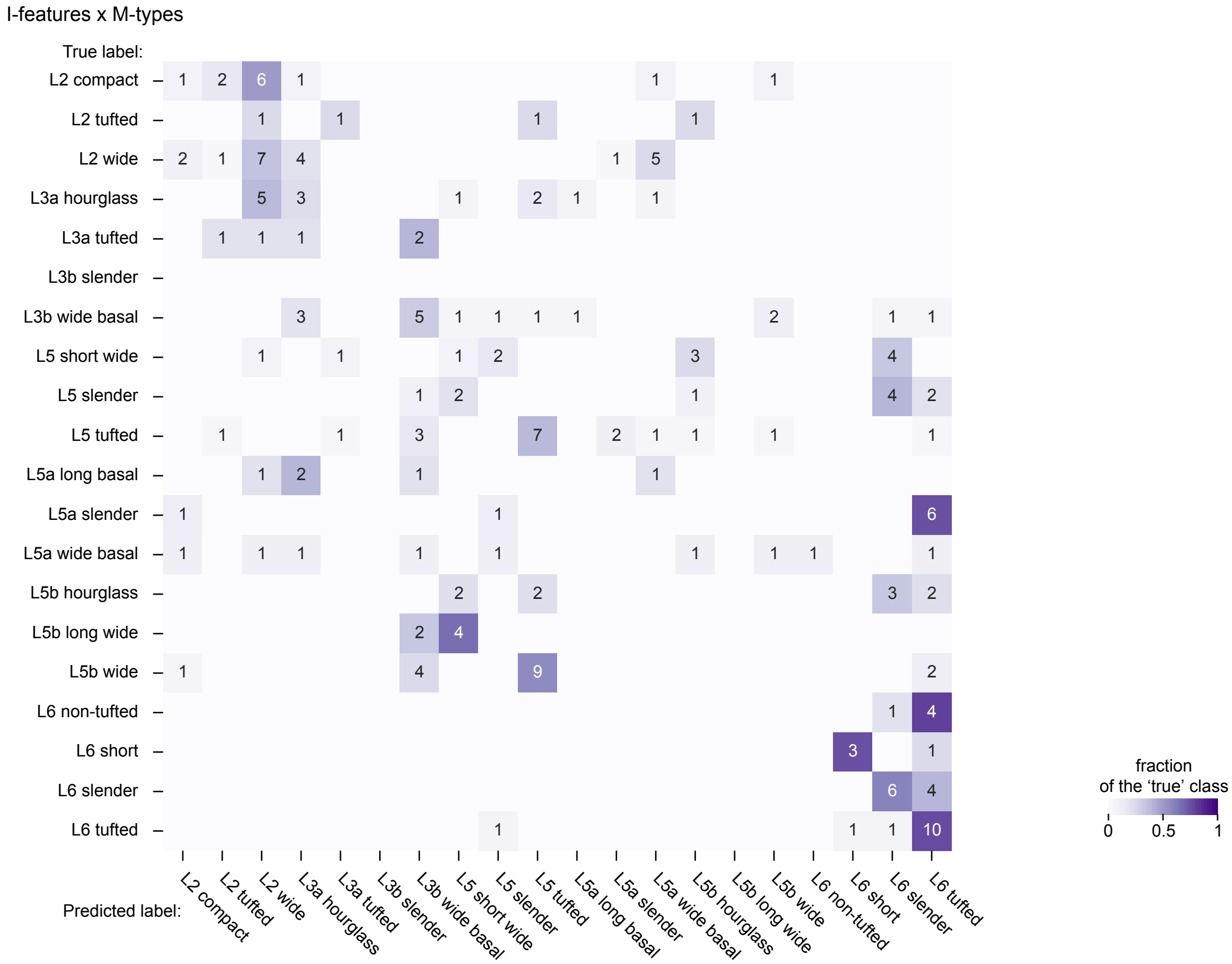
I-features x M-types
True label:
L2 compact
L2 tufted
L2 wide
L3a hourglass
L3a tufted
L3b slender
L3b wide basal
L5 short wide
L5 slender
L5 tufted
L5a long basal
L5a slender
L5a wide basal
L5b hourglass
L5b long wide
L5b wide
L6 non-tufted
L6 short
L6 slender
L6 tufted
Predicted label:
fraction
of the 'true' class
0
0.5
1

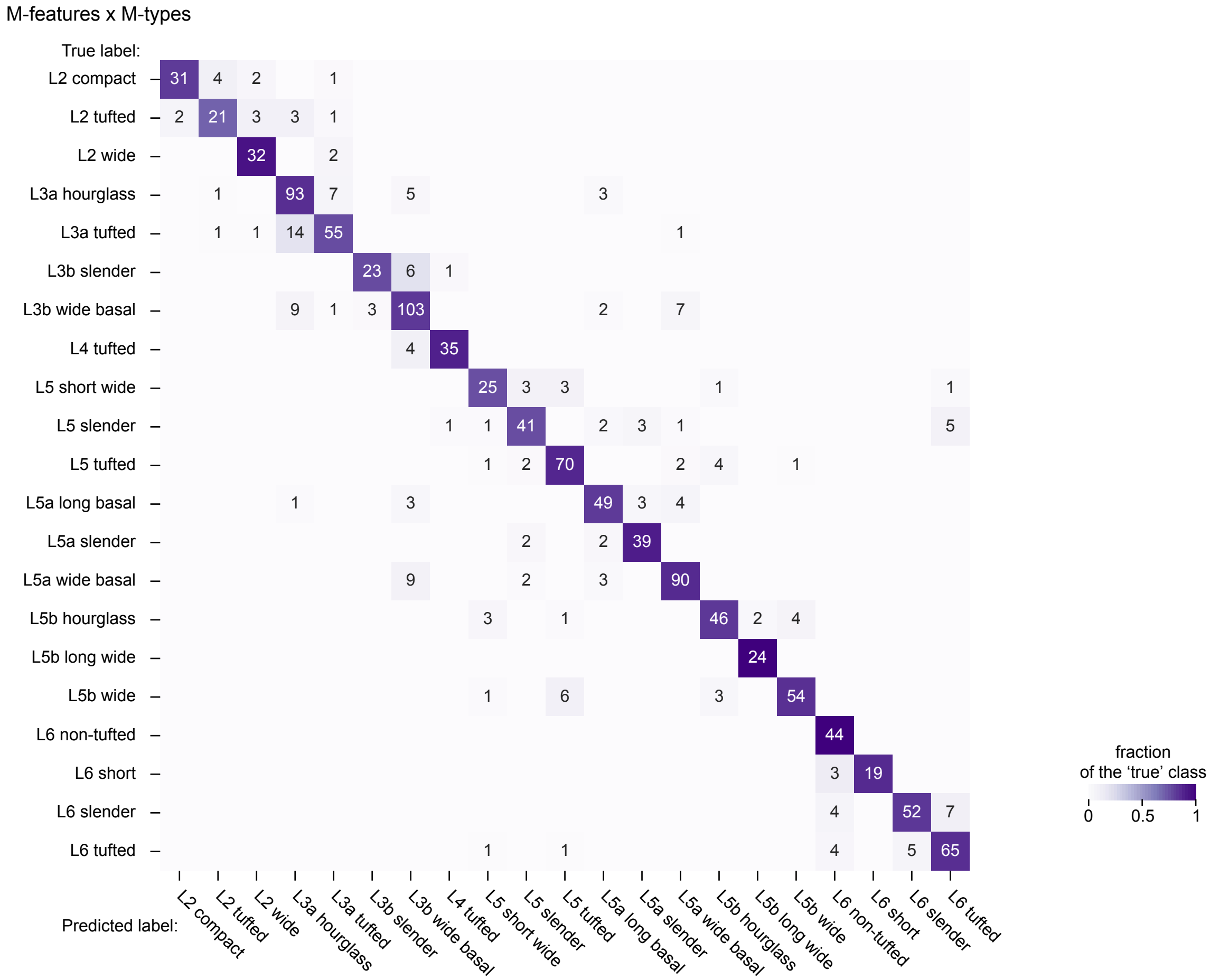
M-features x M-types
True label:
L2 compact
L2 tufted
L2 wide
L3a hourglass
L3a tufted
L3b slender
L3b wide basal
L4 tufted
L5 short wide
L5 slender
L5 tufted
L5a long basal
L5a slender
L5a wide basal
L5b hourglass
L5b long wide
L5b wide
L6 non-tufted
L6 short
L6 slender
L6 tufted
Predicted label:
fraction
of the 'true' class
0
0.5
1

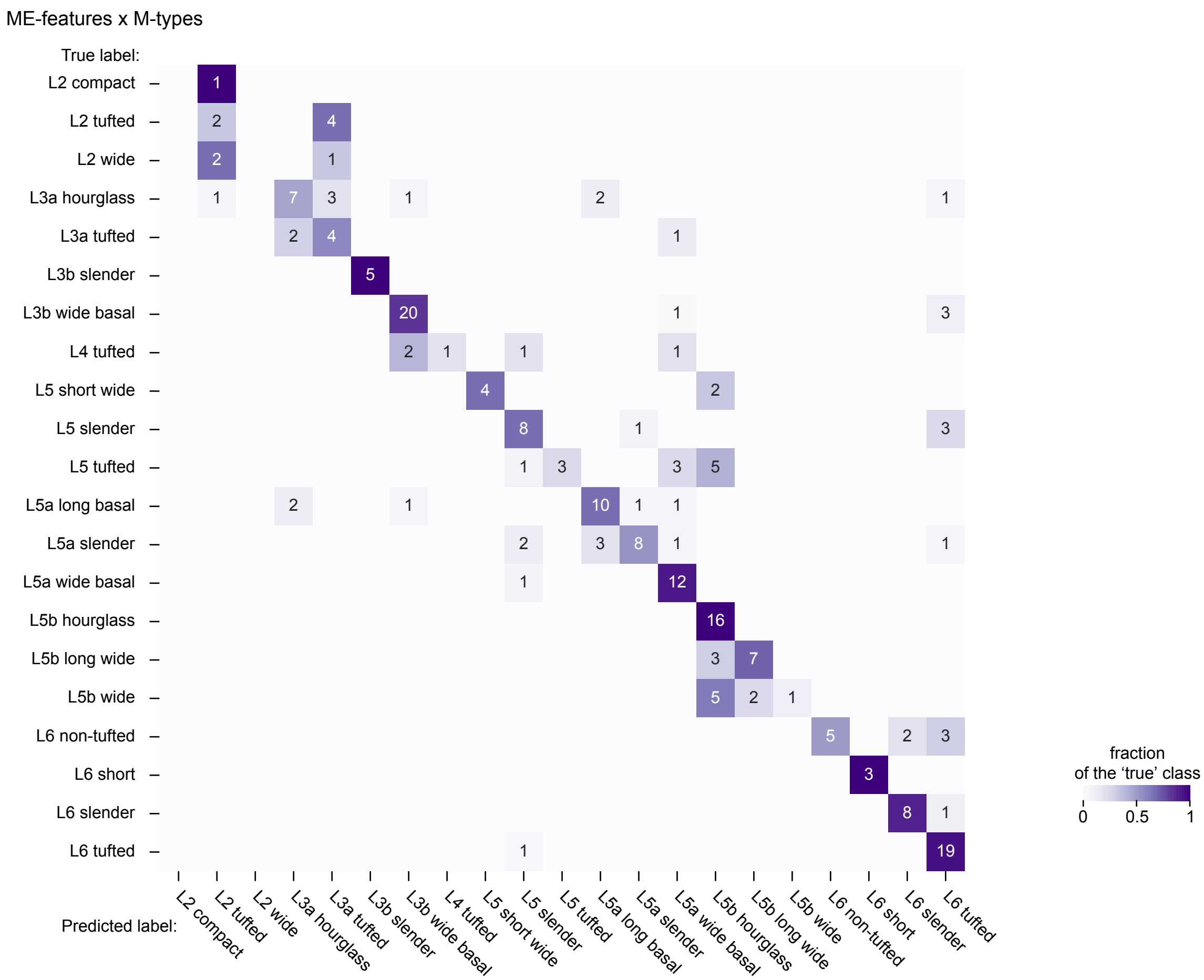
ME-features x M-types
True label:
L2 compact
L2 tufted
L2 wide
L3a hourglass
L3a tufted
L3b slender
L3b wide basal
L4 tufted
L5 short wide
L5 slender
L5 tufted
L5a long basal
L5a slender
L5a wide basal
L5b hourglass
L5b long wide
L5b wide
L6 non-tufted
L6 short
L6 slender
L6 tufted
Predicted label:
fraction
of the 'true' class
0
0.5
1

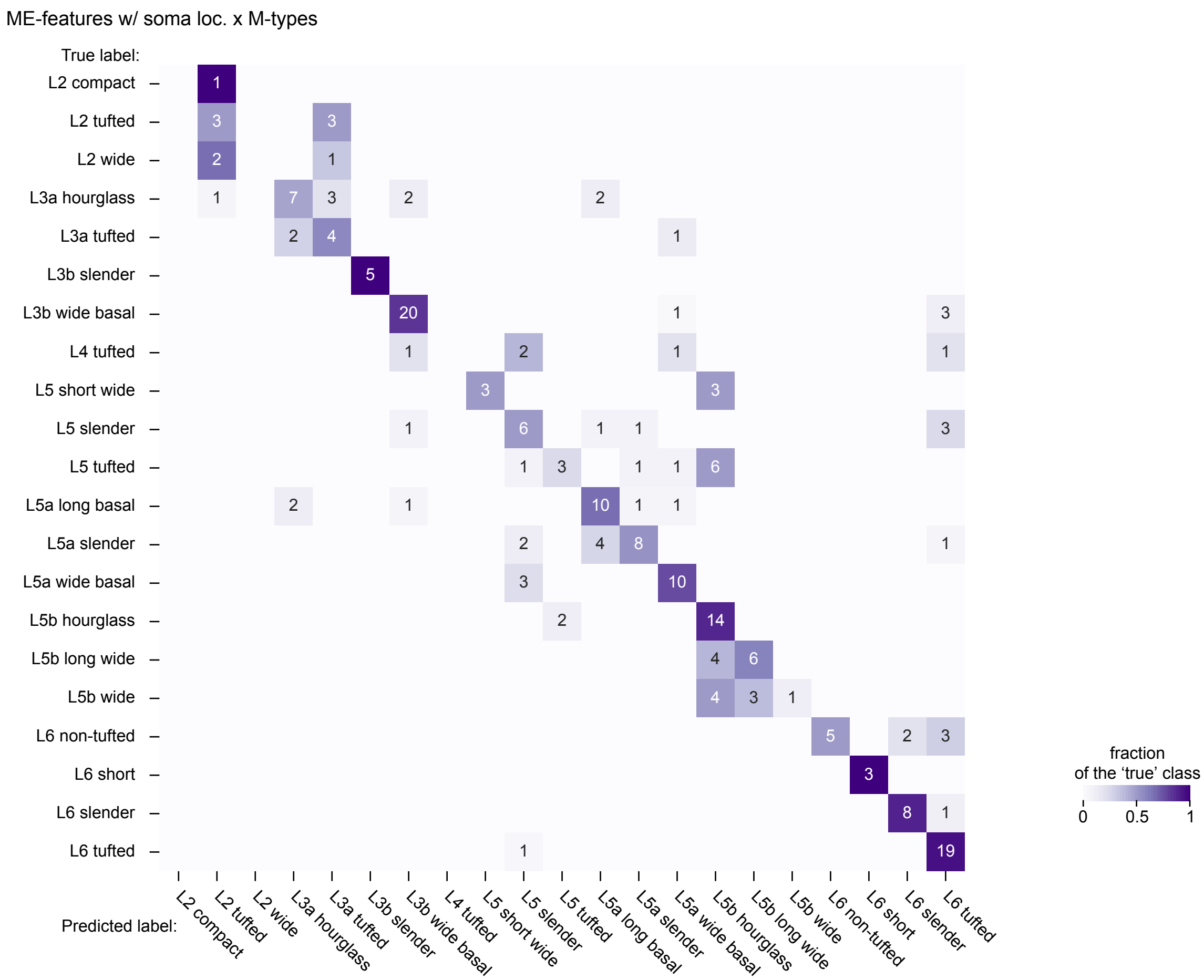
ME-features w/ soma loc. x M-types
True label:
Predicted label:
L2 compact
L2 tufted
L2 wide
L3a hourglass
L3a tufted
L3b slender
L3b wide basal
L4 tufted
L5 short wide
L5 slender
L5 tufted
L5a long basal
L5a slender
L5a wide basal
L5b hourglass
L5b long wide
L5b wide
L6 non-tufted
L6 short
L6 slender
L6 tufted
fraction
of the 'true' class
0
0.5
1

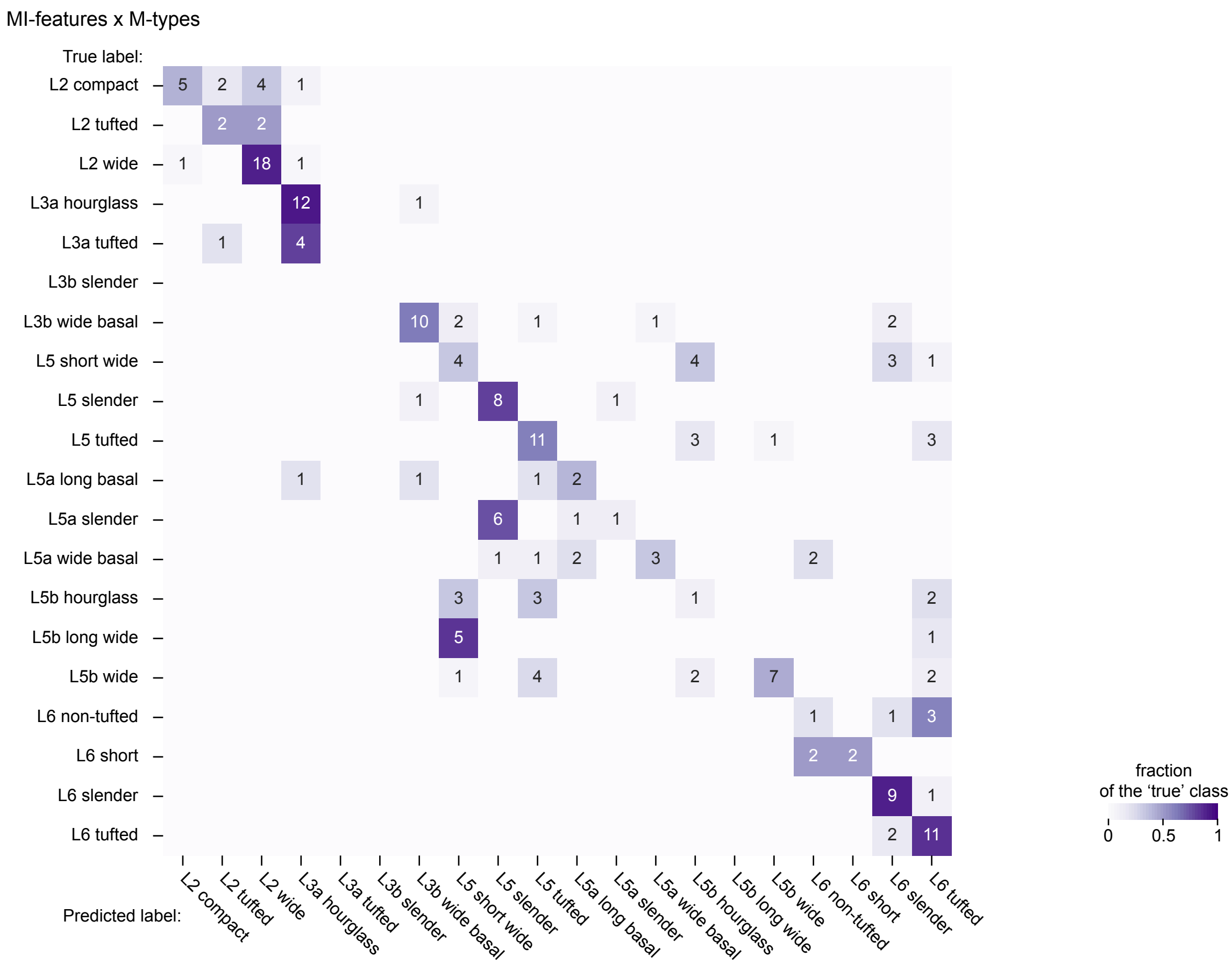
Ml-features x M-types
True label:
L2 compact
L2 tufted
L2 wide
L3a hourglass
L3a tufted
L3b slender
L3b wide basal
L5 short wide
L5 slender
L5 tufted
L5a long basal
L5a slender
L5a wide basal
L5b hourglass
L5b long wide
L5b wide
L6 non-tufted
L6 short
L6 slender
L6 tufted
Predicted label:
fraction
of the ‘true’ class
0
0.5
1

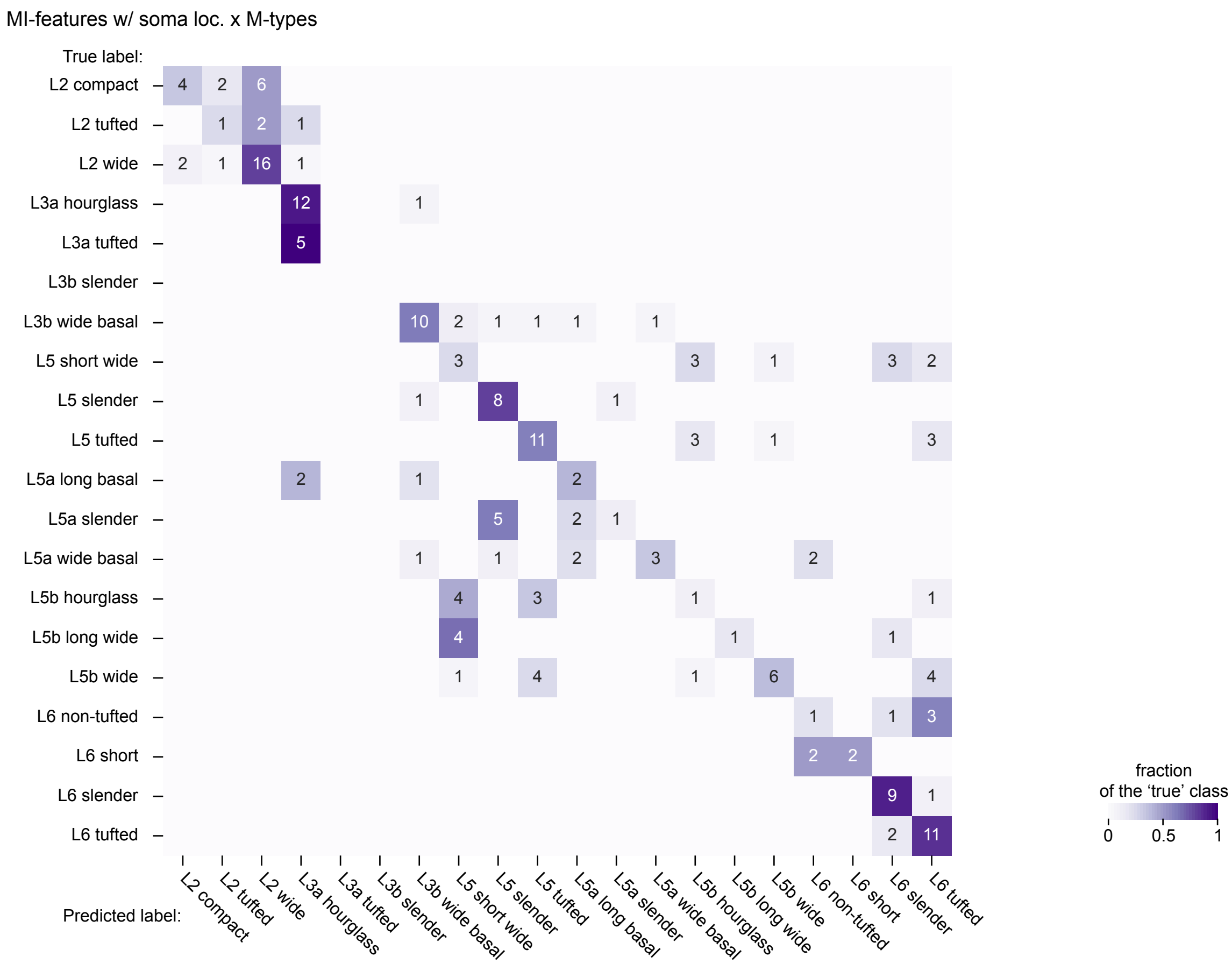
MI-features w/ soma loc. x M-types
True label:
L2 compact
L2 tufted
L2 wide
L3a hourglass
L3a tufted
L3b slender
L3b wide basal
L5 short wide
L5 slender
L5 tufted
L5a long basal
L5a slender
L5a wide basal
L5b hourglass
L5b long wide
L5b wide
L6 non-tufted
L6 short
L6 slender
L6 tufted
Predicted label:
fraction
of the 'true' class
0
0.5
1

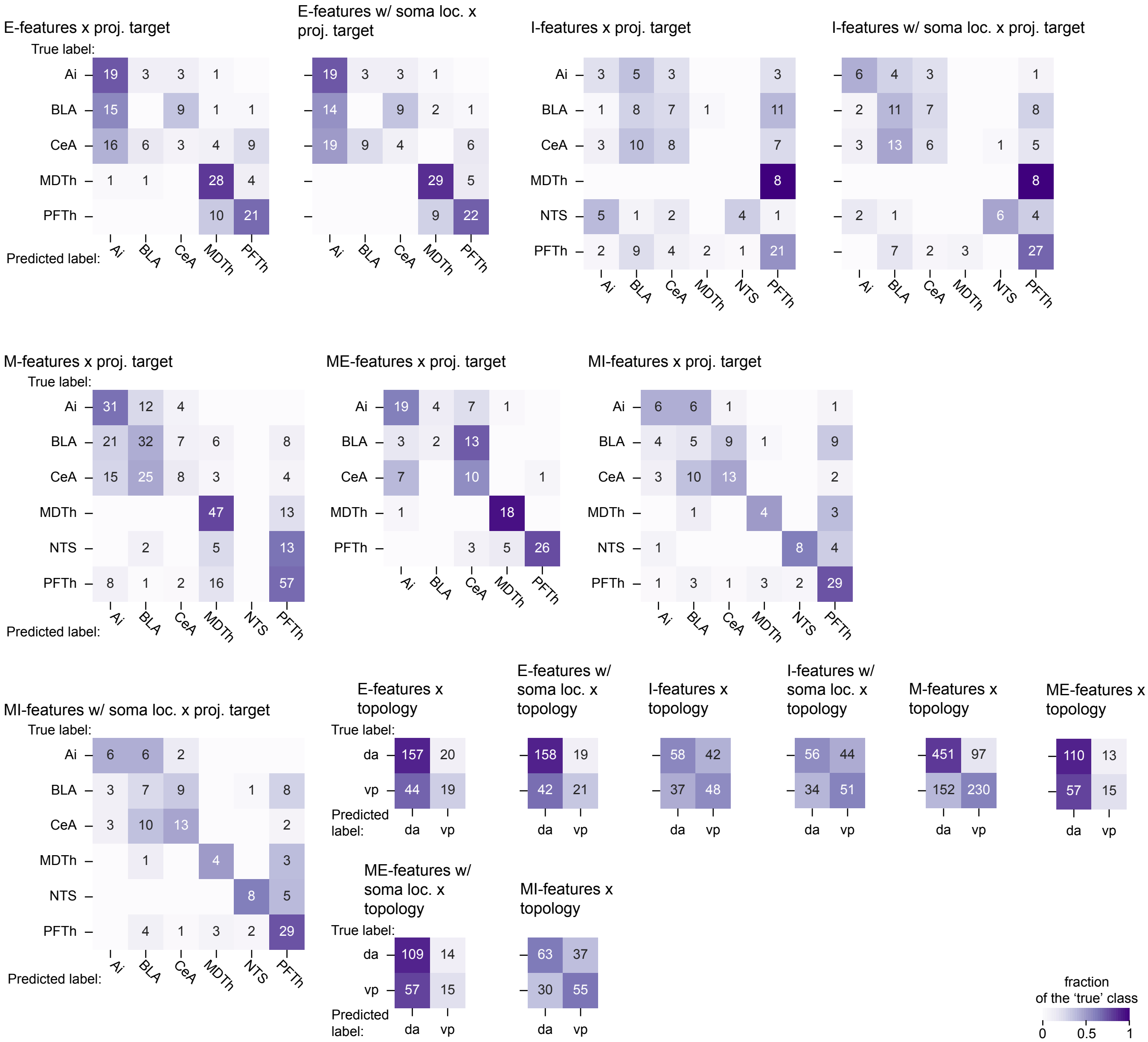

E-features x proj. target
E-features w/ soma loc. x proj. target
I-features x proj. target
I-features w/ soma loc. x proj. target
M-features x proj. target
ME-features x proj. target
MI-features x proj. target
MI-features w/ soma loc. x proj. target
E-features x topology
E-features w/ soma loc. x topology
I-features x topology
I-features w/ soma loc. x topology
M-features x topology
ME-features x topology
ME-features w/ soma loc. x topology
MI-features x topology
True label:
Predicted label:
Ai
BLA
CeA
MDTh
NTS
PFTh
da
vp
fraction
of the 'true' class
0
0.5
1

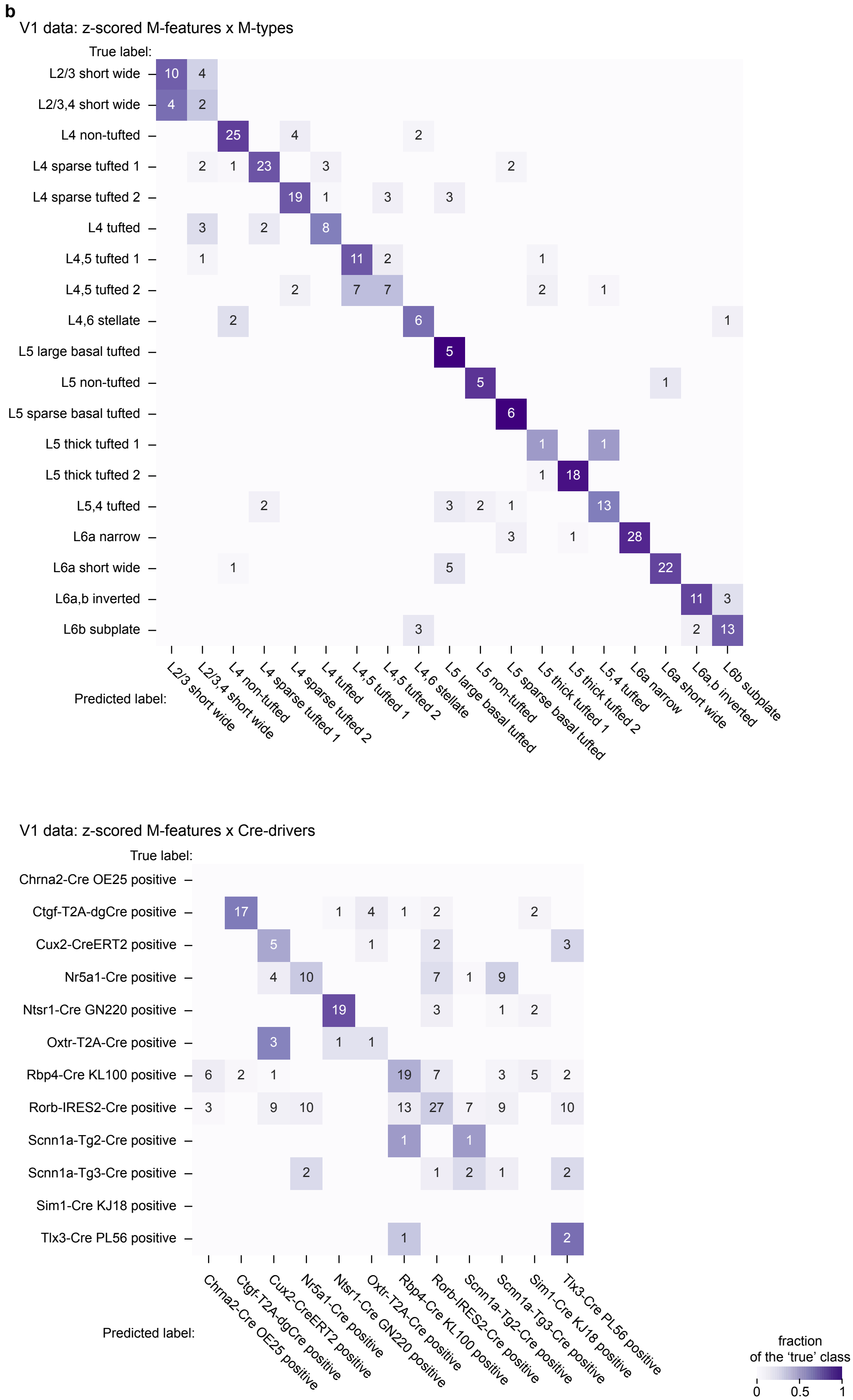
b
V1 data: z-scored M-features x M-types
True label:
L2/3 short wide
L2/3,4 short wide
L4 non-tufted
L4 sparse tufted 1
L4 sparse tufted 2
L4 tufted
L4,5 tufted 1
L4,5 tufted 2
L4,6 stellate
L5 large basal tufted
L5 non-tufted
L5 sparse basal tufted
L5 thick tufted 1
L5 thick tufted 2
L5,4 tufted
L6a narrow
L6a short wide
L6a,b inverted
L6b subplate
Predicted label:
V1 data: z-scored M-features x Cre-drivers
True label:
Chrna2-Cre OE25 positive
Ctgf-T2A-dgCre positive
Cux2-CreERT2 positive
Nr5a1-Cre positive
Ntsr1-Cre GN220 positive
Oxtr-T2A-Cre positive
Rbp4-Cre KL100 positive
Rorb-IRES2-Cre positive
Scnn1a-Tg2-Cre positive
Scnn1a-Tg3-Cre positive
Sim1-Cre KJ18 positive
Tlx3-Cre PL56 positive
Predicted label:
fraction
of the 'true' class
0
0.5
1

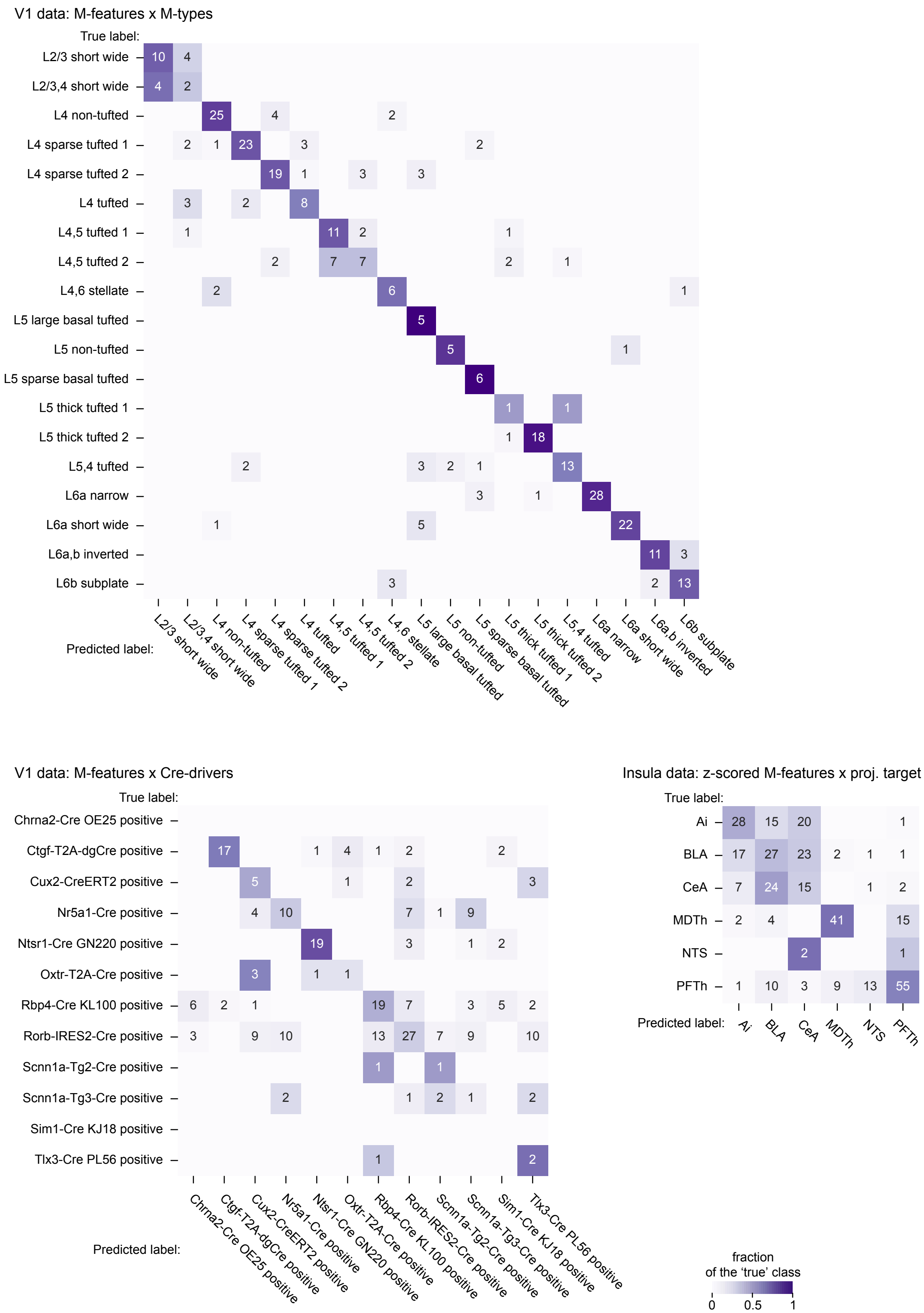
V1 data: M-features x M-types
True label:
L2/3 short wide
L2/3,4 short wide
L4 non-tufted
L4 sparse tufted 1
L4 sparse tufted 2
L4 tufted
L4,5 tufted 1
L4,5 tufted 2
L4,6 stellate
L5 large basal tufted
L5 non-tufted
L5 sparse basal tufted
L5 thick tufted 1
L5 thick tufted 2
L5,4 tufted
L6a narrow
L6a short wide
L6a,b inverted
L6b subplate
Predicted label:
V1 data: M-features x Cre-drivers
Chrna2-Cre OE25 positive
Ctgf-T2A-dgCre positive
Cux2-CreERT2 positive
Nr5a1-Cre positive
Ntsr1-Cre GN220 positive
Oxtr-T2A-Cre positive
Rbp4-Cre KL100 positive
Rorb-IRES2-Cre positive
Scnn1a-Tg2-Cre positive
Scnn1a-Tg3-Cre positive
Sim1-Cre KJ18 positive
Tlx3-Cre PL56 positive
Insula data: z-scored M-features x proj. target
Ai
BLA
CeA
MDTh
NTS
PFTh
fraction
of the 'true' class
0
0.5
1

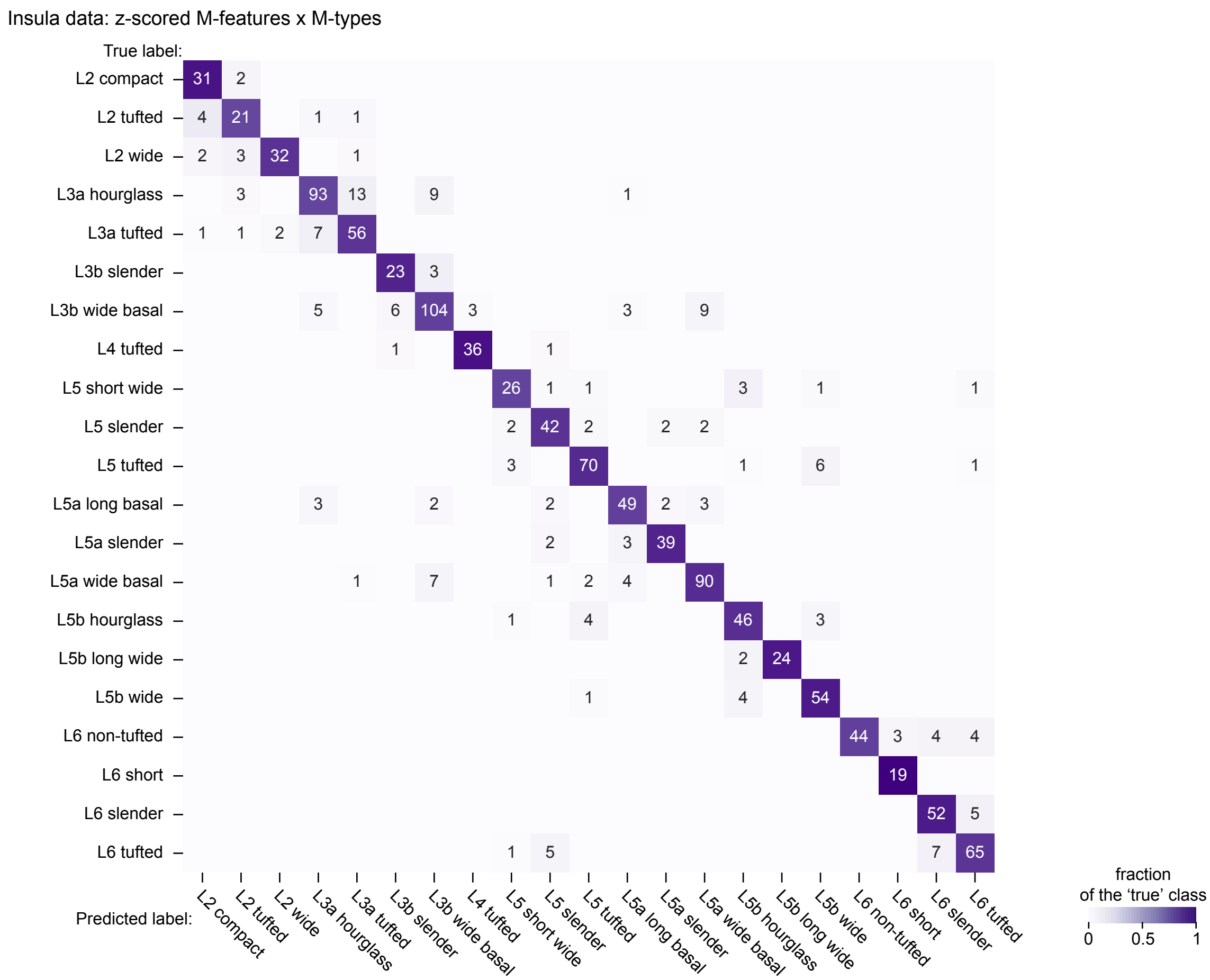

Supplementary Fig. 7: Confusion matrices of trained random forest classifiers. **a**, Performances of classifiers trained on untransformed insular data. Subpanel order matches order of comparisons shown in Fig. 7a. **b**, Performances of classifiers trained on z-score transformed V1 data, untransformed V1 data, and z-score transformed insula data (V1 data from Gouwens et al.[33]). For (**a** and **b**), number of observations displayed in purple-hued boxes were accumulated over five independent instances of classifier training for each type of classifier, e.g., to predict electric (E)-class based on E-features (see Methods). For example, in the case of E-class predicted by E-features, n = 77 cells per classifier instance accumulated to a total of n = 385 cells displayed in the confusion matrix. The performance of each classifier instance was assessed with the withheld dataset (25%). Purple luminance: percentage of neurons correctly predicted by classifiers for a given type, class, projection target, or subregion. da: dorsoanterior; vp: ventroposterior; BLA: basolateral

amygdala; CeA: central amygdala; contra AI: contralateral agranular insula; MD: mediodorsal thalamus; NTS: nucleus tractus solitarius; PF/PO: parafascicular/posterior thalamus; loc.: location; proj.: projection.

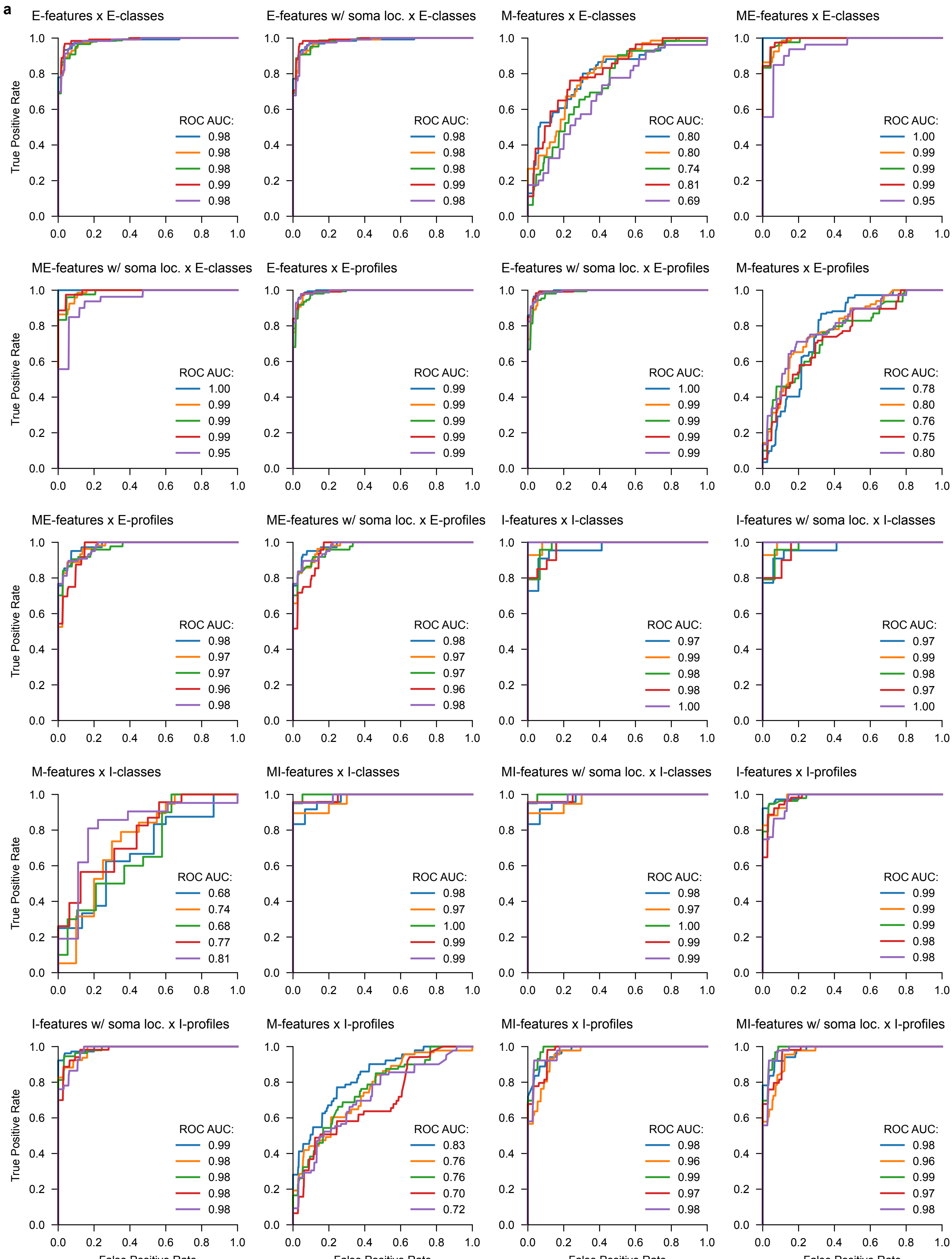
a
E-features x E-classes
ROC AUC: 0.98 0.98 0.98 0.99 0.98
E-features w/ soma loc. x E-classes
ROC AUC: 0.98 0.98 0.98 0.99 0.98
M-features x E-classes
ROC AUC: 0.80 0.80 0.74 0.81 0.69
ME-features x E-classes
ROC AUC: 1.00 0.99 0.99 0.99 0.95
ME-features w/ soma loc. x E-classes
ROC AUC: 1.00 0.99 0.99 0.99 0.95
E-features x E-profiles
ROC AUC: 0.99 0.99 0.99 0.99 0.99
E-features w/ soma loc. x E-profiles
ROC AUC: 1.00 0.99 0.99 0.99 0.99
M-features x E-profiles
ROC AUC: 0.78 0.80 0.76 0.75 0.80
ME-features x E-profiles
ROC AUC: 0.98 0.97 0.97 0.96 0.98
ME-features w/ soma loc. x E-profiles
ROC AUC: 0.98 0.97 0.97 0.96 0.98
I-features x I-classes
ROC AUC: 0.97 0.99 0.98 0.98 1.00
I-features w/ soma loc. x I-classes
ROC AUC: 0.97 0.99 0.98 0.97 1.00
M-features x I-classes
ROC AUC: 0.68 0.74 0.68 0.77 0.81
MI-features x I-classes
ROC AUC: 0.98 0.97 1.00 0.99 0.99
MI-features w/ soma loc. x I-classes
ROC AUC: 0.98 0.97 1.00 0.99 0.99
I-features x I-profiles
ROC AUC: 0.99 0.99 0.99 0.98 0.98
I-features w/ soma loc. x I-profiles
ROC AUC: 0.99 0.98 0.98 0.98 0.98
M-features x I-profiles
ROC AUC: 0.83 0.76 0.76 0.70 0.72
MI-features x I-profiles
ROC AUC: 0.98 0.96 0.99 0.97 0.98
MI-features w/ soma loc. x I-profiles
ROC AUC: 0.98 0.96 0.99 0.97 0.98
True Positive Rate
False Positive Rate

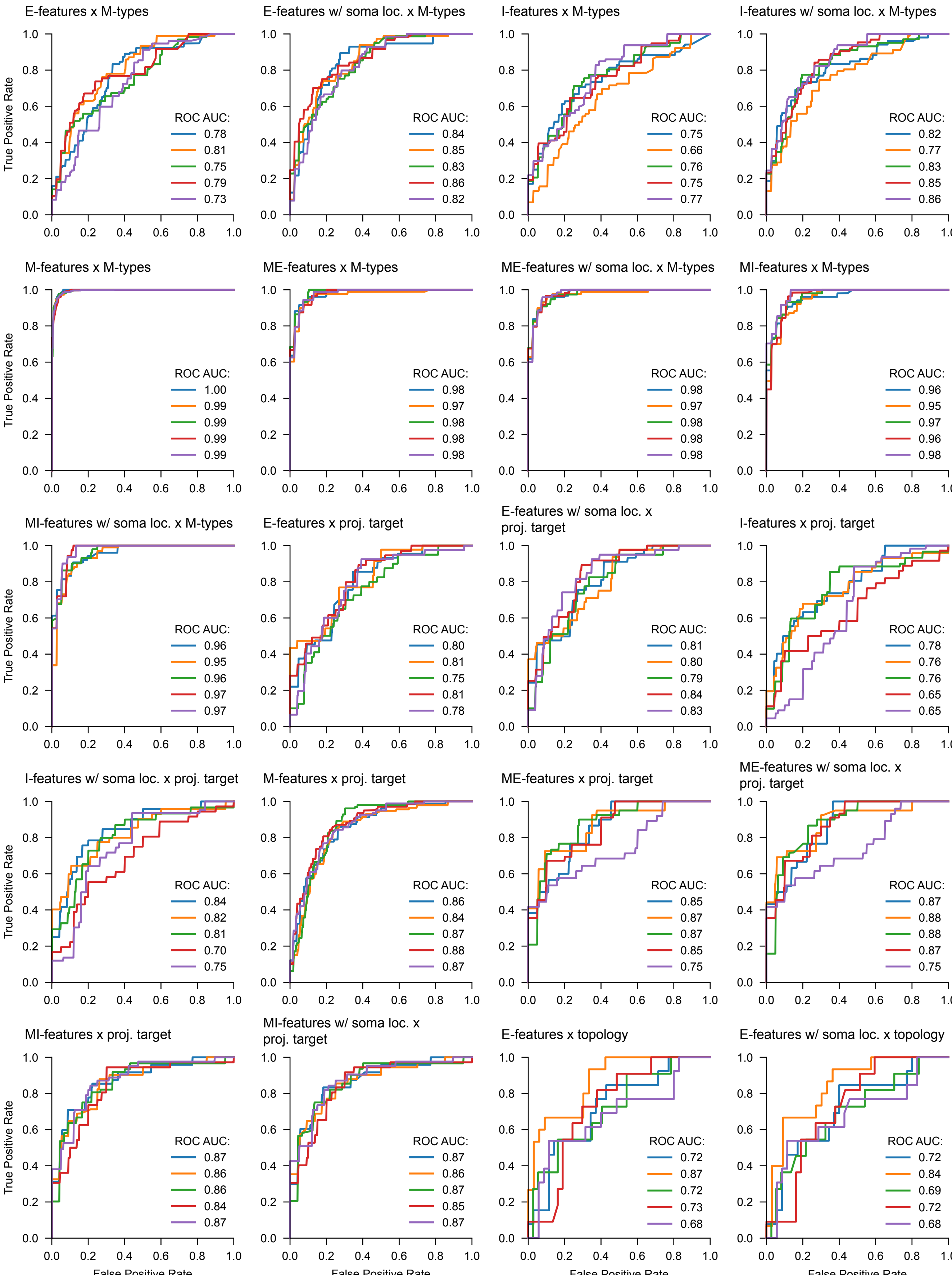
E-features x M-types
ROC AUC:
0.78
0.81
0.75
0.79
0.73
E-features w/ soma loc. x M-types
ROC AUC:
0.84
0.85
0.83
0.86
0.82
I-features x M-types
ROC AUC:
0.75
0.66
0.76
0.75
0.77
I-features w/ soma loc. x M-types
ROC AUC:
0.82
0.77
0.83
0.85
0.86
M-features x M-types
ROC AUC:
1.00
0.99
0.99
0.99
0.99
ME-features x M-types
ROC AUC:
0.98
0.97
0.98
0.98
0.98
ME-features w/ soma loc. x M-types
ROC AUC:
0.98
0.97
0.98
0.98
0.98
MI-features x M-types
ROC AUC:
0.96
0.95
0.97
0.96
0.98
MI-features w/ soma loc. x M-types
ROC AUC:
0.96
0.95
0.96
0.97
0.97
E-features x proj. target
ROC AUC:
0.80
0.81
0.75
0.81
0.78
E-features w/ soma loc. x proj. target
ROC AUC:
0.81
0.80
0.79
0.84
0.83
I-features x proj. target
ROC AUC:
0.78
0.76
0.76
0.65
0.65
I-features w/ soma loc. x proj. target
ROC AUC:
0.84
0.82
0.81
0.70
0.75
M-features x proj. target
ROC AUC:
0.86
0.84
0.87
0.88
0.87
ME-features x proj. target
ROC AUC:
0.85
0.87
0.87
0.85
0.75
ME-features w/ soma loc. x proj. target
ROC AUC:
0.87
0.88
0.88
0.87
0.75
MI-features x proj. target
ROC AUC:
0.87
0.86
0.86
0.84
0.87
MI-features w/ soma loc. x proj. target
ROC AUC:
0.87
0.86
0.87
0.85
0.87
E-features x topology
ROC AUC:
0.72
0.87
0.72
0.73
0.68
E-features w/ soma loc. x topology
ROC AUC:
0.72
0.84
0.69
0.72
0.68
True Positive Rate
False Positive Rate

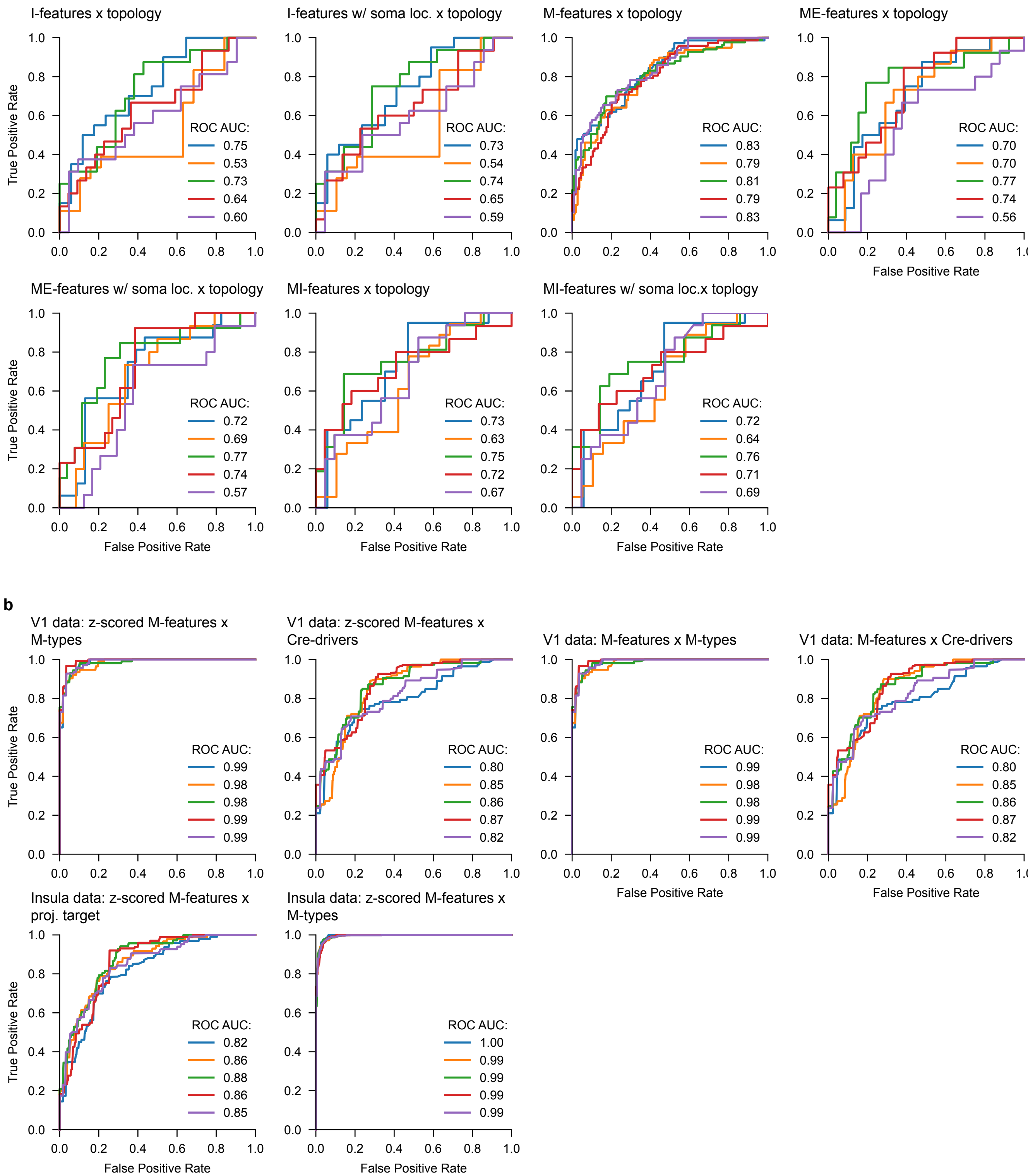

Supplementary Fig. 8: Receiver-operating characteristic (ROC) and total area under the curve (AUC) performances of trained forest classifiers. a and **b**, The ROC and AUC displayed for each independent

instance of classifier training for each type of classifier (using a macro-averaged one-vs.-rest scheme, Methods). **a**, performances of classifiers trained on untransformed insular data. Subpanel order matches order of comparisons shown in Fig. 7a. **b**, performances of classifiers trained on untransformed V1 data, z-score transformed insular, and z-score transformed V1 data (V1 data from Gouwens et al.[33]).

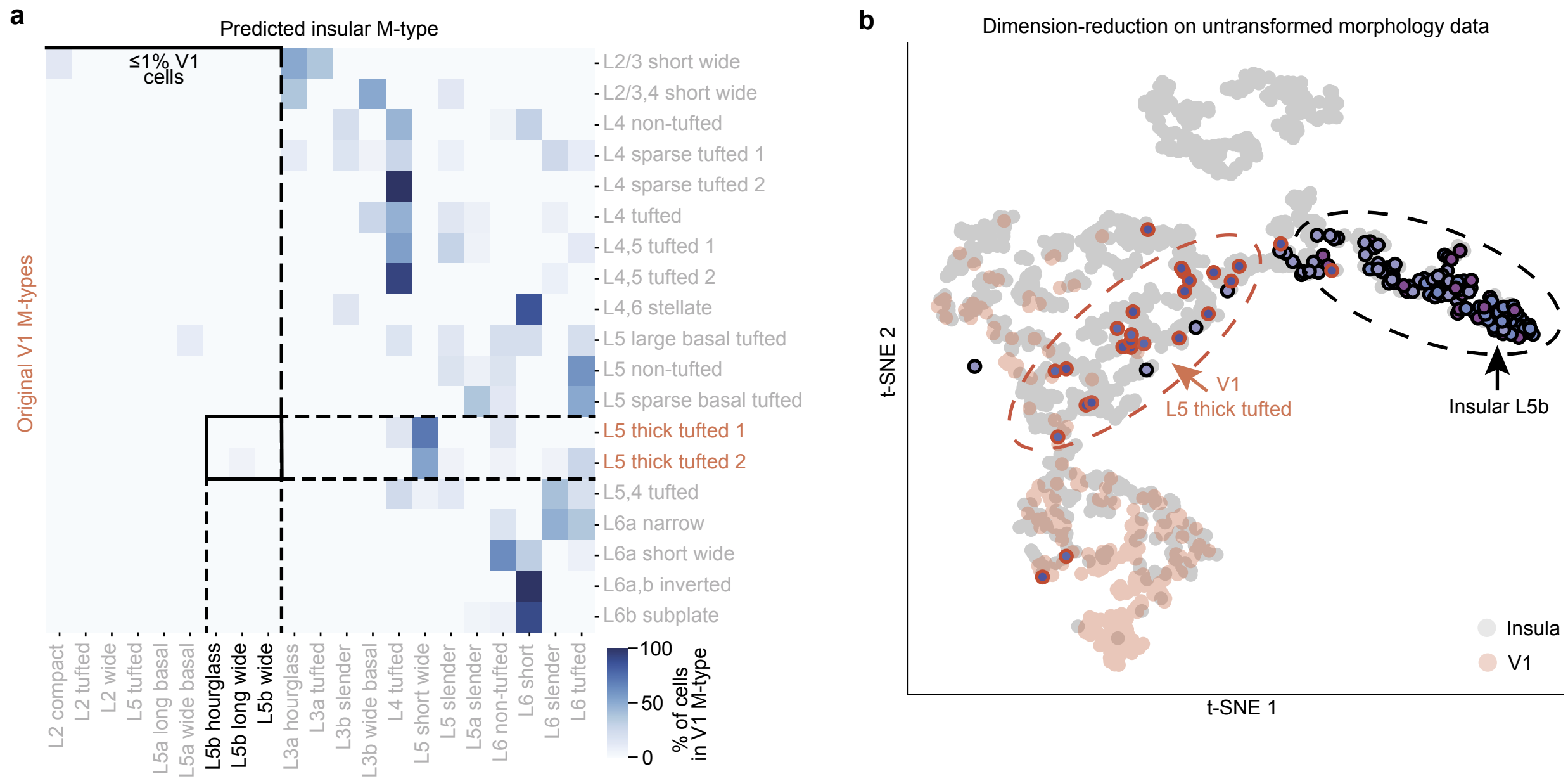

Supplementary Fig. 9: No insular L5b M-type equivalents were found in V1. a, Matching of V1 M-types to insular M-types using classifiers trained on untransformed insular data. The black rectangle highlights the absence of L5b M-type allocations to the largest V1 M-type neurons ('L5 thick tufted 1' and 'L5 thick tufted 2'). Blue luminance: percentage of cells from a given V1 M-type that are allocated to an insular M-type by the classifier. **b**, t-SNE visualization of the untransformed morphology data from both the insular (grey filled-circles) and V1 (red filled-circles) datasets. The largest M-types from both datasets are primarily located in non-overlapping regions of the t-SNE plot (red dashed ellipse: V1 L5 thick tufted M-types; black dashed ellipse: insular L5b M-types). V1 data from Gouwens et al.[33]

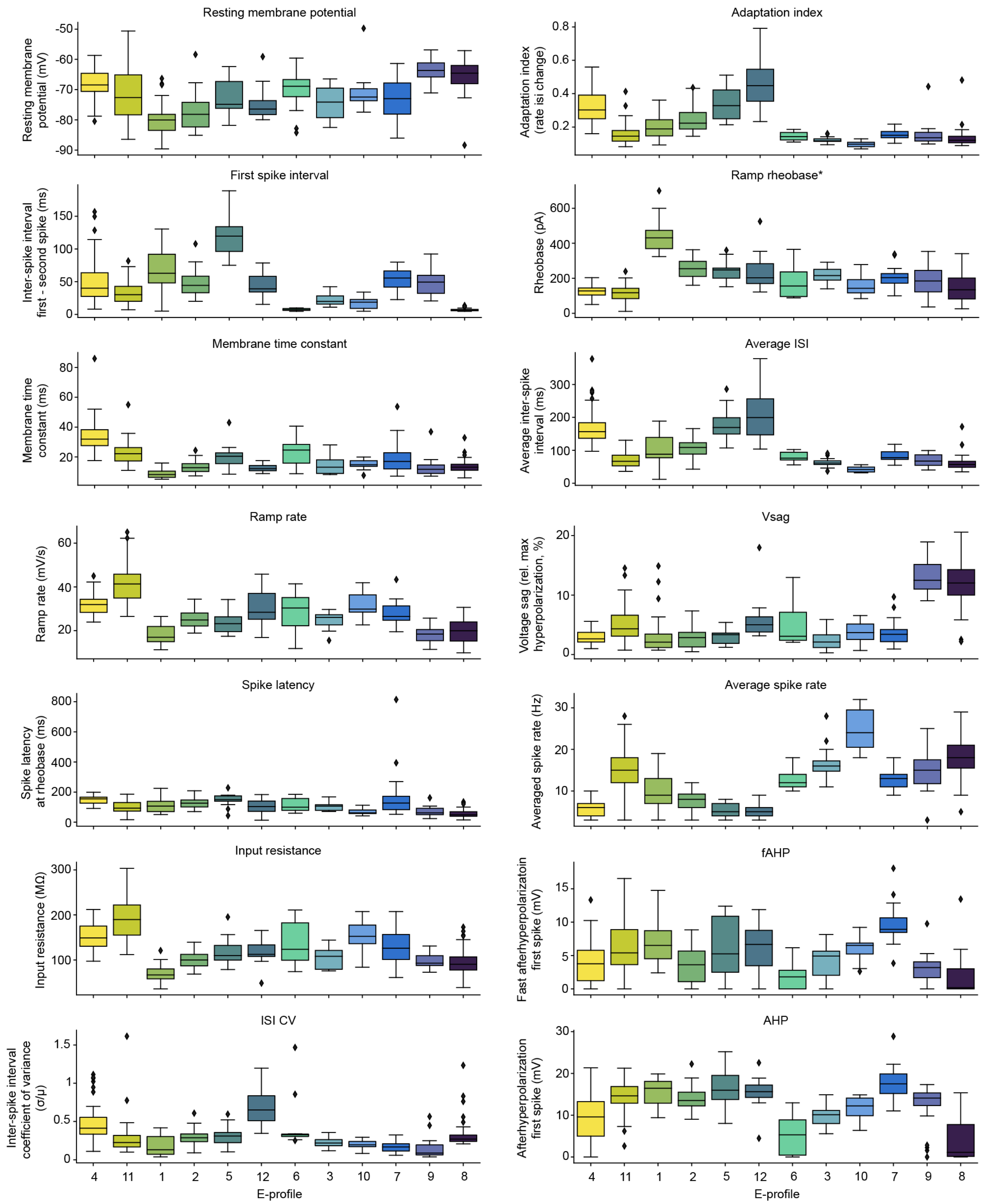
Resting membrane potential
Resting membrane potential (mV)
Adaptation index
Adaptation index (rate isi change)
First spike interval
Inter-spike interval first - second spike (ms)
Ramp rheobase*
Rheobase (pA)
Membrane time constant
Membrane time constant (ms)
Average ISI
Average inter-spike interval (ms)
Ramp rate
Ramp rate (mV/s)
Vsag
Voltage sag (rel. max hyperpolarization, %)
Spike latency
Spike latency at rheobase (ms)
Average spike rate
Averaged spike rate (Hz)
Input resistance
Input resistance (MΩ)
fAHP
Fast afterhyperpolarizatoin first spike (mV)
ISI CV
Inter-spike interval coefficient of variance (σ/μ)
AHP
Afterhyperpolarization first spike (mV)
4 11 1 2 5 12 6 3 10 7 9 8
E-profile

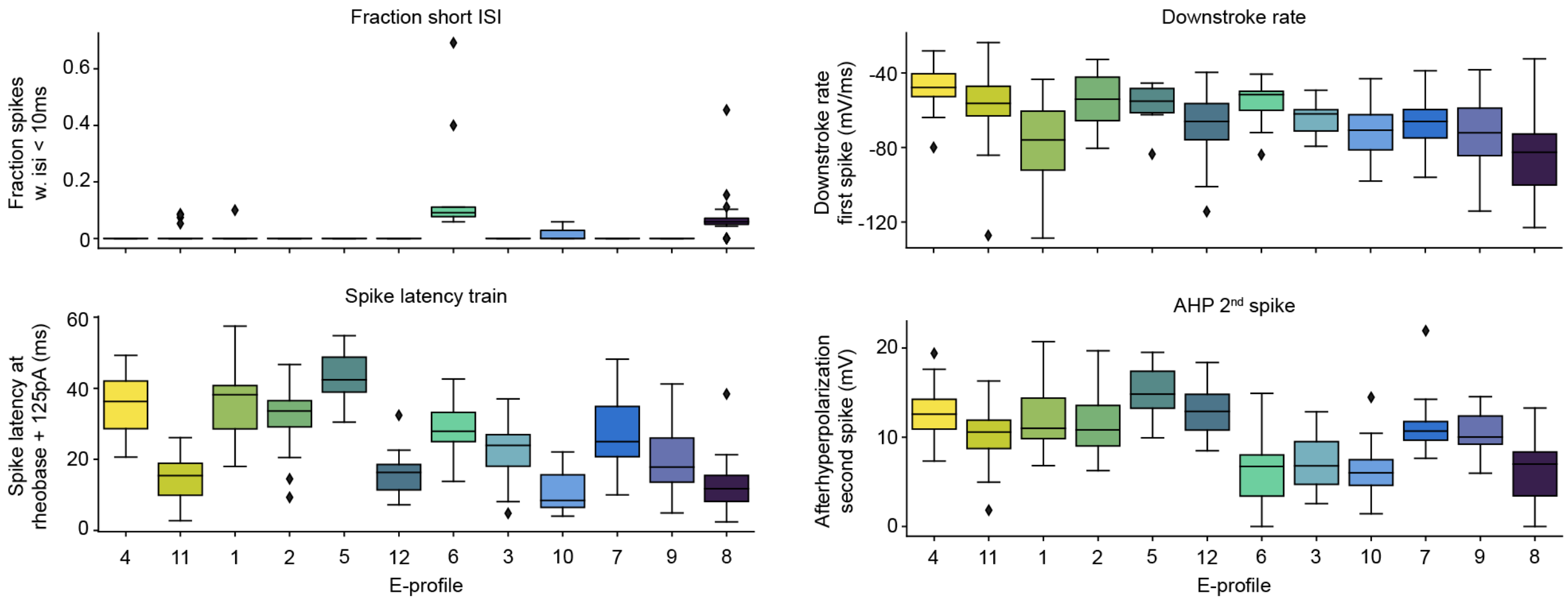

Supplementary Fig. 10: Distinct features of E-profiles. All 16 electrical features used for the iCCA, as well as resting membrane potential and adaptation index. *: Current required to elicit the first action potential during ramping depolarizing current injection. Boxplots are sorted in sequence as shown in the dendrogram from Fig. 4a. Number of observations (n) for all groups provided in Supplementary Table 14. Boxplots use the following convention: center line, median; box limits, upper and lower quartiles; whiskers, 1.5x interquartile range; diamonds, outliers.

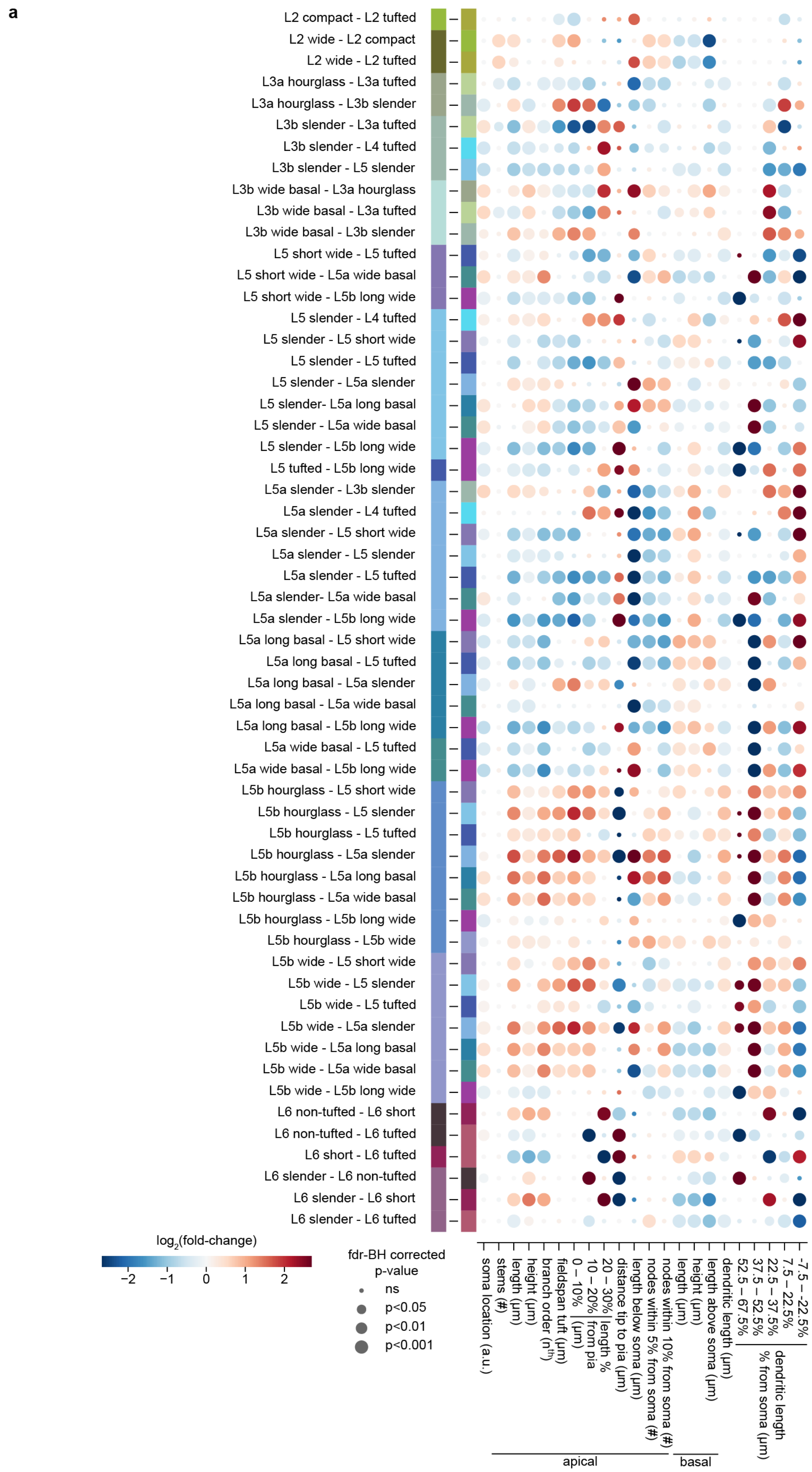
a
L2 compact - L2 tufted
L2 wide - L2 compact
L2 wide - L2 tufted
L3a hourglass - L3a tufted
L3a hourglass - L3b slender
L3b slender - L3a tufted
L3b slender - L4 tufted
L3b slender - L5 slender
L3b wide basal - L3a hourglass
L3b wide basal - L3a tufted
L3b wide basal - L3b slender
L5 short wide - L5 tufted
L5 short wide - L5a wide basal
L5 short wide - L5b long wide
L5 slender - L4 tufted
L5 slender - L5 short wide
L5 slender - L5 tufted
L5 slender - L5a slender
L5 slender- L5a long basal
L5 slender - L5a wide basal
L5 slender - L5b long wide
L5 tufted - L5b long wide
L5a slender - L3b slender
L5a slender - L4 tufted
L5a slender - L5 short wide
L5a slender - L5 slender
L5a slender - L5 tufted
L5a slender- L5a wide basal
L5a slender - L5b long wide
L5a long basal - L5 short wide
L5a long basal - L5 tufted
L5a long basal - L5a slender
L5a long basal - L5a wide basal
L5a long basal - L5b long wide
L5a wide basal - L5 tufted
L5a wide basal - L5b long wide
L5b hourglass - L5 short wide
L5b hourglass - L5 slender
L5b hourglass - L5 tufted
L5b hourglass - L5a slender
L5b hourglass - L5a long basal
L5b hourglass - L5a wide basal
L5b hourglass - L5b long wide
L5b hourglass - L5b wide
L5b wide - L5 short wide
L5b wide - L5 slender
L5b wide - L5 tufted
L5b wide - L5a slender
L5b wide - L5a long basal
L5b wide - L5a wide basal
L5b wide - L5b long wide
L6 non-tufted - L6 short
L6 non-tufted - L6 tufted
L6 short - L6 tufted
L6 slender - L6 non-tufted
L6 slender - L6 short
L6 slender - L6 tufted
log2(fold-change)
−2 −1 0 1 2
fdr-BH corrected p-value
ns
p<0.05
p<0.01
p<0.001
soma location (a.u.)
stems (#)
length (µm)
height (µm)
branch order (nth)
fieldspan tuft (µm)
0 – 10%
10 – 20%
20 – 30%
length % from pia (µm)
distance tip to pia (µm)
length below soma (µm)
nodes within 5% from soma (#)
nodes within 10% from soma (#)
length (µm)
height (µm)
length above soma (µm)
dendritic length (µm)
52.5 – 67.5%
37.5 – 52.5%
22.5 – 37.5%
7.5 – 22.5%
-7.5 – -22.5%
dendritic length % from soma (µm)
apical
basal

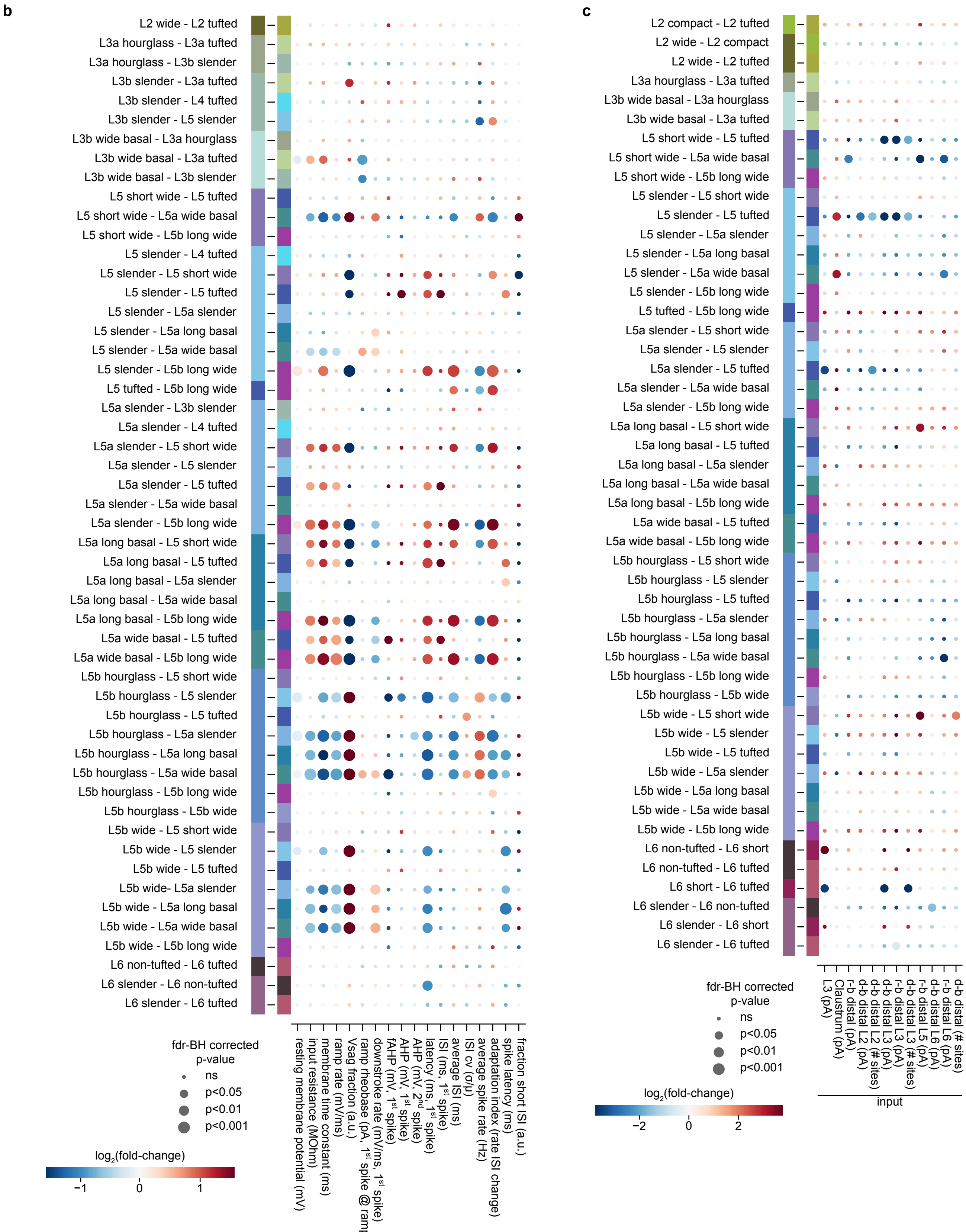
b
L2 wide - L2 tufted
L3a hourglass - L3a tufted
L3a hourglass - L3b slender
L3b slender - L3a tufted
L3b slender - L4 tufted
L3b slender - L5 slender
L3b wide basal - L3a hourglass
L3b wide basal - L3a tufted
L3b wide basal - L3b slender
L5 short wide - L5 tufted
L5 short wide - L5a wide basal
L5 short wide - L5b long wide
L5 slender - L4 tufted
L5 slender - L5 short wide
L5 slender - L5 tufted
L5 slender - L5a slender
L5 slender - L5a long basal
L5 slender - L5a wide basal
L5 slender - L5b long wide
L5 tufted - L5b long wide
L5a slender - L3b slender
L5a slender - L4 tufted
L5a slender - L5 short wide
L5a slender - L5 slender
L5a slender - L5 tufted
L5a slender - L5a wide basal
L5a slender - L5b long wide
L5a long basal - L5 short wide
L5a long basal - L5 tufted
L5a long basal - L5a slender
L5a long basal - L5a wide basal
L5a long basal - L5b long wide
L5a wide basal - L5 tufted
L5a wide basal - L5b long wide
L5b hourglass - L5 short wide
L5b hourglass - L5 slender
L5b hourglass - L5 tufted
L5b hourglass - L5a slender
L5b hourglass - L5a long basal
L5b hourglass - L5a wide basal
L5b hourglass - L5b long wide
L5b hourglass - L5b wide
L5b wide - L5 short wide
L5b wide - L5 slender
L5b wide - L5 tufted
L5b wide- L5a slender
L5b wide - L5a long basal
L5b wide - L5a wide basal
L5b wide - L5b long wide
L6 non-tufted - L6 tufted
L6 slender - L6 non-tufted
L6 slender - L6 tufted
resting membrane potential (mV)
input resistance (MOhm)
membrane time constant (ms)
ramp rate (mV/ms)
Vsag fraction (a.u.)
ramp rheobase (pA, 1st spike @ ramp)
downstroke rate (mV/ms, 1st spike)
fAHP (mV, 1st spike)
AHP (mV, 1st spike)
AHP (mV, 2nd spike)
latency (ms, 1st spike)
ISI (ms, 1st spike)
average ISI (ms)
ISI cv (σ/μ)
average spike rate (Hz)
adaptation index (rate ISI change)
spike latency (ms)
fraction short ISI (a.u.)
fdr-BH corrected p-value
ns
p<0.05
p<0.01
p<0.001
log2(fold-change)
−1
0
1
c
L2 compact - L2 tufted
L2 wide - L2 compact
L2 wide - L2 tufted
L3a hourglass - L3a tufted
L3b wide basal - L3a hourglass
L3b wide basal - L3a tufted
L5 short wide - L5 tufted
L5 short wide - L5a wide basal
L5 short wide - L5b long wide
L5 slender - L5 short wide
L5 slender - L5 tufted
L5 slender - L5a slender
L5 slender - L5a long basal
L5 slender - L5a wide basal
L5 slender - L5b long wide
L5 tufted - L5b long wide
L5a slender - L5 short wide
L5a slender - L5 slender
L5a slender - L5 tufted
L5a slender - L5a wide basal
L5a slender - L5b long wide
L5a long basal - L5 short wide
L5a long basal - L5 tufted
L5a long basal - L5a slender
L5a long basal - L5a wide basal
L5a long basal - L5b long wide
L5a wide basal - L5 tufted
L5a wide basal - L5b long wide
L5b hourglass - L5 short wide
L5b hourglass - L5 slender
L5b hourglass - L5 tufted
L5b hourglass - L5a slender
L5b hourglass - L5a long basal
L5b hourglass - L5a wide basal
L5b hourglass - L5b long wide
L5b hourglass - L5b wide
L5b wide - L5 short wide
L5b wide - L5 slender
L5b wide - L5 tufted
L5b wide - L5a slender
L5b wide - L5a long basal
L5b wide - L5a wide basal
L5b wide - L5b long wide
L6 non-tufted - L6 short
L6 non-tufted - L6 tufted
L6 short - L6 tufted
L6 slender - L6 non-tufted
L6 slender - L6 short
L6 slender - L6 tufted
L3 (pA)
Claustrum (pA)
r-b distal (pA)
d-b distal L2 (pA)
d-b distal L2 (# sites)
d-b distal L3 (pA)
r-b distal L3 (pA)
d-b distal L3 (# sites)
r-b distal L5 (pA)
d-b distal L6 (pA)
r-b distal L6 (pA)
d-b distal (# sites)
input
fdr-BH corrected p-value
ns
p<0.05
p<0.01
p<0.001
log2(fold-change)
−2
0
2

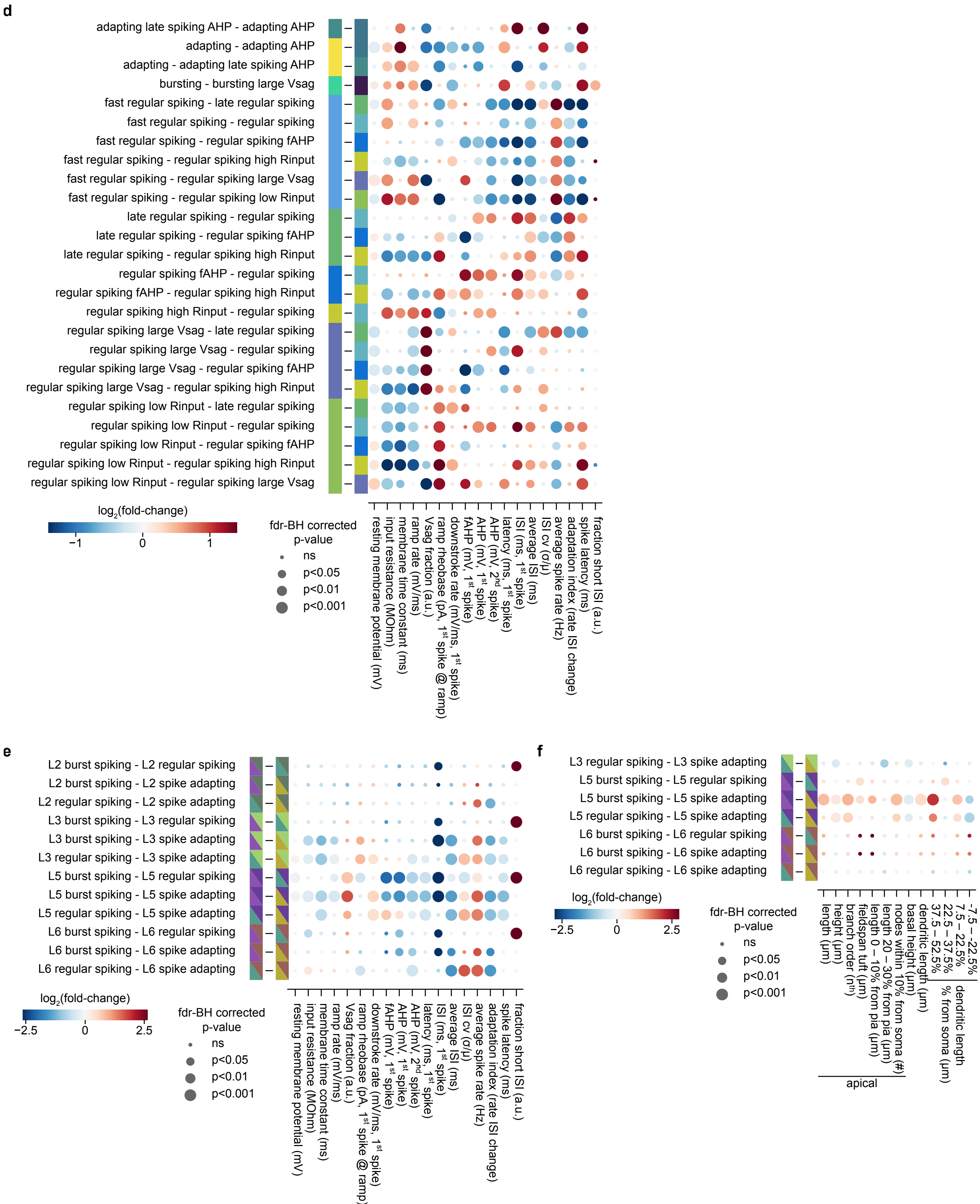
d
adapting late spiking AHP - adapting AHP
adapting - adapting AHP
adapting - adapting late spiking AHP
bursting - bursting large Vsag
fast regular spiking - late regular spiking
fast regular spiking - regular spiking
fast regular spiking - regular spiking fAHP
fast regular spiking - regular spiking high Rinput
fast regular spiking - regular spiking large Vsag
fast regular spiking - regular spiking low Rinput
late regular spiking - regular spiking
late regular spiking - regular spiking fAHP
late regular spiking - regular spiking high Rinput
regular spiking fAHP - regular spiking
regular spiking fAHP - regular spiking high Rinput
regular spiking high Rinput - regular spiking
regular spiking large Vsag - late regular spiking
regular spiking large Vsag - regular spiking
regular spiking large Vsag - regular spiking fAHP
regular spiking large Vsag - regular spiking high Rinput
regular spiking low Rinput - late regular spiking
regular spiking low Rinput - regular spiking
regular spiking low Rinput - regular spiking fAHP
regular spiking low Rinput - regular spiking high Rinput
regular spiking low Rinput - regular spiking large Vsag
log2(fold-change)
−1
0
1
fdr-BH corrected p-value
ns
p<0.05
p<0.01
p<0.001
resting membrane potential (mV)
input resistance (MOhm)
membrane time constant (ms)
ramp rate (mV/ms)
Vsag fraction (a.u.)
ramp rheobase (pA, 1st spike @ ramp)
downstroke rate (mV/ms, 1st spike)
fAHP (mV, 1st spike)
AHP (mV, 1st spike)
AHP (mV, 2nd spike)
latency (ms, 1st spike)
ISI (ms, 1st spike)
average ISI (ms)
ISI cv (σ/μ)
average spike rate (Hz)
adaptation index (rate ISI change)
spike latency (ms)
fraction short ISI (a.u.)
e
L2 burst spiking - L2 regular spiking
L2 burst spiking - L2 spike adapting
L2 regular spiking - L2 spike adapting
L3 burst spiking - L3 regular spiking
L3 burst spiking - L3 spike adapting
L3 regular spiking - L3 spike adapting
L5 burst spiking - L5 regular spiking
L5 burst spiking - L5 spike adapting
L5 regular spiking - L5 spike adapting
L6 burst spiking - L6 regular spiking
L6 burst spiking - L6 spike adapting
L6 regular spiking - L6 spike adapting
log2(fold-change)
−2.5
0
2.5
fdr-BH corrected p-value
ns
p<0.05
p<0.01
p<0.001
resting membrane potential (mV)
input resistance (MOhm)
membrane time constant (ms)
ramp rate (mV/ms)
Vsag fraction (a.u.)
ramp rheobase (pA, 1st spike @ ramp)
downstroke rate (mV/ms, 1st spike)
fAHP (mV, 1st spike)
AHP (mV, 1st spike)
AHP (mV, 2nd spike)
latency (ms, 1st spike)
ISI (ms, 1st spike)
average ISI (ms)
ISI cv (σ/μ)
average spike rate (Hz)
adaptation index (rate ISI change)
spike latency (ms)
fraction short ISI (a.u.)
f
L3 regular spiking - L3 spike adapting
L5 burst spiking - L5 regular spiking
L5 burst spiking - L5 spike adapting
L5 regular spiking - L5 spike adapting
L6 burst spiking - L6 regular spiking
L6 burst spiking - L6 spike adapting
L6 regular spiking - L6 spike adapting
log2(fold-change)
−2.5
0
2.5
fdr-BH corrected p-value
ns
p<0.05
p<0.01
p<0.001
length (μm)
height (μm)
branch order (nth)
fieldspan tuft (μm)
length 0 – 10% from pia (μm)
length 20 – 30% from pia (μm)
nodes within 10% from soma (#)
basal height (μm)
dendritic length (μm)
37.5 – 52.5%
22.5 – 37.5%
7.5 – 22.5%
-7.5 – -22.5%
% from soma (μm)
dendritic length
apical

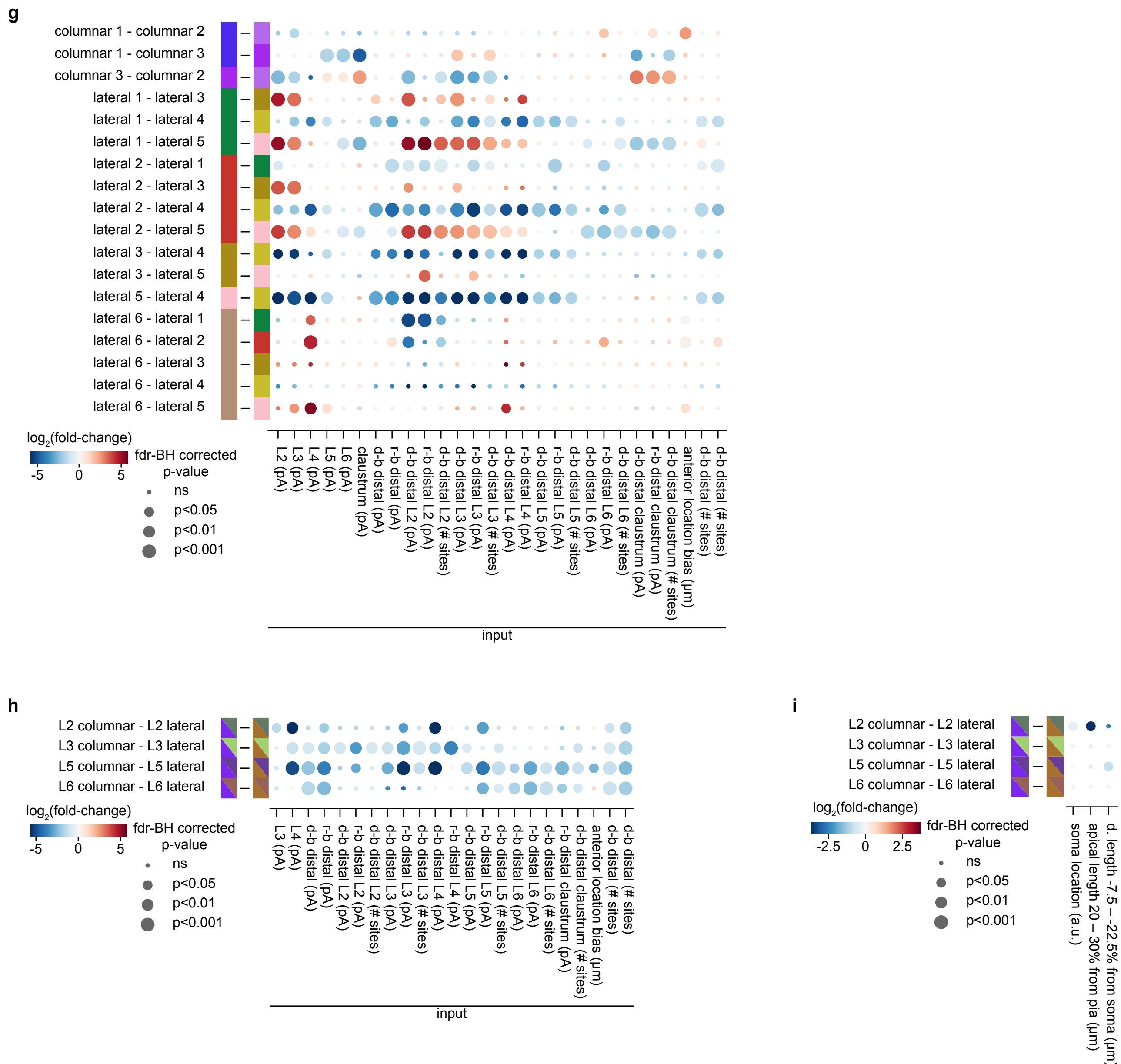
g
columnar 1 - columnar 2
columnar 1 - columnar 3
columnar 3 - columnar 2
lateral 1 - lateral 3
lateral 1 - lateral 4
lateral 1 - lateral 5
lateral 2 - lateral 1
lateral 2 - lateral 3
lateral 2 - lateral 4
lateral 2 - lateral 5
lateral 3 - lateral 4
lateral 3 - lateral 5
lateral 5 - lateral 4
lateral 6 - lateral 1
lateral 6 - lateral 2
lateral 6 - lateral 3
lateral 6 - lateral 4
lateral 6 - lateral 5
log2(fold-change)
-5
0
5
fdr-BH corrected p-value
ns
p<0.05
p<0.01
p<0.001
L2 (pA)
L3 (pA)
L4 (pA)
L5 (pA)
L6 (pA)
claustrum (pA)
d-b distal (pA)
r-b distal (pA)
d-b distal L2 (pA)
r-b distal L2 (pA)
d-b distal L2 (# sites)
d-b distal L3 (pA)
r-b distal L3 (pA)
d-b distal L3 (# sites)
d-b distal L4 (pA)
r-b distal L4 (pA)
d-b distal L5 (pA)
r-b distal L5 (pA)
d-b distal L5 (# sites)
d-b distal L6 (pA)
r-b distal L6 (pA)
d-b distal L6 (# sites)
d-b distal claustrum (pA)
r-b distal claustrum (pA)
d-b distal claustrum (# sites)
anterior location bias (μm)
d-b distal (# sites)
d-b distal (# sites)
input
h
L2 columnar - L2 lateral
L3 columnar - L3 lateral
L5 columnar - L5 lateral
L6 columnar - L6 lateral
log2(fold-change)
-5
0
5
fdr-BH corrected p-value
ns
p<0.05
p<0.01
p<0.001
L3 (pA)
L4 (pA)
d-b distal (pA)
r-b distal (pA)
d-b distal L2 (pA)
r-b distal L2 (pA)
d-b distal L2 (# sites)
d-b distal L3 (pA)
r-b distal L3 (pA)
d-b distal L3 (# sites)
d-b distal L4 (pA)
r-b distal L4 (pA)
d-b distal L5 (pA)
r-b distal L5 (pA)
d-b distal L5 (# sites)
d-b distal L6 (pA)
r-b distal L6 (pA)
d-b distal L6 (# sites)
r-b distal claustrum (pA)
d-b distal claustrum (# sites)
anterior location bias (μm)
d-b distal (# sites)
d-b distal (# sites)
input
i
L2 columnar - L2 lateral
L3 columnar - L3 lateral
L5 columnar - L5 lateral
L6 columnar - L6 lateral
log2(fold-change)
-2.5
0
2.5
fdr-BH corrected p-value
ns
p<0.05
p<0.01
p<0.001
soma location (a.u.)
apical length 20 – 30% from pia (μm)
d. length -7.5 – -22.5% from soma (μm)

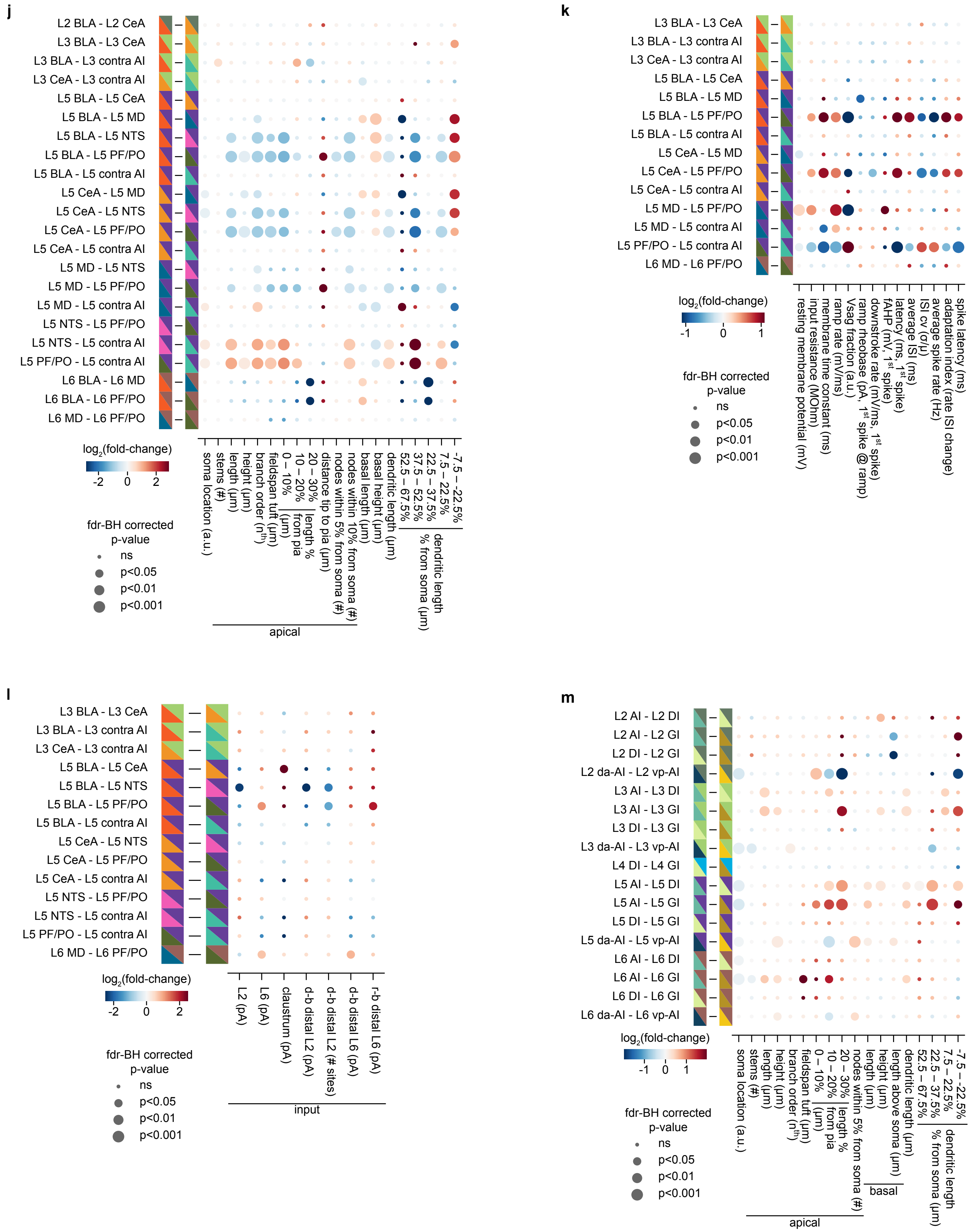
j
L2 BLA - L2 CeA
L3 BLA - L3 CeA
L3 BLA - L3 contra AI
L3 CeA - L3 contra AI
L5 BLA - L5 CeA
L5 BLA - L5 MD
L5 BLA - L5 NTS
L5 BLA - L5 PF/PO
L5 BLA - L5 contra AI
L5 CeA - L5 MD
L5 CeA - L5 NTS
L5 CeA - L5 PF/PO
L5 CeA - L5 contra AI
L5 MD - L5 NTS
L5 MD - L5 PF/PO
L5 MD - L5 contra AI
L5 NTS - L5 PF/PO
L5 NTS - L5 contra AI
L5 PF/PO - L5 contra AI
L6 BLA - L6 MD
L6 BLA - L6 PF/PO
L6 MD - L6 PF/PO
log2(fold-change)
-2 0 2
fdr-BH corrected p-value
ns
p<0.05
p<0.01
p<0.001
soma location (a.u.)
stems (#)
length (μm)
height (μm)
branch order (nth)
fieldspan tuft (μm)
0 – 10%
10 – 20%
20 – 30%
length % from pia (μm)
distance tip to pia (μm)
nodes within 5% from soma (#)
nodes within 10% from soma (#)
basal length (μm)
basal height (μm)
dendritic length (μm)
52.5 – 67.5%
37.5 – 52.5%
22.5 – 37.5%
7.5 – 22.5%
-7.5 – -22.5%
dendritic length % from soma (μm)
apical
k
L3 BLA - L3 CeA
L3 BLA - L3 contra AI
L3 CeA - L3 contra AI
L5 BLA - L5 CeA
L5 BLA - L5 MD
L5 BLA - L5 PF/PO
L5 BLA - L5 contra AI
L5 CeA - L5 MD
L5 CeA - L5 PF/PO
L5 CeA - L5 contra AI
L5 MD - L5 PF/PO
L5 MD - L5 contra AI
L5 PF/PO - L5 contra AI
L6 MD - L6 PF/PO
log2(fold-change)
-1 0 1
fdr-BH corrected p-value
ns
p<0.05
p<0.01
p<0.001
resting membrane potential (mV)
input resistance (MOhm)
membrane time constant (ms)
ramp rate (mV/ms)
Vsag fraction (a.u.)
ramp rheobase (pA, 1st spike @ ramp)
downstroke rate (mV/ms, 1st spike)
fAHP (mV, 1st spike)
latency (ms, 1st spike)
average ISI (ms)
ISI cv (σ/μ)
average spike rate (Hz)
adaptation index (rate ISI change)
spike latency (ms)
l
L3 BLA - L3 CeA
L3 BLA - L3 contra AI
L3 CeA - L3 contra AI
L5 BLA - L5 CeA
L5 BLA - L5 NTS
L5 BLA - L5 PF/PO
L5 BLA - L5 contra AI
L5 CeA - L5 NTS
L5 CeA - L5 PF/PO
L5 CeA - L5 contra AI
L5 NTS - L5 PF/PO
L5 NTS - L5 contra AI
L5 PF/PO - L5 contra AI
L6 MD - L6 PF/PO
log2(fold-change)
-2 0 2
fdr-BH corrected p-value
ns
p<0.05
p<0.01
p<0.001
L2 (pA)
L6 (pA)
claustrum (pA)
d-b distal L2 (pA)
d-b distal L2 (# sites)
d-b distal L6 (pA)
r-b distal L6 (pA)
input
m
L2 AI - L2 DI
L2 AI - L2 GI
L2 DI - L2 GI
L2 da-AI - L2 vp-AI
L3 AI - L3 DI
L3 AI - L3 GI
L3 DI - L3 GI
L3 da-AI - L3 vp-AI
L4 DI - L4 GI
L5 AI - L5 DI
L5 AI - L5 GI
L5 DI - L5 GI
L5 da-AI - L5 vp-AI
L6 AI - L6 DI
L6 AI - L6 GI
L6 DI - L6 GI
L6 da-AI - L6 vp-AI
log2(fold-change)
-1 0 1
fdr-BH corrected p-value
ns
p<0.05
p<0.01
p<0.001
soma location (a.u.)
stems (#)
length (μm)
height (μm)
branch order (nth)
fieldspan tuft (μm)
0 – 10%
10 – 20%
20 – 30%
length % from pia (μm)
nodes within 5% from soma (#)
length (μm)
height (μm)
length above soma (μm)
basal
dendritic length (μm)
52.5 – 67.5%
22.5 – 37.5%
7.5 – 22.5%
-7.5 – -22.5%
dendritic length % from soma (μm)
apical

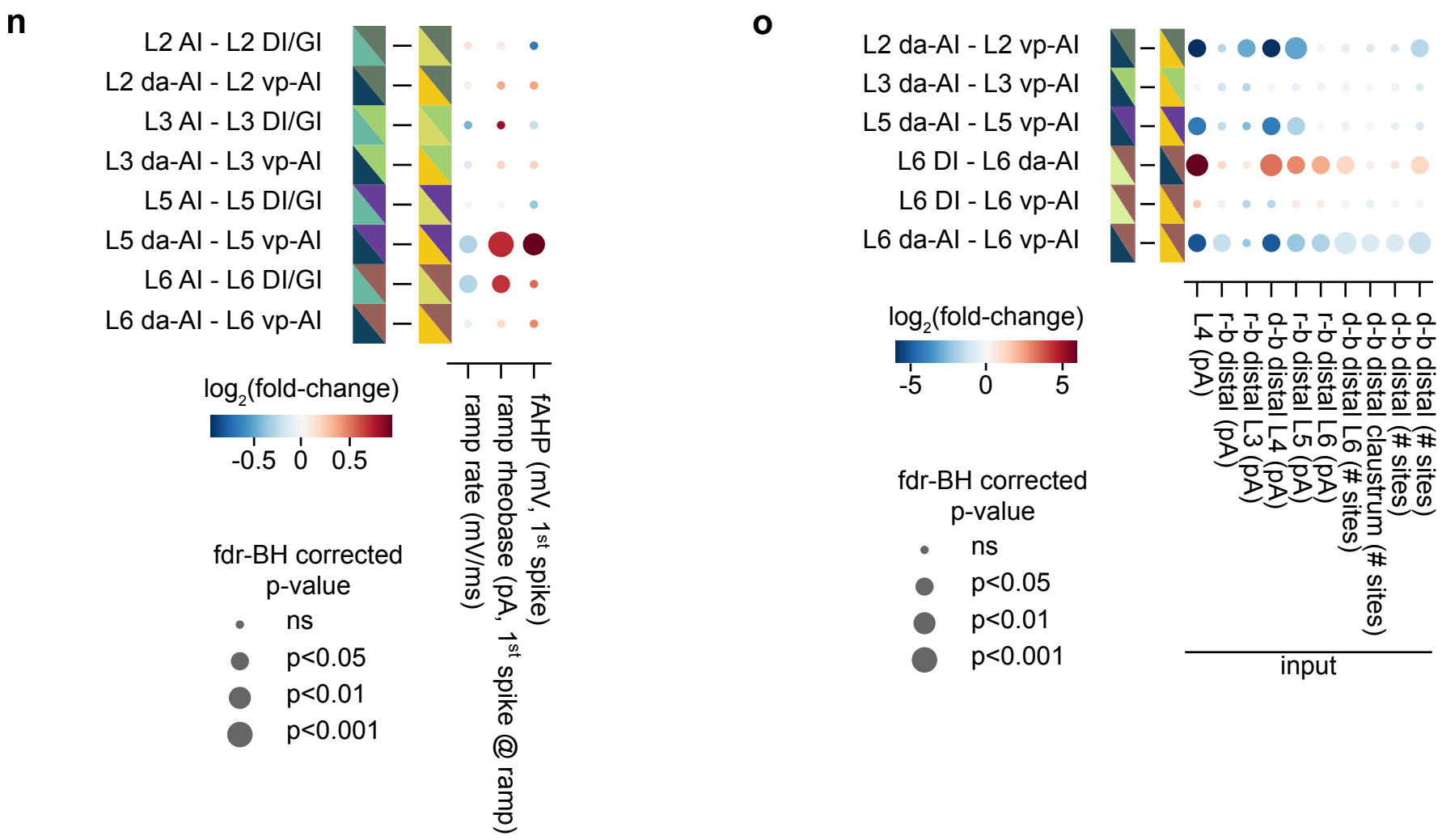

Supplementary Fig. 11: Informative features for pair-wise comparisons between cell types across all modalities. a–o, Pair-wise cross-modal feature comparisons between cell types (n ≥ 3 cells per type) of morphological (**a–c**), electrical (**d–f**), input (**g–l**), and topographical (**m–o**) modalities. In each row, the left and right cell types are color-coded as defined earlier (Figs. 1c, 2a, 3b, 3c, 4a, and 5d). For cell types based on the intersection of two modalities (**h–o**), e.g., layer allocation and input class (**h**), colors corresponding to each modality are shown in triangles. All features used for the iCCA are included (Extended Data Figs. 4a, 6b, and 8b). Bubble plots show fold difference calculated as $\log_2$ of mean feature value of the left cell type divided by that of the right cell type. Red luminance: the mean value of the left cell type > mean value of the right cell type; blue luminance: the reverse. All comparisons were statistically tested using a Mann-Whitney U two-sided test with false-discovery rate Benjamini-Hochberg corrections (fdr-BH corr.), indicated by four bubble sizes. Only features with a significant difference between any cell type pair are shown. For distal input features, the distal input border was defined by boundaries based on either 95% dendritic density (density-based, d-b) or 95% maximum dendritic reach (reach-based, r-b). Number of observations and exact p-values are provided in Supplementary Table 16.

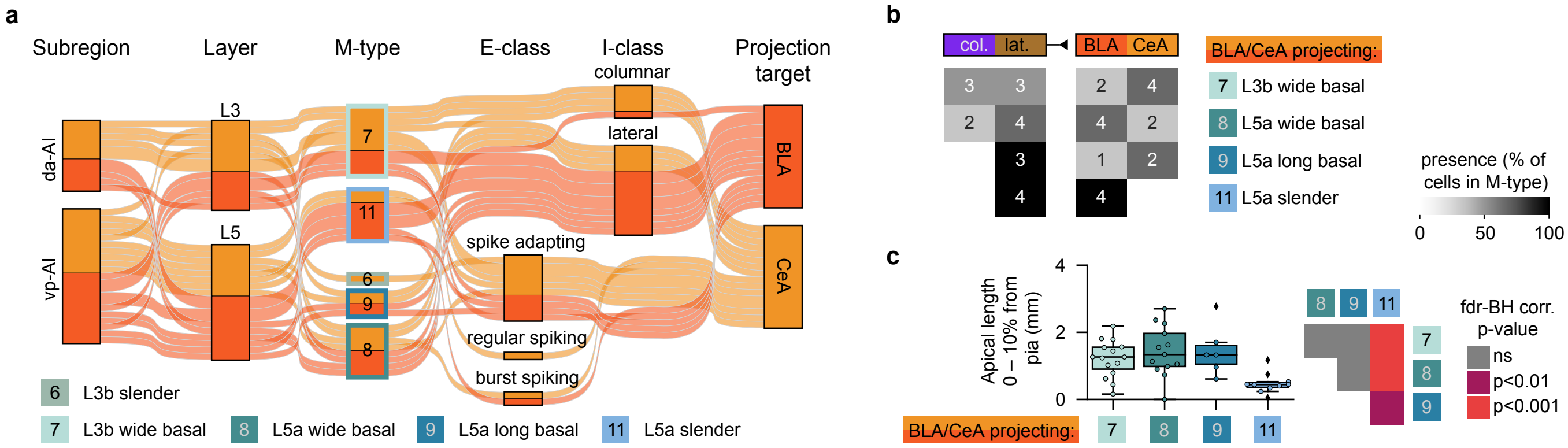

Supplementary Fig. 12: Informative features for cell type identification. **a**, Parallel categories diagram of L3b and L5a amygdala-projecting neurons and their allocations to subregions, M-types, E-classes, and I-classes. **b**, Contingency table of the same subset of M-types in (**a**) versus input classes or projection targets. Grayscale luminance: the percentage of cells within each M-type allocated to a given input class or projection target. Numbers in gray boxes: n of observed cells (when n > 0). **c**, Quantification of apical length within 10% cortical thickness bin from pia (left) and the corresponding heatmap of p-values from the pair-wise statistical comparisons between the same subset of M-types in (**a**) (right, Kruskal-Wallis test H (3) = 14.08, p = 0.003, with post hoc Dunn's two-sided comparisons with false-discovery rate Benjamini-Hochberg correction (fdr-BH corr.)). Circles: individual observations. Diamonds: outliers. Number of observations (n) for all groups and exact p-values provided in Supplementary Table 17.

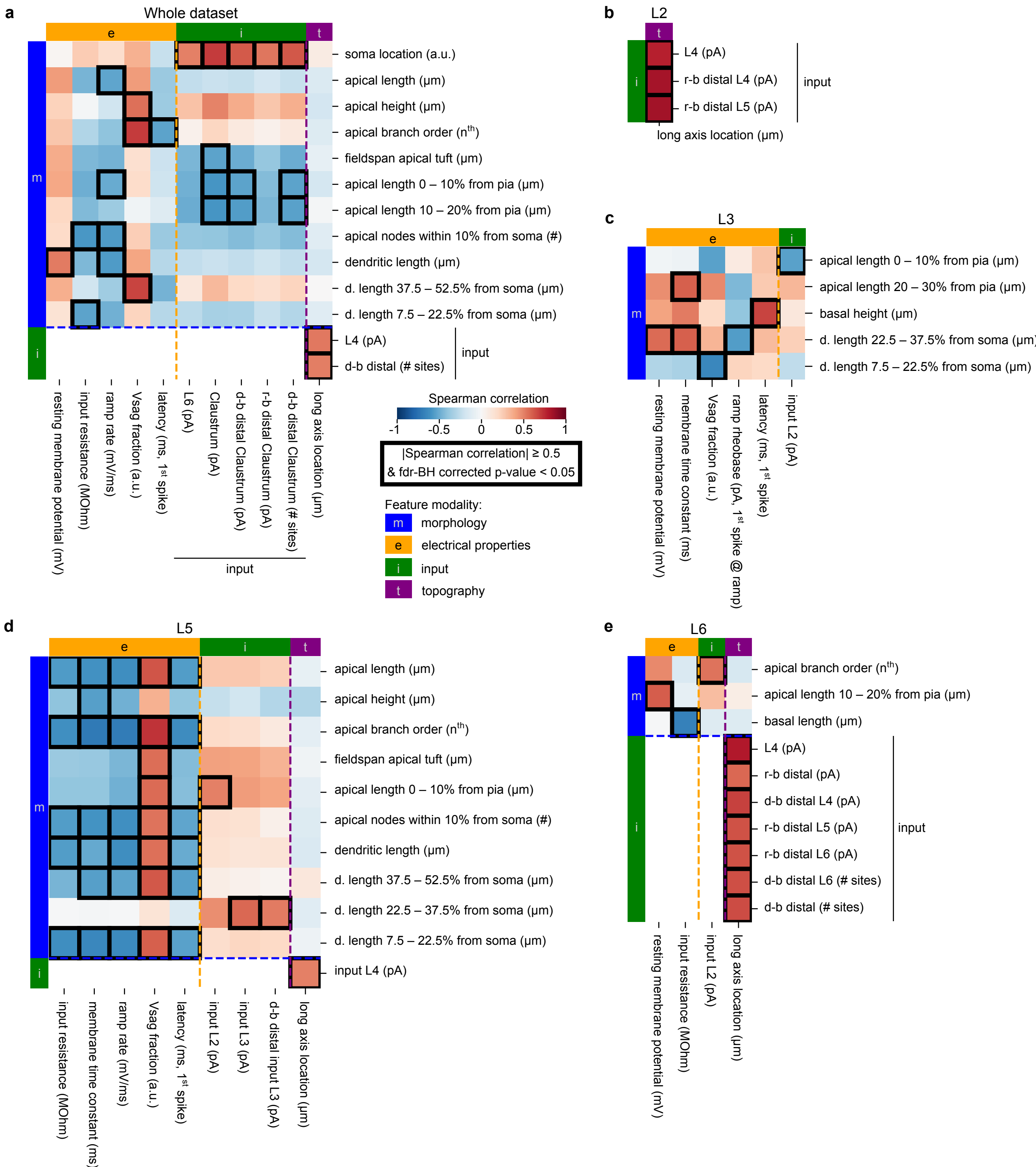

Supplementary Fig. 13: Cross-modal feature correlations. a–**e**, Feature pair-wise Spearman correlation between morphology (m, blue), electrical (e, yellow), input (i, green), and topographic (t, purple) features. Spearman correlations were computed based on the whole dataset (**a**), or subsets based on layer allocation of

the individual cells (**b**–**e**). Note that topology (t, long axis location) correlated with input (i), but not morphology (m) or electrical (e), features in the whole dataset and layer subsets (L2, L5, and L6) (**a**, **b**, **d**, and **e**). Feature pairs with an absolute Spearman correlation ≥ 0.5 and false-discovery rate Benjamini-Hochberg (fdr-BH) corrected p-value < 0.05 are boxed. Only feature pairs yielding significant correlations between any two modalities are shown (Methods, whole dataset: n = 25, L2: n = 3, L3: n = 7, L5: n = 36, L6: n = 10). For lateral input features, the lateral border was defined by boundaries based on either 95% dendritic density (density-based, d-b) or 95% maximum dendritic reach (reach-based, r-b).

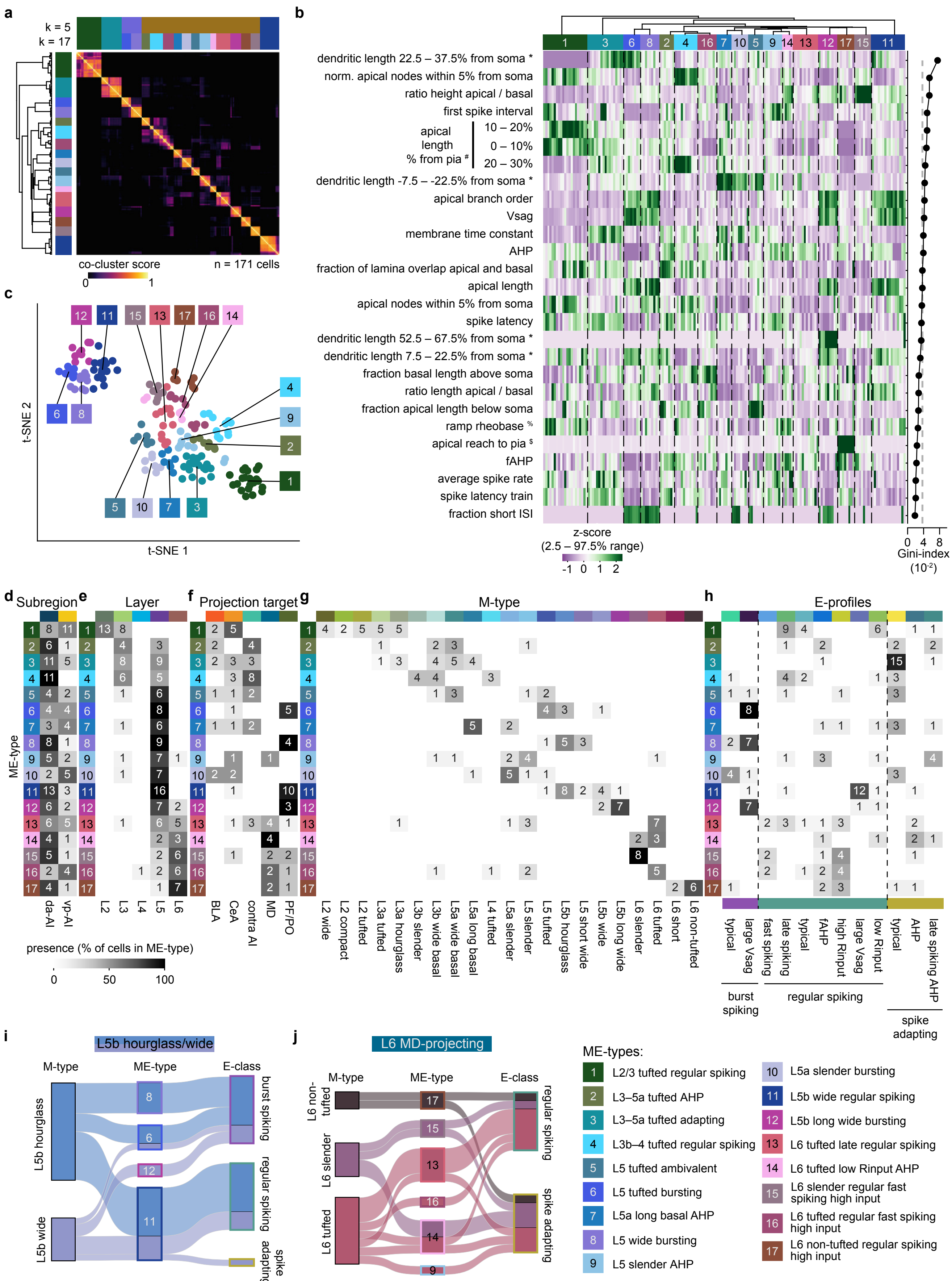
a
k = 5
k = 17
co-cluster score
n = 171 cells
0
1
b
dendritic length 22.5 – 37.5% from soma *
norm. apical nodes within 5% from soma
ratio height apical / basal
first spike interval
apical length % from pia #
10 – 20%
0 – 10%
20 – 30%
dendritic length -7.5 – -22.5% from soma *
apical branch order
Vsag
membrane time constant
AHP
fraction of lamina overlap apical and basal
apical length
apical nodes within 5% from soma
spike latency
dendritic length 52.5 – 67.5% from soma *
dendritic length 7.5 – 22.5% from soma *
fraction basal length above soma
ratio length apical / basal
fraction apical length below soma
ramp rheobase %
apical reach to pia $
fAHP
average spike rate
spike latency train
fraction short ISI
z-score (2.5 – 97.5% range)
-1 0 1 2
0 4 8
Gini-index (10^-2)
c
t-SNE 2
t-SNE 1
d Subregion
e Layer
f Projection target
g M-type
h E-profiles
ME-type
da-AI
vp-AI
L2
L3
L4
L5
L6
BLA
CeA
contra AI
MD
PF/PO
L2 wide
L2 compact
L2 tufted
L3a tufted
L3a hourglass
L3b slender
L3b wide basal
L5a wide basal
L5a long basal
L4 tufted
L5a slender
L5 slender
L5 tufted
L5b hourglass
L5 short wide
L5b wide
L5b long wide
L6 slender
L6 tufted
L6 short
L6 non-tufted
typical
large Vsag
fast spiking
late spiking
typical
fAHP
high Rinput
large Vsag
low Rinput
typical
AHP
late spiking AHP
burst spiking
regular spiking
spike adapting
presence (% of cells in ME-type)
0
50
100
i
L5b hourglass/wide
M-type
ME-type
E-class
L5b hourglass
L5b wide
burst spiking
regular spiking
spike adapting
j
L6 MD-projecting
L6 non-tufted
L6 slender
L6 tufted
ME-types:
1 L2/3 tufted regular spiking
2 L3–5a tufted AHP
3 L3–5a tufted adapting
4 L3b–4 tufted regular spiking
5 L5 tufted ambivalent
6 L5 tufted bursting
7 L5a long basal AHP
8 L5 wide bursting
9 L5 slender AHP
10 L5a slender bursting
11 L5b wide regular spiking
12 L5b long wide bursting
13 L6 tufted late regular spiking
14 L6 tufted low Rinput AHP
15 L6 slender regular fast spiking high input
16 L6 tufted regular fast spiking high input
17 L6 non-tufted regular spiking high input

Supplementary Fig. 14: Analysis of combined morpho-electrical features and subsequent cell groups. **a**, Hierarchical cluster analysis (HCA) on pair-wise co-cluster scores across 171 cells using both morphological and electrical features. Dendrogram-based grouping is color-coded for 17 clusters (k = 17) based on optimal silhouette score, as well as for 5 clusters (k = 5). **b**, Z-scored electrical and morphological features (left), ranked by random forest classifier Gini-index (right, gray dashed line indicates equal feature contribution [1/27 features]). *: Dendritic length located within a 15% local cortical thickness bin positioned relative to the soma, normalized to local cortical thickness. $^{\#}$: Apical length within a 10% local cortical thickness bin positioned relative to the pia, normalized to total apical length. $^{\%}$: Current required to elicit the first action potential during ramping depolarizing current injection. $^{\$}$: Vertical distance between pia and closest apical dendrite, normalized to local cortical thickness. **c**, t-SNE distribution of morpho-electrical types (ME-types). For (**b** and **c**), colored boxes with numbers: ME-types color-coded for k = 17 in (**a**). **d**–**h**, Contingency table of cell allocations to subregion (**d**), layer (**e**), projection target (**f**), M-type (**g**), or E-profile (**h**). Note in (**f**), most ME-types only contain IT (e.g., BLA-, CeA-, or contra AI-projecting) or ET (e.g., MD- or PF/PO thalamus-projecting) neurons, suggesting IT and ET neurons (Fig. 6f) can be readily resolved using ME-types. Grayscale luminance: the percentage of cells in each ME-type allocated to a given modality. Numbers in gray boxes: n of observed cells (when n > 0). **i** and **j**, Parallel categories diagrams of *L5b hourglass* (M-type 14) and *L5b wide* (M-type 16) neurons (**i**; n = 23 cells) and L6 MD-projecting neurons (**j**; n = 14 cells) across M-types, ME-types, and E-classes. Several M-types and E-classes can be sub-specified into multiple ME-types. ME-type legend (bottom right) is shared across all panels.

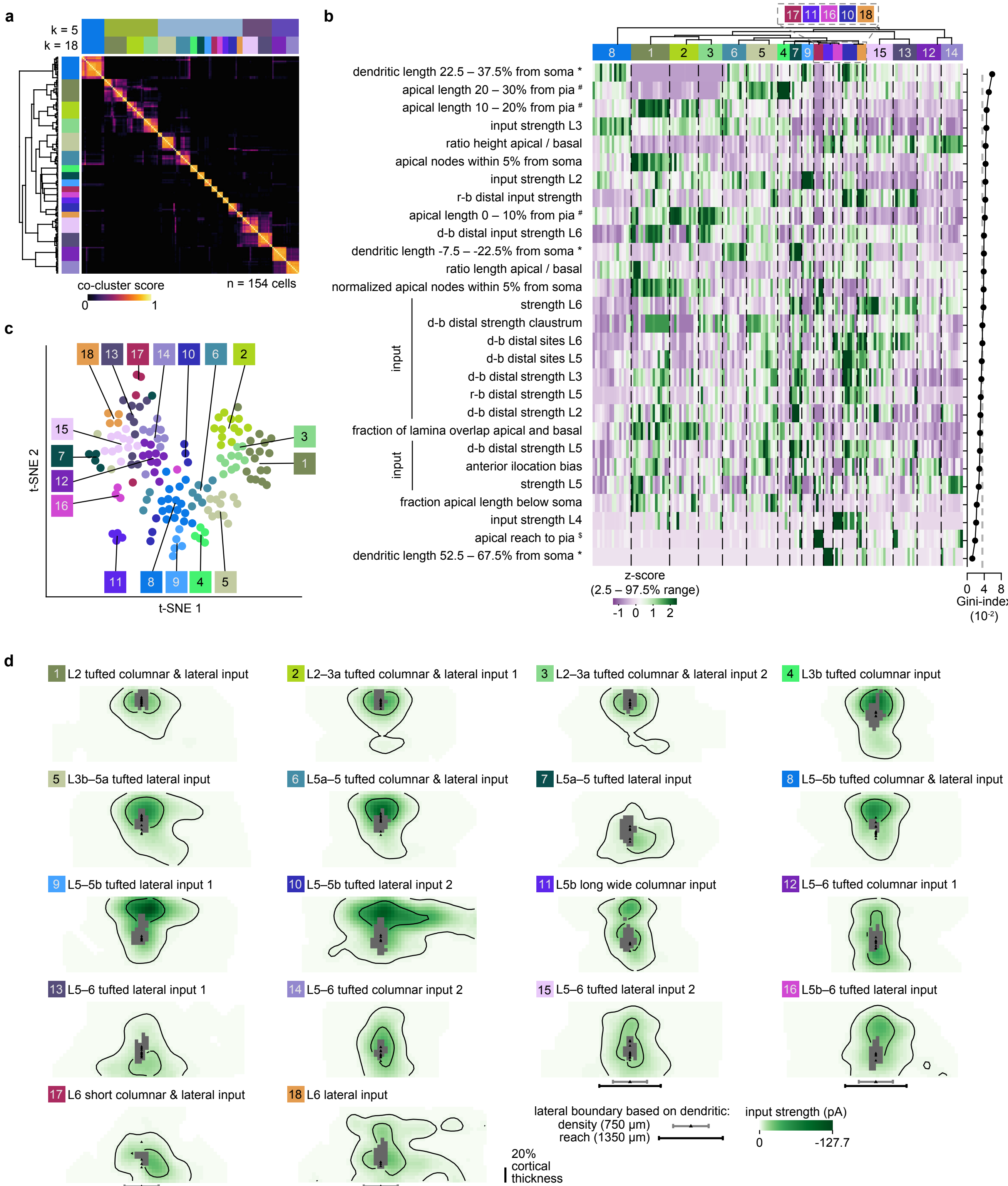
a
k = 5
k = 18
co-cluster score
0
1
n = 154 cells
b
17 11 16 10 18
8 1 2 3 6 5 4 7 9 15 13 12 14
dendritic length 22.5 – 37.5% from soma *
apical length 20 – 30% from pia #
apical length 10 – 20% from pia #
input strength L3
ratio height apical / basal
apical nodes within 5% from soma
input strength L2
r-b distal input strength
apical length 0 – 10% from pia #
d-b distal input strength L6
dendritic length -7.5 – -22.5% from soma *
ratio length apical / basal
normalized apical nodes within 5% from soma
input
strength L6
d-b distal strength claustrum
d-b distal sites L6
d-b distal sites L5
d-b distal strength L3
r-b distal strength L5
d-b distal strength L2
fraction of lamina overlap apical and basal
input
d-b distal strength L5
anterior ilocation bias
strength L5
fraction apical length below soma
input strength L4
apical reach to pia $
dendritic length 52.5 – 67.5% from soma *
z-score
(2.5 – 97.5% range)
-1 0 1 2
0 4 8
Gini-index
(10-2)
c
18 13 17 14 10 6 2
15
7
12
16
3
1
11 8 9 4 5
t-SNE 2
t-SNE 1
d
1 L2 tufted columnar & lateral input
2 L2–3a tufted columnar & lateral input 1
3 L2–3a tufted columnar & lateral input 2
4 L3b tufted columnar input
5 L3b–5a tufted lateral input
6 L5a–5 tufted columnar & lateral input
7 L5a–5 tufted lateral input
8 L5–5b tufted columnar & lateral input
9 L5–5b tufted lateral input 1
10 L5–5b tufted lateral input 2
11 L5b long wide columnar input
12 L5–6 tufted columnar input 1
13 L5–6 tufted lateral input 1
14 L5–6 tufted columnar input 2
15 L5–6 tufted lateral input 2
16 L5b–6 tufted lateral input
17 L6 short columnar & lateral input
18 L6 lateral input
lateral boundary based on dendritic:
density (750 μm)
reach (1350 μm)
input strength (pA)
0
-127.7
20%
cortical
thickness

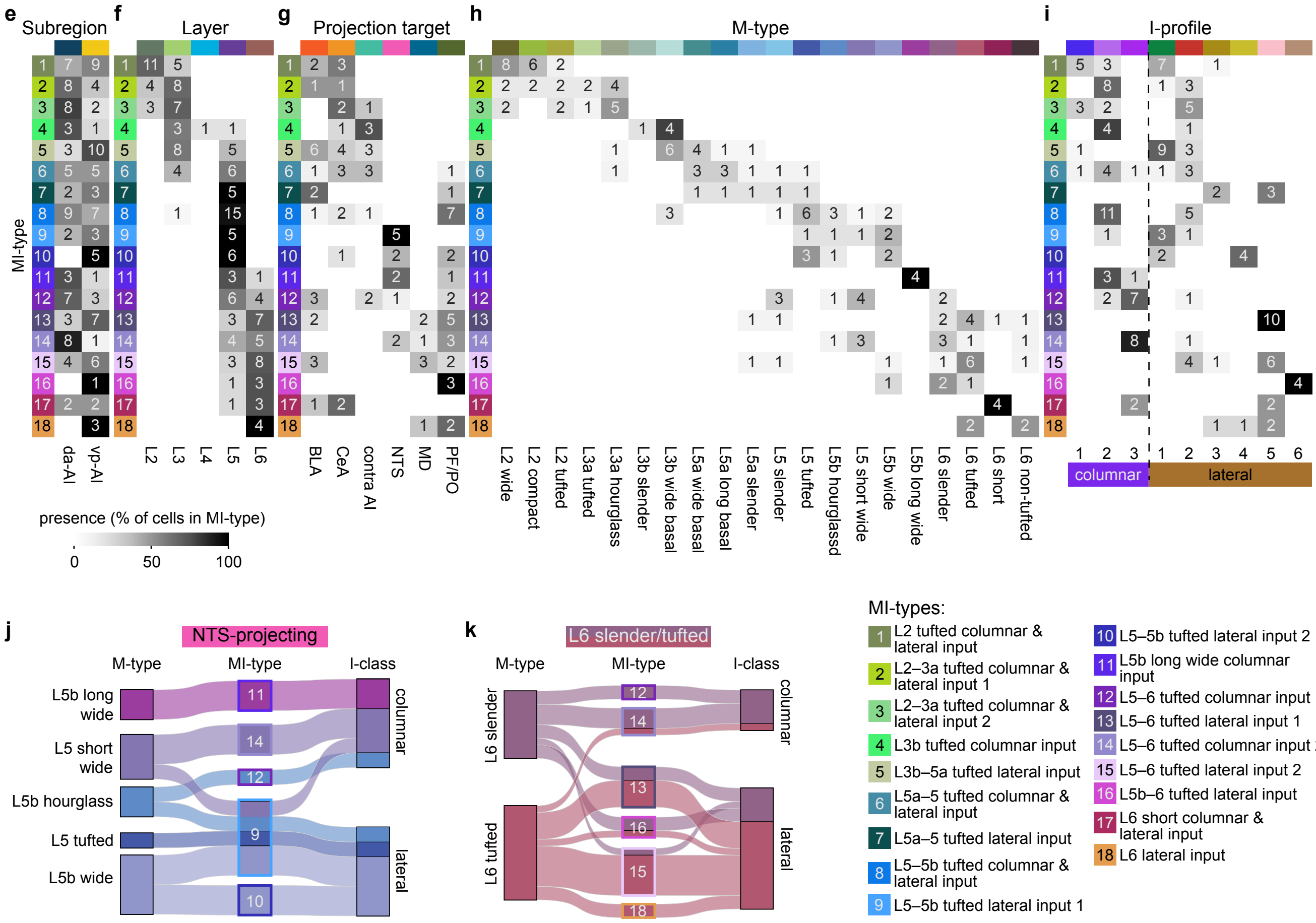

Supplementary Fig. 15: Analysis of combined morpho-input features and subsequent cell groups. a, Hierarchical cluster analysis (HCA) on pair-wise co-cluster scores across 154 cells using both morphological and input features. Dendrogram-based grouping is color-coded for 18 clusters (k = 18) based on optimal silhouette score, as well as for 5 clusters (k = 5). **b**, Z-scored morphological and input features (left), ranked by random forest classifier Gini-index (right, gray dashed line indicates equal feature contribution [1/28 features]). For distal input features, the border was defined by boundaries based on either 95% dendritic density (density-based, d-b) or 95% maximum dendritic reach, (reach-based, r-b). *: Dendritic length located within a 15% local cortical thickness bin positioned relative to the soma, normalized to local cortical thickness. $^{\#}$: Apical length within a 10% local cortical thickness bin positioned relative to the pia, normalized to total apical length. $^{\$}$: Vertical distance between pia and closest apical dendrite, normalized to local cortical thickness. **c**, t-SNE distribution of morpho-input types (MI-types). **d**, Average input maps grouped by MI-

types, aligned by pia, formatted as in Fig. 5b. Maps are vertically interpolated and normalized to the cortical thickness. Gray and black lateral whisker bars: lateral boundary thresholds, used during feature extraction, based on dendritic density (750 µm) or reach (1350 µm), respectively; gray pixels: direct response sites; black triangles: soma locations. For (**b**–**d**), colored boxes with numbers: MI-types color-coded for k = 18 in (**a**). **e**–**i**, Contingency tables of MI-types versus subregion (**e**), layer (**f**), projection target (**g**), M-type (**h**) and I-profile (**i**). Grayscale luminance: the percentage of cells in each MI-type allocated to a given aforementioned modality. Numbers in gray boxes: n of observed cells (when n > 0). **j**, Parallel categories diagrams of NTS-projecting neurons (n = 12). **k**, Parallel categories diagrams of *L6 slender* (M-type 18) and *L6 tufted* (M-type 19) neurons (n = 24). Note in (**i**–**k**), a majority of MI-types (12/18) retain predominance in either columnar or lateral input classes. MI-type legend (bottom right) is shared across all panels.

References

93 Magee, J. C. Dendritic lh normalizes temporal summation in hippocampal CA1 neurons. Nat Neurosci 2, 508–514 (1999). https://doi.org/10.1038/9158

94 Marder, E. Neuromodulation of neuronal circuits: back to the future. Neuron 76, 1–11 (2012). https://doi.org/10.1016/j.neuron.2012.09.010
