# Supplementary material for: Pyramidal cell types and circuit organization of the mouse insular cortex reveal functional specializations
Source: Nat Neurosci. 2026 Aug 7;29(10):2599–612. doi: 10.1038/s41593-026-02391-0 (PMC13633118; doi:10.1038/s41593-026-02391-0)
Supplement: Supplementary file 2 [file 41593_2026_2391_MOESM2_ESM.pdf]

nature portfolio

Corresponding author(s): Bart C. Jongbloets and Tianyi Mao

Last updated by author(s): June 18, 2026

Reporting Summary

Nature Portfolio wishes to improve the reproducibility of the work that we publish. This form provides structure for consistency and transparency in reporting. For further information on Nature Portfolio policies, see our Editorial Policies and the Editorial Policy Checklist.

Statistics

For all statistical analyses, confirm that the following items are present in the figure legend, table legend, main text, or Methods section.

n/a	Confirmed	
☐	☒	The exact sample size (*n*) for each experimental group/condition, given as a discrete number and unit of measurement
☐	☒	A statement on whether measurements were taken from distinct samples or whether the same sample was measured repeatedly
☐	☒	The statistical test(s) used AND whether they are one- or two-sided<br>*Only common tests should be described solely by name; describe more complex techniques in the Methods section.*
☒	☐	A description of all covariates tested
☐	☒	A description of any assumptions or corrections, such as tests of normality and adjustment for multiple comparisons
☐	☒	A full description of the statistical parameters including central tendency (e.g. means) or other basic estimates (e.g. regression coefficient) AND variation (e.g. standard deviation) or associated estimates of uncertainty (e.g. confidence intervals)
☐	☒	For null hypothesis testing, the test statistic (e.g. *F*, *t*, *r*) with confidence intervals, effect sizes, degrees of freedom and *P* value noted<br>*Give P values as exact values whenever suitable.*
☒	☐	For Bayesian analysis, information on the choice of priors and Markov chain Monte Carlo settings
☒	☐	For hierarchical and complex designs, identification of the appropriate level for tests and full reporting of outcomes
☒	☐	Estimates of effect sizes (e.g. Cohen's *d*, Pearson's *r*), indicating how they were calculated

Our web collection on statistics for biologists contains articles on many of the points above.

Software and code

Policy information about availability of computer code

Data collection	As described in Methods – Ephus acquisition software (Suter et al., 2010 Front. Neural Circuits), Neurolucida (RRID:SCR_001775; MBFBioscience), Zen software (RRID:SCR_013672; Zeiss).
Data analysis	As described in Methods – Custom code and software in R (v4.2, RRID:SCR_001905; https://cran.r-project.org), Python (v3.8 and v3.9, RRID:SCR_008394; https://www.python.org), Matlab (v2020a and v2022a, RRID:SCR_001622; https://www.mathworks.com/), FIJI (v2.14, RRID:SCR_002285; https://fiji.sc), L-Measure (v5.2, RRID:SCR_003487; http://cng.gmu.edu:8080/Lm/ and Python-version https://pypi.org/project/pylmeasure/), Photoshop (v23, RRID:SCR_014199), and HBP Neuron Morphology Viewer (https://neuroinformatics.nl/HBP/morphology-viewer/) were used to process and analyze the data and are made available on GitLab (https://gitlab.com/maolab/braincellsuite_submission and https://gitlab.com/maolab/mouseinsularatlas) and Zenodo (https://zenodo.org/records/20714121).

For manuscripts utilizing custom algorithms or software that are central to the research but not yet described in published literature, software must be made available to editors and reviewers. We strongly encourage code deposition in a community repository (e.g. GitHub). See the Nature Portfolio guidelines for submitting code & software for further information.

Data

Policy information about availability of data

All manuscripts must include a data availability statement. This statement should provide the following information, where applicable:

- Accession codes, unique identifiers, or web links for publicly available datasets
- A description of any restrictions on data availability
- For clinical datasets or third party data, please ensure that the statement adheres to our policy

All raw data supporting this study are deposited in format-appropriate repositories (BrainImageLibrary.org for Nissl images, NeuroMorpho.org for morphological reconstructions), and/or Zenodo (https://doi.org/10.5281/zenodo.20714121). Any additional information required to re-analyze the data reported in this paper is available from the corresponding authors upon request.

Research involving human participants, their data, or biological material

Policy information about studies with human participants or human data. See also policy information about sex, gender (identity/presentation), and sexual orientation and race, ethnicity and racism.

Reporting on sex and gender	The morphometric analysis of human pyramidal neurons in this manuscript was conducted on a publicly available dataset published by Kalmbach et al. (2021) Neuron (10.1016/j.neuron.2021.08.030). Descriptions of metadata are available in the original publication. No new information was obtained from human participants.
Reporting on race, ethnicity, or other socially relevant groupings	see above
Population characteristics	see above
Recruitment	see above
Ethics oversight	see above

Note that full information on the approval of the study protocol must also be provided in the manuscript.

Field-specific reporting

Please select the one below that is the best fit for your research. If you are not sure, read the appropriate sections before making your selection.

☒ Life sciences ☐ Behavioural & social sciences ☐ Ecological, evolutionary & environmental sciences

For a reference copy of the document with all sections, see nature.com/documents/nr-reporting-summary-flat.pdf

Life sciences study design

All studies must disclose on these points even when the disclosure is negative.

Sample size

As described in the Methods section – Nissl-based quantitative common reference framework: n = 3 out of 10 mice used for Nissl reconstruction. Quantification of retrograde fluorescent bead distribution (Fig. 3d,e): n = 31 mice. Multimodal database: n = 1,093 insular neurons* from 283 mice. M-type and I-profile classifier test datasets (Fig. 6i, Extended Data Fig. 5b, 9f): n = 17 Ent- / 12 NAc- / 8 NAc Fezf2+-projecting insular neurons from 7 / 6 / 6 mice. CRACM intra-insular connectivity (Fig. 8a–f): n = 23 insular neurons from 7 mice. CTA (Fig. 8h,i): n = 19 mice.

All n numbers align with or exceed sample sizes included in relevant published work (e.g. Gouwens et al., 2019; Xu et al., 2016). In addition, Extended Data Figure 3e confirms that we have reached a saturating sample size.

*Note that the total of 1,130 neurons mentioned in the manuscript includes: the multimodal database (1,093 neurons, including the CRACM intra-insular connectivity), the M-type and I-profile classifier test datasets (37 neurons).

Data exclusions

As described in the Methods sections – Nissl-based quantitative common reference framework: Stained brain slice series were excluded when parts of the insular cortex were damaged or missing. Quantification of retrograde fluorescent bead distribution (Fig. 3d,e): Bead expression was not analyzed when the injection center was off-target. Multimodal database: Dendritic morphology inclusion criteria: intact apical dendrites that extended all the way to the pial surface (except some L6 cell types), a dark biocytin fill that persisted to the distal parts of both apical and basal dendrites, and the presence of dendritic spines to confirm an excitatory neuronal identity. Electrophysiology recordings inclusion criteria: access resistance < 40 MOhm, action potential threshold ≤ -20 mV and amplitude ≥ 40 mV, and single action potential and spike train data were available. LSPS recordings inclusion criteria: minimum acquisition of 2 LSPS maps with access resistance < 45 MOhm and membrane resistance > ~90% of the total resistance. CRACM intra-insular connectivity (Fig. 8a–f): animal inclusion criteria: there must be detectable expression in the posterior insular cortex, and at least one of the neurons recorded showed response to posterior stimulation. CRACM recordings inclusion criteria: minimum acquisition of 2 CRACM maps with access resistance < 45 MOhm. CTA (Fig. 8h,i): Two mice

	from the tetanus toxin treatment group were excluded post hoc due to injections missing the target region(s). Two mice from the control group were excluded due to inability to adapt to the water deprivation paradigm.
Replication	Nissl and retrograde tracer quantifications were performed in replicates of 3 mice (n >= 3 mice per tracer). CRACM intra-insular connectivity (Fig. 8a–f) was performed in replicates of 7 mice.
Randomization	Multiple cells were obtained per mouse (median: 3 cells per mouse, range: 1 – 13, Supplementary Table 6), sample conditions were randomized for experiments. For the bead or channelrhodopsin experiments, no randomization scheme was used to determine which animals received stereotaxic injections and which brain region(s) of interest were injected. For CTA experiments, male and female animals from the same litters were randomly assigned to control or tetanus-toxin groups.
Blinding	Experimenters assessing CTA behavior were blinded to treatment group assignment throughout the experiment. For other experiments, the experimenters were not blind to conditions (injection location, genotype, sex) because the same experimenters were responsible for tracking animal and surgery information. Care was taken to avoid potential bias associated with the knowledge of conditions, as conclusions did not depend on subjective scoring and were reached quantitatively after completing all data acquisition and post hoc analysis.

Reporting for specific materials, systems and methods

We require information from authors about some types of materials, experimental systems and methods used in many studies. Here, indicate whether each material, system or method listed is relevant to your study. If you are not sure if a list item applies to your research, read the appropriate section before selecting a response.

Materials & experimental systems

n/a	Involved in the study
☐	☒ Antibodies
☒	☐ Eukaryotic cell lines
☒	☐ Palaeontology and archaeology
☐	☒ Animals and other organisms
☒	☐ Clinical data
☒	☐ Dual use research of concern
☒	☐ Plants

Methods

n/a	Involved in the study
☒	☐ ChIP-seq
☒	☐ Flow cytometry
☒	☐ MRI-based neuroimaging

Antibodies

Antibodies used	Mouse anti-parvalbumin monoclonal antibody (P3088, RRID:AB_477329, Sigma-Aldrich). Peroxidase AffiniPure Goat anti-mouse IgG (H+L) (#115-035-003, RRID:AB_10015289, Jackson ImmunoResearch).
Validation	Antibody specificity has been tested in PV-Cre;Ai9 mice in Melander et al. (2021) Cell Reports (DOI: 10.1016/j.cellrep.2021.109972).

Animals and other research organisms

Policy information about studies involving animals; ARRIVE guidelines recommended for reporting animal research, and Sex and Gender in Research

Laboratory animals	C57BL/6 mice (CR, RRID:IMSR_CR:027) and PV-Cre (Jax, RRID:IMSR_JAX:008069), Sst-Cre (Jax, RRID:IMSR_JAX:013044), Gad2-Cre (Jax, RRID:IMSR_JAX:010802), Drd1-EGFP (Gensat, RRID:MMRRC_000296-MU), Fezf2-CreER (Jax, RRID:IMSR_JAX:036296), Dat-Cre (Jax, RRID:IMSR_JAX:006660), Ai32 (Jax, RRID:IMSR_JAX:012569), and Ai9 (Jax, RRID:IMSR_JAX:007909) mice—all on a C57BL/6 background—were used, see also Supplementary Tables 4 and 5. Jax, The Jackson Laboratory; CR, Charles River.<br>For electrophysiology experiments, P21-P107 animals were used (for the morphology dataset, median = P40; for the intrinsic properties dataset, median = P38; for the connectivity dataset, median = P38). For the Nissl-based anatomy dataset, P48-50 animals were used.
Wild animals	The study did not involve wild animals
Reporting on sex	Both sexes (168 females / 194 males) were included in the experimental design and their use per genotype is reported in the Methods and Supplementary Table 5.
Field-collected samples	The study did not involve samples collected from the field
Ethics oversight	All procedures were approved by the Oregon Health & Science University Institutional Animal Care and Use Committee (IACUC).

Note that full information on the approval of the study protocol must also be provided in the manuscript.

Plants

Seed stocks	This study did not involve plant samples
Novel plant genotypes	This study did not involve plant samples
Authentication	This study did not involve plant samples
